# Enamine Synthesis via Regiocontrolled 6‐*endo*‐*dig* and 5‐*exo*‐*dig* Tethered Carboamination of Propargylic Alcohols

**DOI:** 10.1002/anie.202411383

**Published:** 2024-10-24

**Authors:** Helena Solé‐Àvila, Mikus Puriņš, Lucas Eichenberger, Jerome Waser

**Affiliations:** ^1^ Laboratory of Catalysis and Organic Synthesis and NCCR Catalysis Institut des Sciences et Ingénierie Chimique École Polytechnique Fédérale de Lausanne 1015 Lausanne Switzerland

**Keywords:** amino alcohol, carboamination, enamine, palladium catalysis, regioselective

## Abstract

Enamines are versatile building blocks for the synthesis of biologically active compounds. Nevertheless, only a limited number of strategies have been reported for preparing trisubstituted enamines in a regio‐ and stereoselective manner. Herein, we report a regiocontrolled 6‐*endo* and 5‐*exo* tethered carboamination of propargylic alcohols for the synthesis of trisubstituted enamines. High regioselectivity was achieved through fine‐tuning of the amine protecting group during the Pd‐catalyzed carboamination. The introduced trifluoromethylated tether enables further stereoselective functionalizations, such as hydrogenation and fluorination.

## Graphical abstract

The regiocontrolled synthesis of enamines was achieved by the Pd‐catalyzed carboamination of propargylic alcohols using molecular aldimine tethers. 5‐*exo* and 6‐*endo* cyclic trisubstituted enamines were selectively prepared based on the choice of the amine protecting group. The obtained enamines were easily transformed in 2,1‐ and 3,1‐amino alcohols and their fluorinated derivatives.

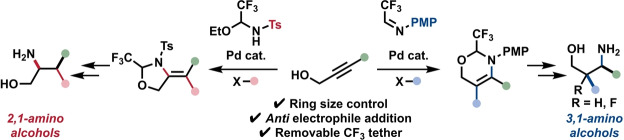

Heteroatom‐containing compounds are ubiquitous in natural products and therapeutics.[^1^, ^2^] Therefore, heteroatom‐rich building blocks are essential to synthesize new bioactive compounds. Enamines are powerful synthetic intermediates first popularized by Stork that have gained prominence with the discovery of organocatalysis.[^3^, ^4^, ^5^, ^6^] Enamines have been broadly applied in the context of traditional polar reactivity, as well as in radical addition reactions.[^7^, ^8^, ^9^] These compounds are often accessed from carbonyl compounds bearing enolizable α hydrogens (Scheme 1A, eq. 1). However, with unbiased substrates, a mixture of regioisomeric enamines is usually formed under thermodynamic control. In addition, the geometry of the double bond can be challenging to control especially for trisubstituted enamines. Another method for preparing enamines involves palladium or copper‐catalyzed C−N cross‐coupling reactions (Scheme 1A, eq. 2).[^10^, ^11^, ^12^, ^13^] However, there are only few examples for the preparation of highly substituted enamines. An alternative disconnection towards trisubstituted enamines is the carboamination of alkynes (Scheme 1A, eq. 3).[^14^, ^15^, ^16^, ^17^, ^18^, ^19^, ^20^, ^21^, ^22^, ^23^, ^24^] In this approach, the *E*/*Z* geometry of the final product can usually be more easily controlled. However, for unbiased or unactivated substrates, the regioselectivity of difunctionalization reactions remains difficult to control. More recently, the difunctionalization of ynamides under Pd catalysis was also reported for the synthesis of trisubstituted enamides (Scheme 1A, eq. 4).[^25^, ^26^] However, the starting materials were usually not commercially available and preserving the integrity of the nitrogen atom in subsequent transformations was difficult to achieve.

**Scheme 1:**
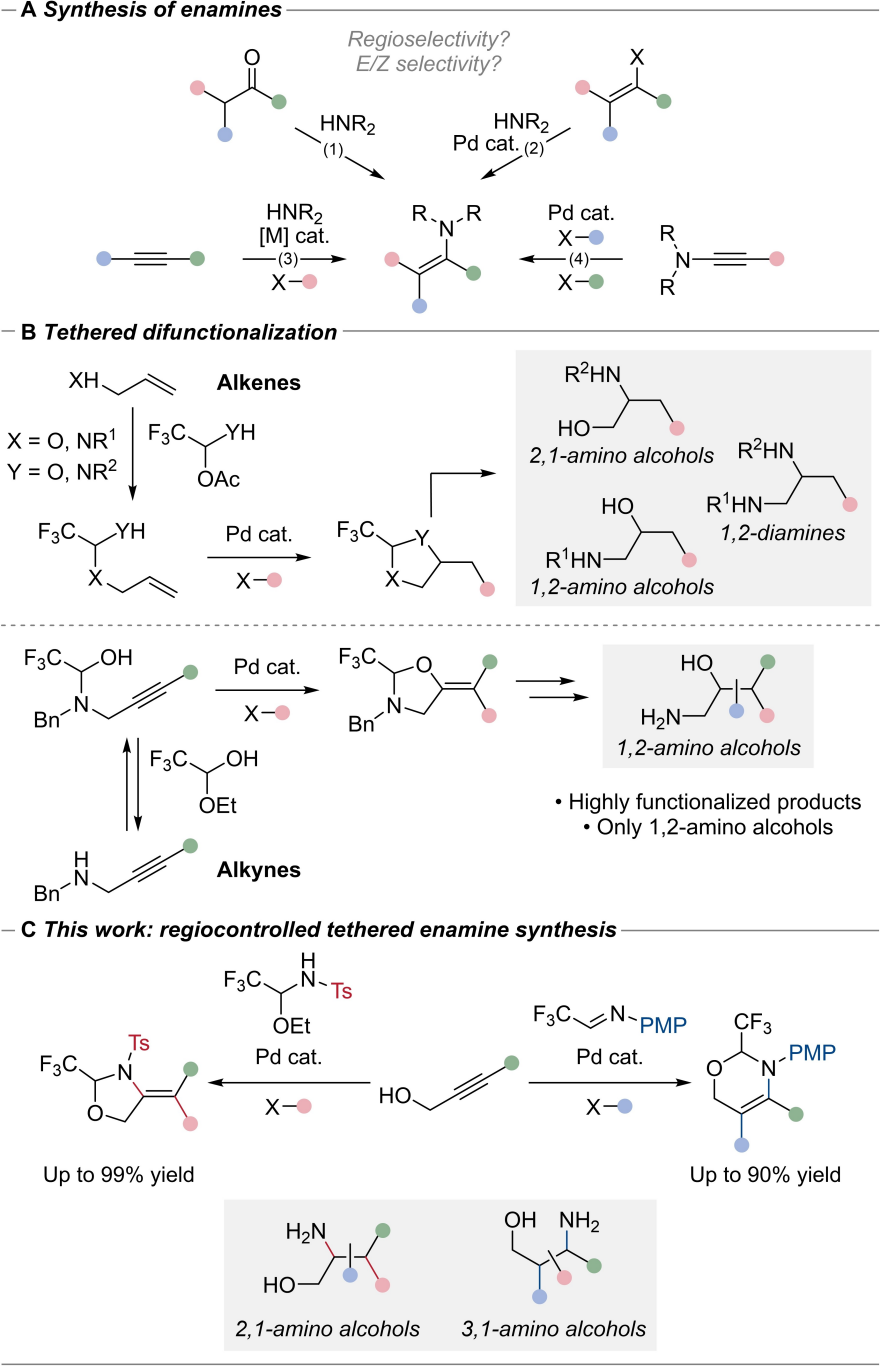
A) Synthesis of trisubstituted enamines. B) Tethered intramolecular difunctionalization of alkenes and alkynes: synthesis of 1,2‐amino alcohols. C) This work: regioselective 5‐*exo* and 6‐*endo* tethered synthesis of enamines.

To answer the challenge of the regio‐ and stereo‐selective difunctionalization of π bonds, our group has first applied the tethering concept to Pd‐catalyzed reactions of alkenes (Scheme 1B).[^27^, ^28^, ^29^, ^30^] In this approach, the reacting nucleophile is first installed onto an existing functionality of the substrate via the formation of an acetal. This achieves temporary intramolecularity and discriminates between the different possible regioisomers via ring size constraint. The molecular tether can then be cleaved to access 1,2‐ and 2,1‐amino alcohols as well as diamines, motifs present in many bioactive compounds.[^31^, ^32^, ^33^] However, this approach works only in the case of β‐unsubstituted alkenes, resulting in less substituted products. These limitations could be overcome when we applied the approach to propargylic amines.[^34^, ^35^] Trisubstituted enol ethers could be obtained with high stereoselectivity, and the installed trifluoromethyl group can be used to control the relative configuration of stereocenters in further synthetic steps, resulting in more functionalized products. Nevertheless, this method was limited exclusively to the formation of 1,2‐amino alcohols. Furthermore, subsequent transformations were limited by the low reactivity of the initially formed trisubstituted enol ethers.^[36]^

Herein, we report the first application of the tethering approach to the stereoselective formation of enamines from propargylic alcohols, and therefore to highly functionalized regioisomeric 2,1‐amino alcohols (Scheme 1C). During our investigations, we discovered that different ring size products could be selectively formed by choice of the molecular tether, thus achieving the regioselective formation of 5‐*exo* and 6‐*endo* cyclic enamines. The later gave access to substituted 3,1‐amino alcohols, an equally important scaffold present in pharmaceuticals and natural products, which could not be access by our tethering approach before. The enhanced reactivity of the enamines allowed us to develop a new fluorination reaction, which could not be performed on the previously reported enol ethers.

A preliminary examination of the carboamination process involved the use of methyl substituted propargylic alcohol **1** **a**, bromoalkyne **2** **a** and various aldimine or hemiaminal molecular tethers **3** **a**–**f**, alongside with Pd_2_(dba)_3_ ⋅ CHCl_3_ as the palladium source and a monophosphine DACH‐phenyl Trost ligand **L1** (Table 1). The decision to employ this particular ligand was based on its sucessful application in our previous research for the difunctionalization of propargylic amines.^[35]^ The aminoalkynylation with tethers bearing carbamate protecting groups such as Boc (**3** **a**), Cbz (**3** **b**) or methyl carbamate (**3** **c**) did not yield any product (entry 1), in contrast to previous carboamination strategies applied to alkenes.[^29^, ^37^] With the use of *para*‐anisidine trifluoroacetaldimine methanol hemiaminal **3** **d**, we were surprised to observe the formation of the 6‐*endo‐dig* cylization product **5** **aa** in 80 % ^1^H NMR yield (entry 2). Substituting **3** **d** with trifluoroaldimine **3** **e** resulted in a yield of 82 % of **5** **aa** (entry 3). Other monophosphine or diphosphine ligands, including RuPhos (**L2**), P(Furyl)_3_ (**L3**) and DPEPhos (**L4**), did not lead to any product formation (entry 4) (see Supporting Information, section C for more details). When employing a tosyl protecting group (**3** **f**), the 5‐*exo‐dig* cyclization product **4** **aa** was produced selectively in 36 % yield (entry 5). The yield could be improved by substituting Cs_2_CO_3_ with K_3_PO_4_ as the base (entry 6). Therefore, the present synthetic methodology can be viewed as a protecting group‐controlled regioselective carboamination of alkynes (a speculative mechanistic proposal is provided in the SI, section F). No product formation was observed in the absence of a Pd catalyst, ligand **L1** or a base, and the yield was impacted when the reaction was performed under air (entries 7 and 8). Lastly, the reaction could be scaled up to 0.4 mmol to selectively obtain **5** and **4** in 75 % and 78 % isolated yield, respectively (entries 9 and 10).

**Table 1:** Optimization of the regioselective formation of enamines **4** and **5**.

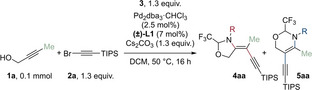			
Entry	Conditions	**4** **aa** (%)^[a]^	**5** **aa** (%)^[a]^
1	With **3** **a**, **3** **b** or **3** **c**	n. d.	n. d.
2	With **3** **d**	n. d.	80
3	With **3** **e**	n. d.	82
4	With **L2**, **L3** or **L4**	n. d.	n. d.
5	With **3** **f** instead of **3** **e**	36	n. d.
6	With **3** **f** and K_3_PO_4_	77	n. d.
7	Without Pd, ligand or base	n. d.	n. d.
8	With **3** **e**, under air	n. d.	34
9	**3** **e**, 0.4 mmol scale^[b]^	n. d.	83 (75)^[c]^
10	**3** **f**, K_3_PO_4_, 0.4 mmol scale^[b]^	81 (78)^[c]^	n. d.
			
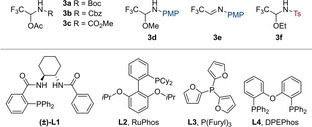			

[a] ^1^H NMR yields were determined using trichloroethylene (1.0 equiv.) as standard. [b] With 10 mol % of ligand. [c] Isolated yield. n. d.=not detected.

Subsequently, we examined the scope of the 6‐*endo*‐selective cyclization (Scheme 2). The model substrate **5** **aa** was isolated in 75 % yield. Several aryl propargylic alcohols, either commercially available (**1** **b**) or prepared in one step from the terminal alkyne,^[38]^ provided the desired *endo*‐cyclization products in good yields. Beside unsubstituted phenyl (**5** **ba**), methoxy, fluoro and chloro groups on the *para*‐position of the phenyl ring, were compatible with the reaction (**5** **ca**, **5** **da**, and **5** **ea**). With the electron‐withdrawing CF_3_ group, **5** **fa** was obtained in 63 % yield, but with a lower regioselectivity. A *meta*‐fluorine substituent was tolerated, to give **5** **ga** in 29 % yield. Furthermore, a thiophene heterocycle could be integrated into the *endo* cyclic product in 81 % yield (**5** **ha**). Other aliphatic substituted propargylic alcohols were also successful, including cyclopropyl **5** **ia** and TBS‐protected alcohol **5** **ja** that were obtained in 64 and 62 % yields, respectively, with perfect 6‐*endo* selectivity. A vinyl group substituted *endo* product could also be accessed in 71 % yield and 3 : 1 regioselectivity (**5** **ka**). Propargylic alcohols with substitution in the α position also participated in the cyclization, one methyl group afforded **5** **la** in 37 % yield and 2 : 1 *dr*, and dimethyl substitution yielded **5** **ma** in 15 %. In addition to silylated alkynes, the aminoalkynylation also worked with aryl substituted alkynyl bromides including phenyl and *para*‐OMe‐, *para*‐CF_3_‐, and *para*‐COMe‐phenyl substituents (**5** **ab**–**d**). α‐CF_3−_vinyl bromide as electrophile gave product **5** **ae** in a moderate 22 % yield.

**Scheme 2:**
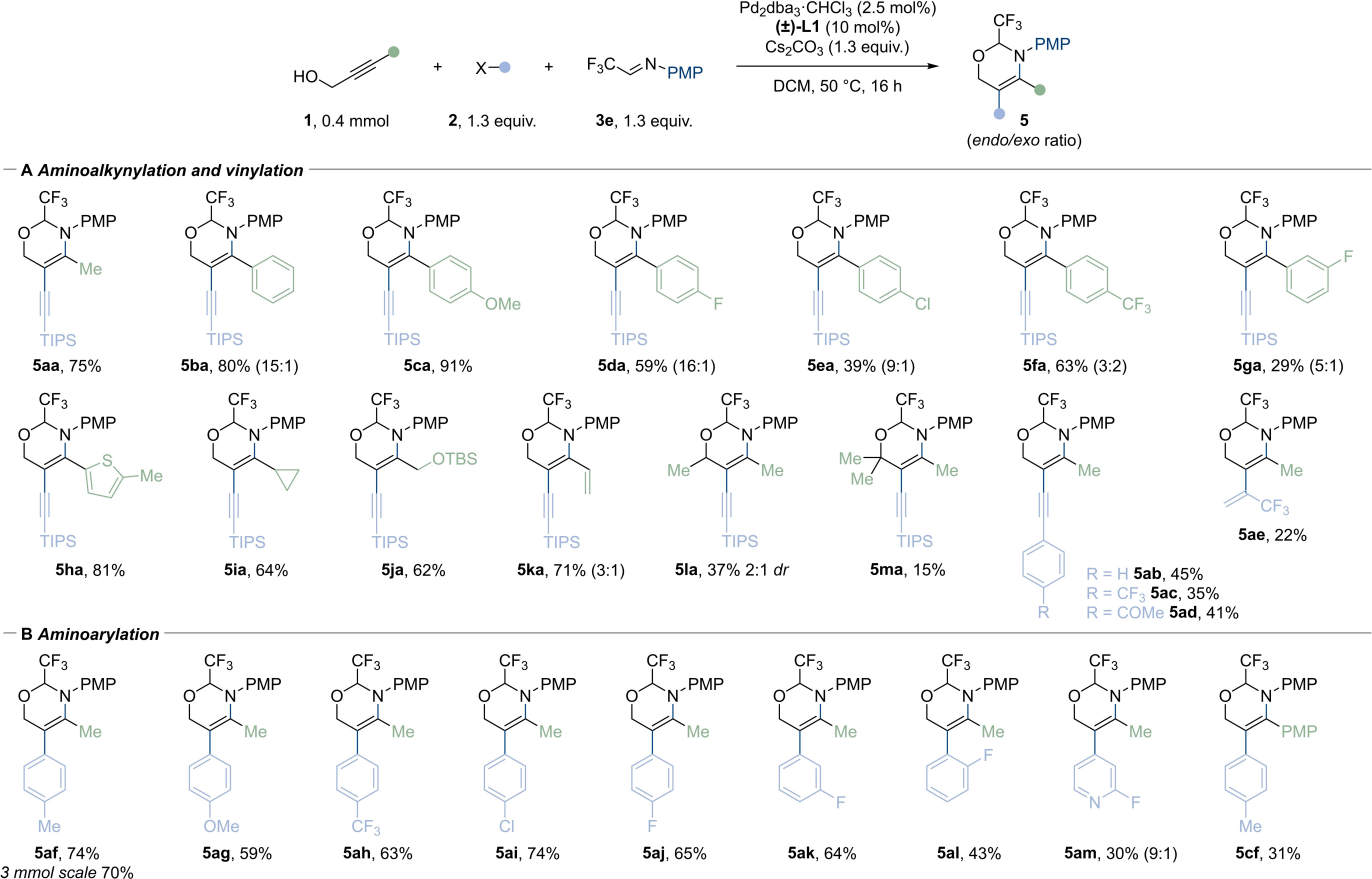
Scope of the regioselective 6‐*endo* tethered A) aminoalkynylation and vinylation (X=Br) and B) aminoarylation (X=I) of propargylic alcohols. Isolated yields are given. *Endo*/*exo* ratios were determined by ^1^H NMR.

We then turned to the related aminoarylation process focusing first on propargylic alcohol **1** **a** as partner. The product **5** **af** was isolated in 74 % yield at a 0.4 mmol scale, and the reaction could also be performed on a 3 mmol scale to give **5** **af** in 70 % yield (760 mg). Multiple substituents could be introduced in *para*‐position of the aromatic ring in yields ranging from 59 to 74 % (**5** **ag**–**j**). Substitution at the *meta*‐ and *ortho*‐positions was also tolerated, affording **5** **ak** and **5** **al** in 64 % and 43 % yield respectively. A *meta*‐fluoro substituted pyridine could be introduced in 30 % yield (**5** **am**). Finally, doubly arylated product **5** **cf** could be obtained in 31 % yield.

We then examined the scope of the 5‐*exo*‐selective cyclization by using sulfonamide‐based tethers **3** **f** and **3** **g** (Scheme 3). Both tosyl and nosyl groups were effective in giving **4** **aaf** and **4** **aag** in 78 % and 53 % yield, respectively. Aryl groups were well tolerated on the propargyl alcohol, including a phenyl group, and electron donating and electron withdrawing groups substituted arenes, which gave products **4** **baf**–**4** **caf** in 85–99 % yield. An aromatic *ortho*‐OMe substitution was tolerated in this case giving 51 % yield (**4** **naf**). Additionally, an aliphatic propargylic alcohol afforded **4** **jaf** in 85 % yield. An α‐substituted propargylic alcohol gave enamine **4** **laf** in 67 % yield and 1 : 1 *dr*. We then examined several aryl iodides as electrophiles. Enamide **4** **aff** was obtained in 63 % yield at a 3 mmol scale. Electron withdrawing groups afforded the products **4** **ahf** and **4** **bhf** in 75 % and 98 % yield, as well as Ns protected enamines **4** **bhg** and **4** **ahg** in 68 % and 62 % yield. Other substituents were also tolerated including a *para*‐ester (**4** **anf**), a *meta*‐CF_3_ (**4** **aof**) and an *ortho*‐fluoro group (**4** **apf**), with yields ranging from 71 to 73 %.

**Scheme 3:**
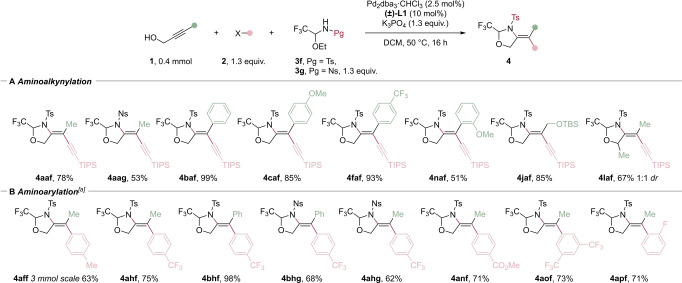
Scope of the regioselective 5‐*exo* tethered A) aminoalkynylation (X=Br) and B) aminoarylation (X=I) of propargylic alcohols. Isolated yields are given. [a] The reaction was carried out in EtOAc at 80 °C.

In our previous work towards the carboetherification of propargylic amines,[^34^, ^35^] we had uncovered that the trifluoromethyl group effectively hinders one of the two sides of the exocyclic olefin of an oxazolidine ring, enabling stereoselective transformations, in particular hydrogenation. We were pleased to observe that the hydrogenation of the 6‐membered ring **5** **af** and the trifluoromethyl derivative **5** **ah** was also completely diastereoselective, giving **6** **af** and **6** **ah** in 74 % and 54 % yield and >20 : 1 *dr* (Scheme 4A). By X‐ray crystallographic analysis we observed that the trifluoromethyl substituent of **5** **af** occupies an axial position, shielding efficiently one face of the alkene, and we therefore assumed an *anti* hydrogenation leading to all *syn*‐product **6** **af**. Following the diastereoselective hydrogenation of **5** **af** and **5** **ah**, the tether could be removed using acidic conditions, yielding **7** **af** and **7** **ah** in 62 % and 66 % yield respectively. From **7** **ah**, the intramolecular substitution of a tosylate generated in situ afforded azetidine **8** in 52 % yield.^[39]^ Furthermore, the PMP group can be removed to afford the deprotected stereodefined 1,3‐amino alcohol **9**, following a reported procedure.^[40]^ Enamine **5** **af** was also subjected to an electrophilic fluorination reaction with NFSI to afford α‐fluoro‐β‐hydroxy ketone **10** in 81 % yield. This reaction is proposed to occur via iminium formation and tether cleavage. To support this hypothesis and conserve the precious nitrogen functionality in the product, we attempted intercepting the iminium intermediate with a hydride source. Gratifyingly, in presence of NaBH_3_CN, fluorinated amine **11** was obtained in 98 % yield and 17 : 1 diastereoselecivity. The tether was then cleaved to give fluorinated amino alcohol **12** in 72 % yield and 22 : 1 *dr*. It is interesting to note that the fluorination reaction is specific to the enamines obtained in this work, as the transformation did not proceed with the enol ethers obtained previously.

**Scheme 4:**
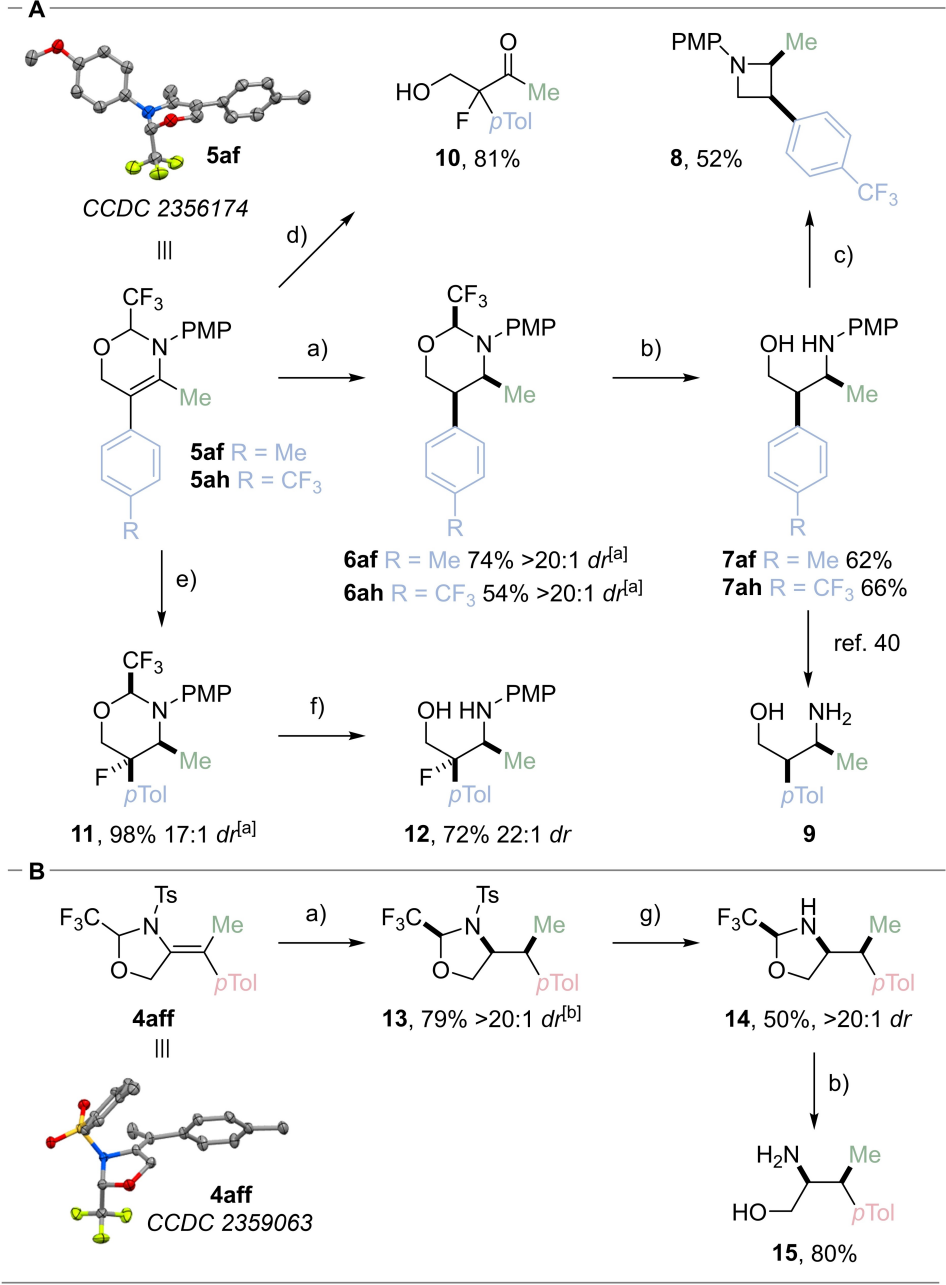
Product modifications of (A) 6‐*endo* enamines **5** and (B) 5‐*exo* enamines **4**. Conditions: a) H_2_ (1 bar), Pd(OH)_2_/C (20 mol %), MeOH/AcOH (2 : 1), rt, 16 h; b) TsOH (7.0 equiv.), THF/H_2_O (9 : 1), rt, 16 h; c) TsCl (2.5 equiv.), DMAP (0.2 equiv.), CHCl_3_, Et_3_N, 70 °C, MW, 1 h; d) NFSI (1.2 equiv.), MeOH, rt, 16 h; e) NFSI (1.2 equiv.), NaBH_3_CN (1.2 equiv.), MeOH, rt, 16 h; f) TsOH (7.0 equiv.), THF/H_2_O (9 : 1), 70 °C, 16 h; g) Mg (200 equiv.), MeOH, rt, 2 h. [a] The relative stereochemistry was determined by ^1^H NMR. [b] The relative stereochemistry was assumed based on the shielding effect of the trifluoromethyl group.

In the case of cyclic enamides obtained in the 5‐exo cyclization process, the Pd‐catalyzed hydrogenation of **4** **aff** afforded **13** in excellent diastereoselectivity and 79 % yield (Scheme 4B). The structure of **4** **aff**, elucidated by X‐ray single‐crystal analysis, supported again the shielding effect of the CF_3_ group. It was also possible to remove the tosyl protecting group from **13** using magnesium, and **14** was obtained in 50 % yield. The tether cleavage was then performed using toluenesulfonic acid to give deprotected 2,1‐amino alcohol **15** in 80 % yield.

In conclusion, we have developed a palladium‐catalyzed 6‐*endo*/5‐*exo* tethered regioselective carboamination of propargylic alcohols to synthesize highly substituted enamines. The choice of the protecting group on the molecular tether enabled us to control the regioselectivity of the transformation. The two cyclization processes gave access to valuable five‐ and six‐membered cyclic enamines from easy to access propargylic alcohols in one step. Due to the shielding effect of the introduced trifluoromethyl group, stereoselective transformations were possible, leading to the formation of heteroatom‐rich heterocycles, 3,1‐ and 2,1‐ amino alcohols and fluorinated compounds, all building blocks of high interest in synthetic and medicinal chemistry.

## Supporting Information

General methods, synthetic procedures, compounds characterization data and copy of NMR spectra of new compounds are provided in the Supporting Information (pdf). Raw data for NMR, MS and IR are freely available on the platform zenodo: https://doi.org/10.5281/zenodo.13134128. The authors have cited additional references within the Supporting Information.[^35^, ^38^, ^39^, ^41^, ^42^, ^43^, ^44^, ^45^, ^46^, ^47^, ^48^, ^49^, ^50^, ^51^, ^52^, ^53^, ^54^, ^55^, ^56^, ^57^, ^58^, ^59^, ^60^, ^61^, ^62^, ^63^, ^64^, ^65^, ^66^, ^67^]

## Conflict of Interests

The authors declare no conflict of interest.

## Supporting information

As a service to our authors and readers, this journal provides supporting information supplied by the authors. Such materials are peer reviewed and may be re‐organized for online delivery, but are not copy‐edited or typeset. Technical support issues arising from supporting information (other than missing files) should be addressed to the authors.

Supporting Information

## Data Availability

The data that support the findings of this study are available in the supplementary material of this article.
