# Supplementary material for: Enamine Synthesis via Regiocontrolled 6‐endo‐dig and 5‐exo‐dig Tethered Carboamination of Propargylic Alcohols
Source: Angew Chem Int Ed Engl. 2024 Oct 24;63(50):e202411383. doi: 10.1002/anie.202411383 (PMC11609970; doi:10.1002/anie.202411383)

Angewandte Chemie
Eine Zeitschrift der Gesellschaft Deutscher Chemiker

Supporting Information

Enamine Synthesis via Regiocontrolled 6-*endo*-*dig* and 5-*exo*-*dig* Tethered Carboamination of Propargylic Alcohols

*H. Solé-Àvila, M. Puriņš, L. Eichenberger, J. Waser**

Supporting Information for

"Enamine Synthesis *via* Regiocontrolled 6-*endo-dig* and 5-*exo-dig* Tethered Carboamination of Propargylic Alcohols"

Helena Solé-Àvila, Mikus Puriņš, Lucas Eichenberger and Jerome Waser*

Laboratory of Catalysis and Organic Synthesis, Ecole Polytechnique Fédérale de Lausanne, EPFL, SB ISIC LCSO, BCH 4306, 1015 Lausanne (Switzerland)

*Correspondence to: jerome.waser@epfl.ch

Table of Contents

A. General Information 3
B. Synthesis of the Starting Materials and Ligands 4
B.1 Synthesis of CF_3 tethers 4
B.2. Synthesis of propargyl alcohols 6
B.3. Synthesis of alkynyl bromides 11
B.4. Synthesis of L1 12
C. Optimization Studies 13
C.1. Ligand screening 14
C.2. Optimization of reaction conditions 14
D. Procedures and product characterization data of carboamination products (enamines) 16
D.1. General Procedure D1 for the 6-*endo-dig* carboamination reaction 16
D.2. Characterization of the 6-*endo-dig* carboamination products 16
D.3. General Procedure D3 for the 5-*exo-dig* carboamination reaction 25
D.4. Characterization of the 5-*exo-dig* carboamination products 25
D.5. Unsuccessful substrates 30
E. Product modifications 30
E.1. Hydrogenation 30
E.2. Tether opening 33
E.3. Electrophilic fluorination 35
F. Proposed reaction mechanism 37
G. X-ray crystallographic data 38
H. References 60
I. NMR Spectra 61
I.1. 6-*endo-dig* carboamination products 61
I.2. 5-*exo-dig* carboamination products 99
I.3. Product modifications 124

A. General Information

The NMR spectra were recorded on a Brucker DPX-400 spectrometer at 400 MHz for ^{1}H, 101 MHz for ^{13}C, 376 MHz for ^{19}F. The chemical shift (δ) for ^{1}H and ^{13}C are given in ppm relative to residual signals of the solvents ($CDCl_3$ - 7.26 ppm ^{1}H NMR and 77.16 ppm ^{13}C NMR). Carbon spectra have been measured using broadband {^{1}H} decoupling. Coupling constants are given in Hertz. The following abbreviations are used to indicate the multiplicity: s, singlet; d, doublet; q, quartet; m, multiplet; bs, broad signal; app, apparent. Infrared spectra were recorded on a JASCO FT-IR B4100 spectrophotometer with an ATR PRO410-S and a ZnSe prisma and are reported as cm^{-1} (w = weak, m = medium, s = strong, br = broad). High resolution mass spectrometric measurements were performed by the mass spectrometry service of ISIC at the EPFL on a MICROMASS (ESI) Q-TOF Ultima API. The raw data obtained from the Q-TOF Waters instrument does not take into account the mass of the electron for the ion, the obtained raw data has been therefore corrected by removing the mass of the electron (5 mDa). The diffraction data for crystal structures were collected by mass spectrometry service of ISIC at the EPFL at low temperature using Cu (323) or Mo (520) K_a radiation on a Rigaku SuperNova dual system in combination with Atlas type CCD detector. The data reduction and correction were carried out by *CrysAlis^{Pro}* (Rigaku Oxford Diffraction, release 1.171.40.68a, **2019**). The solutions and refinements were performed by *SHELXT*[1] and *SHELXL*[2], respectively. The crystal structures were refined using full-matrix least-squares based on F^2 with all non-H atoms defined in anisotropic manner. Hydrogen atoms were placed in calculated positions by means of the "riding" model. Yields of isolated products refer to materials of >95% purity as determined by ^{1}H NMR.

The authors are indebted to the team of the research support service of ISIC at EPFL, particularly to the NMR, X-Ray, and the High Resolution Mass Spectrometry Units.

General Procedures. All reactions were set up under a nitrogen atmosphere in oven-dried glassware using standard Schlenk techniques, unless otherwise stated. Synthesis grade solvents were used as purchased; anhydrous solvents (THF, Et_2O, Toluene and DCM) were taken from a commercial SPS solvent dispenser (H_2O content < 10 ppm, *Karl-Fischer* titration). Chromatographic purification of products was accomplished using flash chromatography (FC) on SiliaFlash P60 silica gel (230 - 400 mesh) or using Biotage Isolera Spektra One with pre-packaged silica cartridges purchased from Buchi, models: Sepacore or GraceResolve (4 g, 12 g, 25 g, 40 g, 80 g, 120 g). For thin layer chromatography (TLC) analysis throughout this work, Merck silica gel 60 F254 TLC glass plates were employed, using UV light as the visualizing agent and basic aqueous potassium permanganate ($KMnO_4$) stain solutions, and heat as developing agents. Organic solutions were concentrated under reduced pressure on a Büchi rotatory evaporator.

Materials. Most of the starting materials used in this study are commercial and were purchased in the highest purity available from Sigma-Aldrich, Fluka, Alfa Aesar, Fluorochem, Enamine and used as received, without further purifications. Tris(dibenzylideneacetone)dipalladium was purchased from Fluorochem and recrystalised in 200 mg portions following a reported procedure.[1]

B. Synthesis of the Starting Materials and Ligands

B.1 Synthesis of CF_3 tethers

1-((*tert*-Butoxycarbonyl)amino)-2,2,2-trifluoroethyl acetate (3a)

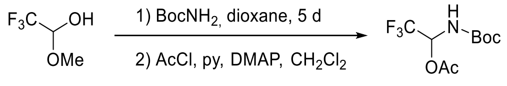

S16 **3a**

Following a reported procedure,[2] a 100 mL pressure tube was charged with *tert*-butyl carbamate (7.03 g, 60.0 mmol), 2,2,2-trifluoro-1-methoxyethanol **S16** (7.70 mL, 66.0 mmol, 1.1 equiv.), 4Å MS (10 g) and dioxane (80 mL). The tube was sealed under nitrogen atmosphere. The resulting mixture was heated at 100 °C for 5 days and then cooled down to rt. The mixture was filtered over Celite and the cake was washed with ether (3 × 20 mL). The volatiles were removed under reduced pressure and the resulting solid was recrystallized in chloroform to afford colorless crystals (8.20 g, 38.1 mmol, 64% for 2 crops).

To a solution of pyridine (0.34 mL, 4.2 mmol, 1.4 equiv.) and DMAP (91 mg, 0.75 mmol, 25 mol%) in DCM (10 mL) at 0 °C was slowly added acetyl chloride (0.25 mL, 3.6 mmol, 1.2 equiv.). To the resulting mixture was added *tert*-butyl (2,2,2-trifluoro-1-hydroxyethyl)carbamate (645 mg, 3.00 mmol) portion-wise. Then the mixture was stirred at 0 °C for 20 min and quenched with water (10 mL). The pH was adjusted to 2 by addition of 0.1 M HCl and the layers were separated. The organic layer was washed with 0.1 M HCl (3 × 20 mL) and brine (30 mL), dried over $MgSO_4$, filtered and concentrated under reduced pressure. The crude residue was purified by column chromatography on silica gel (10:1 (v/v) pentane/EtOAc) affording **3a** (520 mg, 2.02 mmol, 67% yield) as a white solid. Spectral data was consistent with the values reported in the literature.[2]

^{1}H NMR (400 MHz, $CDCl_3$) δ 6.66 (bs, 1H, N*H*), 5.25 (bs, 1H, C*H*), 2.08 (s, 3H, C*H*$_3$), 1.41 (s, 9H, C(C*H*$_3$)$_3$).

^{13}C NMR (101 MHz, $CDCl_3$) δ 168.0, 152.9, 123.1 (q, $J_{C\text{-}F}$ = 281.3), 82.2, 72.0 (q, $J_{C\text{-}F}$ = 39.1 Hz), 28.1, 20.5.

1-(((Benzyloxy)carbonyl)amino)-2,2,2-trifluoroethyl acetate (3b)

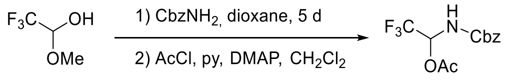

S16 **3b**

Following a reported procedure,[2] a 100 mL pressure tube was charged with benzyl carbamate (2.27 g, 15.0 mmol), 2,2,2-trifluoro-1-methoxyethanol **S16** (1.59 mL, 16.5 mmol, 1.10 equiv.), 4Å MS (3 g) and dioxane (23 mL). The tube was sealed under nitrogen atmosphere. The resulting mixture was heated at 100 °C for 5 d and then cooled down to rt. The mixture was filtered over Celite, and the cake was washed with ether (3 × 5 mL). The volatiles were removed under reduced pressure and the resulting solid was recrystallized in chloroform to afford colorless crystals (1.87 g, 7.50 mmol, 50% for 2 crops).

To a solution of pyridine (1.14 mL, 14.0 mmol, 1.4 equiv.) and DMAP (30 mg, 0.25 mmol, 2.5 mol%) in dichloromethane (50 mL) at 0 °C was slowly added acetyl chloride (0.93 mL, 12 mmol, 1.2 equiv.). To the resulting mixture was added benzyl (2,2,2-trifluoro-1-hydroxyethyl)carbamate (2.50 g, 10.0 mmol, 1 equiv.) portion-wise. Then the mixture was stirred at 0 °C for 20 min and quenched with water (10 mL). The pH was adjusted to 2 by addition of 0.1 M HCl and the layers were separated. The organic layer was washed with 0.1 M HCl (3 × 20 mL) and brine (30 mL), dried over $MgSO_4$, filtered, and concentrated under reduced pressure. The crude residue was purified by column chromatography on silica gel (10:1 (v/v) pentane/EtOAc) affording **3b** (2.40 g, 8.32 mmol, 83% yield) as a white solid. Spectral data was consistent with the values reported in the literature.[2]

^{1}H NMR (400 MHz, $CDCl_3$) δ 7.44 – 7.29 (m, 5H, Ar*H*), 6.80 (dd, *J* = 11.0, 5.6 Hz, 1H, N*H*), 5.58 (s, 1H, C*H*), 5.23 – 5.09 (m, 2H, C*H*$_2$), 2.14 (s, 3H, C*H*$_3$).

^{13}C NMR (101 MHz, $CDCl_3$) δ 168.1, 154.2, 135.3, 128.8 (2), 128.6, 121.7 (q, $J_{C\text{-}F}$ = 281.0 Hz), 72.3 (q, $J_{C\text{-}F}$ = 36.9 Hz), 68.3, 20.6.

^{19}F NMR (376 MHz, $CDCl_3$) δ -80.2.

2,2,2-Trifluoro-1-((methoxycarbonyl)amino)ethyl acetate (3c)

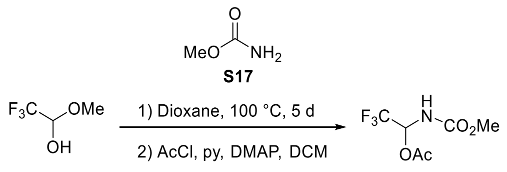

S16 **3c**

Following a slightly modified procedure,[2] a 20 mL microwave vial was charged with methyl carbamate **S17** (2.25 g, 30.0 mmol), 2,2,2-trifluoro-1-methoxyethanol **S16** (3.84 mL, 33.0 mmol, 1.1 equiv.), 4Å MS (4 g) and was filled with dioxane (16 mL). The tube was sealed under nitrogen atmosphere. The resulting mixture was heated at 100 °C for 5

d and then cooled down to rt. The mixture was filtered over Celite and the cake was washed with ethyl acetate (3 × 20 mL). The volatiles were removed under reduced pressure and the residue was purified by column chromatography on silica gel (4:1 (v/v) pentane/EtOAc).

To a solution of pyridine (1.96 mL, 24.3 mmol, 1.4 equiv.) and DMAP (53 mg, 0.43 mmol, 2.5 mol%) in dichloromethane (87 mL) at 0 °C was slowly added acetyl chloride (1.48 mL, 20.8 mmol, 1.2 equiv.). To the resulting mixture was added the previously purified compound (3.00 g, 17.3 mmol) portionwise. Then the mixture was stirred at 0 °C for 20 min and quenched with water (10 mL). The pH was adjusted to 2 by addition of 0.1 M HCl and the layers were separated. The organic layer was washed with 0.1 M HCl (3 × 20 mL) and brine (30 mL), dried over $MgSO_4$, filtered and concentrated under reduced pressure. The crude residue was purified by column chromatography on silica gel (5:1 (v/v) pentane/EtOAc) affording **3c** (3.55 g, 16.5 mmol, 95% yield) as a white solid. Spectral data was consistent with the values reported in the literature.[2]

^{1}H NMR (400 MHz, $CDCl_3$) δ 6.90 – 6.66 (m, 1H, $CHCF_3$), 5.77 – 5.40 (m, 1H, NH), 3.76 (s, 3H, OMe), 2.14 (s, 2H, Ac).

^{13}C NMR (101 MHz, $CDCl_3$) δ 168.1, 154.9, 121.7 (q, $J_{C\text{-}F}$ = 281.0 Hz), 72.3 (q, $J_{C\text{-}F}$ = 36.9 Hz), 53.4, 20.6.

^{19}F NMR (376 MHz, $CDCl_3$) δ -80.3.

4-Methoxy-*N*-(2,2,2-trifluoro-1-methoxyethyl)aniline (3d)

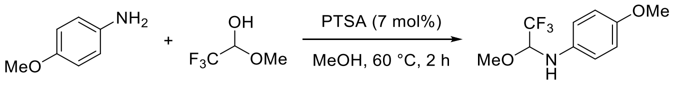

S18 S16 3d

Following a reported procedure,[3] a round bottom flask equipped with a magnetic stirring bar and a condenser was charged with MeOH (40 mL), 4-methoxyaniline **S19** (2.0 g, 16 mmol, 1.0 equiv.), trifluoroacetaldehyde methyl hemiacetal **S18** (6.0 mL, 62 mmol, 3.9 equiv.) and *p*-toluene sulfonic acid (PTSA) monohydrate (223 mg, 1.17 mmol, 0.07 equiv.) and then heated under reflux for 2 h under N_2. After cooling to room temperature, 10% aq. $NaHCO_3$ (30 mL) was added. The mixture was extracted with EtOAc (2 × 50 mL), and dried over Na_2SO_4. The solvent was removed in vacuo and the residue was purified by column chromatography on silica gel (0 – 15% (v/v) EtOAc/pentane) to yield **3d** (1.7 g, 7.4 mmol, 46% yield) as a yellow oil. Spectral data was consistent with the values reported in the literature.[3]

^{1}H NMR (400 MHz, $CDCl_3$) δ 6.82 (d, J = 9.0 Hz, 2H, Ar*H*), 6.75 (d, J = 9.0 Hz, 2H, Ar*H*), 4.88 (dq, J = 10.6, 4.7 Hz, 1H, C*H*), 4.02 (d, J = 10.6 Hz, 1H, N*H*), 3.77 (s, 3H, OCH_3), 3.47 (s, 3H, $CHOCH_3$).

^{13}C NMR (101 MHz, $CDCl_3$) δ 154.2, 138.1, 123.0 (q, J = 283.8 Hz), 116.7, 115.0, 85.0 (q, J = 32.9 Hz), 56.3, 55.7.

^{19}F NMR (376 MHz, $CDCl_3$) δ -80.0 (d, J = 4.6 Hz).

2,2,2-Trifluoro-*N*-(4-methoxyphenyl)ethan-1-imine (3e)

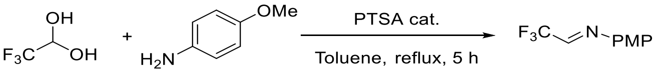

S19 S18 3e

Following a reported procedure,[3] 2,2,2-trifluoroethane-1,1-diol **S19** (3.00 g, 19.4 mmol, 1 equiv.) and toluene (40 mL) were charged in a 100 mL round-bottomed flask equipped with a reflux condenser, a Dean-Stark trap and a magnetic stirring bar. 4-Methoxyaniline **S18** (2.63 g, 21.3 mmol, 1.1 equiv.) and PTSA (12 mg, 0.068 mmol, 0.35 mol%) were added to the reaction flask, and the mixture was stirred at reflux. After the reaction was complete (ca. 5 h, monitoring by ^{1}H and ^{19}F NMR), the solvent was removed in vacuo and aldimine **3e** was purified by distillation. 2,2,2-Trifluoro-*N*-(4-methoxyphenyl)ethan-1-imine **3e** (3.41 g, 16.8 mmol, 87% yield) was obtained as a colorless oil and was stored neat under Ar atmosphere at -20 °C. Spectral data was consistent with the values reported in the literature.[3]

^{1}H NMR (400 MHz, $CDCl_3$) δ 7.83 (q, J = 3.6 Hz, 1H, C*H*), 7.31 – 7.27 (m, 2H, Ar*H*), 6.96 – 6.96 (m, 2H, Ar*H*), 3.84 (s, 3H, CH_3).

^{13}C NMR (101 MHz, $CDCl_3$) δ 160.6, 144.3 (q, J = 38.6 Hz), 140.1, 123.3, 119.7 (q, J = 273.8 Hz), 114.7, 55.7.

^{19}F NMR (376 MHz, $CDCl_3$) δ -70.6 (d, J = 3.6 Hz).

***N*-(1-Ethoxy-2,2,2-trifluoroethyl)-4-methylbenzenesulfonamide (3f)**

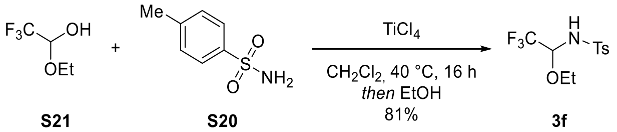

S21 + S20 → 3f (TiCl4; CH2Cl2, 40 °C, 16 h; *then* EtOH; 81%)

Following a reported procedure,[4] a round bottom flask equipped with a magnetic stirring bar under a nitrogen atmosphere was charged with DCM (20 mL), 4-methylbenzenesulfonamide **S20** (1.71 g, 10.0 mmol, 1 equiv.) trifluoroacetaldehyde ethyl hemiacetal **S21** (1.53 mL, 11.0 mmol, 1.1 equiv.) and $TiCl_4$ (2.2 mL, 20 mmol, 2.0 equiv.) were added dropwise. The resulting mixture was stirred for at 40 °C for 16 h, and then treated with ethanol (3.5 mL, 60 mmol, 6.0 equiv.). The crude residue was purified by column chromatography on silica gel (7:2:1 (v/v) pentane/DCM/EtOAc) to afford **3f** (2.41 g, 8.11 mmol, 81% yield) as a white-yellowish solid. Spectral data was consistent with the values reported in literature.[4]

<u>^{1}H NMR</u> (400 MHz, $CDCl_3$) δ 7.75 (d, J = 8.3 Hz, 2H, Ar*H*), 7.31 (d, J = 8.0 Hz, 2H, Ar*H*), 5.32 (t, J = 7.9 Hz, 1H, N*H*), 4.98 (dq, J = 9.4, 4.6 Hz, 1H, CF_3-C*H*), 3.78 (dq, J = 9.4, 7.1 Hz, 1H, O-CH_2), 3.63 (dq, J = 9.4, 7.0 Hz, 1H, O-CH_2), 2.43 (s, 3H, Ar-CH_3), 1.17 (t, J = 7.0 Hz, 3H, CH_3).

<u>^{13}C NMR</u> (101 MHz, $CDCl_3$) δ 144.3, 137.8, 129.9, 127.0, 122.0 (q, $J_{C\text{-}F}$ = 282.7 Hz), 81.4 (q, $J_{C\text{-}F}$ = 35.2 Hz), 65.7, 21.7, 14.7.

<u>^{19}F NMR</u> (376 MHz, $CDCl_3$) δ -80.7.

***N*-(1-Ethoxy-2,2,2-trifluoroethyl)-4-nitrobenzenesulfonamide (3g)**

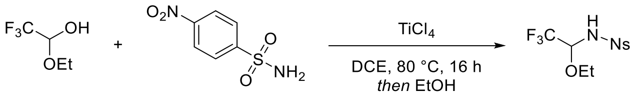

S21 + S22 → 3g (TiCl4; DCE, 80 °C, 16 h; *then* EtOH; 76%)

Following a reported procedure,[4] a round-bottomed flask equipped with a magnetic stirring bar under nitrogen atmosphere was charged with 4-nitrobenzenesulfonamide **S22** (2.02 g, 10.0 mmol) and DCE (20 mL). Then 1-ethoxy-2,2,2-trifluoroethanol **S21** (1.53 mL, 11.0 mmol, 1.1 equiv.) and $TiCl_4$ (2.21 mL, 20.0 mmol, 2.0 equiv.) were added dropwise. The resulting mixture was stirred at 80 °C for 16 h and then treated with ethanol (3.50 mL, 60.0 mmol, 6.0 equiv.). The crude residue was purified by column chromatography on silica gel (3:1 (v/v) pentane/EtOAc) to afford **3g** (2.50 g, 7.61 mmol, 76% yield) as a white solid. Spectral data was consistent with the values reported in literature.[4]

<u>^{1}H NMR</u> (400 MHz, $CDCl_3$) δ 8.40 (d, J = 8.9 Hz, 2H, Ar*H*), 8.09 (d, J = 8.9 Hz, 2H, Ar*H*), 5.61 (dd, J = 39.1, 10.0 Hz, 1H, N*H*), 5.05 (dq, J = 9.1, 4.5 Hz, 1H, CF_3-C*H*), 3.96 – 3.83 (m, 1H, O-CH_2), 3.73 (dq, J = 9.4, 7.0 Hz, 1H, O-CH_2), 1.25 (t, J = 7.1 Hz, 3H, CH_3).

<u>^{13}C NMR</u> (101 MHz, $CDCl_3$) δ 150.5, 146.4, 128.3, 124.6, 121.7 (q, $J_{C\text{-}F}$ = 282.4 Hz), 81.6 (q, $J_{C\text{-}F}$ = 35.5 Hz), 66.0, 14.7.

<u>^{19}F NMR</u> (376 MHz, $CDCl_3$) δ -80.6.

B.2. Synthesis of propargyl alcohols

Commercially available propargylic alcohols:

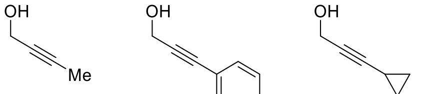

1a 1b 1i

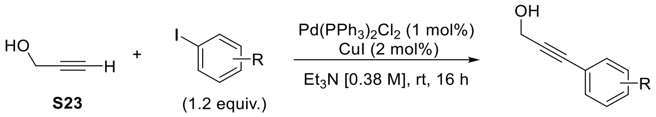

S23 + iodoarene (1.2 equiv.) → product (Pd(PPh3)2Cl2 (1 mol%), CuI (2 mol%); Et3N [0.38 M], rt, 16 h)

General Procedure B2: According to a reported procedure,[5] $Pd(PPh_3)_2Cl_2$ (1 mol%,), CuI (2 mol%) and the iodoarene (1.2 equiv.) were added in a 12 mL microwave tube. The tube was sealed, then put under vacuum and backfilled with nitrogen three times. Triethylamine [0.38 M] was added and the reaction was stirred for 5 min before the addition of propargyl alcohol **S23** (1.0 equiv.). The resulting mixture was stirred for 16 h. Then, the reaction was treated with a saturated $NaHCO_3$ solution. The solution was extracted with ethyl acetate (3 × 20 mL), washed with a saturated NaCl solution (1 × 20 mL) dried over anhydrous $MgSO_4$, filtered and concentrated *in vacuo*. The crude material was purified by flash column chromatography on silica gel to afford the corresponding product.

3-(4-Methoxyphenyl)prop-2-yn-1-ol (1c)

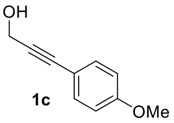

Prepared according to the general procedure B2 using propargyl alcohol (420 mg, 7.50 mmol, 437 µL, 1.00 equiv.) and 4-iodoanisole (2.11 g, 9.00 mmol, 1.20 equiv.). The crude material was purified by column chromatography (30 – 50 % (v/v) EtOAc in pentane) to give **1c** (1.13 g, 6.94 mmol, 93% yield) as a brown solid. Spectral data was consistent with the values reported in literature.[6]

^{1}H NMR (400 MHz, $CDCl_3$) δ 7.43 – 7.33 (m, 2H, Ar*H*), 6.90 – 6.79 (m, 2H, Ar*H*), 4.48 (d, *J* = 6.1 Hz, 2H, HO-C*H*$_2$-C≡C), 3.81 (s, 3H, O-C*H*$_3$), 1.58 (t, *J* = 6.1 Hz, 1H, O*H*).

^{13}C{^{1}H} NMR (101 MHz, $CDCl_3$) δ 159.9, 133.3, 114.7, 114.1, 86.0, 85.9, 55.4, 51.9.

3-(4-Fluorophenyl)prop-2-yn-1-ol (1d)

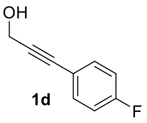

Prepared according to the general procedure B2 using propargyl alcohol (193 mg, 3.44 mmol, 200 µL, 1.00 equiv.) and 1-fluoro-4-iodobenzene (915 mg, 4.12 mmol, 1.20 equiv.). The crude material was purified by column chromatography (0 – 30 % (v/v) EtOAc in pentane) to give **1d** (455 mg, 3.03 mmol, 88% yield) as a yellow solid. Spectral data were consistent with the values reported in literature.[7]

^{1}H NMR (400 MHz, $CDCl_3$) δ 7.46 – 7.38 (m, 2H, Ar*H*), 7.05 – 6.95 (m, 2H, Ar*H*), 4.49 (d, *J* = 6.2 Hz, 2H, C≡C-C*H*$_2$-OH), 1.65 (t, *J* = 6.1 Hz, 1H, O*H*).

^{13}C{^{1}H} NMR (101 MHz, $CDCl_3$) δ 162.8 (d, J_{C-F} = 249.6 Hz), 133.8 (d, J_{C-F} = 8.4 Hz), 118.8 (d, J_{C-F} = 3.6 Hz), 115.8 (d, J_{C-F} = 22.1 Hz), 87.0 (d, J_{C-F} = 1.5 Hz), 84.9, 51.8.

^{19}F NMR (376 MHz, $CDCl_3$) δ -110.6.

3-(4-Chlorophenyl)prop-2-yn-1-ol (1e)

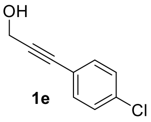

Prepared according to the general procedure B2 using propargyl alcohol (168 mg, 3.00 mmol, 175 µL, 1.00 equiv.) and 1-chloro-4-iodobenzene (858 mg, 3.60 mmol, 1.20 equiv.). The crude material was purified by column chromatography (0 – 30 % (v/v) EtOAc in pentane) to give **1e** (322 mg, 1.93 mmol, 64% yield) as a yellow solid. Spectral data were consistent with the values reported in literature.[6]

^{1}H NMR (400 MHz, $CDCl_3$) δ 7.40 – 7.34 (m, 2H, Ar*H*), 7.32 – 7.27 (m, 2H, Ar*H*), 4.49 (d, *J* = 6.2 Hz, 2H, C≡C-C*H*$_2$-OH), 1.64 (t, *J* = 6.2 Hz, 1H, O*H*).

^{13}C{^{1}H} NMR (101 MHz, $CDCl_3$) δ 134.7, 133.1, 128.8, 121.2, 88.3, 84.8, 51.8.

3-(4-(Trifluoromethyl)phenyl)prop-2-yn-1-ol (1f)

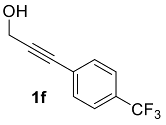

Prepared according to the general procedure B2 using propargyl alcohol (420 mg, 7.50 mmol, 437 µL, 1.00 equiv.) and 1-iodo-4-(trifluoromethyl)benzene (2.45 g, 9.00 mmol, 1.20 equiv.). The crude material was purified by column chromatography (30 – 50 % (v/v) EtOAc in pentane) to give **1f** (1.13 g, 6.94 mmol, 93% yield) as a brown solid. Spectral data were consistent with the values reported in literature.[6]

^{1}H NMR (400 MHz, $CDCl_3$) δ 7.58 (d, *J* = 8.4 Hz, 2H, Ar*H*), 7.54 (d, *J* = 8.3 Hz, 2H, Ar*H*), 4.52 (d, *J* = 6.0 Hz, 2H, HO-C*H*$_2$-C≡C), 1.70 (t, *J* = 6.2 Hz, 1H, O*H*).

^{13}C{^{1}H} NMR (101 MHz, $CDCl_3$) δ 132.1, 130.4 (q, J_{C-F} = 32.7 Hz), 126.5 (q, J_{C-F} = 1.5 Hz), 125.4 (q, J_{C-F} = 3.8 Hz), 124.0 (d, J_{C-F} = 272.2 Hz), 89.8, 84.5, 51.7.

^{19}F NMR (376 MHz, $CDCl_3$) δ -62.9.

3-(3-Fluorophenyl)prop-2-yn-1-ol (1g)

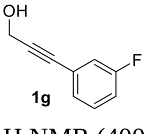

Prepared according to the general procedure B2 using propargyl alcohol (168 mg, 3.00 mmol, 175 µL, 1.00 equiv.) and 1-fluoro-3-iodobenzene (799 mg, 3.60 mmol, 1.20 equiv.). The crude material was purified by column chromatography (0 – 30 % (v/v) EtOAc in pentane) to give **1g** (344 mg, 2.29 mmol, 76% yield) an orange oil. Spectral data were consistent with the values reported in literature.[7]

^{1}H NMR (400 MHz, $CDCl_3$) δ 7.32 – 7.26 (m, 1H, Ar*H*), 7.22 (dt, *J* = 7.7, 1.3 Hz, 1H, Ar*H*), 7.13 (ddd, *J* = 9.4, 2.7, 1.4 Hz, 1H, Ar*H*), 7.04 (tdd, *J* = 8.4, 2.6, 1.2 Hz, 1H, Ar*H*), 4.50 (d, *J* = 6.2 Hz, 2H, C≡C-C*H*$_2$-OH), 1.66 (t, *J* = 6.2 Hz, 1H, O*H*).

^{13}C{^{1}H} NMR (101 MHz, $CDCl_3$) δ 162.5 (d, J_{C-F} = 246.5 Hz), 130.1 (d, J_{C-F} = 8.5 Hz), 127.7 (d, J_{C-F} = 3.0 Hz), 124.5 (d, J_{C-F} = 9.5 Hz), 118.6 (d, J_{C-F} = 22.9 Hz), 116.0 (d, J_{C-F} = 21.1 Hz), 88.3, 84.6 (d, J_{C-F} = 3.4 Hz), 51.7.

^{19}F NMR (376 MHz, $CDCl_3$) δ -112.9.

3-(5-Methylthiophen-2-yl)prop-2-yn-1-ol (1h)

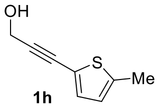

1h

Prepared according to the general procedure B2 using propargyl alcohol (72.9 mg, 1.30 mmol, 75.7 µL, 1.00 equiv.) and 2-iodo-5-methylthiophene (350 mg, 1.56 mmol, 1.20 equiv.). The crude material was purified by column chromatography (0 – 20 % (v/v) EtOAc in pentane) to give **1h** (179 mg, 1.17 mmol, 90% yield) as a black oil. Spectral data were consistent with the values reported in literature.[8]

^{1}H NMR (400 MHz, $CDCl_3$) δ 7.02 (d, *J* = 3.5 Hz, 1H, Ar*H*), 6.62 (dq, *J* = 3.5, 1.1 Hz, 1H, Ar*H*), 4.49 (d, *J* = 6.0 Hz, 2H, C≡C-CH_2-OH), 2.46 (d, *J* = 1.1 Hz, 3H, Ar-CH_3), 1.61 (t, *J* = 6.1 Hz, 1H, O*H*).

^{13}C{^{1}H} NMR (101 MHz, $CDCl_3$) δ 142.5, 132.8, 125.4, 120.0, 90.4, 79.7, 51.9, 15.5.

4-((*tert*-Butyldimethylsilyl)oxy)but-2-yn-1-ol (1j)

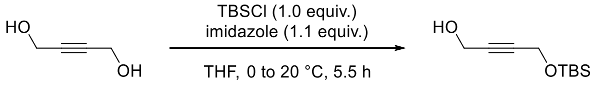

S28 (3.0 equiv.) **1j**

According to a reported procedure,[13] 1,4-but-2-yne-diol **S28** (1.29 g, 15.0 mmol, 3.00 equiv.) was dissolved in 15 mL THF in a 50 mL flame-dried round-bottom flask and the reaction mixture was cooled down to 0 °C. Imidazole (374 mg, 5.50 mmol, 1.00 equiv.) was then added at 0 °C. A solution of TBSCl (753 mg, 5.00 mmol, 1.00 equiv.) in 5 mL THF was added dropwise over 15 min at 0 °C. The reaction mixture was slowly allowed to reach room temperature. After the reaction was completed by TLC (5.5 h), the reaction mixture was diluted with a saturated NH_4Cl solution (20 mL) and extracted with EtOAc (3 × 10 mL). The organic layers were combined and the resulting organic layer was washed with brine (20 mL), dried on $MgSO_4$, filtered and concentrated *in vacuo*. The crude material was purified by flash column chromatography on silica gel (20% (v/v) EtOAc/pentane) to afford the 4-((*tert*-butyldimethylsilyl)oxy)but-2-yn-1-ol **1j** (560 mg, 2.79 mmol, 56% yield) as a yellow oil. Spectral data were consistent with the values reported in literature.[14]

^{1}H NMR (400 MHz, $CDCl_3$) δ 4.36 (t, *J* = 1.8 Hz, 2H, C≡C-CH_2-OTBS), 4.30 (dt, *J* = 6.2, 1.9 Hz, 2H, C≡C-CH_2-OH), 1.50 (t, *J* = 6.2 Hz, 1H, O*H*), 0.91 (s, 9H, Si-C(CH_3)$_3$), 0.12 (s, 6H, Si(CH_3)$_2$).

^{13}C{^{1}H} NMR (101 MHz, $CDCl_3$) δ 84.7, 83.1, 51.9, 51.4, 26.0, 18.5, -5.0.

Pent-4-en-2-yn-1-ol (1k)

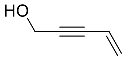

1k

Prepared according to the general procedure B2 using propargyl alcohol (1.15 mL, 20 mmol, 1.00 equiv.), vinylbromide (20 mL of 1 M in THF, 20 mmol, 1.00 equiv.,) and disopropylamine (5.6 mL, 40 mmol, 2.00 equiv.). The crude material was purified by column chromatography (0 – 40 % (v/v) EtOAc in pentane) to give **1k** (1.46 g, 17.8 mmol, 89% yield). Spectral data were consistent with the values reported in literature.[9]

^{1}H NMR (400 MHz, $CDCl_3$) δ 5.82 (ddt, *J* = 17.6, 11.0, 1.9 Hz, 1H, C=C*H*), 5.65 (dd, *J* = 17.6, 1.8 Hz, 1H, C=CH_2), 5.50 (dd, *J* = 11.0, 1.9 Hz, 1H, C=CH_2), 4.39 (d, *J* = 4.6 Hz, 2H, O-CH_2), 1.68 (dt, *J* = 28.0, 5.7 Hz, 1H, O*H*).

Pent-3-yn-2-ol (1l)

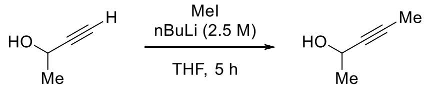

S29 **1l**

Following a reported procedure,[15] but-3-yn-2-ol **S29** (0.50 mL, 6.4 mmol, 1.0 equiv.) and dry THF (11 mL) were stirred at 0 °C. n-Butyllithium (1.6 M hexane, 8.8 mL, 14 mmol, 2.2 equiv.) was added dropwise. After the addition, the mixture was left stirring for 20 min at 0 °C before adding MeI (8.3 mL, 8.3 mmol, 1.3 equiv.) and the solution was removed from the ice bath and allowed to warm to room temperature over the course of 2 h. The solution was cooled back to 0 °C and 1 M NaOH (5 mL) and water (5 mL) were added. The solution was extracted with Et_2O (3 × 10 mL). The organic layers were combined, washed with brine (30 mL), dried with $MgSO_4$, filtered, and concentrated to yield an orange oil. The material was purified by distillation at reduced pressure using a Hickman distillation head to yield pent-3-yn2-ol **1l** as a clear liquid (193 mg, 2.29 mmol, 36% yield). Spectral data were consistent with the values reported in the literature.[16]

^{1}H NMR (400 MHz, $CDCl_3$) δ 4.49 (dq, *J* = 6.5, 2.1 Hz, 1H), 1.83 (d, *J* = 2.1 Hz, 3H), 1.42 (d, *J* = 6.5 Hz, 3H). O*H* was not resolved.

^{13}C{^{1}H} NMR (101 MHz, $CDCl_3$) δ 81.5, 80.3, 58.7, 24.8, 3.6.

2-Methylpent-3-yn-2-ol (1m)

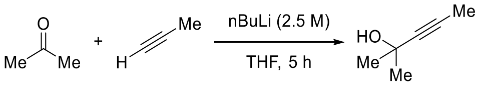

1l

Following a reported procedure,[17] prop-1-yne (1 mol/L solution in THF, 13 mmol, 1.3 equiv.) was dissolved in 25 mL of dry THF in a round-bottom flask. After the mixture was cooled to -78 ºC, a 2.5 M solution of *n*BuLi in hexanes (5.2 mL, 13 mmol, 1.3 equiv.) was added dropwise. The temperature was maintained between -78 ºC and -40 ºC for 1 h with stirring of the solution. Acetone (0.75 mL, 10 mmol, 1.0 equiv.) was then added slowly and the stirring was continued for 3 h allowing the reaction mixture to warm up to room temperature slowly without additional cooling. After completion, the reaction was quenched with a saturated NH_4Cl solution and extracted with DCM. Th evaporation of the solvent led to a crude **1m** (0.99 g, 10 mmol, quant.) which did not need chromatographic purification. Spectral data were consistent with the values reported in the literature.[18]

^{1}H NMR (400 MHz, $CDCl_3$) δ 2.19 (s, 1H), 1.79 (s, 3H), 1.46 (s, 6H).

^{13}C{^{1}H} NMR (101 MHz, $CDCl_3$) δ 84.4, 78.1, 65.3, 31.7, 3.5.

3-(2-Methoxyphenyl)prop-2-yn-1-ol (1n)

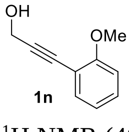

1n

Prepared according to the general procedure B2 using propargyl alcohol (420 mg, 7.50 mmol, 437 µL, 1.00 equiv.) and 2-iodoanisole (2.11 g, 9.00 mmol, 1.20 equiv.). The crude material was purified by column chromatography (0 – 30 % (v/v) EtOAc in pentane) to give **1n** (961 mg, 5.92 mmol, 79 % yield) as a light-yellow solid. Spectral data were consistent with the values reported in literature.[7]

^{1}H NMR (400 MHz, $CDCl_3$) δ 7.42 (dd, *J* = 7.5, 1.8 Hz, 1H, Ar*H*), 7.30 (ddd, *J* = 8.7, 7.7, 1.8 Hz, 1H, Ar*H*), 6.95 – 6.85 (m, 2H, Ar*H*), 4.55 (d, *J* = 6.2 Hz, 2H, HO-CH_2-C≡C), 3.89 (s, 3H, Ar-O-CH_3), 1.70 (q, *J* = 6.0 Hz, 1H, O*H*).

^{13}C{^{1}H} NMR (101 MHz, $CDCl_3$) δ 160.2, 134.0, 130.2, 120.6, 111.8, 110.7, 91.5, 82.1, 55.9, 52.1.

3-(o-Tolyl)prop-2-yn-1-ol (S24)

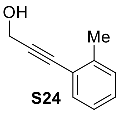

S24

Prepared according to the general procedure B2 using propargyl alcohol (420 mg, 7.50 mmol, 437 µL, 1.00 equiv.) and 2-iodotoluene (1.96 g, 9.00 mmol, 1.20 equiv.). The crude material was purified by column chromatography (0 – 30 % (v/v) EtOAc in pentane) to give **S24** (910 mg, 6.22 mmol, 83 % yield) as an orange oil. Spectral data were consistent with the values reported in literature.[10]

^{1}H NMR (400 MHz, $CDCl_3$) δ 7.41 (dd, *J* = 7.6, 1.4 Hz, 1H, Ar*H*), 7.25 – 7.17 (m, 2H, Ar*H*), 7.17 – 7.08 (m, 1H, Ar*H*), 4.54 (d, *J* = 5.8 Hz, 2H, HO-CH_2-C≡C), 2.43 (s, 3H, Ar-CH_3), 1.67 (t, *J* = 6.1 Hz, 1H, O*H*).

^{13}C{^{1}H} NMR (101 MHz, $CDCl_3$) δ 140.4, 132.2, 129.6, 128.7, 125.7, 122.4, 91.2, 84.8, 51.9, 20.8.

Methyl 4-(3-hydroxyprop-1-yn-1-yl)benzoate (S25)

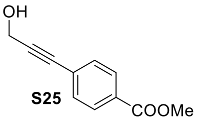

S25

Prepared according to the general procedure B2 using propargyl alcohol (168 mg, 3.00 mmol, 175 µL, 1.00 equiv.) and methyl 4-iodobenzoate (943 mg, 3.60 mmol, 1.20 equiv.). The crude material was purified by column chromatography (0 – 30 % (v/v) EtOAc in pentane) to give **S25** (405 mg, 2.12 mmol, 71 % yield) as a light-yellow solid. Spectral data were consistent with the values reported in literature.[7]

^{1}H NMR (400 MHz, $CDCl_3$) δ 8.03 – 7.94 (m, 2H, Ar*H*), 7.54 – 7.45 (m, 2H, Ar*H*), 4.52 (d, *J* = 6.2 Hz, 2H, C≡C-CH_2-OH), 3.92 (s, 3H, COOCH_3), 1.68 (td, *J* = 6.3, 0.9 Hz, 1H, O*H*).

^{13}C{^{1}H} NMR (101 MHz, $CDCl_3$) δ 166.6, 131.7, 130.0, 129.6, 127.4, 90.3, 85.1, 52.4, 51.8.

3-(2-Fluorophenyl)prop-2-yn-1-ol (S26)

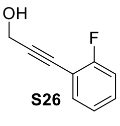

S26

Prepared according to the general procedure B2 using propargyl alcohol (168 mg, 3.00 mmol, 175 µL, 1.00 equiv.) and 1-fluoro-2-iodobenzene (799 mg, 3.60 mmol, 1.20 equiv.). The crude material was purified by column chromatography (0 – 30 % (v/v) EtOAc in pentane) to give **S26** (346 mg, 2.30 mmol, 77 % yield) an orange oil. Spectral data were consistent with the values reported in literature.[11]

^{1}H NMR (400 MHz, $CDCl_3$) δ 7.43 (td, *J* = 7.4, 1.8 Hz, 1H, Ar*H*), 7.35 – 7.27 (m, 1H, Ar*H*), 7.13 – 7.03 (m, 2H, Ar*H*), 4.54 (d, *J* = 6.0 Hz, 2H, C≡C-CH_2-OH), 1.76 (t, *J* = 6.1 Hz, 1H, O*H*).

^{13}C{^{1}H} NMR (101 MHz, $CDCl_3$) δ 163.0 (d, J_{C-F} = 251.7 Hz), 133.8 (d, J_{C-F} = 1.4 Hz), 130.4 (d, J_{C-F} = 8.0 Hz), 124.1 (d, J_{C-F} = 3.7 Hz), 115.7 (d, J_{C-F} = 21.0 Hz), 111.2 (d, J_{C-F} = 15.7 Hz), 92.6, 92.5, 51.8.

^{19}F NMR (376 MHz, $CDCl_3$) δ -110.2.

3-(Pyridin-3-yl)prop-2-yn-1-ol (S27)

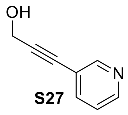

Prepared according to the general procedure B2 using propargyl alcohol (100 mg, 1.78 mmol, 104 µL, 1.00 equiv.) and 3-iodopyridine (439 mg, 2.14 mmol, 1.20 equiv.). The crude material was purified by column chromatography (90 – 100 % (v/v) EtOAc in pentane) to give **S27** (177 mg, 1.32 mmol, 75 % yield) as a colorless solid. Spectral data were consistent with the values reported in literature.[12]

^{1}H NMR (400 MHz, $CDCl_3$) δ 8.73 (dd, *J* = 2.3, 0.9 Hz, 1H, Ar*H*), 8.54 (dd, *J* = 4.9, 1.7 Hz, 1H, Ar*H*), 7.73 (dt, *J* = 7.9, 1.9 Hz, 1H, Ar*H*), 7.42 – 7.03 (m, 1H, Ar*H*), 4.52 (d, *J* = 6.2 Hz, 2H, C≡C-CH_2-OH), 2.56 (br, 1H, O*H*).

^{13}C{^{1}H} NMR (101 MHz, $CDCl_3$) δ 152.5, 148.9, 138.9, 123.3, 120.1, 91.3, 82.3, 51.5.

***tert*-Butyl 4-hydroxybut-2-ynoate (S31)**

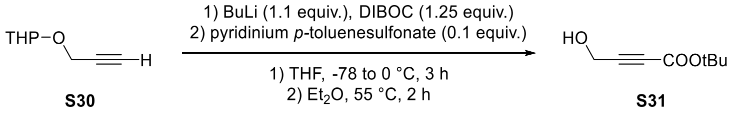

According to a reported procedure,[19] a 50 mL round-bottom flask was flame dried and put under inert atmosphere. 2-(Prop-2-yn-1-yloxy)tetrahydro-2*H*-pyran **S30** (1.40 g, 10.0 mmol, 1.00 equiv.) and dry THF (16 mL) were added and the solution was cooled to −78 °C. n-BuLi (2.5 M solution in hexanes, 4.40 mL, 11.0 mmol, 1.1 equiv.) was slowly added, the mixture was stirred for 1.5 h at −78 °C. A solution of di-*tert*-butyl dicarbonate (2.73 g, 12.5 mmol, 1.25 equiv.) in dry THF (2.5 mL) was slowly added to the mixture over a period of 20 min. The mixture was stirred at −78 °C for 1 h before being removed from the cooling bath and placed in an ice-water bath (0 °C), in which it was stirred for 30 min. The reaction was quenched through the addition of saturated aqueous NH_4Cl (25 mL). The separated aqueous layer was extracted with EtOAc (3 × 20 mL) and then the combined organic layers were washed with brine (25 mL), dried on $MgSO_4$, filtered, and concentrated. This material was subjected to the subsequent reaction without further purification. The crude product was dissolved in absolute EtOH (40 mL) and pyridinium *p*-toluenesulfonate (0.251 g, 1.00 mmol, 1.00 equiv.) was added. The mixture was heated to 55 °C and stirred at that temperature for 2 h. After this time, the reaction was quenched through the addition of saturated aqueous $NaHCO_3$ (100 mL). The mixture was concentrated under reduced pressure to remove the EtOH. The aqueous slurry was extracted with Et_2O (5 × 20 mL). The combined organic layers were washed with brine, dried on $MgSO_4$, and concentrated under reduced pressure. The crude material was purified by flash column chromatography on silica gel (0 – 20% (v/v) EtOAc/pentane) to afford **S31** as a colorless oil (592 mg, 3.79 mmol, 38% yield). Spectral data was consistent with the values reported in literature.[19]

^{1}H NMR (400 MHz, $CDCl_3$) δ 4.38 (d, *J* = 6.4 Hz, 2H, C≡C-CH_2-OH), 1.70 (t, *J* = 6.5 Hz, 1H, O*H*), 1.50 (s, 9H, COOC(CH_3)$_3$).

^{13}C{^{1}H} NMR (101 MHz, $CDCl_3$) δ 152.5, 84.1, 82.9, 78.8, 50.9, 28.1.

4-(Dimethyl(naphthalen-1-yl)silyl)but-2-yn-1-ol (S33)

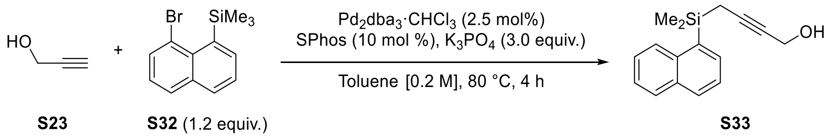

According to a reported procedure,[20] an oven-dried 8 mL microwave tube equipped with a Teflon coated stirring bar was charged with SPhos (123 mg, 300 µmol, 10.0 mol%), $Pd_2dba_3 \cdot CHCl_3$ (77.6 mg, 75.0 µmol, 2.5 mol%) and tripotassium phosphate (1.91 g, 9.00 mmol, 3.0 equiv.) in the glove box. Toluene (9 mL) was added and the mixture was stirred at 50 °C for 10 minutes. Afterwards, a solution of (8-bromonaphthalen-1-yl)-trimethylsilane **S32** (1.01 g, 3.60 mmol, 1.20 equiv.) in toluene (6 mL) and prop-2-yn-1-ol **S23** (168 mg, 175 µL, 3.00 mmol, 1.00 equiv.) were added. The resulting solution was then stirred at 80 °C for 4 h. Next, the reaction mixture was allowed to cool down to room temperature and filtered through a plug of silica gel eluting with EtOAc (100 mL) and concentrated *in vacuo*. The crude material was purified by flash column chromatography on silica gel (5-15% (v/v) EtOAc/pentane) to afford the 5-(dimethyl(naphthalen-1-yl)silyl)pent-3-yn-1-ol **S33** (303 mg, 1.19 mmol, 40% yield) as a yellow oil. Spectral data were consistent with the values reported in literature.[20]

^{1}H NMR (400 MHz, $CDCl_3$) δ 8.10 – 8.03 (m, 1H, Ar*H*), 7.92 – 7.86 (m, 2H, Ar*H*), 7.72 (dd, *J* = 6.8, 1.3 Hz, 1H, Ar*H*), 7.56 – 7.44 (m, 3H, Ar*H*), 4.21 (t, *J* = 2.6 Hz, 2H, C≡C-CH_2-OH), 1.98 (t, *J* = 2.6 Hz, 2H, Si-CH_2-C≡C), 0.59 (s, 6H, Si(CH_3)$_2$).

^{13}C{^{1}H} NMR (101 MHz, $CDCl_3$) δ 136.9, 135.4, 133.9, 133.5, 130.5, 129.4, 128.0, 126.0, 125.7, 125.2, 84.3, 78.3, 51.8, 7.2, -1.6.

***tert*-Butyl (4-hydroxybut-2-yn-1-yl)carbamate (S36)**

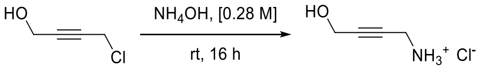

S34 **S35**

According to a reported procedure,[21] 4-chloro-2-butyn-l-ol **S34** (740 mg, 7.07 mmol) was mixed with 25 mL of concentrated ammonium hydroxide. The homogeneous mixture was stirred overnight. The mixture was evaporated by stirring on a heating plate at 90 °C with a flow of nitrogen passing through until no appreciable ammonia fumes were detectable. The crude material was recrystallized from methanol to afford the 4-hydroxybut-2-yn-1-aminium chloride **S35** as a light brown solid (227 mg, 1.62 mmol, 23% yield).

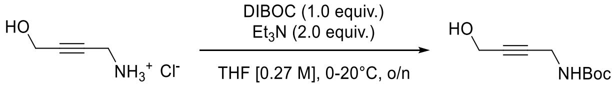

S35 **S36**

According to a reported procedure,[22] triethylamine (328 mg, 3.24 mmol, 452 µL, 2.00 equiv.) was added dropwise at 0 °C to a solution of 4-hydroxybut-2-yn-1-aminium chloride **S35** (227 mg, 1.62 mmol, 1.00 equiv.) and di-*tert*-butyl dicarbonate (357 mg, 1.63 mmol, 1.01 equiv.) in anhydrous THF (6 mL). The mixture was stirred overnight at room temperature. The solvent was evaporated and the residue was dissolved in dichloromethane (30 mL). The organic layer was washed with water (2 × 15 mL). The combined aqueous phases were extracted with dichloromethane (2 × 20 mL). The combined organic extracts were dried over $MgSO_4$, filtered and concentrated *in vacuo*. The crude material was purified by flash column chromatography on silica gel (5% (v/v) MeOH/DCM) to afford **S36** as an orange oil (209 mg, 1.12 mmol, 70% yield). Spectral data were consistent with the values reported in literature.[22]

^{1}H NMR (400 MHz, $CDCl_3$) δ 4.70 (br, 1H, N*H*Boc), 4.26 (dt, *J* = 4.0, 1.9 Hz, 2H, C≡C-CH_2-NHBoc), 3.96 (d, *J* = 5.6 Hz, 2H, C≡C-CH_2-OH), 1.45 (s, 9H, NHC(CH_3)$_3$).

^{13}C{^{1}H} NMR (101 MHz, $CDCl_3$) δ 155.5, 82.2, 81.4, 80.6, 51.2, 30.7, 28.5.

B.3. Synthesis of alkynyl bromides

2-Bromo-1-triisopropylsilyl acetylene (2a)

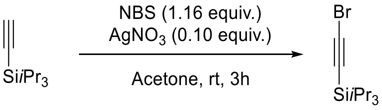

S37 **2a**

Following a reported procedure,[23] triisopropylsilylacetylene **S37** (4.07 g, 22.3 mmol, 1.00 equiv.) was dissolved in acetone (150 mL). *N*-bromosuccinimide (4.60 g, 52.9 mmol, 1.16 equiv.) was added, followed by $AgNO_3$ (381 mg, 2.23 mmol, 0.10 equiv.). The resulting mixture was stirred at room temperature for 3 h and it was then poured onto ice. After ice being allowed to melt, the aqueous layer was extracted with pentane (3 × 100 mL). The combined organic layers were dried over $MgSO_4$, filtered and concentrated in vacuo to afford pure 2-bromo-1-triisopropylsilyl acetylene **2a** (4.85 g, 18.6 mmol, 83% yield) as a colorless oil. Spectral data were consistent with the values reported in literature.[23]

^{1}H NMR (400 MHz, $CDCl_3$) δ 1.08 (d, *J* = 1.4 Hz, 21H, Si(C*H*(CH_3)$_2$)$_3$).

^{13}C{^{1}H} NMR (101 MHz, $CDCl_3$) δ 83.6, 61.9, 18.6, 11.4.

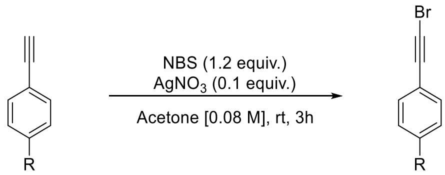

General procedure B3: According to a reported procedure,[24] NBS (1.2 equiv.) and $AgNO_3$ (0.1 equiv.) were added to a solution of aryl alkyne (1.0 equiv.) in acetone (83 mM). The resulting solution was stirred under nitrogen at room temperature for 3 hours. After removing the excess acetone, the reaction was quenched with water. The organic layer was extracted with pentane (3 × 30 mL), dried over $MgSO_4$ and concentrated under reduced pressure to obtain pure bromoalkyne.

(Bromoethynyl)benzene (2b)

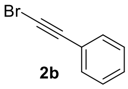

Prepared according to the general procedure B3 using phenylacetylene (102 mg, 1.00 mmol, 1.00 equiv.). No further purification was needed to give **2b** (160 mg, 0.88 mmol, 88 % yield) as a yellow oil. Spectral data were consistent with the values reported in literature.[25]

^{1}H NMR (400 MHz, $CDCl_3$) δ 7.48 – 7.42 (m, 2H, Ar*H*), 7.37 – 7.27 (m, 3H, Ar*H*).

$^{13}C\{^{1}H\}$ NMR (101 MHz, $CDCl_3$) δ 132.2, 128.8, 128.5, 122.9, 80.2, 49.9.

1-(Bromoethynyl)-4-(trifluoromethyl)benzene (2c)

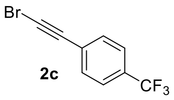

Prepared according to the general procedure B3 using 4-(trifluoromethyl)phenylacetylene (170 mg, 1.00 mmol, 1.00 equiv.). No further purification was needed to give **2c** (184 mg, 0.740 mmol, 74% yield) as a yellow solid. Spectral data were consistent with the values reported in literature.[25]

^{1}H NMR (400 MHz, $CDCl_3$) δ 7.58 (d, J = 8.7 Hz, 2H, Ar*H*), 7.55 (d, J = 8.7 Hz, 2H, Ar*H*).

$^{13}C\{^{1}H\}$ NMR (101 MHz, $CDCl_3$) δ 132.4, 130.6 (q, $J_{C\text{-}F}$ = 32.8 Hz), 126.6, 125.5 (q, $J_{C\text{-}F}$ = 3.9 Hz), 124.0 (q, $J_{C\text{-}F}$ = 272.3 Hz), 79.0, 53.1.

^{19}F NMR (376 MHz, $CDCl_3$) δ -62.9.

1-(4-(Bromoethynyl)phenyl)ethan-1-one (2d)

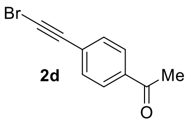

Prepared according to the general procedure B3 using 1-(4-ethynylphenyl)ethan-1-one (577 mg, 4.00 mmol, 1.00 equiv.). No further purification was needed to give **2d** (866 mg, 3.88 mmol, 97% yield) as a yellow solid. Spectral data were consistent with the values reported in literature.[26]

^{1}H NMR (400 MHz, $CDCl_3$) δ 7.86 (d, J = 8.1 Hz, 2H, Ar*H*), 7.49 (d, J = 8.1 Hz, 2H, Ar*H*), 2.56 (s, 3H, C*H*$_3$).

$^{13}C\{^{1}H\}$ NMR (101 MHz, $CDCl_3$) δ 197.2, 136.7, 132.2, 128.2, 127.5, 79.5, 53.8, 26.6.

B.4. Synthesis of L1

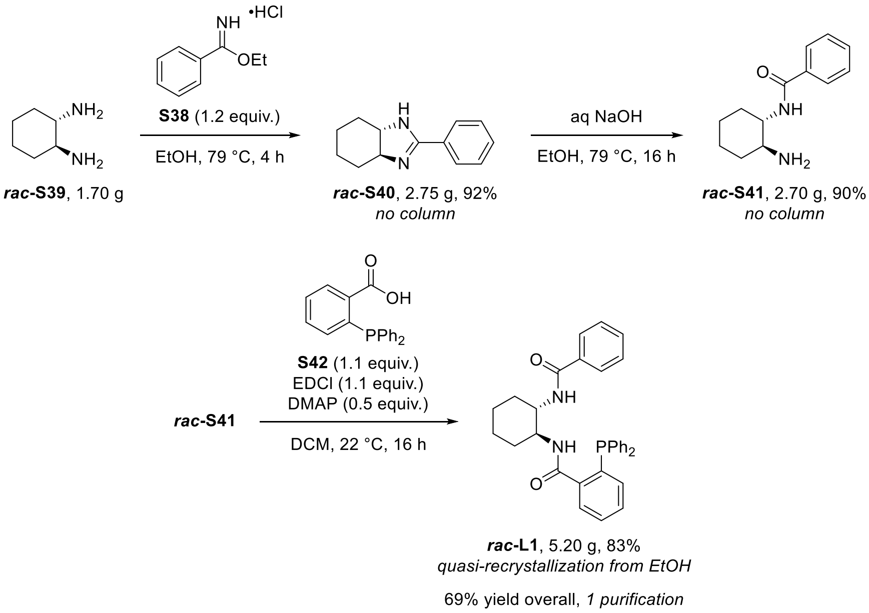

***rac*-2-Phenyl-3a,4,5,6,7,7a-hexahydro-1*H*-benzo[*d*]imidazole (*rac*-S40)**

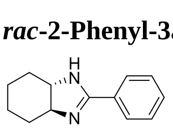

Following a reported procedure,[27] ethyl benzimidate hydrochloride **S38** (3.30 g, 18.0 mmol, 1.2 equiv.) in EtOH (15 mL) was stirred at room temperature under nitrogen and *rac*-cyclohexane-1,2-diamine **S39** (1.70 g, 15.0 mmol, 1.0 equiv.) was added to the solution in one portion. The solution was heated to reflux and stirred for 4 hours. 1 M NaOH (50 mL) was then added and the mixture was extracted with 5% MeOH in DCM. The organic layer was dried over Na_2SO_4 and concentrated to afford the crude product *rac*-**S40** (2.75 g, 13.7 mmol, 92% yield) as a white solid, which was used without further purification. Spectral data was consistent with the values reported in the literature.[27]

^{1}H NMR (400 MHz, $CDCl_3$) δ 7.81 – 7.75 (m, 2H, Ar*H*), 7.49 – 7.36 (m, 3H, Ar*H*), 5.03 (bs, 1H, N*H*), 3.25 – 3.00 (m, 2H, N-C*H*-CH$_2$ and NH-C*H*-CH$_2$), 2.49 – 2.13 (m, 2H, N-CH-C*H*$_2$), 1.93 – 1.76 (m, 2H, NH-CH-C*H*$_2$), 1.64 – 1.43 (m, 2H, -CH$_2$-C*H*$_2$-CH$_2$-), 1.43 – 1.23 (m, 2H, -CH$_2$-C*H*$_2$-CH$_2$-).

***rac*-*N*-(-2-Aminocyclohexyl)benzamide (*rac*-S41)**

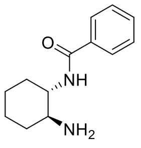

***rac*-xx**

Following a reported procedure,[27] to compound *rac*-**S40** (2.75 g, 13.7 mmol, 1.0 equiv.) was added 5% NaOH (25 mL) and EtOH/H_2O (2:1, 54 mL) and the solution was heated to reflux for 16 hours. After cooling to room temperature, the ethanol was removed in vacuum, and the crude product was extracted with DCM. The desired product *rac*-**S41** (2.70 g, 12.4 mmol, 90% yield) as a white solid, which was used without further purification. Spectral data was consistent with the values reported in the literature.[27]

^{1}H NMR (400 MHz, $CDCl_3$) δ 7.82 – 7.75 (m, 2H, Ar*H*), 7.55 – 7.39 (m, 3H, Ar*H*), 6.07 (s, 1H, N*H*), 3.78 – 3.65 (m, 1H, NHC*H*CH_2), 2.49 (td, *J* = 10.3, 4.0 Hz, 1H, NH_2-C*H*-CH_2), 2.15 (d, *J* = 12.5 Hz, 1H, NH-CH-CH_{2a}), 2.07 – 1.98 (m, 1H, NH-CH-CH_{2b}), 1.80 – 1.72 (m, 2H, NH-CH-CH_2), 1.47 – 1.15 (m, 6H, NH_2 and 2 × -CH_2-CH_2-CH_2-).

***rac*-*N*-(2-Benzamidocyclohexyl)-2-(diphenylphosphino)benzamide (*rac*-L1)**

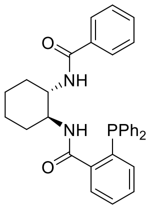

***rac*-L1**

To a stirred solution of 2-(diphenylphosphino)benzoic acid **S42** (2.96 g, 13.6 mmol, 1.1 equiv.) and DMAP (753 mg, 6.2 mmol, 0.5 equiv.) in DCM (80 mL) was added EDC·HCl (2.63 g, 13.6 mmol, 1.1 equiv.) at 0 °C. The mixture was stirred for a few minutes and allowed to reach room temperature. Then, compound *rac*-**S41** (2.70 g, 12.4 mmol, 1.0 equiv.) was added followed by DCM (20 mL). The resulting mixture was stirred at room temperature for 16 hours. The mixture was then quenched with 1 M HCl (80 mL) and extracted with DCM (2 × 50 mL). The combined organic layers were washed with brine and dried over sodium sulfate. The solvent was removed under vacuum and the crude mixture was purified by recrystallization in EtOH to obtain the desired compound **L1** (5.20 g, 10.3 mmol, 83% yield) as a white solid. Spectral data was consistent with the values reported in the literature.[28]

^{1}H NMR (400 MHz, $CDCl_3$) δ 7.84 – 7.77 (m, 2H, Ar*H*), 7.48 – 7.15 (m, 16H, Ar*H*), 7.08 – 6.97 (m, 1H, N*H*), 6.93 – 6.83 (m, 1H Ar*H*), 6.09 (d, *J* = 8.4 Hz, 1H, N*H*), 3.99 – 3.91 (m, 1H, NH-C*H*-CH_2), 3.85 – 3.73 (m, 1H, NH-C*H*-CH_2), 2.24 (d, *J* = 12.1 Hz, 1H, NH-CH-CH_{2a}), 1.88 (dd, *J* = 13.2, 2.9 Hz, 1H, NH-CH-CH_{2b}), 1.78 – 1.71 (m, 2H, NH-CH-CH_{2a} and NH-CH-CH_{2b}), 1.36 – 1.20 (m, 3H, -CH_2-CH_2-CH_2-), 1.18 – 1.03 (m, 1H, -CH_2-CH_2-CH_2-).

^{13}C{^{1}H} NMR (101 MHz, $CDCl_3$) δ 170.2, 167.7, 141.2, 140.9, 137.4, 137.1, 136.1,134.5, 134.4, 134.1, 133.9, 131.4, 130.4, 129.1, 129.0, 128.9, 128.8, 128.75, 128.7, 128.6, 127.7, 127.6 127.3, 55.6, 53.3, 32.5, 32.0, 25.0, 24.7. The peaks are listed not accounting for C-P coupling.

^{31}P NMR (162 MHz, $CDCl_3$) δ -10.90.

C. Optimization Studies

General Procedure for Optimization: An oven-dried 8 mL microwave tube equipped with a Teflon coated stirring bar was charged with Pd_2dba_3·$CHCl_3$ (2.6 mg, 2.5 μmol, 2.5 mol%), **Ligand** (10 μmol, 7 mol%) and Base (130 μmol, 1.3 equiv.). The tube was evacuated and back-filled with N_2 three times. Then, the solvent (0.5 mL) was added and the mixture was stirred at 50 °C for 15 minutes. Afterwards, the corresponding propargyl alcohol (0.100 mmol), aldimine **3d** or **3e** (0.130 mmol, 1.3 equiv.) and aryl iodide (0.130 mmol, 1.3 equiv.) were added. The resulting solution was then stirred at 80 °C for 16 h. Next, the reaction mixture was allowed to cool down to room temperature and filtered through a plug of silica gel eluting with DCM in pentane (10 mL) and the mixture was concentrated in vacuo. The crude material was purified by flash column chromatography on silica gel to afford the corresponding product.

C.1. Ligand screening

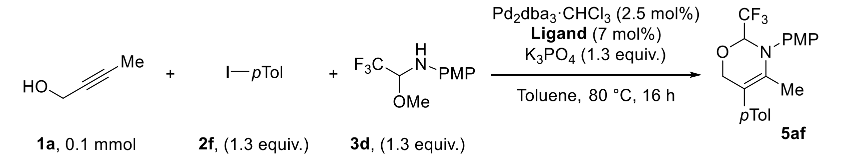

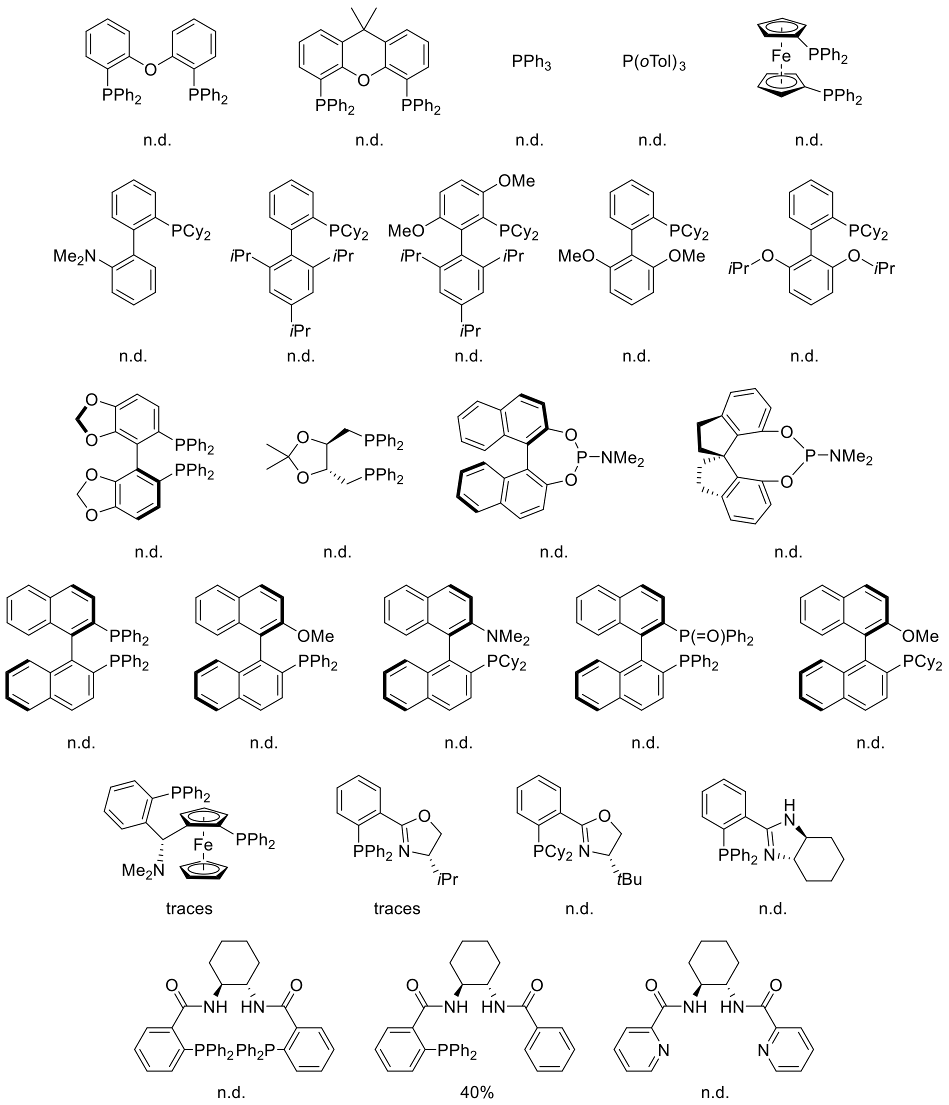

C.2. Optimization of reaction conditions

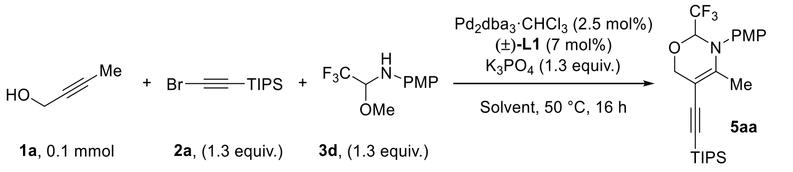

Entry	Solvent	Yield[a] (%)
**1**	Toluene	40
**2**	DCM	47
**3**	DME	20
**4**	MeCN	38
**5**	EtOAc	62
**6**	THF	45
**7**	Acetone	0
**8**	DMF	0

[a] NMR yield using trichloroethylene as an internal standard.

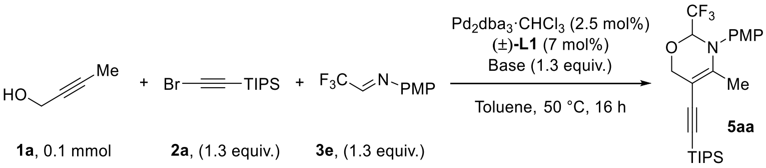

Entry	Base	Yield[a] (%)
**1**	K_3PO_4	62
**2**	Na_3PO_4	35
**3**	K_2CO_3	17
**4**	Cs_2CO_3	75
**5**	Cs_2CO_3	0[b]
**6**	Cs_2CO_3	82[c]
**7**	KOH	20
**8**	Et_3N	0
**9**	DBU	0

[a] NMR yield using trichloroethylene as an internal standard. [b] in EtOAc. [c] in DCM.

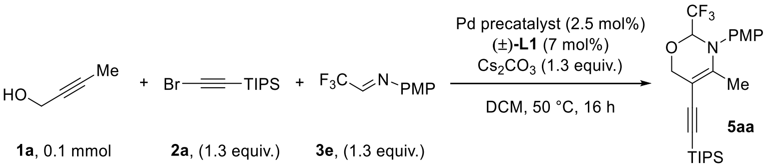

Entry	Pd precatalyst	Yield[a] (%)
**1**	$Pd_2dba_3 \cdot CHCl_3$	82
**2**	$Pd(OAc)_2$	58
**3**	$Pd(PhCN)_2Cl_2$	15
**4**	PdCpCinammyl	68
**5**	No Pd, L or base	0

[a] NMR yield using trichloroethylene as an internal standard.

D. Procedures and product characterization data of carboamination products (enamines)

D.1. General Procedure D1 for the 6-*endo-dig* carboamination reaction

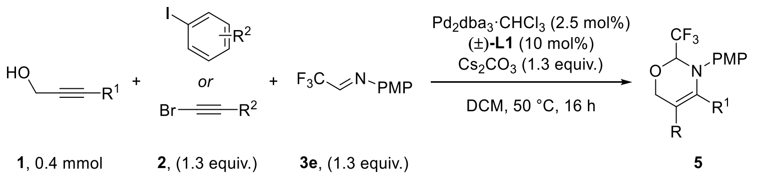

An oven-dried 8 mL microwave tube equipped with a Teflon coated stirring bar was charged with $Pd_2dba_3{\cdot}CHCl_3$ (10.4 mg, 10.0 µmol, 2.5 mol%), **(±)-L1** (20.3 mg, 40.0 µmol, 10 mol%) and Cs_2CO_3 (169 mg, 40.0 µmol, 1.3 equiv.). The tube was evacuated and back-filled with N_2 three times. Then, DCM (2.0 mL) was added and the mixture was stirred at 50 °C for 15 minutes. Afterwards, the corresponding propargyl alcohol (0.400 mmol), aldimine **3e** (114 mg, 90.0 µL, 0.560 mmol, 1.4 equiv.) and the corresponding bromoalkyne or aryl iodide (0.520 mmol) were added. The resulting solution was then stirred at 50 °C for 16 h. Next, the reaction mixture was allowed to cool down to room temperature and filtered through a plug of silica gel eluting with DCM in pentane (25 mL). Ethanolamine (101 mg, 100 µL, 1.66 mmol) was added and the mixture was concentrated in vacuo. The crude material was purified by flash column chromatography on silica gel using a Biotage flash chromatography machine to afford the corresponding product.

D.2. Characterization of the 6-*endo-dig* carboamination products

3-(4-Methoxyphenyl)-4-methyl-2-(trifluoromethyl)-5-((triisopropylsilyl)ethynyl)-3,6-dihydro-2*H*-1,3-oxazine (5aa)

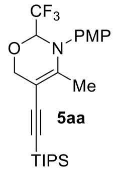

5aa

Prepared according to the general procedure D1 using but-2-yn-1-ol **1a** (28 mg, 30 µL, 0.40 mmol, 1.0 equiv.) and 1-bromo-2-(triisopropylsilyl)acetylene **2a** (136 mg, 120 µ, 520 µmol, 1.30 equiv.). The crude material was purified by column chromatography (0 – 5 % (v/v) Et_2O in pentane) to give **5aa** (135 mg, 0.298 mmol, 75% yield) as a pale-yellow oil.

R_f (20 % DCM/Pentane) = 0.50.

^{1}H NMR (400 MHz, $CDCl_3$) δ 7.16 – 7.04 (m, 2H, Ar*H*), 6.92 – 6.80 (m, 2H, Ar*H*), 4.93 (q, *J* = 6.5 Hz, 1H, CH-CF_3), 4.43 (dp, *J* = 14.5, 1.6 Hz, 1H, O-CH_2-C), 4.34 (dp, *J* = 14.5, 1.7 Hz, 1H, O-CH_2-C), 3.81 (s, 3H, O-CH_3), 1.90 (t, *J* = 1.8 Hz, 3H, C=C-CH_3), 1.16 – 0.98 (m, 21H, Si(C*H*(CH_3)$_2$)$_3$).

^{13}C{^{1}H} NMR (101 MHz, $CDCl_3$) δ 158.4, 142.9, 138.6, 128.1, 123.2 (q, $J_{C\text{-}F}$ = 288.8 Hz), 114.7,103.3, 97.1, 94.3, 85.1 (q, $J_{C\text{-}F}$ = 33.4 Hz), 63.9, 55.7, 18.83, 18.78, 11.5.

^{19}F NMR (376 MHz, $CDCl_3$) δ -75.9 (d, *J* = 6.2 Hz).

IR (cm^{-1}) 2940 (m), 2868 (m), 2137 (m), 1636 (m), 1620 (w), 1510 (s), 1461 (m), 1385 (m), 1248 (s), 1164 (s), 1120 (s).

HRMS (ESI/QTOF) m/z: [M + H]$^+$ Calcd for $C_{24}H_{35}F_3NO_2Si^+$ 454.2384; Found 454.2383.

3-(4-Methoxyphenyl)-4-phenyl-2-(trifluoromethyl)-5-((triisopropylsilyl)ethynyl)-3,6-dihydro-2*H*-1,3-oxazine (5ba)

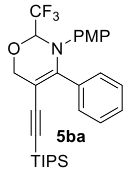

5ba

Prepared according to the general procedure D1 using 3-phenylprop-2-yn-1-ol **1b** (53 mg, 50 µL, 0.40 mmol, 1.00 equiv.) and 1-bromo-2-(triisopropylsilyl)acetylene **2a** (136 mg, 120 µL, 520 µmol, 1.30 equiv.). The crude material was purified by column chromatography (0 – 5 % (v/v) EtOAc in pentane) to give **5ba** (165 mg, 0.320 mmol, 80% yield) as a pale-yellow oil. The product is coeluting with an isomeric product in a 15:1 ratio determined by integration of the ^{19}F NMR spectra. The ratio was found to be unchanged before and after the purification.

R_f (20 % DCM/Pentane) = 0.44.

^{1}H NMR (400 MHz, $CDCl_3$) δ 7.61 – 7.55 (m, 2H, Ar*H*), 7.20 – 7.10 (m, 3H, Ar*H*), 6.99 – 6.92 (m, 2H, Ar*H*), 6.71 – 6.63 (m, 2H, Ar*H*), 5.18 (q, *J* = 6.4 Hz, 1H, C*H*-CF_3), 4.57 (dq, *J* = 15.3, 1.5 Hz, 1H, O-CH_aCH_b-C=C), 4.50 (dq, *J* = 15.4, 1.3 Hz, 1H, O-CH_aCH_b-C=C), 3.68 (s, 3H, O-CH_3), 1.02 – 0.96 (m, 21H, Si(C*H*(CH_3)$_2$)$_3$).

^{13}C{^{1}H} NMR (101 MHz, $CDCl_3$) δ 157.1, 144.5, 140.0, 135.2, 129.6, 128.5, 127.8, 126.2, 123.2 (d, $J_{C\text{-}F}$ = 288.2 Hz), 114.4, 94.8, 85.7 (q, $J_{C\text{-}F}$ = 33.6 Hz), 64.8, 55.5, 18.7 (2C), 11.4.

^{19}F NMR (376 MHz, $CDCl_3$) δ -75.7 (d, *J* = 6.3 Hz).

IR (cm^{-1}) 2971 (s), 2892 (s), 2857 (m), 2134 (w), 1629 (w), 1507 (m), 1391 (m), 1248 (s), 1176 (m), 1162 (m).

HRMS (ESI/QTOF) m/z: [M + H]$^+$ Calcd for $C_{29}H_{37}F_3NO_2Si^+$ 516.2540; Found 516.2540.

3,4-Bis(4-methoxyphenyl)-2-(trifluoromethyl)-5-((triisopropylsilyl)ethynyl)-3,6-dihydro-2*H*-1,3-oxazine (5ca)

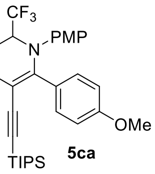

5ca

Prepared according to the general procedure D1 using 3-(4-methoxyphenyl)prop-2-yn-1-ol **1c** (65 mg, 0.40 mmol, 1.0 equiv.) and 1-bromo-2-(triisopropylsilyl)acetylene **2a** (136 mg, 120 μ, 520 μmol, 1.30 equiv.). The crude material was purified by column chromatography (0 – 5 % (v/v) EtOAc in pentane) and then preparative TLC (20 % (v/v) DCM in pentane) to give **5ca** (199 mg, 0.365 mmol, 91% yield) as a pale-yellow oil.

R_f (20 % DCM/Pentane) = 0.86.

^{1}H NMR (400 MHz, $CDCl_3$) δ 7.56 – 7.51 (m, 2H, Ar*H*), 6.98 – 6.92 (m, 2H, Ar*H*), 6.73 – 6.65 (m, 4H, Ar*H*), 5.17 (q, *J* = 6.4 Hz, 1H, C*H*-CF_3), 4.55 (dq, *J* = 15.1, 1.4 Hz, 1H, O-C$H_a$$CH_b$-C=C), 4.48 (dq, *J* = 15.1, 1.3 Hz, 1H, O-CH_aCH_b-C=C), 3.72 (s, 3H, O-CH_3), 3.69 (s, 3H, O-CH_3), 1.05 – 0.97 (m, 21H, Si(C*H(*CH_3)$_2$)$_3$).

^{13}C{^{1}H} NMR (101 MHz, $CDCl_3$) δ 159.7, 157.0, 144.2, 140.2, 130.9, 127.7, 126.1, 123.2 (q, $J_{C\text{-}F}$ = 288.2 Hz), 114.4, 113.2, 103.6, 102.1, 94.5, 85.7 (q, $J_{C\text{-}F}$ = 33.8 Hz), 64.9, 55.5, 55.3, 18.8, 11.5.

^{19}F NMR (376 MHz, $CDCl_3$) δ -75.6 (d, *J* = 6.3 Hz).

IR (cm^{-1}) 2956 (s), 2917 (m), 2866 (m), 2132 (m), 1610 (m), 1507 (s), 1464 (m), 1377 (m), 1282 (m), 1237 (s).

HRMS (ESI/QTOF) m/z: [M + H]$^+$ Calcd for $C_{30}H_{39}F_3NO_3Si^+$ 546.2646; Found 546.2649.

3-(4-Methoxyphenyl)-4-(4-fluorophenyl)-2-(trifluoromethyl)-5-((triisopropylsilyl)ethynyl)-3,6-dihydro-2*H*-1,3-oxazine (5da)

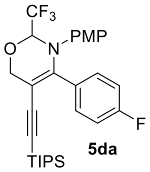

5da

Prepared according to the general procedure D1 using 3-(4-fluorophenyl)prop-2-yn-1-ol **1d** (60.1 mg, 400 μmol, 1.00 equiv.) and 1-bromo-2-(triisopropylsilyl)acetylene **2a** (136 mg, 520 μmol, 1.30 equiv.). The crude material was purified by column chromatography (0 – 5 % (v/v) Et_2O in pentane) to give **5da** (126 mg, 0.236 mmol, 59% yield) as a pale-yellow oil. The product is coeluting with an isomeric product in a 16:1 ratio determined by integration of the ^{19}F NMR spectra of CH-C*F*$_3$. The ratio was found to be unchanged before and after the purification.

R_f (10% Et_2O/Pentane) = 0.66.

^{1}H NMR (400 MHz, $CDCl_3$) δ 7.61 – 7.51 (m, 2H, Ar*H*), 6.97 – 6.91 (m, 2H, Ar*H*), 6.90 – 6.83 (m, 2H, Ar*H*), 6.70 – 6.65 (m, 2H, Ar*H*), 5.16 (q, *J* = 6.4 Hz, 1H, CF_3-C*H*), 4.56 (dd, *J* = 15.4, 1.5 Hz, 1H, O-CH_2-C), 4.49 (dd, *J* = 15.4, 1.3 Hz, 1H, O-CH_2-C), 3.70 (s, 3H, Ar-O-CH_3), 1.00 (s, 21H, Si(C*HCH*$_3$)$_3$)

^{13}C{^{1}H} NMR (101 MHz, $CDCl_3$) δ 162.6 (d, $J_{C\text{-}F}$ = 248.2 Hz), 157.3, 143.5, 139.8, 131.4 (d, $J_{C\text{-}F}$ = 8.3 Hz), 131.3 (d, $J_{C\text{-}F}$ = 3.3 Hz), 126.3, 123.2 (q, $J_{C\text{-}F}$ = 287.8 Hz), 114.9 (d, $J_{C\text{-}F}$ = 21.7 Hz), 114.5, 103.1, 103.0, 95.0, 85.7 (q, $J_{C\text{-}F}$ = 33.9 Hz), 64.7, 55.5, 18.7, 11.4.

^{19}F NMR (376 MHz, $CDCl_3$) δ -75.7, -112.6.

IR (cm^{-1}) 2950 (m), 2860 (m), 2132 (w), 1624 (m), 1510 (s), 1465 (m), 1383 (m), 1248 (s), 1176 (s), 1144 (s).

HRMS (APPI/LTQ-Orbitrap) m/z: [M]$^+$ Calcd for $C_{29}H_{35}F_4NO_2Si^+$ 533.2368; Found 533.2374.

3-(4-Methoxyphenyl)-4-(4-chlorophenyl)-2-(trifluoromethyl)-5-((triisopropylsilyl)ethynyl)-3,6-dihydro-2*H*-1,3-oxazine (5ea)

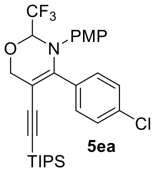

5ea

Prepared according to the general procedure D1 using 3-(4-chlorophenyl)prop-2-yn-1-ol **1e** (66.6 mg, 400 μmol, 1.00 equiv.) and 1-bromo-2-(triisopropylsilyl)acetylene **2a** (136 mg, 520 μmol, 1.30 equiv.). The crude material was purified by column chromatography (0 – 5 % (v/v) Et_2O in pentane) to give **5ea** (86 mg, 0.16 mmol, 39% yield) as a pale-yellow oil. The product is coeluting with an isomeric product in a 9:1 ratio determined by integration of the ^{19}F NMR spectra. The ratio was found to be unchanged before and after the purification.

R_f (10% Et_2O/Pentane) = 0.66.

^{1}H NMR (400 MHz, $CDCl_3$) δ 7.56 – 7.49 (m, 2H, Ar*H*), 7.17 – 7.12 (m, 2H, Ar*H*), 6.96 – 6.91 (m, 2H, Ar*H*), 6.73 – 6.65 (m, 2H, Ar*H*), 5.16 (q, *J* = 6.4 Hz, 1H, CF_3-C*H*), 4.55 (dd, *J* = 15.5, 1.5 Hz, 1H, O-CH_2-C), 4.48 (dd, *J* = 15.4, 1.4 Hz, 1H, O-CH_2-C), 3.70 (s, 3H, Ar-O-CH_3), 1.01 (s, 21H, Si(C*HCH*$_3$)$_3$).

^{13}C{^{1}H} NMR (101 MHz, $CDCl_3$) δ 157.3, 143.3, 139.7, 134.2, 133.7, 130.9, 128.1, 126.2, 123.1 (q, $J_{C\text{-}F}$ = 288.4 Hz), 114.5, 103.6, 102.7, 95.6, 85.6 (q, $J_{C\text{-}F}$ = 33.6 Hz), 64.7, 55.5, 18.7, 11.4.

^{19}F NMR (376 MHz, $CDCl_3$) δ -75.7 (d, *J* = 6.3 Hz).

IR (cm^{-1}) 2946 (w), 2255 (w), 1794 (w), 1474 (w), 1386 (w), 1281 (w), 1087 (w).

HRMS (APPI/LTQ-Orbitrap) m/z: [M]$^+$ Calcd for $C_{29}H_{35}ClF_3NO_2Si^+$ 549.2072; Found 549.2094.

3-(4-Methoxyphenyl)-4-(4-(trifluoromethyl)phenyl)-2-(trifluoromethyl)-5-((triisopropylsilyl)ethynyl)-3,6-dihydro-2*H*-1,3-oxazine (5fa)

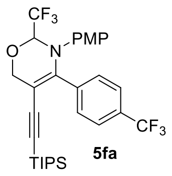

5fa

Prepared according to the general procedure D1 using 3-(4-(trifluoromethyl)phenyl)prop-2-yn-1-ol **1f** (80.1 mg, 400 μmol, 1.00 equiv.) and 1-bromo-2-(triisopropylsilyl)acetylene **2a** (136 mg, 520 μmol, 1.30 equiv.). The crude material was purified by column chromatography (0 – 5 % (v/v) Et_2O in pentane) to give **5f** (148 mg, 0.254 mmol, 63% yield) as a pale-yellow oil. The product is coeluting with an isomeric product in a 3:2 ratio determined by integration of the ^{19}F NMR spectra of CH-C*F*$_3$. The ratio was found to be unchanged before and after the purification.

R_f (5% Et_2O/Pentane) = 0.63.

Major isomer:

^1H NMR (400 MHz, $CDCl_3$) δ 7.73 – 7.63 (m, 2H, Ar*H*), 7.49 – 7.39 (m, 2H, Ar*H*), 7.02 – 6.91 (m, 2H, Ar*H*), 6.75 – 6.64 (m, 2H, Ar*H*), 5.17 (q, *J* = 5.7 Hz, 1H, CF_3-C*H*), 4.58 (dd, *J* = 15.6, 1.5 Hz, 1H, O-C*H*$_2$-C), 4.50 (dd, *J* = 15.6, 1.3 Hz, 1H, O-C*H*$_2$-C), 3.70 (s, 3H, Ar-O-C*H*$_3$), 0.98 (d, *J* = 1.1 Hz, 21H, Si(C*HCH*$_3$)$_3$).

^{19}F NMR (376 MHz, $CDCl_3$) δ -62.8, -75.7 (d, *J* = 6.3 Hz).

Minor isomer:

^1H NMR (400 MHz, $CDCl_3$) δ 7.33 – 7.27 (m, 2H, Ar*H*), 7.23 (d, *J* = 8.3 Hz, 2H, Ar*H*), 6.88 – 6.75 (m, 2H, Ar*H*), 6.60 – 6.48 (m, 2H, Ar*H*), 5.33 (q, *J* = 4.8 Hz, 1H, CF_3-C*H*), 5.25 – 5.19 (m, 1H, O-C*H*$_2$-C), 5.12 (d, *J* = 13.8 Hz, 1H, O-C*H*$_2$-C), 3.65 (s, 3H, Ar-O-C*H*$_3$), 1.12 (s, 21H, Si(C*HCH*$_3$)$_3$).

^{19}F NMR (376 MHz, $CDCl_3$) δ -62.5, -80.4 (d, *J* = 4.8 Hz).

Given as a list of peaks due to the heavy overlap of peaks:

$^{13}C\{^1H\}$ NMR (101 MHz, $CDCl_3$) δ 157.6, 157.4, 147.9, 143.2, 139.5, 138.9, 134.0, 130.4, 129.9, 128.1, 126.2, 124.9, 124.9, 124.6, 124.32, 124.28, 114.8, 114.6, 114.3, 105.6, 104.7, 102.3, 95.9, 94.9, 94.3, 85.8, 85.4, 73.8, 64.6, 55.6, 55.5, 18.90, 18.88, 18.7, 11.5, 11.4.

IR (cm^{-1}) 2945 (m), 2867 (m), 2135 (w), 1620 (m), 1512 (s), 1324 (s), 1246 (s), 1145 (s), 1065 (s).

HRMS (ESI/QTOF) m/z: $[M + H]^+$ Calcd for $C_{30}H_{36}F_6NO_2Si^+$ 584.2414; Found 584.2419.

3-(4-Methoxyphenyl)-4-(3-fluorophenyl)-2-(trifluoromethyl)-5-((triisopropylsilyl)ethynyl)-3,6-dihydro-2*H*-1,3-oxazine (5ga)

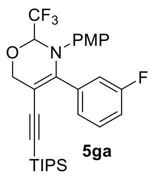

5ga

Prepared according to the general procedure D1 using 3-(3-fluorophenyl)prop-2-yn-1-ol **1g** (60.1 mg, 400 μmol, 1.00 equiv.) and 1-bromo-2-(triisopropylsilyl)acetylene **2a** (136 mg, 520 μmol, 1.30 equiv.). The crude material was purified by column chromatography (0 – 5 % (v/v) Et_2O in pentane) to give **5ga** (61 mg, 0.11 mmol, 29% yield) as a pale-yellow oil. The product is coeluting with an isomeric product in a 5:1 ratio determined by integration of the ^{19}F NMR spectra of CH-C*F*$_3$. The ratio was found to be unchanged before and after the purification.

R_f (5% Et_2O/Pentane) = 0.60.

^1H NMR (400 MHz, $CDCl_3$) δ 7.39 – 7.29 (m, 2H, Ar*H*), 7.14 (td, *J* = 8.0, 5.9 Hz, 1H, Ar*H*), 6.97 – 6.94 (m, 2H, Ar*H*), 6.90 – 6.85 (m, 1H, Ar*H*), 6.74 – 6.66 (m, 2H, Ar*H*), 5.16 (q, *J* = 6.4 Hz, 1H, CF_3-C*H*), 4.57 (dq, *J* = 15.5, 1.5 Hz, 1H, O-C*H*$_2$-C), 4.49 (dd, *J* = 15.5, 1.3 Hz, 1H, O-C*H*$_2$-C), 3.70 (s, 3H, Ar-O-C*H*$_3$), 1.01 (s, 21H, Si(C*HCH*$_3$)$_3$).

$^{13}C\{^1H\}$ NMR (101 MHz, $CDCl_3$) δ 162.4 (d, *J* = 245.0 Hz), 157.3, 143.2, 139.7, 137.4 (d, J_{C-F} = 8.1 Hz), 129.3 (d, J_{C-F} = 8.3 Hz), 126.1, 125.3 (d, J_{C-F} = 3.0 Hz), 123.1 (q, J_{C-F} = 288.2 Hz), 116.6 (d, J_{C-F} = 22.6 Hz), 115.5 (d, J_{C-F} = 21.3 Hz), 114.5, 104.0, 102.5, 95.8, 85.6 (q, J_{C-F} = 33.8 Hz), 64.7, 55.5, 18.7, 11.4.

^{19}F NMR (376 MHz, $CDCl_3$) δ -75.7 (d, *J* = 6.2 Hz), -80.6 (d, *J* = 4.8 Hz).

IR (cm^{-1}) 2944 (m), 2863 (m), 2135 (m), 1617 (m), 1584 (m), 1510 (s), 1324 (m), 1246 (s), 1173 (s), 1137 (s), 1087 (s).

HRMS (ESI/QTOF) m/z: $[M + H]^+$ Calcd for $C_{29}H_{36}F_4NO_2Si^+$ 534.2446; Found 534.2441.

3-(4-Methoxyphenyl)-4-(5-methylthiophen-2-yl)-2-(trifluoromethyl)-5-((triisopropylsilyl)ethynyl)-3,6-dihydro-2*H*-1,3-oxazine (5ha)

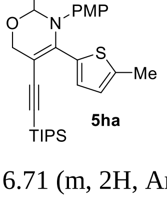

5ha

Prepared according to the general procedure D1 using 3-(thiophen-2-yl)prop-2-yn-1-ol **1h** (61 mg, 0.40 mmol, 1.0 equiv.) and 1-bromo-2-(triisopropylsilyl)acetylene **2a** (136 mg, 120 μL, 520 μmol, 1.30 equiv.). The crude material was purified by column chromatography (0 – 5% (v/v) EtOAc in pentane) to give **5ha** (189 mg, 0.324 mmol, 81% yield) as a pale-yellow oil.

R_f (20 % DCM/Pentane) = 0.46.

^1H NMR (400 MHz, $CDCl_3$) δ 7.29 (d, *J* = 3.6 Hz, 1H, HetAr*H*), 7.06 – 7.00 (m, 2H, Ar*H*), 6.80 – 6.71 (m, 2H, Ar*H*), 6.50 (dq, *J* = 3.7, 1.1 Hz, 1H, HetAr*H*), 5.14 (q, *J* = 6.3 Hz, 1H, C*H*CF_3), 4.52 (dd, *J* = 15.2, 1.4 Hz, 1H, O-C*H*$_a$CH$_b$-C=C), 4.47 (dd, *J* = 15.3, 1.2 Hz, 1H, O-CH$_a$C*H*$_b$-C=C), 3.74 (s, 3H, O-C*H*$_3$), 1.12 – 1.03 (m, 21H, Si(C*H*(C*H*$_3$)$_2$)$_3$).

$^{13}C\{^1H\}$ NMR (101 MHz, $CDCl_3$) δ 157.2, 141.7, 140.7, 138.5, 135.7, 129.7, 125.5, 124.9, 123.0 (d, J_{C-F} = 287.5 Hz), 114.5, 103.2, 103.0, 98.4, 85.9 (q, J_{C-F} = 34.0 Hz), 64.9, 55.5, 18.8, 15.4, 11.5.

^{19}F NMR (376 MHz, $CDCl_3$) δ -75.8 (d, *J* = 6.2 Hz).

IR (cm^{-1}) 2958 (m), 2898 (m), 2866 (m), 2131 (m), 1614 (m), 1508 (s), 1464 (m), 1385 (m), 1298 (m), 1269 (m), 1245 (s).

HRMS (ESI/QTOF) m/z: $[M + H]^+$ Calcd for $C_{28}H_{37}F_3NO_2SSi^+$ 536.2261; Found 536.2261.

4-Cyclopropyl-3-(4-methoxyphenyl)-2-(trifluoromethyl)-5-((triisopropylsilyl)ethynyl)-3,6-dihydro-2*H*-1,3-oxazine (5ia)

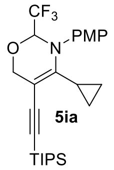

Prepared according to the general procedure D1 using 3-cyclopropylprop-2-yn-1-ol **1i** (38 mg, 36 µL, 0.40 mmol, 1.0 equiv.) and 1-bromo-2-(triisopropylsilyl)acetylene **2a** (136 mg, 120 µ, 520 µmol, 1.30 equiv.). The crude material was purified by column chromatography (0 – 5 % (v/v) EtOAc in pentane) to give **5ia** (123 mg, 0.256 mmol, 64% yield) as a pale-yellow oil.

R_f (20 % DCM/Pentane) = 0.46.

^1H NMR (400 MHz, $CDCl_3$) δ 7.10 – 7.00 (m, 2H, Ar*H*), 6.91 – 6.75 (m, 2H, Ar*H*), 4.97 (q, *J* = 6.4 Hz, 1H, C*H*-CF_3), 4.35 (p, *J* = 1.3 Hz, 2H, O-CH_2-C=C), 3.80 (s, 3H, O-CH_3), 1.63 (dddt, *J* = 11.9, 8.4, 5.4, 1.4 Hz, 1H, C*H*($CH_aH_bCH_aH_b$)), 1.12 – 1.07 (m, 21H, Si(C*H*(CH_3)$_2$)$_3$), 0.80 (tdd, *J* = 9.0, 6.5, 4.3 Hz, 1H, CH(C$H_a$$H_b$$CH_aH_b$)), 0.70 (dddd, *J* = 9.4, 6.6, 5.2, 4.3 Hz, 1H, CH($CH_a$$H_b$$CH_aH_b$)), 0.51 (dddd, *J* = 9.4, 6.4, 5.5, 4.8 Hz, 1H, CH(CH_aH_bC$H_a$$H_b$)), 0.37 (dddd, *J* = 9.2, 8.3, 6.5, 4.8 Hz, 1H, CH($CH_aH_bCH_a$$H_b$)).

$^{13}C\{^1H\}$ NMR (101 MHz, $CDCl_3$) δ 157.6, 146.5, 140.1, 126.1, 123.0 (d, J_{C-F} = 288.4 Hz), 114.5, 102.64, 102.62, 96.5, 85.7 (q, J_{C-F} = 33.5 Hz), 64.3, 55.6, 18.8 (2C), 14.0, 11.5, 9.4, 6.4.

^{19}F NMR (376 MHz, $CDCl_3$) δ -75.9 (d, *J* = 6.3 Hz).

IR (cm^{-1}) 2971 (s), 2899 (s), 2869 (s), 2128 (w), 1628 (w), 1513 (m), 1384 (m), 1248 (s).

HRMS (ESI/QTOF) m/z: $[M + H]^+$ Calcd for $C_{26}H_{37}F_3NO_2Si^+$ 480.2540; Found 480.2536.

4-(((*tert*-Butyldimethylsilyl)oxy)methyl)-3-(4-methoxyphenyl)-2-(trifluoromethyl)-5-((triisopropylsilyl)ethynyl)-3,6-dihydro-2*H*-1,3-oxazine (5ja)

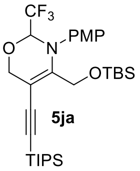

Prepared according to the general procedure D1 using 4-((*tert*-butyldimethylsilyl)oxy)but-2-yn-1-ol **1j** (80 mg, 0.40 mmol, 1.0 equiv.) and 1-bromo-2-(triisopropylsilyl)acetylene **2a** (136 mg, 120 µL, 520 µmol, 1.30 equiv.). The crude material was purified by column chromatography (0 – 5 % (v/v) EtOAc in pentane) and then by preparative TLC (20 % (v/v) DCM in pentane) to give **5ja** (144 mg, 0.247 mmol, 62% yield) as a pale-yellow oil.

R_f (20 % DCM/Pentane) = 0.44.

^1H NMR (400 MHz, $CDCl_3$) δ 7.20 – 7.13 (m, 2H, Ar*H*), 6.87 – 6.80 (m, 2H, Ar*H*), 4.92 (q, *J* = 6.4 Hz, 1H, C*H*-CF_3), 4.58 (d, *J* = 12.6 Hz, 1H, C$H_a$$H_b$-O-Si), 4.43 (dt, *J* = 15.1, 1.5 Hz, 1H, O-C$H_a$$CH_b$-C=C), 4.36 (dp, *J* = 15.0, 1.8 Hz, 1H, O-CH_aCH_b-C=C), 4.07 (ddd, *J* = 12.6, 2.2, 1.2 Hz, 1H, $CH_a$$H_b$-O-Si), 3.80 (s, 3H, OC$H_3$), 1.13 – 1.01 (m, 21H, Si(C*H*(CH_3)$_2$)$_3$), 0.81 (s, 9H, OSi$(CH_3)_2$C(CH_3)$_3$), -0.07 (s, 3H, OSi(CH_3)$_a$$(CH_3)_b$C(C$H_3$)$_3$), -0.13 (s, 3H, OSi$(CH_3)_a$(C$H_3$)$_b$C(C$H_3$)$_3$).

$^{13}C\{^1H\}$ NMR (101 MHz, $CDCl_3$) δ 158.3, 145.1, 138.3, 128.4, 122.9 (d, J_{C-F} = 288.8 Hz), 114.2, 101.9, 98.3, 95.6, 85.2 (d, J_{C-F} = 33.6 Hz), 63.8, 59.6, 55.5, 25.7, 18.7, 18.0, 11.3, -5.6, -5.7.

^{19}F NMR (376 MHz, $CDCl_3$) δ -75.6 (d, *J* = 6.3 Hz).

IR (cm^{-1}) 2955 (s), 2900 (m), 2864 (s), 2142 (w), 1632 (m), 1510 (s), 1465 (m), 1395 (m), 1313 (m), 1248 (s), 1163 (s).

HRMS (ESI/QTOF) m/z: $[M + H]^+$ Calcd for $C_{30}H_{49}F_3NO_3Si_2^+$ 584.3198; Found 584.3199.

3-(4-Methoxyphenyl)-2-(trifluoromethyl)-5-((triisopropylsilyl)ethynyl)-4-vinyl-3,6-dihydro-2*H*-1,3-oxazine (5ka)

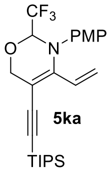

Prepared according to the general procedure D1 using pent-4-en-2-yn-1-ol **1k** (33 mg, 0.40 mmol, 1.0 equiv.) and 1-bromo-2-(triisopropylsilyl)acetylene **2a** (136 mg, 120 µL, 520 µmol, 1.30 equiv.). The crude material was purified by column chromatography (0 – 5% (v/v) EtOAc in pentane) to give **5ka** (133 mg, 0.286 mmol, 71% yield) as a pale-yellow oil. The product is coeluting with a with an isomeric product in a 3:1 ratio determined by integration of the ^{19}F-NMR spectra. The ratio was found to be unchanged before and after the purification.

R_f (20 % DCM/Pentane) = 0.40.

Major isomer:

^{1}H NMR (400 MHz, $CDCl_3$) δ 7.07 – 6.98 (m, 2H, Ar*H*), 6.85 – 6.78 (m, 2H, Ar*H*), 6.64 (dd, *J* = 17.5, 11.1 Hz, 1H, C*H*=CH_2), 5.25 (dd, *J* = 17.5, 1.2 Hz, 1H, CH=C$H_a$$H_b$), 5.16 (dd, *J* = 11.2, 1.1 Hz, 1H, CH=C$H_a$$H_b$), 4.46 (s, 2H, O-C$H_2$-C=C), 3.78 (s, 3H, O-C$H_3$), 1.11 – 1.08 (m, 21H, Si(C*H*(CH_3)$_2$)$_3$).

^{19}F NMR (376 MHz, $CDCl_3$) δ -75.9 (d, *J* = 6.4 Hz).

Minor isomer:

^{1}H NMR (400 MHz, $CDCl_3$) δ 7.18 – 7.10 (m, 2H, Ar*H*), 6.91 – 6.85 (m, 2H, Ar*H*), 5.88 (dd, *J* = 16.8, 10.3 Hz, 1H, C*H*=CH_2), 5.38 (dd, *J* = 16.8, 1.8 Hz, 1H, CH=C$H_a$$H_b$), 5.19 (d, *J* = 4.8 Hz, 1H), 5.10 (d, *J* = 13.6 Hz, 1H, O-C$H_a$$H_b$-C=C), 4.98 (d, *J* = 13.8 Hz, 1H, O-C$H_a$$H_b$C=C), 4.82 (dd, *J* = 10.4, 1.8 Hz, 1H, CH=C$H_a$$H_b$), 3.81 (s, 3H, O-C$H_3$), 1.11 – 1.10 (m, 21H, Si(C*H*(CH_3)$_2$)$_3$).

^{19}F NMR (376 MHz, $CDCl_3$) δ -80.7 (d, *J* = 4.8 Hz).

Given as a list of peaks due to the heavy overlap of peaks:

^{13}C{^{1}H} NMR (101 MHz, $CDCl_3$) δ 158.2, 157.3, 147.3, 142.5, 141.0, 136.4, 130.3, 130.0, 126.4, 125.7, 123.0 (q, J_{C-F} = 287.9 Hz), 119.9, 115.0, 114.5, 113.4, 106.6, 102.6, 101.6, 98.9, 95.8, 95.6, 95.4, 95.0, 86.1 (q, J_{C-F} = 33.7 Hz), 73.2, 64.2, 55.6, 18.9, 18.8, 11.5, 11.4.

HRMS (ESI/QTOF) m/z: $[M + H]^+$ Calcd for $C_{25}H_{35}F_3NO_2Si^+$ 466.2384; Found 466.2375.

3-(4-Methoxyphenyl)-4,6-dimethyl-2-(trifluoromethyl)-5-((triisopropylsilyl)ethynyl)-3,6-dihydro-2*H*-1,3-oxazine (5la)

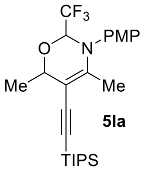

5la

Prepared according to the general procedure D1 using pent-3-yn-2-ol **1l** (33.7 mg, 400 μmol, 1.00 equiv.) and 1-bromo-2-(triisopropylsilyl)acetylene **2a** (136 mg, 520 μmol, 1.30 equiv.). The crude material was purified by column chromatography (0 – 5% (v/v) Et_2O in pentane) to give **5la** (2:1 *dr*, 69 mg, 0.15 mmol, 37% yield) as a pale-yellow oil. The product is coeluting in a 2:1 *dr* ratio. The diastereomeric ratio was determined by integration of ^{19}F NMR spectra and were found unchanged before and after purification.

R_f (10% Et_2O/Pentane) = 0.51.

Major diastereoisomer:

^{1}H NMR (400 MHz, $CDCl_3$) δ 7.09 – 6.99 (m, 2H, Ar*H*), 6.85 (d, *J* = 8.9 Hz, 2H, Ar*H*), 5.02 (q, *J* = 5.6 Hz, 1H, CF_3-C*H*), 4.59 – 4.46 (m, 1H, CH_3-C*H*), 3.80 (s, 3H, Ar-CH_3), 1.86 (d, *J* = 1.7 Hz, 3H, C=C-CH_3), 1.53 (d, *J* = 6.7 Hz, 3H, CH_3-CH), 1.09 (s, 21H, Si(C*H*(CH_3)$_2$)$_3$).

^{19}F NMR (376 MHz, $CDCl_3$) δ -75.3 (d, *J* = 5.6 Hz).

Minor diastereoisomer:

^{1}H NMR (400 MHz, $CDCl_3$) δ 7.08 (dd, *J* = 5.6, 3.3 Hz, 2H, Ar*H*), 6.88 – 6.85 (m, 2H, Ar*H*), 4.91 (q, *J* = 6.6 Hz, 1H, CF_3-C*H*), 4.63 – 4.54 (m, 1H, CH_3-C*H*), 3.80 (s, 3H, Ar-CH_3), 1.89 (d, *J* = 1.5 Hz, 3H, C=C-CH_3), 1.52 (d, *J* = 6.2 Hz, 3H, CH_3-CH), 1.09 (s, 21H, Si(C*H*(CH_3)$_2$)$_3$).

^{19}F NMR (376 MHz, $CDCl_3$) δ -75.9 (d, *J* = 6.8 Hz).

Given as a list of peaks due to the heavy overlap of peaks:

^{13}C{^{1}H} NMR (101 MHz, $CDCl_3$) δ 158.4, 146.3, 142.7, 138.8, 136.4, 128.5, 127.9, 122.8, 122.4 (q, *J* = 284.6 Hz), 114.7, 114.6, 114.4, 106.1, 104.0, 103.6, 103.0, 95.9, 95.3, 85.1 (q, *J* = 33.6 Hz), 84.7 (q, *J* = 33.4 Hz), 71.9, 69.1, 55.7, 55.6, 20.9, 20.6, 18.8, 11.55, 11.53.

IR (cm^{-1}) 2944 (w), 2866 (w), 2361 (w), 2262 (w), 2132 (w), 1509 (w), 1246 (w), 910 (s).

HRMS (ESI/QTOF) m/z: $[M + H]^+$ Calcd for $C_{25}H_{37}F_3NO_2Si^+$ 468.2540; Found 468.2550.

3-(4-Methoxyphenyl)-4,6,6-trimethyl-2-(trifluoromethyl)-5-((triisopropylsilyl)ethynyl)-3,6-dihydro-2*H*-1,3-oxazine (5ma)

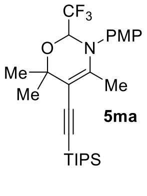

5ma

Prepared according to the general procedure D1 using 2-methylpent-3-yn-ol **1m** (39.3 mg, 400 μmol, 1.00 equiv.) and 1-bromo-2-(triisopropylsilyl)acetylene **2a** (136 mg, 520 μmol, 1.30 equiv.). The crude material was purified by column chromatography (0 – 10 % (v/v) Et_2O in pentane) to give **5ma** (28.9 mg, 0.060 mmol, 15% yield) as a pale-yellow oil.

R_f (10% Et_2O/Pentane) = 0.52.

^{1}H NMR (400 MHz, $CDCl_3$) δ 7.13 – 7.04 (m, 2H, Ar*H*), 6.90 – 6.81 (m, 2H, Ar*H*), 5.00 (q, *J* = 6.0 Hz, 1H, CF_3-C*H*), 3.80 (s, 3H, Ar-O-CH_3), 1.86 (s, 3H, C=C-CH_3), 1.57 (s, 3H, O-C-CH_3), 1.52 (s, 3H, O-C-CH_3), 1.10 (s, 21H, Si(C*H*(CH_3)$_2$)$_3$).

^{13}C{^{1}H} NMR (101 MHz, $CDCl_3$) δ 158.2, 144.3, 136.7, 128.2, 122.5 (q, J_{C-F} = 285.0 Hz), 114.3, 109.7, 104.0, 95.6, 82.7 (q, J_{C-F} = 33.5 Hz), 75.9, 55.5, 28.9, 27.9, 18.9, 18.7, 11.4.

^{19}F NMR (376 MHz, $CDCl_3$) δ -75.0 (d, *J* = 6.0 Hz).

IR (cm^{-1}) 2942 (m), 2865 (m), 2361 (w), 2135 (m), 1626 (m), 1512 (s), 1462 (m), 1383 (m), 1249 (s), 1141 (s).

HRMS (Sicrit plasma/LTQ-Orbitrap) m/z: $[M + H]^+$ Calcd for $C_{26}H_{39}F_3NO_2Si^+$ 482.2697; Found 482.2697.

3-(4-Methoxyphenyl)-4-methyl-2-(trifluoromethyl)-5-(phenylethynyl)-3,6-dihydro-2*H*-1,3-oxazine (5ab)

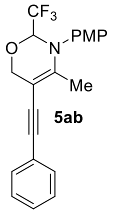

Prepared according to the general procedure D1 using but-2-yn-1-ol **1a** (28 mg, 30 µL, 0.40 mmol, 1.00 equiv.) and (bromoethynyl)benzene **1b** (94.1 mg, 520 µmol, 1.30 equiv.). The crude material was purified by column chromatography (0 – 5 % (v/v) Et_2O in pentane) to give **5ab** (67 mg, 0.17 mmol, 45% yield) as a pale-yellow oil.

R_f (10% Et_2O/Pentane) = 0.55.

^{1}H NMR (400 MHz, $CDCl_3$) δ 7.44 – 7.38 (m, 2H, Ar*H*), 7.33 – 7.26 (m, 3H, Ar*H*), 7.16 – 7.08 (m, 2H, Ar*H*), 6.91 – 6.85 (m, 2H, Ar*H*), 4.97 (q, *J* = 6.5 Hz, 1H, CF_3-C*H*), 4.57 – 4.38 (m, 2H, O-CH_2-C), 3.82 (s, 3H, Ar-O-CH_3), 1.96 (t, *J* = 1.8 Hz, 3H, N-C-CH_3).

^{13}C{^{1}H} NMR (101 MHz, $CDCl_3$) δ 158.3, 142.2, 138.5, 131.0, 128.3, 127.9, 127.7, 123.8, 123.0 (q, J_{C-F} = 288.8 Hz), 114.6, 96.7, 93.4, 85.7, 85.0 (q, J_{C-F} = 33.7 Hz), 63.7, 55.5, 18.7.

^{19}F NMR (376 MHz, $CDCl_3$) δ -75.9 (d, *J* = 6.5 Hz).

IR (cm^{-1}) 2929 (s), 2360 (m), 1636 (s), 1509 (s), 1394 (s), 1296 (s), 1243 (s), 1164 (s).

HRMS (ESI/QTOF) m/z: $[M + H]^+$ Calcd for $C_{21}H_{19}F_3NO_2^+$ 374.1362; Found 374.1362.

3-(4-Methoxyphenyl)-4-methyl-2-(trifluoromethyl)-5-((4-(trifluoromethyl)phenyl)ethynyl)-3,6-dihydro-2*H*-1,3-oxazine (5ac)

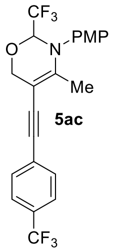

Prepared according to the general procedure D1 using but-2-yn-1-ol **1a** (28 mg, 30 µL, 0.40 mmol, 1.00 equiv.) and 1-(bromoethynyl)-4-(trifluoromethyl)benzene **2c** (129 mg, 520 µmol, 1.30 equiv.). The crude material was purified by column chromatography (0 – 5 % (v/v) Et_2O in pentane) to give **5ac** (62 mg, 0.14 mmol, 35% yield) as a pale-yellow oil.

R_f (10% Et_2O/Pentane) = 0.55.

^{1}H NMR (400 MHz, $CDCl_3$) δ 7.58 – 7.52 (m, 2H, Ar*H*), 7.51 – 7.45 (m, 2H, Ar*H*), 7.16 – 7.10 (m, 2H, Ar*H*), 6.92 – 6.85 (m, 2H, Ar*H*), 4.98 (q, *J* = 6.4 Hz, 1H, CF_3-C*H*), 4.53 (dp, *J* = 14.5, 1.7 Hz, 1H, O-CH_2-C), 4.44 (dp, *J* = 14.6, 1.7 Hz, 1H, O-CH_2-C), 3.82 (s, 3H, Ar-O-CH_3), 1.97 (t, *J* = 1.8 Hz, 3H, N-C-CH_3).

^{13}C{^{1}H} NMR (101 MHz, $CDCl_3$) δ 158.5, 143.5, 138.1, 131.0, 129.1 (q, J_{C-F} = 32.8 Hz), 128.1, 127.7, 125.2 (q, J_{C-F} = 3.8 Hz), 124.0 (q, J_{C-F} = 272.2 Hz), 123.0 (q, J_{C-F} = 288.9 Hz), 114.7, 95.3, 92.3, 88.6, 84.9 (q, J_{C-F} = 33.5 Hz), 63.5, 55.5, 18.8.

^{19}F NMR (376 MHz, $CDCl_3$) δ -62.7, -75.9.

IR (cm^{-1}) 2925 (w), 2190 (w), 1609 (m), 1511 (s), 1397 (m), 1328 (s), 1249 (m), 1165 (s), 1123 (s).

1-(4-((3-(4-Methoxyphenyl)-4-methyl-2-(trifluoromethyl)-3,6-dihydro-2*H*-1,3-oxazin-5-yl)ethynyl)phenyl)ethan-1-one (5ad)

Prepared according to the general procedure D1 using but-2-yn-1-ol **1a** (28 mg, 30 µL, 0.40 mmol, 1.00 equiv.) and 1-(4-(bromoethynyl)phenyl)ethan-1-one **2d** (116 mg, 520 µmol, 1.30 equiv.). The crude material was purified by column chromatography (0 – 20 % (v/v) Et_2O in pentane) to give **5ad** (68 mg, 0.16 mmol, 41% yield) as a pale-yellow oil.

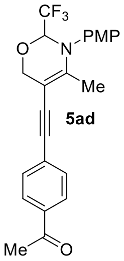

R_f (20% Et_2O/Pentane) = 0.28.

^{1}H NMR (400 MHz, $CDCl_3$) δ 7.81 (d, *J* = 8.4 Hz, 2H, Ar*H*), 7.39 (d, *J* = 8.4 Hz, 2H, Ar*H*), 7.10 – 7.02 (m, 2H, Ar*H*), 6.85 – 6.77 (m, 2H, Ar*H*), 4.91 (q, *J* = 6.4 Hz, 1H, CF_3-C*H*), 4.47 (d, *J* = 14.5 Hz, 1H, O-CH_2-C), 4.37 (d, *J* = 14.5 Hz, 1H, O-CH_2-C), 3.74 (s, 3H, Ar-O-CH_3), 2.52 (s, 3H, Ar-CO-CH_3), 1.90 (s, 3H, N-C-CH_3).

^{13}C{^{1}H} NMR (101 MHz, $CDCl_3$) δ 197.4, 158.7, 143.8, 138.2, 135.6, 131.0, 129.0, 128.4, 128.3, 123.1 (q, J_{C-F} = 289.2 Hz), 114.8, 95.4, 93.2, 89.9, 85.0 (q, J_{C-F} = 33.5 Hz), 63.7, 55.7, 26.7, 18.9.

^{19}F NMR (376 MHz, $CDCl_3$) δ -75.8 (d, *J* = 6.3 Hz).

IR (cm^{-1}) 2964 (w), 2869 (w), 2362 (w), 2186 (m), 1681 (m), 1594 (m), 1509 (s), 1246 (s), 1160 (s), 1112 (s).

HRMS (ESI/QTOF) m/z: $[M + H]^+$ Calcd for $C_{23}H_{21}F_3NO_3^+$ 416.1468; Found 416.1469.

3-(4-Methoxyphenyl)-4-methyl-2-(trifluoromethyl)-5-(3,3,3-trifluoroprop-1-en-2-yl)-3,6-dihydro-2*H*-1,3-oxazine (5ae)

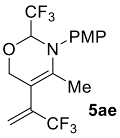

Prepared according to the general procedure D1 using but-2-yn-1-ol **1a** (28 mg, 30 μL, 0.40 mmol, 1.00 equiv.) and 2-bromo-3,3,3-trifluoro-1-propene **2e** (91.0 mg, 520 μmol, 1.30 equiv.). The crude material was purified by column chromatography (0 – 5 % (v/v) Et_2O in pentane) to give **5ae** (32 mg, 0.090 mmol, 22% yield) as a pale-yellow oil.

R_f (5% Et_2O/Pentane) = 0.53.

^{1}H NMR (400 MHz, $CDCl_3$) δ 7.14 – 7.05 (m, 2H, Ar*H*), 6.92 – 6.83 (m, 2H, Ar*H*), 6.00 (q, *J* =1.5 Hz, 1H, CF_3-C=CH_2), 5.51 (q, *J* = 1.4 Hz, 1H, CF_3-C=CH_2), 4.94 (q, *J* = 6.6 Hz, 1H, CF_3-C*H*), 4.40 (dt, *J* = 15.5, 1.8 Hz, 1H, O-CH_2-C), 4.36 – 4.22 (m, 1H, O-CH_2-C), 3.81 (s, 3H, Ar-O-CH_3), 1.67 (t, *J* = 1.9 Hz, 3H, N-C-CH_3).

$^{13}C\{^{1}H\}$ NMR (101 MHz, $CDCl_3$) δ 158.0, 139.1, 135.8, 135.3 (q, J_{C-F} = 31.4 Hz), 127.1, 124.3 (q, J_{C-F} = 5.2 Hz), 123.06 (q, J_{C-F} = 288.2 Hz), 123.05 (q, J_{C-F} = 274.7 Hz), 114.6, 110.3, 85.1 (q, J_{C-F} = 33.6 Hz), 64.3, 55.5, 17.2.

^{19}F NMR (376 MHz, $CDCl_3$) δ -66.7, -76.2 (d, *J* = 6.8 Hz).

IR (cm^{-1}) 2953 (w), 1675 (w), 1510 (s), 1391 (m), 1293 (m), 1246 (s), 1166 (s), 1116 (s), 1060 (m).

HRMS (ESI/QTOF) m/z: $[M + H]^+$ Calcd for $C_{16}H_{16}F_6NO_2^+$ 368.1080; Found 368.1070.

3-(4-Methoxyphenyl)-4-methyl-5-(p-tolyl)-2-(trifluoromethyl)-3,6-dihydro-2*H*-1,3-oxazine (5af)

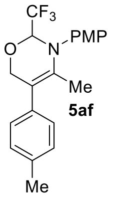

Prepared according to the general procedure D1 using but-2-yn-1-ol (28 mg, 30 μL, 0.40 mmol, 1.00 equiv.) and 4-iodotoluene (113 mg, 520 μmol, 1.30 equiv.). The crude material was purified by column chromatography (0 – 5 % (v/v) Et_2O in pentane) to give **5af** (107 mg, 0.290 mmol, 74% yield) as a pale-yellow amorphous solid.

3 mmol scale:

Prepared according to the general procedure D1 using but-2-yn-1-ol (210 mg, 224 μL, 3.00 mmol, 1.00 equiv.) and 4-iodotoluene (850 mg, 3.90 mmol, 1.30 equiv.). The crude material was purified by column chromatography (0 – 5 % (v/v) Et_2O in pentane) to give **5af** (759 mg, 2.09 mmol, 70% yield) as a pale-yellow amorphous solid.

R_f (10% Et_2O/Pentane) = 0.47.

^{1}H NMR (400 MHz, $CDCl_3$) 7.20 – 7.10 (m, 6H, Ar*H*), 6.95 – 6.82 (m, 2H, Ar*H*), 5.01 (q, *J* = 6.6 Hz, 1H, CF_3-C*H*), 4.60 – 4.47 (m, 1H, O-CH_2-C), 4.50 – 4.37 (m, 1H, O-CH_2-C), 3.81 (s, 3H, Ar-O-CH_3), 2.36 (s, 3H, Ar-CH_3), 1.63 (t, *J* = 1.9 Hz, 3H, N-C-CH_3).

$^{13}C\{^{1}H\}$ NMR (101 MHz, $CDCl_3$) δ 157.8, 140.1, 137.0, 135.1, 131.7, 129.3, 129.2, 126.9, 123.5 (q, J_{C-F} = 288.2 Hz), 118.8, 114.7, 85.6 (q, J_{C-F} = 33.3 Hz), 65.7, 55.8, 21.4, 17.3.

^{19}F NMR (376 MHz, $CDCl_3$) δ -75.9 (d, *J* = 6.8 Hz).

IR (cm^{-1}) 2967 (m), 1671 (w), 1509 (s), 1390 (m), 1249 (s), 1173 (s), 1118 (s).

HRMS (ESI/QTOF) m/z: $[M + H]^+$ Calcd for $C_{20}H_{21}F_3NO_2^+$ 364.1519; Found 364.1508.

3-(4-Methoxyphenyl)-4-methyl-2-(trifluoromethyl)-5-(4-methoxyphenyl)-3,6-dihydro-2*H*-1,3-oxazine (5ag)

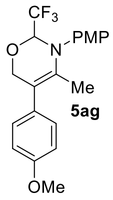

Prepared according to the general procedure D1 using but-2-yn-1-ol **1a** (28 mg, 30 μL, 0.40 mmol, 1.00 equiv.) and 4-iodoanisole **2g** (122 mg, 520 μmol, 1.30 equiv.). The crude material was purified by column chromatography (0 – 5 % (v/v) Et_2O in pentane) to give **5ag** (90 mg, 0.24 mmol, 59% yield) as a pale-yellow amorphous solid.

R_f (5% Et_2O/Pentane) = 0.30.

^{1}H NMR (400 MHz, $CDCl_3$) δ 7.17 – 7.11 (m, 4H, Ar*H*), 6.92 – 6.85 (m, 4H, Ar*H*), 5.00 (q, *J* = 6.7 Hz, 1H, CF_3-C*H*), 4.56 – 4.46 (m, 1H, O-CH_2-C), 4.42 (dt, *J* = 15.5, 1.6 Hz, 1H, O-CH_2-C), 3.82 (s, 3H, Ar-O-CH_3), 3.81 (s, 3H, Ar-O-CH_3), 1.62 (t, *J* = 1.9 Hz, 3H, N-C-CH_3).

$^{13}C\{^{1}H\}$ NMR (101 MHz, $CDCl_3$) δ 158.8, 157.7, 140.1, 131.6, 130.30, 130.26, 126.7, 123.4 (q, J_{C-F} = 288.1 Hz), 118.5, 114.6, 114.0, 85.5 (q, J_{C-F} = 33.4 Hz), 65.7, 55.7, 55.4, 17.2.

^{19}F NMR (376 MHz, $CDCl_3$) δ -75.91 (d, *J* = 6.9 Hz).

IR (cm^{-1}) 2950 (w), 2845 (w), 1678 (w), 1617 (w), 1512 (s), 1465 (m), 1389 (m), 1281 (m), 1244 (s), 1157 (s), 1109 (m).

HRMS (ESI/QTOF) m/z: $[M + H]^+$ Calcd for $C_{20}H_{21}F_3NO_3^+$ 380.1468; Found 380.1468.

3-(4-Methoxyphenyl)-4-methyl-2-(trifluoromethyl)-5-(4-trifluoromethylphenyl)-3,6-dihydro-2*H*-1,3-oxazine (5ah)

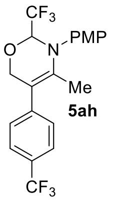

5ah

Prepared according to the general procedure D1 using but-2-yn-1-ol **1a** (28 mg, 30 µL, 0.40 mmol, 1.00 equiv.) and 1-iodo-4-(trifluoromethyl)benzene **2h** (141 mg, 520 µmol, 1.30 equiv.). The crude material was purified by column chromatography (0 – 5% (v/v) Et_2O in pentane) to give **5ah** (106 mg, 0.254 mmol, 63% yield) as a pale-yellow oil.

$\underline{R_f}$ (5% Et_2O/Pentane) = 0.43.

$\underline{^1H\ NMR}$ (400 MHz, $CDCl_3$) δ 7.65 – 7.58 (m, 2H, Ar*H*), 7.38 – 7.30 (m, 2H, Ar*H*), 7.17 – 7.11 (m, 2H, Ar*H*), 6.93 – 6.84 (m, 2H, Ar*H*), 5.02 (q, *J* = 6.6 Hz, 1H, CF_3-C*H*), 4.57 (dp, *J* = 15.4, 1.7 Hz, 1H, O-CH_2-C), 4.45 (dq, *J* = 13.8, 1.6 Hz, 1H, O-CH_2-C), 3.81 (s, 3H, Ar-O-CH_3), 1.63 (t, *J* = 1.9 Hz, 3H, N-C-CH_3).

$\underline{^{13}C\{^1H\}\ NMR}$ (101 MHz, $CDCl_3$) δ 157.9, 141.8, 139.4, 133.0, 129.5, 129.2 (q, $J_{C\text{-}F}$ = 32.5 Hz), 128.64 (q, $J_{C\text{-}F}$ = 81.6 Hz), 127.0, 125.39 (q, $J_{C\text{-}F}$ = 3.8 Hz), 124.1 (q, $J_{C\text{-}F}$ = 265.4 Hz), 123.2 (q, $J_{C\text{-}F}$ = 288.3 Hz) 114.6, 85.3 (q, $J_{C\text{-}F}$ = 33.3 Hz), 65.0, 55.5, 17.1.

$\underline{^{19}F\ NMR}$ (376 MHz, $CDCl_3$) -62.5, -76.0 (d, *J* = 6.5 Hz).

<u>IR</u> (cm^{-1}) 2937 (w), 1674 (w), 1617 (w), 1510 (s), 1386 (w), 1328 (s), 1247 (s), 1170 (s), 1121 (s), 1069 (m).

<u>HRMS</u> (ESI/QTOF) m/z: $[M + H]^+$ Calcd for $C_{20}H_{18}F_6NO_2^+$ 418.1236; Found 418.1231.

5-(4-Chlorophenyl)-3-(4-methoxyphenyl)-4-methyl-2-(trifluoromethyl)-3,6-dihydro-2*H*-1,3-oxazine (5ai)

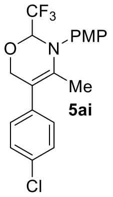

5ai

Prepared according to the general procedure D1 using but-2-yn-1-ol **1a** (28 mg, 30 µL, 0.40 mmol, 1.00 equiv.) and 1-chloro-4-iodobenzene **2i** (124 mg, 520 µmol, 1.30 equiv.). The crude material was purified by column chromatography (0 – 5 % (v/v) Et_2O in pentane) to give **5ai** (107 mg, 0.290 mmol, 74% yield) as a pale-yellow amorphous solid.

$\underline{R_f}$ (10% Et_2O/Pentane) = 0.47.

$\underline{^1H\ NMR}$ (400 MHz, $CDCl_3$) δ 7.36 – 7.29 (m, 2H, Ar*H*), 7.19 – 7.10 (m, 4H, Ar*H*), 6.91 – 6.83 (m, 2H, Ar*H*), 5.00 (q, *J* = 6.6 Hz, 1H, CF_3-C*H*), 4.58 – 4.47 (m, 1H, O-CH_2-C), 4.47 – 4.38 (m, 1H, O-CH_2-C), 3.81 (s, 3H, Ar-O-CH_3), 1.61 (t, *J* = 1.9 Hz, 3H, N-C-CH_3).

$\underline{^{13}C\{^1H\}\ NMR}$ (101 MHz, $CDCl_3$) δ 157.9, 139.7, 136.5, 133.1, 132.5, 130.6, 128.8, 127.0, 123.4 (q, $J_{C\text{-}F}$ = 288.3 Hz), 117.2, 114.6, 85.4 (q, $J_{C\text{-}F}$ = 33.4 Hz), 65.3, 55.7, 17.2.

$\underline{^{19}F\ NMR}$ (376 MHz, $CDCl_3$) δ -75.9 (d, *J* = 6.7 Hz).

<u>IR</u> (cm^{-1}) 2923 (m), 1671 (w), 1512 (s), 1386 (m), 1292 (m), 1242 (s), 1173 (s), 1116 (s), 1037 (m).

<u>HRMS</u> (ESI/QTOF) m/z: $[M + H]^+$ Calcd for $C_{19}H_{18}ClF_3NO_2^+$ 384.0973; Found 384.0970.

5-(4-Fluorophenyl)-3-(4-methoxyphenyl)-4-methyl-2-(trifluoromethyl)-3,6-dihydro-2*H*-1,3-oxazine (5aj)

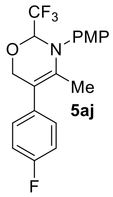

5aj

Prepared according to the general procedure D1 using but-2-yn-1-ol **1a** (28 mg, 30 µL, 0.40 mmol, 1.00 equiv.) and 4-fluoroiodobenzene **2j** (115 mg, 520 µmol, 1.30 equiv.). The crude material was purified by column chromatography (0 – 5 % (v/v) Et_2O in pentane) to give **5aj** (96 mg, 0.26 mmol, 65% yield) as a pale-yellow amorphous solid.

$\underline{R_f}$ (5% Et_2O/Pentane) = 0.43.

$\underline{^1H\ NMR}$ (400 MHz, $CDCl_3$) δ 7.22 – 7.16 (m, 2H, Ar*H*), 7.16 – 7.10 (m, 2H, Ar*H*), 7.08 – 7.01 (m, 2H, Ar*H*), 6.91 – 6.85 (m, 2H, Ar*H*), 5.00 (q, *J* = 6.7 Hz, 1H, CF_3-C*H*), 4.50 (dp, *J* = 15.3, 1.7 Hz, 1H, O-CH_2-C), 4.42 (dp, *J* = 15.5, 1.5 Hz, 1H, O-CH_2-C), 3.81 (s, 3H, Ar-O-CH_3), 1.60 (t, *J* = 2.0 Hz, 3H, N-C-CH_3).

$\underline{^{13}C\{^1H\}\ NMR}$ (101 MHz, $CDCl_3$) δ 162.1 (d, $J_{C\text{-}F}$ = 246.1 Hz), 157.8, 139.8, 133.9 (d, $J_{C\text{-}F}$ = 3.4 Hz), 132.2, 130.9 (d, $J_{C\text{-}F}$ = 8.0 Hz), 126.9, 123.4 (q, $J_{C\text{-}F}$ = 288.5 Hz), 117.6, 115.5 (d, $J_{C\text{-}F}$ = 21.3 Hz), 114.6, 85.5 (q, $J_{C\text{-}F}$ = 33.4 Hz), 65.5, 55.7, 17.2.

$\underline{^{19}F\ NMR}$ (376 MHz, $CDCl_3$) δ -75.9 (d, *J* = 6.9 Hz), -115.2.

<u>IR</u> (cm^{-1}) 2919 (w), 1672 (w), 1512 (s), 1393 (w), 1247 (s), 1169 (m), 1119 (s), 1037 (m).

<u>HRMS</u> (ESI/QTOF) m/z: $[M + H]^+$ Calcd for $C_{19}H_{18}F_4NO_2^+$ 368.1268; Found 368.1266.

5-(3-Fluorophenyl)-3-(4-methoxyphenyl)-4-methyl-2-(trifluoromethyl)-3,6-dihydro-2*H*-1,3-oxazine (5ak)

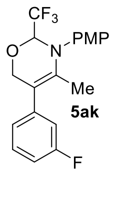

5ak

Prepared according to the general procedure D1 using but-2-yn-1-ol **1a** (28 mg, 30 µL, 0.40 mmol, 1.00 equiv.) and 3-fluoroiodobenzene **2k** (115 mg, 520 µmol, 1.30 equiv.). The crude material was purified by column chromatography (0 – 5 % (v/v) Et_2O in pentane) to give **5ak** (94 mg, 0.26 mmol, 64% yield) as a pale-yellow amorphous solid.

$\underline{R_f}$ (10% Et_2O/Pentane) = 0.66.

^{1}H NMR (400 MHz, $CDCl_3$) δ 7.32 (td, *J* = 7.9, 6.1 Hz, 1H, Ar*H*), 7.18 – 7.09 (m, 2H, Ar*H*), 7.03 – 6.91 (m, 3H, Ar*H*), 6.91 – 6.84 (m, 2H, Ar*H*), 5.00 (q, *J* = 6.6 Hz, 1H, CF_3-C*H*), 4.53 (dt, *J* = 15.4, 1.6 Hz, 1H, O-CH_2-C), 4.44 (d, *J* = 15.4 Hz, 1H, O-CH_2-C), 3.81 (s, 3H, Ar-O-CH_3), 1.64 (t, *J* = 1.9 Hz, 3H, N-C-CH_3).

^{13}C{^{1}H} NMR (101 MHz, $CDCl_3$) δ 162.9 (d, J_{C-F} = 246.5 Hz), 157.9, 140.3 (d, J_{C-F} = 7.8 Hz), 139.7, 132.3, 130.1 (d, J_{C-F} = 8.5 Hz), 127.0, 125.0 (d, J_{C-F} = 2.8 Hz), 123.3 (q, J_{C-F} = 288.4 Hz), 117.3, 116.2 (d, J_{C-F} = 20.9 Hz), 114.7, 114.1 (d, J_{C-F} = 21.0 Hz), 85.4 (q, J_{C-F} = 33.3 Hz), 65.3, 55.7, 17.3.

^{19}F NMR (376 MHz, $CDCl_3$) δ -76.0 (d, *J* = 6.7 Hz), -113.13(td, *J* = 9.1, 6.1 Hz).

IR (cm^{-1}) 2960 (w), 2845 (w), 2359 (m), 2339 (w), 1674 (w), 1581 (m), 1510 (s), 1292 (s), 1245 (s), 1177 (s), 1119 (s).

HRMS (ESI/QTOF) m/z: [M + H]$^+$ Calcd for $C_{19}H_{18}F_4NO_2^+$ 368.1268; Found 368.1265.

5-(2-Fluorophenyl)-3-(4-methoxyphenyl)-4-methyl-2-(trifluoromethyl)-3,6-dihydro-2*H*-1,3-oxazine (5al)

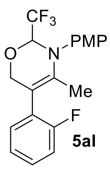

Prepared according to the general procedure D1 using but-2-yn-1-ol **1a** (28 mg, 30 µL, 0.40 mmol, 1.00 equiv.) and 2-fluoroiodobenzene **2l** (115 mg, 520 µmol, 1.30 equiv.). The crude material was purified by column chromatography (0 – 5 % (v/v) Et_2O in pentane) to give **5al** (64 mg, 0.17 mmol, 43% yield) as a pale-yellow amorphous solid.

R_f (10% Et_2O/Pentane) = 0.66.

^{1}H NMR (400 MHz, $CDCl_3$) δ 7.33 – 7.23 (m, 1H, Ar*H*), 7.24 – 7.05 (m, 5H, Ar*H*), 6.93 – 6.84 (m, 2H, Ar*H*), 5.02 (q, *J* = 6.6 Hz, 1H, CF_3-C*H*), 4.52 (dd, *J* = 15.3, 1.6 Hz, 1H, O-CH_2-C), 4.43 (d, *J* = 15.3 Hz, 1H, O-CH_2-C), 3.81 (s, 3H, Ar-O-CH_3), 1.57 (q, *J* = 1.2 Hz, 3H, N-C-CH_3).

^{13}C{^{1}H} NMR (101 MHz, $CDCl_3$) δ 160.6 (d, J_{C-F} = 246.1 Hz), 157.8, 139.8, 133.7, 131.8 (d, J_{C-F} = 3.7 Hz), 129.3 (d, J_{C-F} = 8.1 Hz), 127.0, 125.2 (d, J_{C-F} = 16.6 Hz), 124.3 (d, J_{C-F} = 3.6 Hz), 123.4 (q, J_{C-F} = 288.0 Hz), 116.0 (d, J_{C-F} = 22.4 Hz), 114.6, 112.4, 85.5 (q, J_{C-F} = 33.3 Hz), 64.7, 55.7, 17.3.

^{19}F NMR (376 MHz, $CDCl_3$) δ -74.0 – -78.6 (m), -114.7.

IR (cm^{-1}) 2964 (w), 2844 (w), 2350 (w), 2247 (w), 1675 (w), 1509 (s), 1390 (m), 1292 (s), 1245 (s), 1173 (s), 1116 (s).

HRMS (ESI/QTOF) m/z: [M + H]$^+$ Calcd for $C_{19}H_{18}F_4NO_2^+$ 368.1268; Found 368.1267.

5-(2-Fluoropyridin-4-yl)-3-(4-methoxyphenyl)-4-methyl-2-(trifluoromethyl)-3,6-dihydro-2*H*-1,3-oxazine (5am)

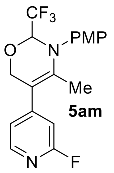

Prepared according to the general procedure D1 using but-2-yn-1-ol **1a** (28 mg, 30 µL, 0.40 mmol, 1.00 equiv.) and 2-fluoro-4-iodopyridine **2m** (116 mg, 520 µmol, 1.30 equiv.). The crude material was purified by column chromatography (0 – 30 % (v/v) Et_2O in pentane) to give **5am** (44.2 mg, 0.120 mmol, 30% yield) as a pale-yellow oil. The product is coeluting with a with an isomeric product in a 9:1 ratio determined by integration of the ^{19}F NMR spectra. The ratio was found to be unchanged before and after the purification.

Major diastereomer:

R_f (10% Et_2O/Pentane) = 0.21.

^{1}H NMR (400 MHz, $CDCl_3$) δ 8.17 (d, *J* = 5.2 Hz, 1H, Ar*H*), 7.18 – 7.09 (m, 2H, Ar*H*), 7.02 (dt, *J* = 5.2, 1.7 Hz, 1H, Ar*H*), 6.94 – 6.84 (m, 2H, Ar*H*), 6.77 (s, 1H, Ar*H*), 5.01 (q, *J* = 6.5 Hz, 1H, CF_3-C*H*), 4.59 (dt, *J* = 15.1, 1.6 Hz, 1H, O-CH_2-C), 4.46 (d, *J* = 15.1 Hz, 1H, O-CH_2-C), 3.81 (s, 3H, Ar-O-CH_3), 1.72 (t, *J* = 1.8 Hz, 3H, N-C-CH_3).

^{13}C{^{1}H} NMR (101 MHz, $CDCl_3$) δ 164.2 (d, J_{C-F} = 239.0 Hz), 158.4, 151.8 (d, J_{C-F} = 8.2 Hz), 147.7 (d, J_{C-F} = 15.7 Hz), 138.8, 135.2, 127.6, 123.2 (d, J_{C-F} = 288.6 Hz), 121.7 (d, J_{C-F} = 4.0 Hz), 114.8, 113.3 (d, J_{C-F} = 3.2 Hz), 109.4 (d, J_{C-F} = 36.9 Hz), 85.2 (q, J_{C-F} = 33.5 Hz), 64.3, 55.7, 17.5.

^{19}F NMR (376 MHz, $CDCl_3$) δ -68.0, -76.0 (d, *J* = 6.4 Hz).

IR (cm^{-1}) 2925 (w), 2362 (w), 2336 (w), 1685 (w), 1609 (m), 1551 (w), 1404 (m), 1296 (m), 1184 (s).

HRMS (ESI/QTOF) m/z: [M + H]$^+$ Calcd for $C_{18}H_{17}F_4N_2O_2^+$ 369.1221; Found 369.1227.

3,4-Bis(4-methoxyphenyl)-5-(*p*-tolyl)-2-(trifluoromethyl)-3,6-dihydro-2*H*-1,3-oxazine (5cf)

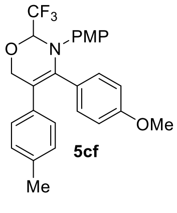

Prepared according to the general procedure D1 using 3-(4-methoxyphenyl)prop-2-yn-1-ol **1c** (70 mg, 0.43 mmol, 1.0 equiv.) and 4-iodotoluene **2f** (113 mg, 520 µmol, 1.30 equiv.). The crude material was purified by column chromatography (0 – 5 % (v/v) Et_2O in pentane) to give **5cf** (61.4 mg, 0.135 mmol, 31% yield) as a pale-yellow oil.

R_f (10% Et_2O/Pentane) = 0.54.

^{1}H NMR (400 MHz, $CDCl_3$) δ 7.06 (d, *J* = 8.6 Hz, 2H, Ar*H*), 7.00 (d, *J* = 8.3 Hz, 4H, Ar*H*), 6.93 (d, *J* = 7.9 Hz, 2H, Ar*H*), 6.69 (d, *J* = 8.8 Hz, 2H, Ar*H*), 6.55 (d, *J* = 8.6 Hz, 2H, Ar*H*), 5.27 (q, *J*

= 6.4 Hz, 1H, CF_3-C*H*), 4.88 (d, *J* = 15.6 Hz, 1H, O-C*H*$_2$-C), 4.39 (d, *J* = 15.6 Hz, 1H, O-C*H*$_2$-C), 3.69 (s, 3H, Ar-O-C*H*$_3$), 3.66 (s, 3H, Ar-O-C*H*$_3$), 2.29 (s, 3H, Ar-C*H*$_3$).

^{13}C{^{1}H} NMR (101 MHz, $CDCl_3$) δ 158.8, 156.7, 140.4, 136.4, 135.6, 135.1, 131.6, 129.2, 129.1, 128.1, 125.7, 123.4 (q, $J_{C\text{-}F}$ = 288.2 Hz), 120.7, 114.3, 113.2, 85.8 (q, $J_{C\text{-}F}$ = 33.5 Hz), 65.7, 55.4, 55.1, 21.3.

^{19}F NMR (376 MHz, $CDCl_3$) δ -75.5 (d, *J* = 6.5 Hz).

IR (cm^{-1}) 2912 (w), 2359 (w), 2258 (w), 1608 (w), 1509 (s), 1245 (s), 1140 (s).

HRMS (ESI/QTOF) m/z: $[M + H]^+$ Calcd for $C_{26}H_{25}F_3NO_3^+$ 456.1781; Found 456.1781.

D.3. General Procedure D3 for the 5-*exo-dig* carboamination reaction

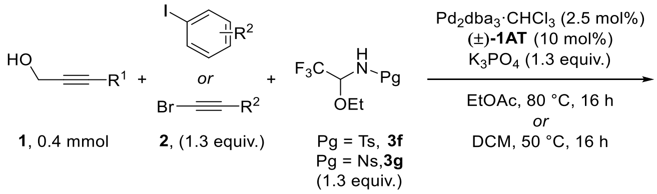

An oven-dried 8 mL microwave tube equipped with a Teflon coated stirring bar was charged with Pd_2dba_3·$CHCl_3$ (10.4 mg, 10.0 μmol, 2.5 mol%), **(±)-L1** (20.3 mg, 40.0 μmol, 10 mol%) and K_3PO_4 (110 mg, 40.0 μmol, 1.3 equiv.). The tube was evacuated and back-filled with N_2 three times. Then, DCM (2.0 mL, alkynylation) or EtOAc (2.0 mL, arylation) was added and the mixture was stirred at 50 °C for 15 minutes. Afterwards, the corresponding propargyl alcohol (0.400 mmol), tosylamine **3f** (167 mg, 0.560 mmol, 1.4 equiv.) and the corresponding bromoalkyne or aryl iodide (0.520 mmol) were added. The resulting solution was then stirred at 50 °C for 16 h for alkynylation and at 80 °C for 16 h for arylation. Next, the reaction mixture was allowed to cool down to room temperature and filtered through a plug of silica gel eluting with DCM in pentane (25 mL) and the mixture was concentrated in vacuo. The crude material was purified by flash column chromatography on silica gel using a Biotage flash chromatography machine to afford the corresponding product.

D.4. Characterization of the 5-*exo-dig* carboamination products

(*E*)-3-Tosyl-2-(trifluoromethyl)-4-(4-(triisopropylsilyl)but-3-yn-2-ylidene)oxazolidine (4aaf)

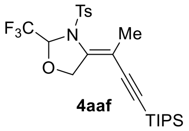

4aaf

Prepared according to the general procedure D3 using but-2-yn-1-ol **1a** (28 mg, 30 μL, 0.40 mmol, 1.00 equiv.) and 1-bromo-2-(triisopropylsilyl)acetylene **2a** (136 mg, 520 μmol, 1.30 equiv.). The crude material was purified by column chromatography (0 – 10 % (v/v) Et_2O in pentane) to give **4aaf** (157.1 mg, 0.313 mmol, 78% yield) as a pale-yellow amorphous solid.

R_f (5% Et_2O/Pentane) = 0.47.

^{1}H NMR (400 MHz, $CDCl_3$) δ 7.74 – 7.66 (m, 2H, Ar*H*), 7.36 – 7.29 (m, 2H, Ar*H*), 5.71 (q, *J* = 4.9 Hz, 1H, CF_3-C*H*), 4.43 (dq, *J* = 12.4, 1.6 Hz, 1H, O-C*H*$_2$-C), 3.53 – 3.44 (m, 1H, O-C*H*$_2$-C), 2.45 (s, 3H, Ar-C*H*$_3$), 2.16 (dd, *J* = 2.2, 1.6 Hz, 3H, N-C-C*H*$_3$), 1.05 (s, 21H, Si(C*H(*C*H*$_3$)$_2$)$_3$).

^{13}C{^{1}H} NMR (101 MHz, $CDCl_3$) δ 145.8, 138.4, 133.6, 130.1, 128.2, 122.2 (q, $J_{C\text{-}F}$ = 285.0 Hz), 114.0, 104.7, 98.9, 89.1 (q, $J_{C\text{-}F}$ = 35.8 Hz), 70.0, 21.9, 20.0, 18.7, 11.2.

^{19}F NMR (376 MHz, $CDCl_3$) δ -80.5 (d, *J* = 4.9 Hz).

IR (cm^{-1}) 2943 (m), 2867 (m), 2142 (w), 1465 (w), 1375 (m), 1188 (s), 1175 (s), 1152 (s).

HRMS (Nanochip-based ESI/LTQ-Orbitrap) m/z: $[M + H]^+$ Calcd for $C_{24}H_{35}F_3NO_3SSi^+$ 502.2054; Found 502.2056.

(*E*)-3-((4-Nitrophenyl)sulfonyl)-2-(trifluoromethyl)-4-(4-(triisopropylsilyl)but-3-yn-2-ylidene)oxazolidine (4aag)

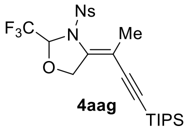

4aag

Prepared according to the general procedure D3 using but-2-yn-1-ol **1a** (28 mg, 30 μL, 0.40 mmol, 1.00 equiv.), 1-bromo-2-(triisopropylsilyl)acetylene **2a** (136 mg, 520 μmol, 1.30 equiv.) and **3g** (184 mg, 560 μmol, 1.40 equiv.). The crude material was purified by column chromatography (0 – 10 % (v/v) Et_2O in pentane) to give **4aag** (112.8 mg, 0.212 mmol, 53% yield) as a pale-yellow amorphous solid.

R_f (5% Et_2O/Pentane) = 0.33.

^{1}H NMR (400 MHz, $CDCl_3$) δ 8.43 – 8.35 (m, 2H, Ar*H*), 8.07 – 7.99 (m, 2H, Ar*H*), 5.73 (q, *J* = 4.9 Hz, 1H, CF_3-C*H*), 4.49 (dq, *J* = 12.8, 1.7 Hz, 1H, O-C*H*$_2$-C), 3.57 (dd, *J* = 12.8, 2.5 Hz, 1H, O-C*H*$_2$-C), 2.22 – 2.15 (m, 3H, N-C-C*H*$_3$), 1.05 (d, *J* = 2.1 Hz, 21H, Si(C*H(*C*H*$_3$)$_2$)$_3$).

^{13}C{^{1}H} NMR (101 MHz, $CDCl_3$) δ 151.2, 142.2, 137.0, 129.5, 124.6, 121.9 (q, $J_{C\text{-}F}$ = 285.4 Hz), 115.2, 103.9, 100.4, 89.0 (q, $J_{C\text{-}F}$ = 36.2 Hz), 70.0, 20.3, 18.7, 11.1.

^{19}F NMR (376 MHz, $CDCl_3$) δ -80.2 (d, *J* = 4.8 Hz).

IR (cm^{-1}) 2946 (m), 2867 (m), 2143 (w), 1537 (m), 1382 (m), 1349 (m), 1188 (s), 1152 (s).

HRMS (APCI/QTOF) m/z: $[M + H]^+$ Calcd for $C_{23}H_{32}F_3N_2O_5SSi^+$ 533.1748; Found 533.1752.

(*E*)-4-(1-Phenyl-3-(triisopropylsilyl)prop-2-yn-1-ylidene)-3-tosyl-2-(trifluoromethyl)oxazolidine (4baf)

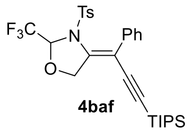

4baf

Prepared according to the general procedure D3 using 3-phenylprop-2-yn-1-ol **1b** (53 mg, 50 µL, 0.40 mmol, 1.00 equiv.) and 1-bromo-2-(triisopropylsilyl)acetylene **2a** (136 mg, 520 µmol, 1.30 equiv.). The crude material was purified by column chromatography (0 – 10 % (v/v) Et_2O in pentane) to give **4baf** (223 mg, 0.395 mmol, 99% yield) as a pale-yellow amorphous solid.

R_f (10% Et_2O/Pentane) = 0.52.

^{1}H NMR (400 MHz, $CDCl_3$) δ 7.69 (d, *J* = 6.9 Hz, 2H, Ar*H*), 7.56 (d, *J* = 8.3 Hz, 2H, Ar*H*), 7.39 – 7.23 (m, 5H, Ar*H*), 5.91 (q, *J* = 5.0 Hz, 1H, CF_3-C*H*), 4.64 (d, *J* = 13.3 Hz, 1H, O-CH_2-C), 3.85 (d, *J* = 13.4 Hz, 1H, O-CH_2-C), 2.44 (s, 3H, Ar-CH_3), 1.09 (s, 21H, Si(C*H*(CH_3)$_2$)$_3$).

^{13}C{^{1}H} NMR (101 MHz, $CDCl_3$) δ 145.5, 138.4, 135.1, 133.6, 129.8, 128.4, 128.3, 128.2, 128.0, 122.3 (q, $J_{C\text{-}F}$ = 285.5 Hz), 115.7, 103.4, 100.4, 89.4 (q, $J_{C\text{-}F}$ = 35.7 Hz), 71.0, 21.7, 18.6, 11.1.

^{19}F NMR (376 MHz, $CDCl_3$) δ -80.1.

IR (cm^{-1}) 2944 (m), 2867 (m), 2359 (w), 2143 (w), 1379 (m), 1173 (s), 1154 (s).

HRMS (ESI/QTOF) m/z: $[M + Na]^+$ Calcd for $C_{29}H_{36}F_3NNaO_3SSi^+$ 586.2029; Found 586.2034.

(*E*)-4-(1-(4-Methoxyphenyl)-3-(triisopropylsilyl)prop-2-yn-1-ylidene)-3-tosyl-2-(trifluoromethyl)oxazolidine (4caf)

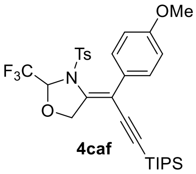

4caf

Prepared according to the general procedure D3 using 3-(4-methoxyphenyl)prop-2-yn-1-ol **1c** (65 mg, 0.40 mmol, 1.00 equiv.) and 1-bromo-2-(triisopropylsilyl)acetylene **2a** (136 mg, 520 µmol, 1.30 equiv.). The crude material was purified by column chromatography (0 – 10 % (v/v) Et_2O in pentane) to give **4caf** (188 mg, 0.316 mmol, 85% yield) as a pale-yellow amorphous solid.

R_f (20% Et_2O/Pentane) = 0.63.

^{1}H NMR (400 MHz, $CDCl_3$) δ 7.70 – 7.62 (m, 2H, Ar*H*), 7.61 – 7.52 (m, 2H, Ar*H*), 7.26 (d, *J* = 8.0 Hz, 2H, Ar*H*), 6.90 – 6.81 (m, 2H, Ar*H*), 5.90 (q, *J* = 5.0 Hz, 1H, CF_3-C*H*), 4.61 (d, *J* = 13.2 Hz, 1H, O-CH_2-C), 3.83 (s, 3H, Ar-O-CH_3), 3.80 (d, *J* = 13.2 Hz, 1H, O-CH_2-C), 2.43 (s, 3H, Ar- CH_3), 1.08 (s, 21H, Si(C*H*(CH_3)$_2$)$_3$).

^{13}C{^{1}H} NMR (101 MHz, $CDCl_3$) δ 159.8, 145.6, 136.9, 133.8, 129.9, 129.9, 128.2, 127.5, 122.4 (q, $J_{C\text{-}F}$ = 285.4 Hz), 115.6, 113.7, 103.7, 100.2, 89.6 (q, $J_{C\text{-}F}$ = 35.7 Hz), 71.1, 55.3, 21.8, 18.8, 11.3.

^{19}F NMR (376 MHz, $CDCl_3$) δ -80.0 (d, *J* = 5.0 Hz).

IR (cm^{-1}) 2946 (w), 2866 (w), 2356 (w), 2256 (w), 2139 (w), 1511 (w), 1377 (w), 1249 (m), 1177 (m), 1152 (m), 731 (s).

HRMS (ESI/QTOF) m/z: $[M + Na]^+$ Calcd for $C_{30}H_{38}F_3NNaO4SSi^+$ 616.2135; Found 616.2148.

(*E*)-3-Tosyl-2-(trifluoromethyl)-4-(1-(4-(trifluoromethyl)phenyl)-3-(triisopropylsilyl)prop-2-yn-1-ylidene)oxazolidine (4faf)

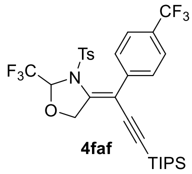

4faf

Prepared according to the general procedure D3 using 3-(4-(trifluoromethyl)phenyl)prop-2-yn-1-ol **1f** (80.1 mg, 400 µmol, 1.00 equiv.) and 1-bromo-2-(triisopropylsilyl)acetylene **2a** (136 mg, 520 µmol, 1.30 equiv.). The crude material was purified by column chromatography (0 – 10 % (v/v) Et_2O in pentane) to give **4faf** (234 mg, 0.370 mmol, 93% yield) as a pale-yellow amorphous solid.

R_f (20% Et_2O/Pentane) = 0.78.

^{1}H NMR (400 MHz, $CDCl_3$) δ 7.73 (d, *J* = 8.2 Hz, 2H, Ar*H*), 7.55 (d, *J* = 8.3 Hz, 2H, Ar*H*), 7.49 (d, *J* = 8.3 Hz, 2H, Ar*H*), 7.25 (d, *J* = 8.3 Hz, 2H, Ar*H*), 5.95 (q, *J* = 5.0 Hz, 1H, CF_3-C*H*), 4.69 (d, *J* = 13.6 Hz, 1H, O-CH_2-C), 4.01 (d, *J* = 13.6 Hz, 1H, O-CH_2-C), 2.43 (s, 3H, Ar-CH_3), 1.08 (d, *J* = 2.8 Hz, 21H, Si(C*H*(CH_3)$_2$)$_3$).

^{13}C{^{1}H} NMR (101 MHz, $CDCl_3$) δ 145.8, 140.3, 138.8, 133.7, 130.4 (q, $J_{C\text{-}F}$ = 32.3 Hz), 129.9, 128.6, 127.78, 125.2 (q, $J_{C\text{-}F}$ = 11.4 Hz), 124.0 (q, $J_{C\text{-}F}$ = 272.0 Hz), 122.3 (q, $J_{C\text{-}F}$ = 285.3 Hz), 114.0, 102.6, 101.2, 89.4 (t, $J_{C\text{-}F}$ = 35.8 Hz), 71.2, 21.6, 18.6, 11.1.

^{19}F NMR (376 MHz, $CDCl_3$) δ -62.6, -80.0 (d, *J* = 5.0 Hz).

IR (cm^{-1}) 2946 (m), 2867 (m), 2366 (w), 2143 (w), 1381 (m), 1324 (s), 1159 (s), 1128 (s).

HRMS (ESI/QTOF) m/z: $[M + Na]^+$ Calcd for $C_{30}H_{35}F_6NNaO_3SSi^+$ 654.1903; Found 654.1910.

(*E*)-4-(1-(2-Methoxyphenyl)-3-(triisopropylsilyl)prop-2-yn-1-ylidene)-3-tosyl-2-(trifluoromethyl)oxazolidine (4naf)

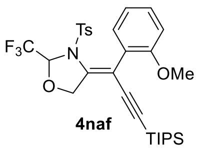

4naf

Prepared according to the general procedure D3 using 3-(2-methoxyphenyl)prop-2-yn-1-ol **1n** (64.9 mg, 400 μmol, 1.00 equiv.) and 1-bromo-2-(triisopropylsilyl)acetylene **2a** (136 mg, 520 μmol, 1.30 equiv.). The crude material was purified by column chromatography (0 – 10 % (v/v) Et_2O in pentane) to give **4naf** (122 mg, 0.205 mmol, 51% yield) as a pale-yellow amorphous solid.

R_f (20% Et_2O/Pentane) = 0.63.

^{1}H NMR (400 MHz, $CDCl_3$) δ 7.56 (d, *J* = 8.3 Hz, 2H, Ar*H*), 7.43 – 7.20 (m, 4H, Ar*H*), 6.96 (td, *J* = 7.5, 0.9 Hz, 1H, Ar*H*), 6.86 (d, *J* = 8.2 Hz, 1H, Ar*H*), 5.92 (q, *J* = 5.1 Hz, 1H, CF_3-C*H*), 4.70 (d, *J* = 13.4 Hz, 1H, O-CH_2-C), 3.92 (d, *J* = 13.4 Hz, 1H, O-CH_2-C), 3.84 (s, 3H, Ar-O-CH_3), 2.43 (s, 3H, Ar-CH_3), 1.05 (s, 21H, Si(C*H*(CH_3)$_2$)$_3$).

^{13}C{^{1}H} NMR (101 MHz, $CDCl_3$) δ 157.1, 145.1, 140.1, 134.1, 130.1, 129.6, 127.9, 124.7, 122.3 (q, $J_{C\text{-}F}$ = 285.5 Hz), 120.3, 111.2, 110.8, 104.0, 98.1, 90.1 (q, $J_{C\text{-}F}$ = 36.1 Hz), 71.4, 55.1, 21.7, 18.6, 11.1.

^{19}F NMR (376 MHz, $CDCl_3$) δ -80.0 (d, *J* = 5.1 Hz).

IR (cm^{-1}) 2942 (m), 2867 (m), 2362 (w), 2140 (w), 1463 (m), 1378 (m), 1177 (s), 1155 (s).

HRMS (ESI/QTOF) m/z: [M + Na]$^+$ Calcd for $C_{30}H_{38}F_3NNaO_4SSi^+$ 616.2135; Found 616.2146.

(*Z*)-4-(1-((*tert*-Butyldimethylsilyl)oxy)-4-(triisopropylsilyl)but-3-yn-2-ylidene)-3-tosyl-2-(trifluoromethyl)oxazolidine (4jaf)

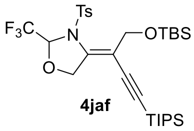

4jaf

Prepared according to the general procedure D3 using 4-((*tert*-butyldimethylsilyl)oxy)but-2-yn-1-ol **1j** (80.1 mg, 400 μmol, 1.00 equiv.) and 1-bromo-2-(triisopropylsilyl)acetylene **2a** (136 mg, 520 μmol, 1.30 equiv.). The crude material was purified by column chromatography (0 – 10 % (v/v) Et_2O in pentane) to give **4jaf** (215 mg, 0.341 mmol, 85% yield) as a pale-yellow amorphous solid.

R_f (10% Et_2O/Pentane) = 0.46.

^{1}H NMR (400 MHz, $CDCl_3$) δ 7.68 (d, *J* = 8.4 Hz, 2H, Ar*H*), 7.32 (d, *J* = 8.0 Hz, 2H, Ar*H*), 5.74 (q, *J* = 4.9 Hz, 1H, CF_3-C*H*), 4.64 (d, *J* = 13.2 Hz, 1H, CH_2-OTBS), 4.51 – 4.41 (m, 2H, O-CH_2-C and CH_2-OTBS), 3.53 (d, *J* = 13.5 Hz, 1H, O-CH_2-C), 2.45 (s, 3H, Ar-CH_3), 1.06 (s, 21H, Si(C*H*(CH_3)$_2$)$_3$), 0.91 (s, 9H, Si-C(CH_3)$_3$), 0.08 (d, *J* = 7.7 Hz, 6H, Si-(CH_3)$_2$).

^{13}C{^{1}H} NMR (101 MHz, $CDCl_3$) δ 146.0, 138.2, 133.3, 130.1, 128.3, 122.1 (q, $J_{C\text{-}F}$ = 285.1 Hz), 118.0, 102.4, 100.7, 89.5 (q, $J_{C\text{-}F}$ = 35.8 Hz), 70.3, 62.1, 26.0, 21.9, 18.7, 18.5, 11.2, -5.2, -5.3.

^{19}F NMR (376 MHz, $CDCl_3$) δ -80.2 (d, *J* = 4.9 Hz).

IR (cm^{-1}) 2944 (m), 2863 (m), 2360 (m), 2339 (m), 2154 (w), 1465 (m), 1375 (m), 1191 (s), 1153 (s).

HRMS (ESI/QTOF) m/z: [M + Na]$^+$ Calcd for $C_{30}H_{48}F_3NNaO_4SSi_2^+$ 654.2687; Found 654.2685.

(*E*)-5-Methyl-3-tosyl-2-(trifluoromethyl)-4-(4-(triisopropylsilyl)but-3-yn-2-ylidene)oxazolidine (4laf)

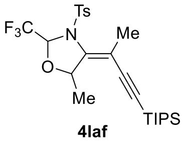

4laf

Prepared according to the general procedure D3 using **1l** (33.7 mg, 400 μmol, 1.00 equiv.) and 1-bromo-2-(triisopropylsilyl)acetylene **2a** (136 mg, 520 μmol, 1.30 equiv.). The crude material was purified by column chromatography (0 – 10 % (v/v) Et_2O in pentane) to give **4laf** (1:1 *dr*, 142 mg, 0.269 mmol, 67% yield) as a pale-yellow oil. The diastereomeric ratio was determined by integration of ^{19}F NMR spectra and was found unchanged before and after purification.

R_f (10% Et_2O/Pentane) = 0.45.

Diastereoisomer 1:

^{1}H NMR (400 MHz, $CDCl_3$) δ 7.79 – 7.73 (m, 2H, Ar*H*), 7.36 (d, *J* = 8.0 Hz, 2H, Ar*H*), 5.77 (q, *J* = 5.0 Hz, 1H, CF_3-C*H*), 4.89 (qd, *J* = 6.6, 1.4 Hz, 1H, CH_3-C*H*), 2.46 (s, 3H, Ar-CH_3), 2.11 (d, *J* = 1.4 Hz, 3H, C=C-CH_3), 1.07 (d, *J* = 2.5 Hz, 21H, Si(C*H*(CH_3)$_2$)$_3$), 0.85 (d, *J* = 6.4 Hz, 3H, CH-CH_3).

^{19}F NMR (376 MHz, $CDCl_3$) δ -80.73 (d, *J* = 5.0 Hz).

Diastereoisomer 2:

^{1}H NMR (400 MHz, $CDCl_3$) δ 7.72 – 7.67 (m, 2H, Ar*H*), 7.32 (d, *J* = 8.0 Hz, 2H, Ar*H*), 5.60 (q, *J* = 5.0 Hz, 1H, CF_3-C*H*), 3.68 (dtt, *J* = 6.3, 4.3, 2.1 Hz, 1H, CH_3-C*H*), 2.46 (s, 3H, Ar-CH_3), 2.23 (d, *J* = 2.1 Hz, 3H, C=C-CH_3), 1.51 (d, *J* = 6.3 Hz, 3H, CH-CH_3), 1.07 (d, *J* = 2.5 Hz, 21H, Si(C*H*(CH_3)$_2$)$_3$).

^{19}F NMR (376 MHz, $CDCl_3$) δ -80.69 (d, *J* = 5.0 Hz).

Given as a list of peaks due to the heavy overlap of peaks:

^{13}C{^{1}H} NMR (101 MHz, $CDCl_3$) δ 145.8, 145.7, 141.7, 141.6, 134.9, 133.6, 130.2, 130.1, 128.5, 128.3, 122.3 (q, $J_{C\text{-}F}$ = 286.1 Hz), 122.0 (q, $J_{C\text{-}F}$ = 284.6 Hz), 114.4, 113.0, 104.8, 100.5, 99.9, 87.7 (q, $J_{C\text{-}F}$ = 35.8 Hz), 86.7 (q, $J_{C\text{-}F}$ = 35.9 Hz), 78.9, 77.3, 22.9, 21.9, 21.8, 21.1, 18.73, 18.71, 11.32, 11.30.

IR (cm^{-1}) 2957 (w), 2871 (w), 2361 (w), 2258 (w), 2136 (w), 1372 (w), 1188 (m), 907 (s).

HRMS (ESI/QTOF) m/z: $[M + Na]^+$ Calcd for $C_{25}H_{36}F_3NNaO_3SSi^+$ 538.2029; Found 538.2034.

(*E*)-4-(1-(*p*-Tolyl)ethylidene)-3-tosyl-2-(trifluoromethyl)oxazolidine (4aff)

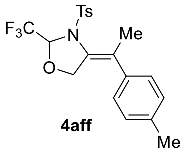

4aff

3 mmol scale:

Prepared according to the general procedure D1 using but-2-yn-1-ol **1a** (210 mg, 224 µL, 3.00 mmol, 1.00 equiv.) and 4-iodotoluene **2f** (850 mg, 3.90 mmol, 1.30 equiv.). The crude material was purified by column chromatography (0 – 10 % (v/v) Et_2O in pentane) to give **4aff** (783 mg, 1.90 mmol, 63% yield) as a pale-yellow amorphous solid.

R_f (20% Et_2O/Pentane) = 0.47.

^{1}H NMR (400 MHz, $CDCl_3$) δ 7.81 (d, J = 8.2 Hz, 2H, Ar*H*), 7.40 (d, J = 8.1 Hz, 2H, Ar*H*), 7.13 (d, J = 7.9 Hz, 2H, Ar*H*), 6.92 (d, J = 8.0 Hz, 2H, Ar*H*), 5.72 (q, J = 4.7 Hz, 1H, CF_3-C*H*), 3.89 (d, J = 11.0 Hz, 1H, O-CH_2-C), 3.45 – 3.26 (m, 1H, O-CH_2-C), 2.50 (s, 3H, SO_2-Ph-CH_3), 2.39 (s, 3H, C=C-CH_3), 2.34 (s, 3H, Ar-CH_3).

^{13}C{^{1}H} NMR (101 MHz, $CDCl_3$) δ 145.7, 138.5, 137.2, 136.2, 134.2, 130.2, 129.4, 128.3, 128.0, 127.1, 122.3 (q, J_{C-F} = 284.6 Hz), 87.0 (q, J_{C-F} = 35.7 Hz), 68.0, 21.9, 21.3, 21.0.

^{19}F NMR (376 MHz, $CDCl_3$) δ -80.7 (d, J = 4.8 Hz).

IR (cm^{-1}) 2926 (w), 2359 (w), 1718 (w), 1512 (m), 1365 (m), 1290 (m), 1174 (s), 1155 (s).

HRMS (APCI/QTOF) m/z: $[M + Na]^+$ Calcd for $C_{20}H_{20}F_3NNaO_3S^+$ 434.1008; Found 434.1003.

(*E*)-3-Tosyl-2-(trifluoromethyl)-4-(1-(4-(trifluoromethyl)phenyl)ethylidene)oxazolidine (4ahf)

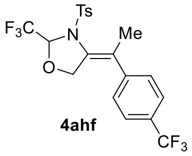

4ahf

Prepared according to the general procedure D3 using but-2-yn-1-ol **1a** (28 mg, 30 µL, 400 µmol, 1.00 equiv.) and 1-iodo-4-(trifluoromethyl)benzene **2h** (141 mg, 520 µmol, 1.30 equiv.). The crude material was purified by column chromatography (0 – 10 % (v/v) Et_2O in pentane) to give **4ahf** (139 mg, 0.299 mmol, 75% yield) as a pale-yellow amorphous solid.

R_f (20% Et_2O/Pentane) = 0.48.

^{1}H NMR (400 MHz, $CDCl_3$) δ 7.85 – 7.77 (m, 2H, Ar*H*), 7.60 (d, J = 8.1 Hz, 2H, Ar*H*), 7.42 (d, J = 8.0 Hz, 2H, Ar*H*), 7.15 (d, J = 8.0 Hz, 2H, Ar*H*), 5.75 (q, J = 4.8 Hz, 1H, CF_3-C*H*), 3.86 (dd, J = 11.3, 1.3 Hz, 1H, O-CH_2-C), 3.37 (dd, J = 11.3, 2.1 Hz, 1H, O-CH_2-C), 2.51 (s, 3H, Ar-CH_3), 2.42 (dd, J = 2.0, 1.3 Hz, 3H, CH_3).

^{13}C{^{1}H} NMR (101 MHz, $CDCl_3$) δ 146.0, 143.9, 134.6, 134.1, 130.6 (q, J_{C-F} = 32.6 Hz), 130.3, 130.0, 128.2, 127.6, 125.8 (q, J_{C-F} = 3.7 Hz), 124.0 (q, J_{C-F} = 272.0 Hz), 122.2 (q, J_{C-F} = 284.7 Hz), 87.3 (q, J_{C-F} = 35.8 Hz), 67.9, 21.9, 21.2.

^{19}F NMR (376 MHz, $CDCl_3$) δ -62.8, -80.7 (d, J = 4.8 Hz).

IR (cm^{-1}) 2356 (w), 1620 (w), 1598 (w), 1368 (m), 1325 (s), 1167 (s), 1103 (s).

HRMS (ESI/QTOF) m/z: $[M + Na]^+$ Calcd for $C_{20}H_{17}F_6NNaO_3S^+$ 488.0726; Found 488.0724.

(*E*)-4-(Phenyl(4-(trifluoromethyl)phenyl)methylene)-3-tosyl-2-(trifluoromethyl)oxazolidine (4bhf)

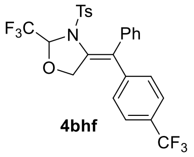

4bhf

Prepared according to the general procedure D3 using 3-phenylprop-2-yn-1-ol **1b** (53 mg, 50 µL, 400 µmol, 1.00 equiv.) and 1-iodo-4-(trifluoromethyl)benzene **2h** (141 mg, 520 µmol, 1.30 equiv.). The crude material was purified by column chromatography (0 – 10 % (v/v) Et_2O in pentane) to give **4bhf** (206 mg, 0.391 mmol, 98% yield) as a pale-yellow amorphous solid.

R_f (20% Et_2O/Pentane) = 0.51.

^{1}H NMR (400 MHz, $CDCl_3$) δ 7.52 – 7.40 (m, 4H, Ar*H*), 7.25 – 7.12 (m, 7H, Ar*H*), 7.01 (d, J = 8.1 Hz, 2H, Ar*H*), 5.84 (q, J = 4.9 Hz, 1H, CF_3-C*H*), 4.02 (d, J = 11.9 Hz, 1H, O-CH_2-C), 3.96 (d, J = 11.9 Hz, 1H, O-CH_2-C), 2.38 (s, 3H, Ar-CH_3).

^{13}C{^{1}H} NMR (101 MHz, $CDCl_3$) δ 145.5, 143.8, 138.8, 135.4, 134.5, 130.8, 130.7 (q, J_{C-F} = 32.7 Hz), 130.0, 129.8, 129.6, 128.5, 128.3, 128.1, 125.6 (q, J_{C-F} = 3.7 Hz), 124.0 (d, J_{C-F} = 272.2 Hz), 122.5 (d, J_{C-F} = 285.0 Hz), 68.8, 21.8.

^{19}F NMR (376 MHz, $CDCl_3$) δ -62.8, -79.9 (d, J = 4.9 Hz).

IR (cm^{-1}) 2359 (w), 1598 (w), 1372 (w), 1324 (s), 1156 (s), 905 (s).

HRMS (ESI/QTOF) m/z: $[M + Na]^+$ Calcd for $C_{25}H_{19}F_6NNaO_3S^+$ 550.0882; Found 550.0890.

(*E*)-3-((4-Nitrophenyl)sulfonyl)-4-(phenyl(4-(trifluoromethyl)phenyl)methylene)-2-(trifluoromethyl)oxazolidine (4bhg)

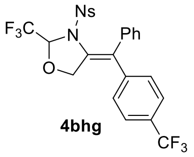

4bhg

Prepared according to the general procedure D3 using 3-phenylprop-2-yn-1-ol **1b** (53 mg, 50 µL, 400 µmol, 1.00 equiv.), 1-iodo-4-(trifluoromethyl)benzene **2h** (141 mg, 520 µmol, 1.30 equiv.) and **3g** (184 mg, 560 µmol, 1.40 equiv.). The crude material was purified by column chromatography (0 – 10 % (v/v) Et_2O in pentane) to give **4bhg** (153 mg, 0.274 mmol, 68% yield) as a pale-yellow amorphous solid.

Rf (20% Et_2O/Pentane) = 0.43.

^{1}H NMR (400 MHz, $CDCl_3$) δ 8.16 – 8.07 (m, 2H, Ar*H*), 7.60 (d, *J* = 8.1 Hz, 2H, Ar*H*), 7.57 – 7.51 (m, 2H, Ar*H*), 7.24 – 7.04 (m, 7H, Ar*H*), 6.10 (q, *J* = 4.9 Hz, 1H, CF_3-C*H*), 4.58 (d, *J* = 11.9 Hz, 1H, O-CH_2-C), 4.31 (d, *J* = 11.9 Hz, 1H, O-CH_2-C).

^{13}C{^{1}H} NMR (101 MHz, $CDCl_3$) δ 150.4, 144.0, 143.2, 138.5, 135.4, 131.1 (q, J_{C-F} = 32.8 Hz), 130.7, 129.83, 129.6, 128.8, 128.7, 128.6, 125.8 (q, J_{C-F} = 3.7 Hz), 124.1, 123.9 (q, J_{C-F} = 272.3 Hz), 122.4 (q, J_{C-F} = 284.7 Hz), 86.9 (q, J_{C-F} = 36.0 Hz), 69.1.

^{19}F NMR (376 MHz, $CDCl_3$) δ -62.8, -79.7 (d, *J* = 4.9 Hz).

IR (cm^{-1}) 2357 (w), 1614 (w), 1537 (m), 1353 (m), 1327 (s), 1171 (s), 1156 (s).

HRMS (ESI/QTOF) m/z: [M + H^{-1}]$^-$ Calcd for $C_{24}H_{15}F_6N_2O_5S^-$ 557.0611; Found 557.0610.

(*E*)-3-((4-Nitrophenyl)sulfonyl)-2-(trifluoromethyl)-4-(1-(4-(trifluoromethyl)phenyl)ethylidene)oxazolidine (4ahg)

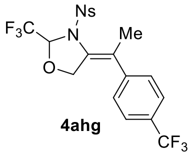

4ahg

Prepared according to the general procedure D3 using but-2-yn-1-ol **1a** (28 mg, 30 μL, 0.40 mmol, 1.00 equiv.), 1-iodo-4-(trifluoromethyl)benzene **2h** (141 mg, 520 μmol, 1.30 equiv.) and **3g** (184 mg, 560 μmol, 1.40 equiv.). The crude material was purified by column chromatography (0 – 10 % (v/v) Et_2O in pentane) to give **4ahg** (123 mg, 0.248 mmol, 62% yield) as a pale-yellow amorphous solid.

Rf (20% Et_2O/Pentane) =

^{1}H NMR (400 MHz, $CDCl_3$) δ 8.52 – 8.41 (m, 2H, Ar*H*), 8.19 – 8.09 (m, 2H, Ar*H*), 7.63 (d, *J* = 8.1 Hz, 2H, Ar*H*), 7.17 (d, *J* = 8.0 Hz, 2H, Ar*H*), 5.76 (q, *J* = 4.7 Hz, 1H, CF_3-C*H*), 3.96 (dd, *J* = 11.6, 1.3 Hz, 1H, O-CH_2-C), 3.58 – 3.47 (m, 1H, O-CH_2-C), 2.45 – 2.39 (m, 3H, CH_3).

^{13}C{^{1}H} NMR (101 MHz, $CDCl_3$) δ 151.3, 143.2, 142.8, 135.9, 131.1 (q, J_{C-F} = 33.2 Hz), 129.6, 128.9, 127.5, 126.1 (q, *J* = 3.8 Hz), 124.8, 123.8 (q, J_{C-F} = 272.4 Hz), 122.0 (q, J_{C-F} = 285.3 Hz), 87.1 (d, J_{C-F} = 36.2 Hz), 68.0, 21.4.

^{19}F NMR (376 MHz, $CDCl_3$) δ -62.9, -80.4 (d, *J* = 4.5 Hz).

IR (cm^{-1}) 3108 (w), 2921 (w), 2258 (w), 1536 (w), 1325 (m), 1181 (m), 903 (s).

Methyl (*E*)-4-(1-(3-tosyl-2-(trifluoromethyl)oxazolidin-4-ylidene)ethyl)benzoate (4anf)

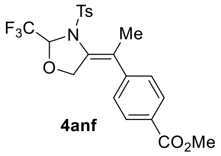

4anf

Prepared according to the general procedure D3 but-2-yn-1-ol **1a** (28 mg, 30 μL, 0.40 mmol, 1.00 equiv.) and methyl 4-iodobenzoate **2n** (136 mg, 520 μmol, 1.30 equiv.). The crude material was purified by column chromatography (0 – 10 % (v/v) Et_2O in pentane) to give **4anf** (129 mg, 0.284 mmol, 71% yield) as a pale-yellow amorphous solid.

Rf (20% Et_2O/Pentane) = 0.17.

^{1}H NMR (400 MHz, $CDCl_3$) δ 8.00 (d, *J* = 8.4 Hz, 2H, Ar*H*), 7.81 (d, *J* = 8.3 Hz, 2H, Ar*H*), 7.41 (d, *J* = 8.0 Hz, 2H, Ar*H*), 7.10 (d, *J* = 8.4 Hz, 2H, Ar*H*), 5.74 (q, *J* = 4.7 Hz, 1H, CF_3-C*H*), 3.92 (s, 3H, CO_2-CH_3), 3.86 (dd, *J* = 11.3, 1.3 Hz, 1H, O-CH_2-C), 3.37 (dd, *J* = 11.3, 2.1 Hz, 1H, O-CH_2-C), 2.51 (s, 3H, Ar-CH_3), 2.44 – 2.40 (m, 3H, CH_3).

^{13}C{^{1}H} NMR (101 MHz, $CDCl_3$) δ 166.6, 146.0, 144.8, 135.1, 134.1, 130.3, 130.2, 130.1, 129.8, 128.3, 127.3, 122.2 (q, J_{C-F} = 285.0 Hz), 87.2 (q, J_{C-F} = 35.9 Hz), 67.9, 52.4, 21.9, 21.1.

^{19}F NMR (376 MHz, $CDCl_3$) δ -80.7 (d, *J* = 4.8 Hz).

IR (cm^{-1}) 2954 (w), 2359 (w), 1723 (s), 1609 (w), 1368 (m), 1283 (s), 1177 (s).

HRMS (ESI/QTOF) m/z: [M + Na]$^+$ Calcd for $C_{21}H_{20}F_3NNaO_5S^+$ 478.0906; Found 478.0913.

(*E*)-4-(1-(3,5-Bis(trifluoromethyl)phenyl)ethylidene)-3-tosyl-2-(trifluoromethyl)oxazolidine (4aof)

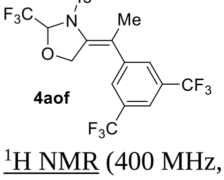

4aof

Prepared according to the general procedure D3 but-2-yn-1-ol **1a** (28 mg, 30 μL, 0.40 mmol, 1.00 equiv.) and 1-iodo-3,5-bis(trifluoromethyl)benzene **2o** (177 mg, 520 μmol, 1.30 equiv.). The crude material was purified by column chromatography (0 – 10 % (v/v) Et_2O in pentane) to give **4aof** (155 mg, 0.291 mmol, 73% yield) as a pale-yellow amorphous solid.

Rf (20% Et_2O/Pentane) = 0.67.

^{1}H NMR (400 MHz, $CDCl_3$) δ 7.81 (d, *J* = 8.3 Hz, 3H, Ar*H*), 7.43 (d, *J* = 8.2 Hz, 4H, Ar*H*), 5.82 (q, *J* = 4.7 Hz, 1H, CF_3-C*H*), 3.80 (dd, *J* = 11.4, 1.3 Hz, 1H, O-CH_2-C), 3.30 (dd, *J* = 11.4, 2.1 Hz, 1H, O-CH_2-C), 2.52 (s, 3H, Ar-CH_3), 2.46 – 2.44 (m, 3H, CH_3).

^{13}C{^{1}H} NMR (101 MHz, $CDCl_3$) δ 146.5, 142.3, 133.8, 132.5, 132.4 (q, J_{C-F} = 33.6 Hz), 131.7, 130.3, 128.3, 127.5, 127.4, 123.1 (q, J_{C-F} = 272.8 Hz), 122.5 – 122.2 (m), 122.16 (q, J_{C-F} = 284.6 Hz), 87.5 (t, J_{C-F} = 35.8 Hz), 67.6, 21.9, 21.2.

^{19}F NMR (376 MHz, $CDCl_3$) δ -63.0, -80.7 (d, *J* = 4.8 Hz).

IR (cm^{-1}) 2359 (w), 1382 (m), 1281 (s), 1177 (s), 1141 (s), 1051 (w).

$\underline{\text{HRMS}}$ (APCI/QTOF) m/z: [M + H]$^+$ Calcd for $C_{21}H_{17}F_9NO_3S^+$ 534.0780; Found 534.0787.

(*E*)-4-(1-(2-Fluorophenyl)ethylidene)-3-tosyl-2-(trifluoromethyl)oxazolidine (4apf)

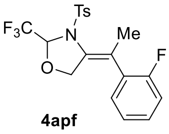

4apf

Prepared according to the general procedure D3 using but-2-yn-1-ol **1a** (28 mg, 30 µL, 0.40 mmol, 1.00 equiv.) and 1-fluoro-2-iodobenzene **2p** (115 mg, 520 µmol, 1.30 equiv.). The crude material was purified by column chromatography (0 – 10 % (v/v) Et_2O in pentane) to give **4apf** (119 mg, 0.286 mmol, 71% yield) as a pale-yellow amorphous solid.

R_f (20% Et_2O/Pentane) = 0.45.

^{1}H NMR (400 MHz, $CDCl_3$) δ 7.83 (d, *J* = 8.3 Hz, 2H, Ar*H*), 7.37 (d, *J* = 8.1 Hz, 2H, Ar*H*), 7.33 – 7.26 (m, 1H, Ar*H*), 7.17 – 7.01 (m, 3H, Ar*H*), 5.71 (q, *J* = 4.8 Hz, 1H, CF_3-C*H*), 3.84 (dd, *J* = 11.3, 1.3 Hz, 1H, O-CH_2-C), 3.31 – 3.22 (m, 1H, O-CH_2-C), 2.47 (s, 3H, Ar-CH_3), 2.43 – 2.36 (m, 3H, CH_3).

^{13}C{^{1}H} NMR (101 MHz, $CDCl_3$) δ 127.62 (d, J_{C-F} = 15.9 Hz), 145.7, 133.9, 130.2, 130.15, 130.1, 129.1 (d, *J* = 3.4 Hz), 128.4, 127.6 (d, J_{C-F} = 15.9 Hz), 124.6 (d, J_{C-F} = 3.6 Hz), 122.3 (q, J_{C-F} = 284.9 Hz), 116.2 (d, J_{C-F} = 22.3 Hz), 87.8 (q, J_{C-F} = 35.8 Hz), 68.2 (d, J_{C-F} = 4.5 Hz), 21.9, 21.5.

^{19}F NMR (376 MHz, $CDCl_3$) δ -80.6 (d, *J* = 4.8 Hz), -115.1 (dt, *J* = 11.2, 6.2 Hz).

IR (cm^{-1}) 2359 (w), 1492 (m), 1368 (m), 1289 (m), 1173 (s), 1153 (s).

HRMS (ESI/QTOF) m/z: [M + Na]$^+$ Calcd for $C_{19}H_{17}F_4NNaO_3S^+$ 438.0757; Found 438.0768.

D.5. Unsuccessful substrates

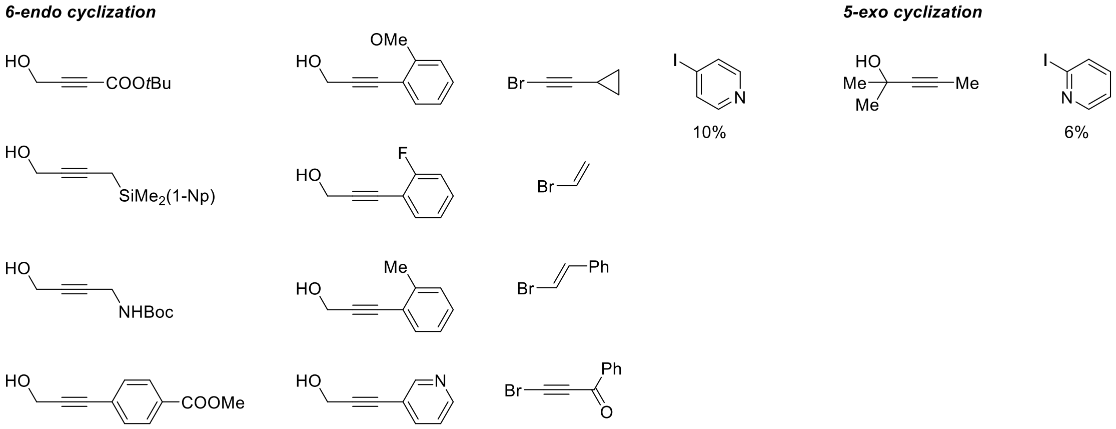

E. Product modifications

E.1. Hydrogenation

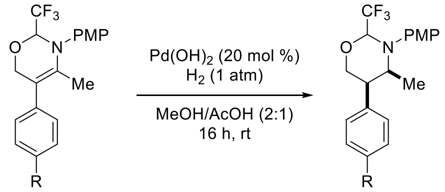

General Procedure E1: An oven-dried 25 mL round-bottom flask equipped with a Teflon coated stirring bar was charged with $Pd(OH)_2$/C (20 mol%) and the tetrasubstituted olefin (1.00 equiv.). The flask was sealed and evacuated and back-filled with N_2 three times. MeOH (1.3 mL) and AcOH (0.6 mL) were added and the suspension was stirred at room temperature for 10 minutes under a nitrogen flow. Then, a hydrogen balloon was connected to the flask through a needle and the mixture was vigorously stirred at room temperature for 16 hours. Then, the reaction mixture was degassed by bubbling nitrogen for 10 minutes and filtered through a plug of celite eluting with 10 mL of MeOH. The crude extract was washed with saturated $NaHCO_3$ and extracted with DCM (3 × 25 mL). The combined organic layer was dried over Na_2SO_4, filtered and concentrated in vacuum. The crude material was purified by flash column chromatography on silica gel to afford the corresponding product as a single diastereoisomer.

3-(4-Methoxyphenyl)-4-methyl-5-(p-tolyl)-2-(trifluoromethyl)-1,3-oxazinane (6af)

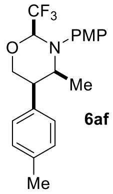

Prepared according to the general procedure E1 using **5af** (36 mg, 0.10 mmol, 1.00 equiv.). The crude material was purified by column chromatography (8:2 pentane/EtOAc) to give **6af** (27 mg, 74 µmol, 74% yield) as a colorless oil and as a single diastereoisomer.

R_f (10% Et_2O/Pentane) = 0.46.

^{1}H NMR (400 MHz, $CDCl_3$) δ 7.46 (d, *J* = 8.0 Hz, 2H, Ar*H*), 7.19 – 7.10 (m, 4H, Ar*H*), 6.89 – 6.81 (m, 2H, Ar*H*), 4.92 (q, *J* = 4.8 Hz, 1H, CF_3-C*H*), 4.33 (dd, *J* = 11.3, 3.6 Hz, 1H, O-CH_2-C), 4.13 (dd, *J* = 11.3, 3.7 Hz, 1H, O-CH_2-C), 3.80 (s, 3H, Ar-O-CH_3), 3.55 (qd, *J* = 6.6, 4.3 Hz, 1H, C*H*-CH_3), 2.88 (q, *J* = 3.7 Hz, 1H, C*H*-Ar), 2.35 (s, 3H, Ar-CH_3), 0.69 (d, *J* = 6.6 Hz, 3H, CH_3).

^{13}C{^{1}H} NMR (101 MHz, $CDCl_3$) δ 157.8, 139.7, 137.1, 136.4, 129.8, 128.9, 127.7, 122.8 (q, J_{C-F} = 284.5 Hz), 114.3, 88.2 (q, J_{C-F} = 30.5 Hz), 70.1, 58.7, 55.5, 44.7, 21.2, 18.2.

^{19}F NMR (376 MHz, $CDCl_3$) δ -74.4 (d, *J* = 4.8 Hz).

IR (cm^{-1}) 2973 (w), 2853 (w), 2362 (w), 1609 (w), 1509 (s), 1460 (w), 1239 (m), 1170 (s), 821 (m).

HRMS (ESI/QTOF) m/z: [M + H]$^+$ Calcd for $C_{20}H_{23}F_3NO_2^+$ 366.1675; Found 366.1683.

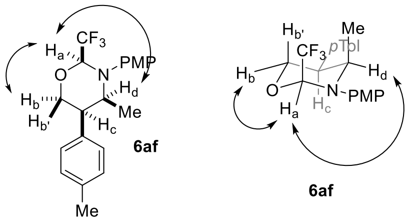

NOE 1D, set o1 4.92 ppm (H_a) on the site to be irradiated, d8 300 ms

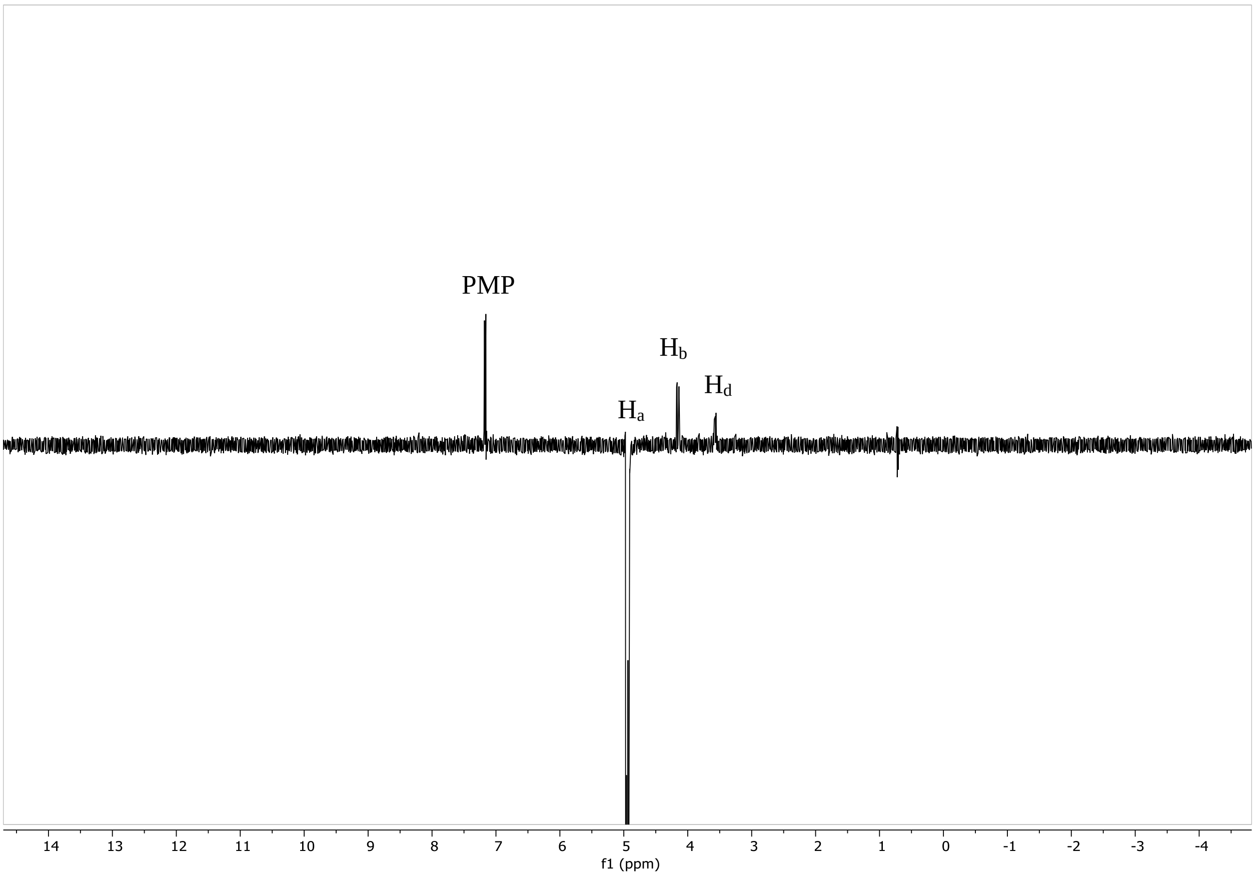

3-(4-Methoxyphenyl)-4-methyl-2-(trifluoromethyl)-5-(4-(trifluoromethyl)phenyl)-1,3-oxazinane (6ah)

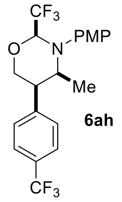

6ah

Prepared according to the general procedure E1 using **5ah** (56 mg, 0.13 mmol, 1.00 equiv.). The crude material was purified by column chromatography (8:2 pentane/EtOAc) to give **6ah** (30 mg, 72 µmol, 54% yield) as a colorless oil and as a single diastereoisomer.

R_f (9:1 Pentane/EtOAc) = 0.20.

^{1}H NMR (400 MHz, $CDCl_3$) δ 7.72 (d, *J* = 8.1 Hz, 2H, Ar*H*), 7.52 (d, *J* = 8.2 Hz, 2H, Ar*H*), 7.11 – 7.02 (m, 2H, Ar*H*), 6.82 – 6.73 (m, 2H, Ar*H*), 4.80 (q, *J* = 4.3 Hz, 1H, CF_3-C*H*), 4.20 (dd, *J* = 11.5, 2.1 Hz, 1H, O-CH_2-C), 4.09 (dd, *J* = 11.5, 3.4 Hz, 1H, O-CH_2-C), 3.72 (s, 3H, Ar-O-CH_3), 3.48 (qd, *J* = 6.5, 3.9 Hz, 1H, C*H*-CH_3), 2.80 (q, *J* = 3.4 Hz, 1H, C*H*-Ar), 0.53 (d, *J* = 6.5 Hz, 3H, CH_3).

^{13}C{^{1}H} NMR (101 MHz, $CDCl_3$) δ 158.3, 144.3, 138.2, 130.6, 129.2 (q, J_{C-F} = 32.3 Hz), 128.5, 125.0 (q, J_{C-F} = 3.7 Hz), 124.5 (d, J_{C-F} = 271.6 Hz), 122.5 (q, J_{C-F} = 283.8 Hz), 114.4, 89.1 (q, J_{C-F} = 30.1 Hz), 70.9, 58.7, 55.5, 45.6, 18.6.

^{19}F NMR (376 MHz, $CDCl_3$) δ -62.4, -74.6 (d, *J* = 4.2 Hz).

IR (cm^{-1}) 2934 (w), 2858 (w), 2359 (m), 2327 (w), 1711 (w), 1613 (w), 1510 (m), 1325 (s), 1118 (s).

HRMS (nanochip-ESI/LTQ-Orbitrap) m/z: [M + H]$^+$ Calcd for $C_{20}H_{20}F_6NO_2^+$ 420.1393; Found 420.1383.

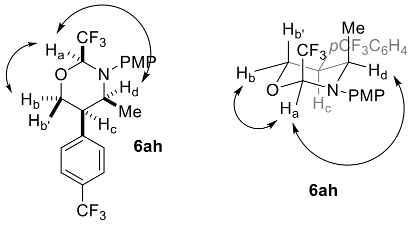

NOE 1D, set o1 4.80 ppm (H_a) on the site to be irradiated, d8 300 ms

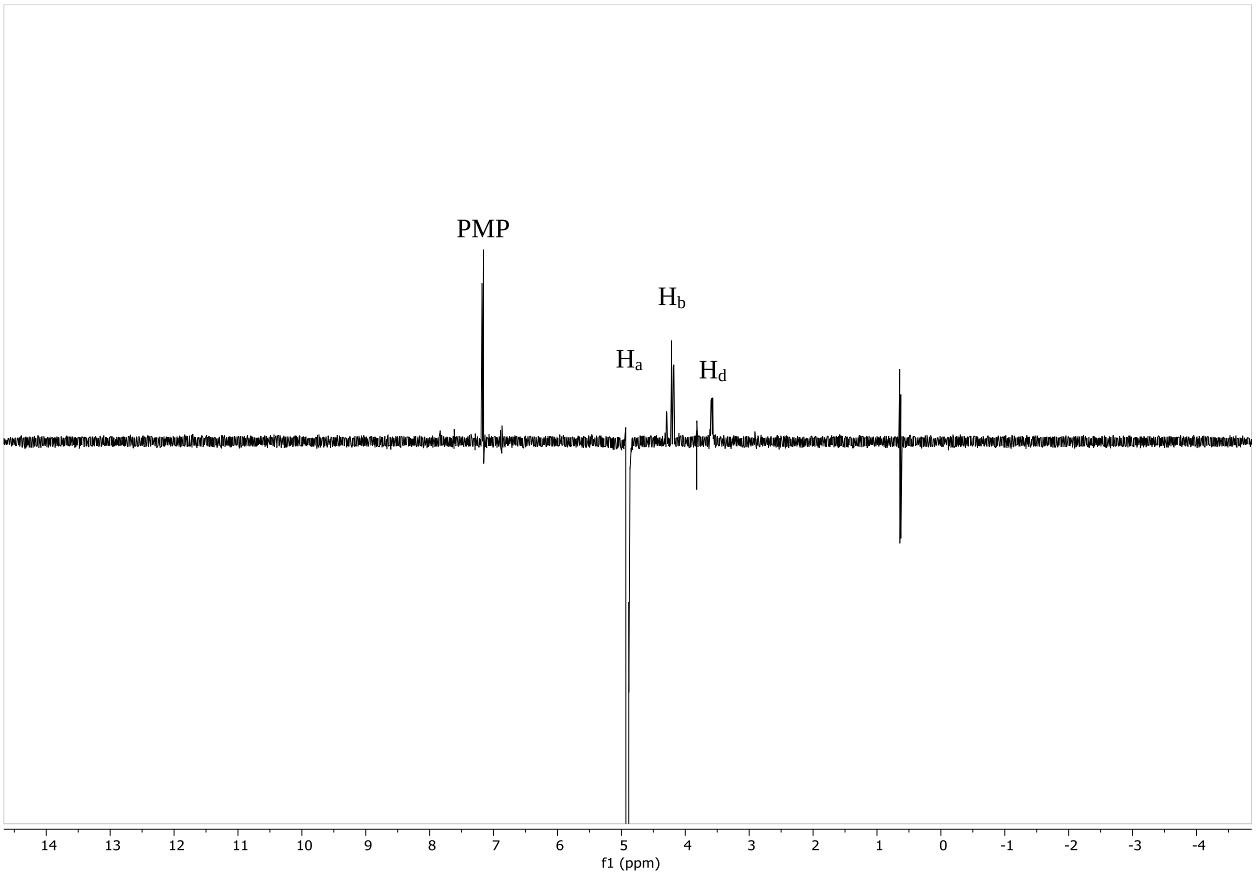

4-(1-(*p*-Tolyl)ethyl)-3-tosyl-2-(trifluoromethyl)oxazolidine (13)

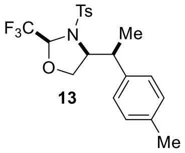

Prepared according to the general procedure E1 using **4aff** (41 mg, 0.10 mmol, 1.00 equiv.) and EtOAc (2 mL) as solvent. The crude material was purified by column chromatography (8:2 pentane/EtOAc) to give **13** (33 mg, 79 µmol, 79% yield) as a white amorphous solid and as a single diastereoisomer.

R_f (10% Et_2O/Pentane) = 0.41.

^{1}H NMR (400 MHz, $CDCl_3$) δ 7.55 (d, *J* = 8.1 Hz, 2H, Ar*H*), 7.27 (d, *J* = 8.3 Hz, 2H, Ar*H*), 7.06 (q, *J* = 8.0 Hz, 4H, Ar*H*), 5.49 (q, *J* = 5.3 Hz, 1H, CF_3-C*H*), 4.10 (ap dq, *J* = 20.7, 7.3 Hz, 2H, O-CH_2-C), 3.72 (ap t, *J* = 7.4 Hz, 1H, N-C*H*), 3.36 (p, *J* = 7.0 Hz, 1H, C*H*-CH_3), 2.43 (s, 3H, SO_2-Ar-CH_3), 2.32 (s, 3H, CH-Ar-CH_3), 1.28 (d, *J* = 7.1 Hz, 3H, CH-CH_3).

^{13}C{^{1}H} NMR (101 MHz, $CDCl_3$) δ 144.9, 139.2, 136.7, 134.1, 130.0, 129.4, 128.2, 127.6, 122.6 (q, J_{C-F} = 285.5 Hz), 87.8 (d, J_{C-F} = 36.0 Hz), 69.7, 65.3, 39.7, 21.8, 21.2, 14.4.

^{19}F NMR (376 MHz, $CDCl_3$) δ -77.8 (d, *J* = 5.2 Hz).

IR (cm^{-1}) 2975 (w), 2911 (w), 2362 (m), 2323 (w), 1512 (w), 1364 (m), 1289 (m), 1185 (s), 1159 (s).

HRMS (ESI/QTOF) m/z: $[M + Na]^+$ Calcd for $C_{20}H_{22}F_3NNaO_3S^+$ 436.1165; Found 436.1167.

4-(1-(*p*-Tolyl)ethyl)-2-(trifluoromethyl)oxazolidine (14)

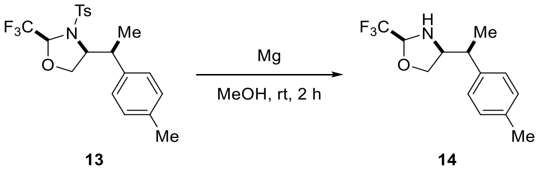

To a solution of **13** (27.0 mg, 65.3 µmol, 1.0 equiv.) in MeOH (6.5 mL) is added Mg powder (172 mg. 6.50 mmol, 100 equiv.). The reaction is fitted with an N_2 balloon and sonicated for 1 h. Additional Mg powder (172 mg. 6.50 mmol, 100 equiv.) was added, and the reaction is sonicated for another hour until all Mg is consumed. The reaction is quenched with saturated aqueous NH_4Cl (20 mL) and H_2O (20 mL), and the aqueous layer is extracted with DCM (3 × 40 mL). The combined organic layer is dried over Na_2SO_4, filtered, and concentrated under vacuum. The residue was purified by column chromatography on silica gel (0 – 10% (v/v) Et_2O in pentane) to yield **14** (8.5 mg, 33 µmol, 50% yield) as a colorless oil and as a single diastereoisomer.

R_f (10% Et_2O/Pentane) = 0.26.

^{1}H NMR (400 MHz, $CDCl_3$) δ 7.14 (s, 4H, Ar*H*), 4.88 – 4.78 (m, 1H, CF_3-C*H*), 4.17 (ap t, *J* = 7.1 Hz, 1H, O-CH_2-C), 3.71 (ap t, *J* = 8.3 Hz, 1H, O-CH_2-C), 3.55 (p, *J* = 7.9 Hz, 1H, NH-C*H*-CH_2), 2.78 – 2.66 (m, 1H, C*H*-CH_3), 2.33 (s, 3H, Ar-CH_3), 2.19 (t, *J* = 7.8 Hz, 1H, N*H*), 1.21 (d, *J* = 7.0 Hz, 3H, CH-CH_3).

^{13}C{^{1}H} NMR (101 MHz, $CDCl_3$) δ 141.5, 136.4, 129.4, 127.4, 123.5 (q, J_{C-F} = 283.1 Hz), 87.1 (q, J_{C-F} = 34.0 Hz), 71.4, 64.1, 43.5, 21.2, 18.9.

^{19}F NMR (376 MHz, $CDCl_3$) δ -81.8 (d, *J* = 5.0 Hz).

IR (cm^{-1}) 3364 (w), 2925 (m), 2855 (w), 2359 (m), 2330 (w), 2258 (w), 1725 (w), 1461 (w), 1294 (w), 1152 (m), 910 (m), 731 (s).

HRMS (ESI/QTOF) m/z: $[M + H]^+$ Calcd for $C_{13}H_{17}F_3NO^+$ 260.1257; Found 260.1264.

E.2. Tether opening

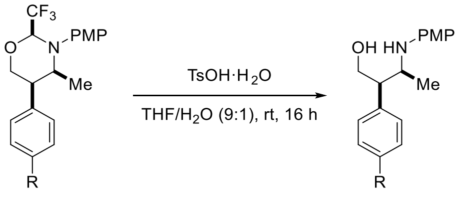

General Procedure E2: To a solution of starting material (1.00 equiv.) in THF/H_2O (9:1), was added TsOH·H_2O (7.00 equiv.). The reaction was stirred at room temperature for 16 h. Then the mixture was diluted with DCM (20 mL). The organic layer was washed with an aqueous 2 M NaOH solution (2 × 10 mL), dried over Na_2SO_4, filtered and concentrated under reduced pressure. The residue was purified by column chromatography to afford the corresponding product.

3-((4-Methoxyphenyl)amino)-2-(p-tolyl)butan-1-ol (7af)

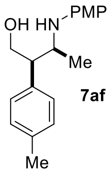

Prepared according to the general procedure E2 using **6af** (27 mg, 74 µmol, 1.00 equiv.). The crude material was purified by column chromatography on silica gel (1:1 pentane/EtOAc) to give **7af** (13 mg, 46 µmol, 62% yield) as a colorless oil.

$\underline{R_f}$ (1:1 Pentane/EtOAc) = 0.63.

$\underline{^1H\ NMR}$ (400 MHz, $CDCl_3$) δ 7.17 (s, 4H, Ar*H*), 6.81 – 6.73 (m, 2H, Ar*H*), 6.66 – 6.57 (m, 2H, Ar*H*), 4.09 (dd, *J* = 10.7, 7.0 Hz, 1H, O-CH_2-C), 3.97 (dd, *J* = 10.7, 6.4 Hz, 1H, O-CH_2-C), 3.89 – 3.78 (m, 1H, C*H*-CH_3), 3.75 (s, 3H, Ar-O-CH_3), 3.01 (td, *J* = 6.7, 4.3 Hz, 1H, C*H*-Ar), 2.35 (s, 3H, Ar-CH_3), 1.14 (d, *J* = 6.5 Hz, 3H, CH-CH_3). N*H* and O*H* were not resolved.

$\underline{^{13}C\{^1H\}\ NMR}$ (101 MHz, $CDCl_3$) δ 152.6, 141.6, 136.9, 136.0, 129.5, 129.0, 115.8, 115.1, 64.4, 55.9, 52.3, 51.7, 21.2, 18.9.

<u>IR</u> (cm^{-1}) 3377 (w), 2932 (w), 2361 (m), 2251 (w), 1668 (w), 1510 (s), 1235 (m), 1029 (m).

<u>HRMS</u> (APCI/QTOF) m/z: $[M + H]^+$ Calcd for $C_{18}H_{24}NO_2^+$ 286.1802; Found 286.1809.

3-((4-Methoxyphenyl)amino)-2-(4-(trifluoromethyl)phenyl)butan-1-ol (7ah)

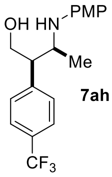

Prepared according to the general procedure E2 using **6ah** (30 mg, 72 µmol, 1.00 equiv.). The crude material was purified by column chromatography on silica gel (1:1 pentane/EtOAc) to give **7ah** (16 mg, 48 µmol, 66% yield) as a colorless oil.

$\underline{R_f}$ (1:1 Pentane/EtOAc) = 0.52.

$\underline{^1H\ NMR}$ (400 MHz, $CDCl_3$) δ 7.61 (d, *J* = 8.1 Hz, 2H, Ar*H*), 7.41 (d, *J* = 8.1 Hz, 2H, Ar*H*), 6.83 – 6.74 (m, 2H, Ar*H*), 6.68 – 6.59 (m, 2H, Ar*H*), 4.14 (dd, *J* = 10.7, 6.9 Hz, 1H, O-CH_2-C), 4.02 (dd, *J* = 10.7, 6.1 Hz, 1H, O-CH_2-C), 3.94 – 3.85 (m, 1H, C*H*-CH_3), 3.75 (s, 3H, Ar-O-CH_3), 3.14 – 3.05 (m, 1H, C*H*-Ar), 1.15 (d, *J* = 6.5 Hz, 3H, CH-CH_3). N*H* and O*H* were not resolved.

$\underline{^{13}C\{^1H\}\ NMR}$ (101 MHz, $CDCl_3$) δ 152.9, 143.7, 141.2, 129.5 (q, $J_{C\text{-}F}$ = 32.6 Hz), 129.5, 125.6 (q, $J_{C\text{-}F}$ = 3.6 Hz), 124.3 (q, $J_{C\text{-}F}$ = 272.2 Hz), 116.0, 115.1, 64.0, 55.9, 52.5, 51.9, 19.0.

$\underline{^{19}F\ NMR}$ (376 MHz, $CDCl_3$) δ -62.5.

<u>IR</u> (cm^{-1}) 3369 (w), 2928 (w), 2356 (w), 1620 (w), 1512 (s), 1327 (s), 1240 (m), 1164 (m), 1122 (s).

<u>HRMS</u> (nanochip-ESI/LTQ-Orbitrap) m/z: $[M + H]^+$ Calcd for $C_{18}H_{21}F_3NO_2^+$ 340.1519; Found 340.1513.

1-(4-Methoxyphenyl)-2-methyl-3-(4-(trifluoromethyl)phenyl)azetidine (8)

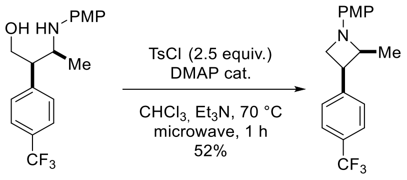

7ah 8

Following a reported procedure,[29] DMAP (1.1 mg, 8.7 µmol, 20 mol%), Et_3N (24 µL, 0.17 mmol, 4.6 equiv.) and TsCl (16 mg, 87 µmol, 2.5 equiv.) were successively added to a solution of **7ah** (12.6 mg, 0.037 mmol, 1.0 equiv.) in $CHCl_3$ (0.7 mL) and the mixture was subjected to microwave irradiation at 70 °C for 1 h. Then, brine (10 mL) was added and the organic materials were extracted with EtOAc (4 × 20 mL). The organic layers were combined, dried with Na_2SO_4, and concentrated in vacuum. The residue was purified by column chromatography on silica gel (0 – 20% (v/v) Et_2O in pentane) to obtain **8** (6.2 mg, 19 µmol, 52% yield) as a colorless oil and as a single diastereoisomer.

$\underline{R_f}$ (10% Et_2O/Pentane) = 0.36.

$\underline{^1H\ NMR}$ (400 MHz, $CDCl_3$) δ 7.63 (d, *J* = 8.2 Hz, 2H, Ar*H*), 7.56 (d, *J* = 8.1 Hz, 2H, Ar*H*), 6.89 – 6.80 (m, 2H, Ar*H*), 6.61 – 6.53 (m, 2H, Ar*H*), 4.37 (p, *J* = 6.7 Hz, 1H, C*H*-CH_3), 4.05 (d, *J* = 5.8 Hz, 1H, N-CH_2-C), 3.98 (t, *J* = 7.8 Hz, 1H, C*H*-Ar), 3.79 (d, *J* = 7.1 Hz, 1H, N-CH_2-C), 3.77 (s, 3H, Ar-O-CH_3), 1.03 (d, *J* = 6.4 Hz, 3H, CH_3).

$\underline{^{13}C\{^1H\}\ NMR}$ (201 MHz, $CDCl_3$) 152.9, 146.6, 144.2, 129.3, 129.1 (q, $J_{C\text{-}F}$ = 31.8 Hz), 125.3 (q, $J_{C\text{-}F}$ = 3.9 Hz), 114.9, 113.8, 63.5, 56.2, 56.0, 39.8, 18.4. 1C not resolved.

$\underline{^{19}F\ NMR}$ (376 MHz, $CDCl_3$) δ -62.3.

<u>HRMS</u> (ESI/QTOF) m/z: $[M + H]^+$ Calcd for $C_{18}H_{19}F_3NO^+$ 322.1413; Found 322.1418.

2-Amino-3-(*p*-tolyl)butan-1-ol (15)

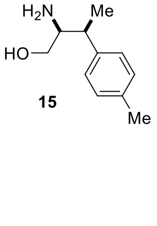

Prepared according to the general procedure E2 using **14** (8.5 mg, 33 µmol, 1.00 equiv.). The crude material was purified by preparative RP-HPLC on an Agilent 1260 HPLC system with a G2260A 1260 Prep ALS Autosampler, a G1361a 1260 Prep Pump, a G1365C 1260 MWD detector and a G1364B 1260 FC-PS collector, coupled with a Waters XBridge semi-preparative C18 column (19 ×

150 mm, 5 µm). H_2O (solvent A) and H_2O:MeCN 5:95 (solvent B), each containing 0.1% TFA, were used as the mobile phase at a flow rate of 20 mL/min^{-1}. The following method was used: 100% A to 100% B in 20 minutes. The desired product **15** was obtained as a colorless oil (4.7 mg, 26 µmol, 80%).

R_f (1:1 Pentane/EtOAc) = 0.62.

^{1}H NMR (800 MHz, CD_3CN) δ 7.19 (q, *J* = 8.2 Hz, 4H, Ar*H*), 3.90 (dd, *J* = 12.4, 3.1 Hz, 1H, O-C*H*$_2$-C), 3.75 (dd, *J* = 12.4, 6.3 Hz, 1H, O-C*H*$_2$-C), 3.39 (ddd, *J* = 9.6, 6.2, 3.2 Hz, 1H, C*H*-NH$_2$), 3.04 (dt, *J* = 9.6, 7.1 Hz, 1H, C*H*-CH$_3$), 2.32 (s, 3H, Ar-C*H*$_3$), 1.27 (d, *J* = 7.0 Hz, 3H, CH-C*H*$_3$). O*H* and N*H*$_2$ were not resolved.

^{13}C{^{1}H} NMR (201 MHz, CD_3CN) δ 139.5, 138.2, 130.7, 128.7, 60.0, 59.7, 40.0, 21.0, 18.5.

IR (cm^{-1}) 3388 (s), 2360 (s), 2341 (m), 1676 (s), 1199 (m), 1145 (m).

HRMS (ESI/QTOF) m/z: [M + H]$^+$ Calcd for $C_{11}H_{18}NO^+$ 180.1383; Found 180.1385.

E.3. Electrophilic fluorination

3-Fluoro-4-hydroxy-3-(*p*-tolyl)butan-2-one (10)

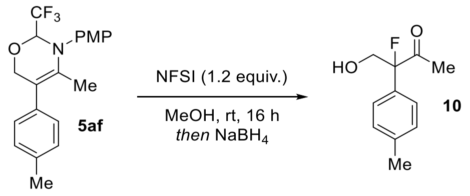

An oven-dried 10 mL microwave vial equipped with a Teflon coated stirring bar was charged with **5af** (36 mg, 0.10 mmol, 1.00 equiv.) and NFSI (38 mg, 0.12 mmol, 1.20 equiv.). The vial was then sealed, evacuated and back-filled with N_2 three times and MeOH (1.0 mL) was added. The resulting mixture was stirred at room temperature for 16 h. Then, the reaction mixture was quenched with $NaBH_4$ (1.5 equiv.) and the reaction was stirred at room temperature for 15 min (no ketone reduction was observed). Then, 1 M HCl (5 mL) was added, and the mixture was extracted with DCM (3 × 5 mL), dried with Na_2SO_4 and concentrated in vacuo. The crude material was purified by column chromatography on silica gel (0 – 30% (v/v) Et_2O in pentane) to afford the product **10** (16 mg, 81 µmol, 81% yield) as a pink oil.

R_f (1:1 Pentane/Et_2O) = 0.52.

^{1}H NMR (400 MHz, $CDCl_3$) δ 7.36 – 7.29 (m, 2H, Ar*H*), 7.21 (d, *J* = 8.4 Hz, 2H, Ar*H*), 4.28 (ddd, *J* = 29.4, 12.6, 5.5 Hz, 1H, O-C*H*$_2$-C), 3.89 (ap t, *J* = 14.3 Hz, 1H, O-C*H*$_2$-C), 2.35 (s, 3H, Ar-C*H*$_3$), 2.26 (d, *J* = 5.2 Hz, 3H, C*H*$_3$), 2.06 (br s, 1H, O*H*).

^{13}C{^{1}H} NMR (101 MHz, $CDCl_3$) δ 207.0 (d, J_{C-F} = 30.9 Hz), 139.0, 131.3 (d, J_{C-F} = 22.0 Hz), 129.6 (d, J_{C-F} = 1.6 Hz), 124.5 (d, J_{C-F} = 9.5 Hz), 102.8 (d, J_{C-F} = 188.2 Hz), 67.2 (d, J_{C-F} = 22.6 Hz), 26.1, 21.2.

^{19}F NMR (376 MHz, $CDCl_3$) δ -172.6 (ddd, *J* = 29.3, 16.8, 5.2 Hz).

IR (cm^{-1}) 3437 (w), 2921 (w), 2360 (m), 2247 (w), 1722 (m), 1361 (w), 1062 (m), 910 (s).

HRMS (APCI/QTOF) m/z: [M + Na]$^+$ Calcd for $C_{11}H_{13}FNaO_2^+$ 219.0792; Found 219.0788.

5-Fluoro-3-(4-methoxyphenyl)-4-methyl-5-(*p*-tolyl)-2-(trifluoromethyl)-1,3-oxazinane (11)

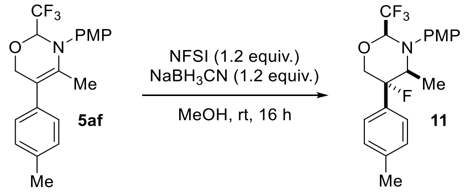

An oven-dried 10 mL microwave vial equipped with a Teflon coated stirring bar was charged with **5af** (110 mg, 0.303 mmol, 1.00 equiv.), NFSI (114 mg, 0.360 mmol, 1.20 equiv.) and $NaBH_3CN$ (23 mg, 0.36 mmol, 1.20 equiv.). The vial was then sealed, evacuated and back-filled with N_2 three times and MeOH (3.0 mL) was added. The resulting mixture was stirred at room temperature for 16 h. Then, brine (5 mL) was added, and the mixture was extracted with EtOAc (3 × 5 mL), dried with Na_2SO_4 and concentrated in vacuo. The crude material was purified by column chromatography on silica gel (0 – 30% (v/v) DCM in pentane) to afford the product **11** (17:1 *dr*, 90.3 mg, 0.298 mmol, 98% yield) as a colorless oil. The diastereomeric ratio was determined by integration of ^{19}F NMR spectra and was found unchanged before and after purification.

R_f (10% Et_2O/Pentane) = 0.51.

Major diastereoisomer:

^{1}H NMR (400 MHz, $CDCl_3$) δ 7.51 (d, *J* = 8.3 Hz, 2H, Ar*H*), 7.21 (d, *J* = 8.2 Hz, 2H, Ar*H*), 7.18 – 7.12 (m, 2H, Ar*H*), 6.89 – 6.79 (m, 2H, Ar*H*), 5.12 (q, *J* = 5.1 Hz, 1H, CF$_3$-C*H*), 4.55 (dd, *J* = 20.0, 12.2 Hz, 1H, O-C*H*$_2$-C), 4.08

(dd, J = 14.9, 12.2 Hz, 1H, O-CH_2-C), 3.79 (s, 3H, Ar-O-CH_3), 3.65 (dq, J = 13.9, 6.9 Hz, 1H, CH-CH$_3$), 2.37 (s, 3H, Ar-CH_3), 0.76 (d, J = 6.9 Hz, 3H, CH-CH_3).

$^{13}C\{^1H\}$ NMR (101 MHz, CDCl$_3$) δ 157.6, 139.9, 138.1, 135.5 (d, $J_{C\text{-}F}$ = 22.5 Hz), 129.0 (d, $J_{C\text{-}F}$ = 1.3 Hz), 127.3, 125.5 (d, $J_{C\text{-}F}$ = 9.9 Hz), 123.0 (q, $J_{C\text{-}F}$ = 286.2 Hz), 114.5, 93.8 (d, $J_{C\text{-}F}$ = 177.2 Hz), 86.7 (q, $J_{C\text{-}F}$ = 31.6 Hz), 70.6 (d, $J_{C\text{-}F}$ = 28.2 Hz), 62.3 (d, $J_{C\text{-}F}$ = 28.2 Hz), 55.6, 21.2, 16.2.

^{19}F NMR (376 MHz, CDCl$_3$) δ -75.1 (d, J = 5.0 Hz), -152.7 (q, J = 15.0 Hz).

IR (cm^{-1}) 2986 (w), 2942 (w), 2838 (w), 2362 (w), 1512 (s), 1462 (w), 1273 (m), 1242 (s), 1166 (s), 1044 (m).

HRMS (APCI/QTOF) m/z: [M + H]$^+$ Calcd for $C_{20}H_{22}F_4NO_2^+$ 384.1581; Found 384.1578.

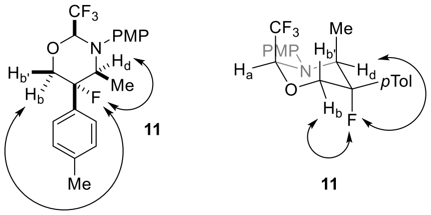

^1H-^{19}F HOESY (1D), set o2p on resonance with ^{19}F peak -152.7 ppm (F) to irradiate, 90 pulse

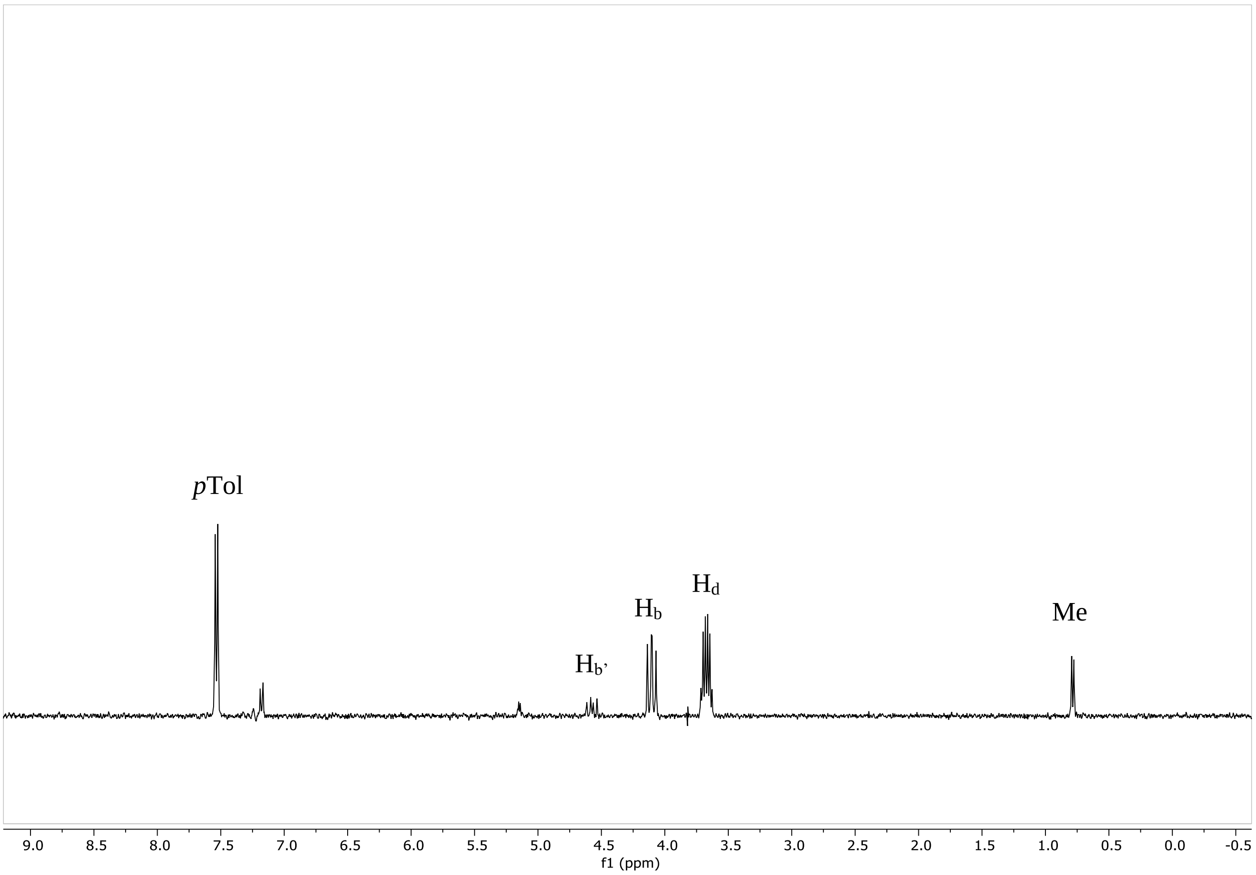

2-Fluoro-3-((4-methoxyphenyl)amino)-2-(*p*-tolyl)butan-1-ol (12)

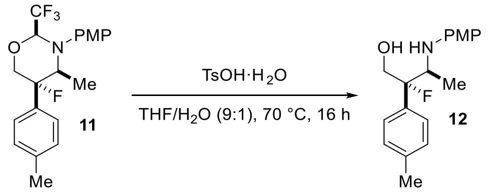

To a solution of **11** (47 mg, 0.12 mmol, 1.00 equiv.) in THF/H_2O (9:1), was added TsOH·H_2O (164 mg, 0.860 mmol, 7.00 equiv.). The reaction was stirred at 70 ºC for 16 h. Then the mixture was diluted with DCM (20 mL). The organic layer was washed with an aqueous 1 M NaOH solution (2 × 10 mL), dried over Na_2SO_4, filtered and concentrated under reduced pressure. The residue was purified by column chromatography on silica gel (10 – 40% (v/v) Et_2O in pentane) to afford **12** (22:1 *dr*, 27 mg, 88 µmol, 72% yield) as a colorless oil. The diastereomeric ratio was determined by integration of ^{19}F NMR spectra and was found unchanged before and after purification.

R_f (1:1 pentane/Et_2O) = 0.52.

Major diastereoisomer:

<u>^{1}H NMR</u> (400 MHz, $CDCl_3$) δ 7.30 (d, J = 8.2 Hz, 2H, ArH), 7.22 (d, J = 8.0 Hz, 2H, ArH), 6.85 – 6.71 (m, 2H, ArH), 6.70 – 6.62 (m, 2H, ArH), 4.18 (dd, J = 24.4, 12.3 Hz, 1H, O-CH_2-C), 4.11 – 4.04 (m, 1H, CH-CH_3), 3.99 (dd, J = 19.3, 12.3 Hz, 1H, O-CH_2-C), 3.75 (s, 3H, Ar-O-CH_3), 2.38 (s, 3H, Ar-CH_3), 1.08 (dd, J = 6.5, 1.8 Hz, 3H, CH-CH_3). NH and OH were not resolved.

<u>^{13}C{^{1}H} NMR</u> (101 MHz, $CDCl_3$) δ 152.7, 141.2, 138.0, 134.3 (d, J_{C-F} = 21.6 Hz), 129.1 (d, J_{C-F} = 1.4 Hz), 125.7 (d, J_{C-F} = 9.9 Hz), 115.7, 115.1, 100.6 (d, J_{C-F} = 178.7 Hz), 66.0 (d, J_{C-F} = 22.0 Hz), 55.9, 53.0 (d, J_{C-F} = 30.0 Hz), 21.2, 15.7 (d, J_{C-F} = 2.7 Hz).

<u>^{19}F NMR</u> (376 MHz, $CDCl_3$) δ -165.5 – -170.3 (m).

<u>IR</u> (cm^{-1}) 3411 (w), 2939 (w), 2360 (w), 2247 (w), 1512 (s), 1235 (m), 1040 (m), 910 (s).

<u>HRMS</u> (APCI/QTOF) m/z: $[M + H]^+$ Calcd for $C_{18}H_{23}FNO_2^+$ 304.1707; Found 304.1706.

F. Proposed reaction mechanism

We hypothesize that the reaction of the propargylic alcohol **1** with the aldimine or imine tether **3**, either with PMP or Ts substituents proceeds first, giving hemiaminal intermediate **T**. Then, intermediate **T** can react with complex **II**, generated by oxidative addition of the Pd(0) catalyst **I** on the bromoalkyne or aryl iodide.

The observed products **4** and **5** would be most probably formed by reductive elimination from a vinyl-Pd^{II} intermediate **IVa** for PMP and **IVb** for Ts. The simplest explanation would be therefore that a direct *anti*-aminopalladation step is occuring either in a 6-endo or 5-exo fashion, depending on the substitutent on the nitrogen. Such an outcome may be rationalized by a steric argument, as there is potentially less steric interference between the nitrogen substituent and the alkyne substituent in the vinyl-Pd species **IVa** (and the transition state leading to it). However, *syn*-aminopalladation/isomerization pathways could also be suggested. Due to geomtetrical constraints, only the 5-exo *syn*-palladation product **IVc** could be formed. *Cis*/*trans* isomerization to intermediate **IVb** is a known process. In the case of PMP, a dyotropic rearrangement to give **IVa** from **IVb-PMP** would be needed in addition. This scenario seems less probable, as dyotropic rearrangements with Pd normally take place only at higher oxidation states. For the case of the more acidic and less bulky NHTs, Pd-N coordination facilitated by deprotonation is more likely, generating **V**, which would be more prone to undergo *syn*-palladation. For the less acidic NHPMP, such formation of a covalent bond with Pd would be more difficult. Therefore, a second speculative explanation of the experimental results would be that the reaction follow two different mechanism pathways in dependence of the protecting group on nitrogen: deprotonation-coordination, *syn*-palladation, *cis*/*trans* isomerization and reductive elimination in case of Ts, and *anti*-palladation, deprotonation, reductive elimination in the case of PMP.

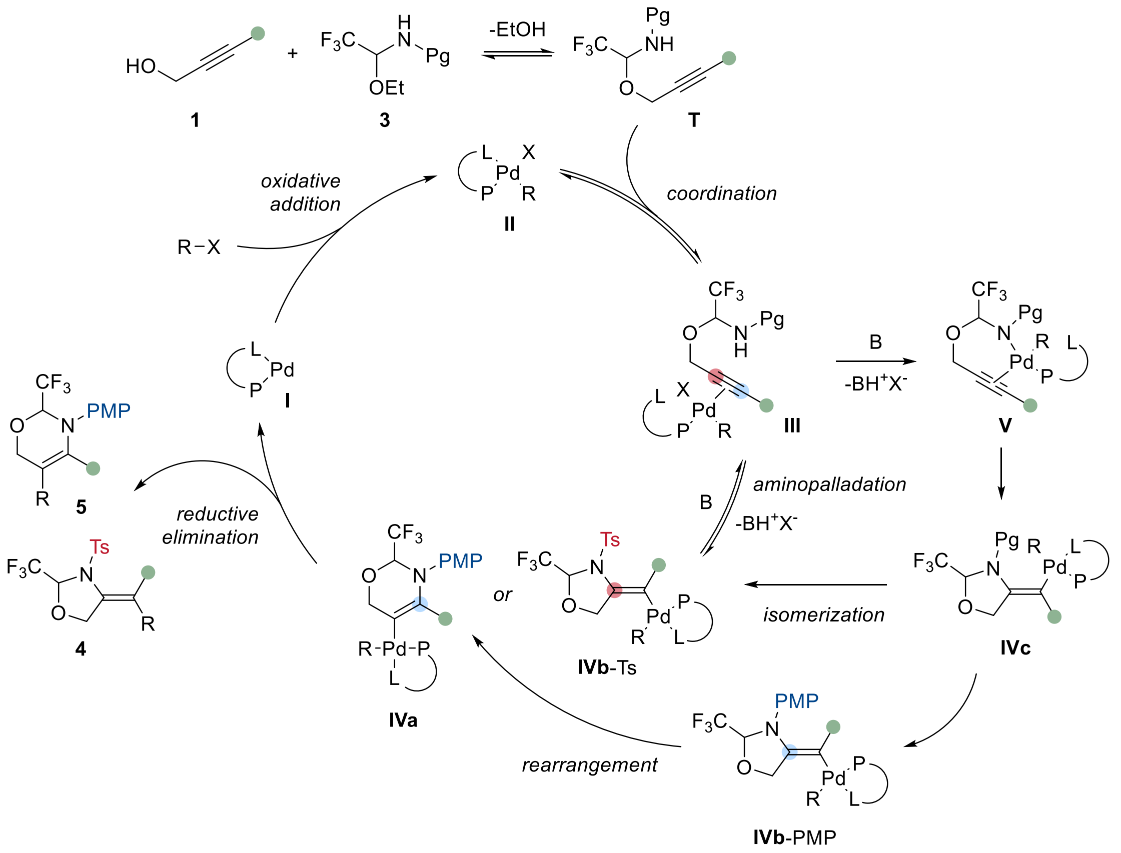

G. X-ray crystallographic data

3-(4-Methoxyphenyl)-4-methyl-5-(p-tolyl)-2-(trifluoromethyl)-3,6-dihydro-2*H*-1,3-oxazine (5af)

CCDC deposition Number 2356174

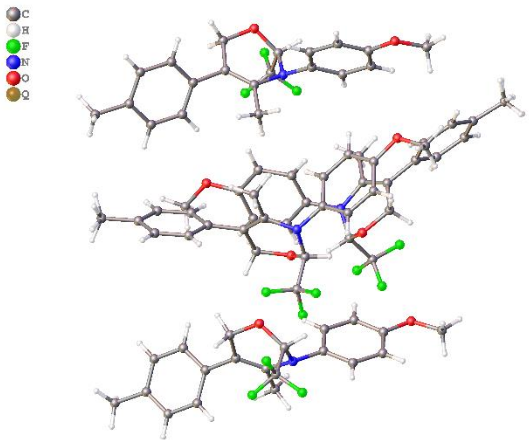

Crystals of compound **5af** were collected upon crystallization via vapour diffusion with Et_2O/pentane at 5 °C.

Experimental. Single clear pale colourless irregular-shaped crystals of **5af** were used as supplied. A suitable crystal with dimensions 0.53 × 0.39 × 0.32 mm^3 was selected and mounted on a SuperNova, Dual, Cu at home/near, AtlasS2 diffractometer. The crystal was kept at a steady T = 139.99(10) K during data collection. The structure was solved with the ShelXT (Sheldrick, 2015) solution program using dual methods and by using Olex2 1.5 (Dolomanov et al., 2009) as the graphical interface. The model was refined with ShelXL 2019/3 (Sheldrick, 2015) using full matrix least squares minimisation on $\boldsymbol{F}^2$.

Crystal Data. $C_{20}H_{20}F_3NO_2$, M_r = 363.37, triclinic, P-1 (No. 2), a = 15.1531(3) Å, b = 15.7015(3) Å, c = 17.3907(3) Å, α = 110.6290(17)°, β = 109.3152(17)°, γ = 91.1167(15)°, V = 3610.83(13) $Å^3$, T = 139.99(10) K, Z = 8, Z' = 4, μ(Cu K_α) = 0.905, 43380 reflections measured, 14873 unique (R_{int} = 0.0284) which were used in all calculations. The final wR_2 was 0.1095 (all data) and R_1 was 0.0401 (I≥2 σ(I)).

Compound	5af
Formula	$C_{20}H_{20}F_3NO_2$
$D_{calc.}$/ g cm^{-3}	1.337
μ/mm^{-1}	0.905
Formula Weight	363.37
Colour	clear pale colourless
Shape	irregular
Size/mm^3	0.53×0.39×0.32
T/K	139.99(10)
Crystal System	triclinic
Space Group	P-1
a/Å	15.1531(3)
b/Å	15.7015(3)
c/Å	17.3907(3)
α/°	110.6290(17)
β/°	109.3152(17)
γ/°	91.1167(15)
V/Å^3	3610.83(13)
Z	8
Z'	4
Wavelength/Å	1.54184
Radiation type	Cu K_α
Θ_{min}/°	3.044
Θ_{max}/°	76.015
Measured Refl's.	43380
Indep't Refl's	14873
Refl's I≥2 σ(I)	12971
R_{int}	0.0284
Parameters	958
Restraints	0
Largest Peak	0.235
Deepest Hole	-0.246
GooF	1.019
wR_2 (all data)	0.1095
wR_2	0.1046
R_1 (all data)	0.0459
R_1	0.0401

Structure Quality Indicators

**Reflections:**	d min (CuKα) 2Θ=152.0° 0.79	I/σ(I) 36.9	Rint m=2.92 2.84%	Full 135.4° 99% to 152.0° 99.7
**Refinement:**	Shift 0.001	Max Peak 0.2	Min Peak -0.2	GooF 1.019

A clear pale colourless irregular-shaped crystal with dimensions 0.53 × 0.39 × 0.32 mm^3 was mounted. Data were collected using a SuperNova, Dual, Cu at home/near, AtlasS2 diffractometer operating at T = 139.99(10) K.

Data were measured using ω scans with Cu K_α radiation. The diffraction pattern was indexed and the total number of runs and images was based on the strategy calculation from the program CrysAlisPro system (CCD 43.115a 64-bit (release 15-03-2024)). The maximum resolution that was achieved was Θ = 76.015° (0.79 Å).

The unit cell was refined using CrysAlisPro 1.171.43.116a (Rigaku OD, 2024) on 23916 reflections, 55% of the observed reflections.

Data reduction, scaling and absorption corrections were performed using CrysAlisPro 1.171.43.116a

(Rigaku OD, 2024). The final completeness is 99.70 % out to 76.015° in Θ. A gaussian absorption correction was performed using CrysAlisPro 1.171.43.116a (Rigaku Oxford Diffraction, 2024). The numerical absorption correction was based on gaussian integration over a multifaceted crystal model. The empirical absorption correction was done using spherical harmonics, implemented in SCALE3 ABSPACK scaling algorithm. The absorption coefficient μ of this crystal is 0.905 mm^{-1} at this wavelength (λ = 1.54184Å) and the minimum and maximum transmissions are 0.187 and 1.000.

The structure was solved and the space group *P*-1 (# 2) determined by the ShelXT (Sheldrick, 2015) structure solution program using dual methods and refined by full matrix least squares minimisation on $\boldsymbol{F^2}$ using version 2019/3 of ShelXL (Sheldrick, 2015). All non-hydrogen atoms were refined anisotropically. Hydrogen atom positions were calculated geometrically and refined using the riding model.

The value of Z' is 4. The moiety formula is C20 H20 F3 N O2.

Data Plots: Diffraction Data

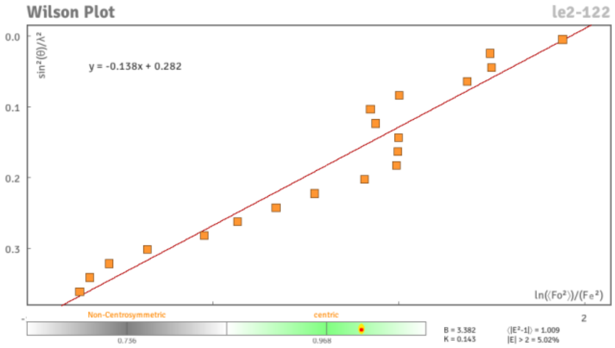

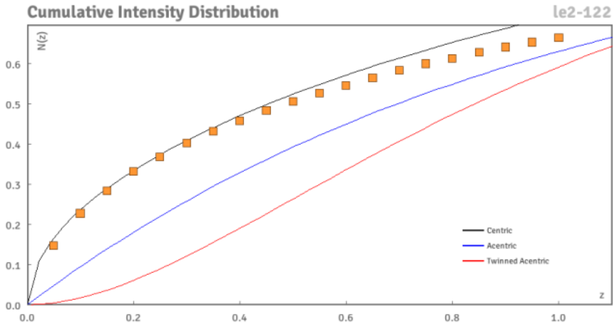

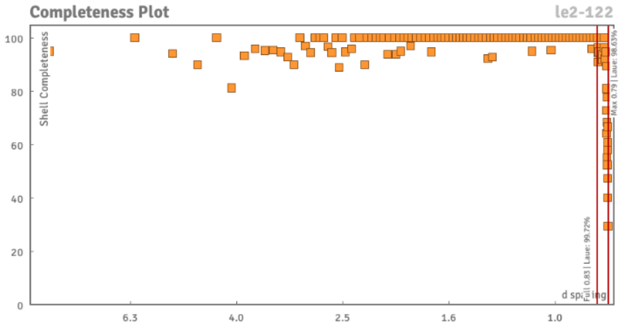

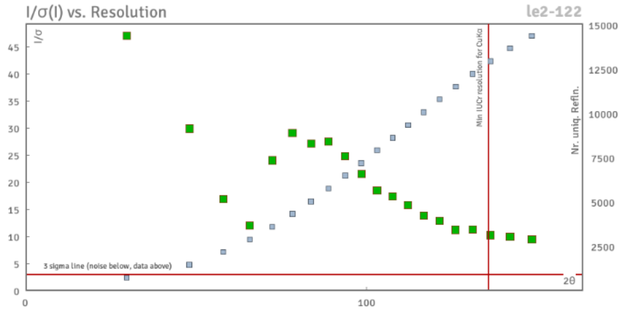

Data Plots: Refinement and Data

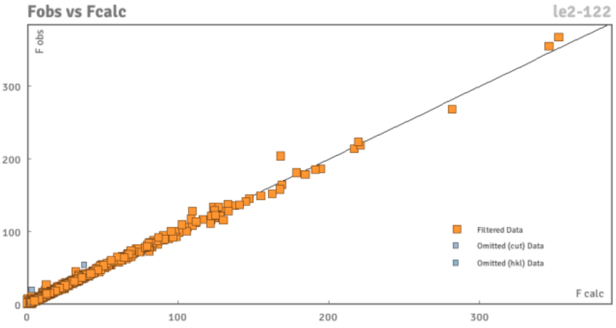

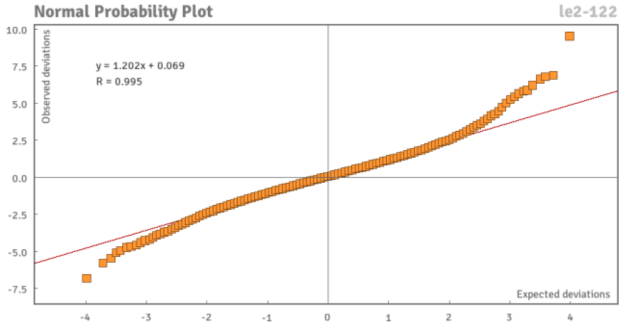

Reflection Statistics

Total reflections (after filtering)	43380	Unique reflections	14873
Completeness	0.986	Mean I/σ	19.54
hkl_{max} collected	(16, 18, 21)	hkl_{min} collected	(-19, -19, -20)

hkl_{max} used	(17, 18, 21)	hkl_{min} used	(-19, -19, 0)
Lim d_{max} collected	100.0	Lim d_{min} collected	0.77
d_{max} used	14.52	d_{min} used	0.79
Friedel pairs	4585	Friedel pairs merged	1
Inconsistent equivalents	33	R_{int}	0.0284
R_{sigma}	0.0271	Intensity transformed	0
Omitted reflections	2	Omitted by user (OMIT hkl)	0
Multiplicity	(8684, 4900, 2728, 1490, 769, 360, 166, 97, 89, 65, 42, 31, 25, 10, 3, 1)	Maximum multiplicity	16
Removed systematic absences	0	Filtered off (Shel/OMIT)	0

Table S1: Fractional Atomic Coordinates (×10[4]) and Equivalent Isotropic Displacement Parameters (Å[2]×10[3]) for **5af**. U_{eq} is defined as 1/3 of the trace of the orthogonalised U_{ij}.

Atom	x	y	z	U_{eq}
F1	4474.4(6)	2428.8(6)	4252.8(6)	47.28(19)
F2	5446.1(7)	3443.1(8)	5446.6(6)	66.1(3)
F3	5048.5(7)	3681.1(6)	4241.2(7)	56.4(2)
O1	6484.3(6)	2103.8(6)	4911.2(6)	39.08(19)
O2	8722.0(7)	3120.3(7)	2384.0(7)	45.0(2)
N1	5895.9(8)	2161.5(7)	3473.8(7)	36.0(2)
C1	4825.5(9)	-113.4(9)	3343.3(8)	35.1(2)
C2	5226.1(9)	-817.9(9)	3576.3(9)	39.1(3)
C3	4680.7(9)	-1647.6(9)	3359.9(9)	39.5(3)
C4	3710.4(9)	-1805.0(9)	2899.3(8)	38.2(3)
C5	3311.3(9)	-1109.4(10)	2654.5(10)	44.0(3)
C6	3852.2(9)	-274.4(10)	2873.2(9)	42.5(3)
C7	5410.0(9)	794.2(9)	3640.6(8)	35.2(2)
C8	5453.9(9)	1224.8(8)	3112.6(8)	35.1(2)
C9	6599.2(9)	2393.6(9)	3162.6(8)	36.3(2)
C10	6677.7(10)	3247.6(9)	3104.2(9)	42.0(3)
C11	7376.7(11)	3520.6(9)	2846.3(9)	42.6(3)
C12	8001.2(9)	2925.3(9)	2638.7(8)	38.3(3)
C13	7921.9(10)	2063.4(9)	2687.1(9)	41.1(3)
C14	7227.9(10)	1801.3(9)	2949.5(9)	39.3(3)
C15	3110.9(10)	-2691.7(10)	2684.6(11)	47.8(3)
C16	5908.7(9)	1237.9(9)	4627.3(8)	38.7(3)
C17	6118.0(9)	2636.2(9)	4408.4(8)	36.0(2)
C18	5259.9(10)	3037.6(9)	4581.3(9)	41.0(3)
C19	4993.9(10)	826.0(9)	2125.1(8)	39.8(3)
C20	8842.0(14)	4003.5(11)	2352.6(13)	59.2(4)
F4	1010.6(6)	-82.0(6)	7945.3(6)	51.9(2)
F5	614.7(7)	1186.8(8)	7845.2(7)	64.0(3)
F6	1725.5(7)	623.5(7)	7418.8(6)	59.6(2)
O3	1671.4(7)	1647.0(6)	9589.2(6)	37.96(19)
O4	6469.1(7)	2740.8(7)	10243.1(7)	44.5(2)
N2	2855.8(7)	814.8(7)	9170.6(7)	35.5(2)
C21	1660.7(9)	-498.9(9)	10103.2(8)	35.3(2)
C22	1515.6(9)	-276.9(9)	10898.8(9)	38.3(3)
C23	1153.1(9)	-952.1(10)	11105.7(9)	41.9(3)
C24	925.7(9)	-1869.4(10)	10532.1(10)	42.2(3)
C25	1068.1(10)	-2088.2(10)	9740.5(10)	44.3(3)
C26	1425.5(10)	-1418.8(9)	9523.7(9)	41.9(3)
C27	1986.8(9)	226.8(8)	9852.4(8)	35.0(2)
C28	2723.4(9)	213.3(8)	9585.4(8)	34.8(2)
C29	2092.5(9)	1319.9(8)	8949.8(8)	35.4(2)
C30	1353.5(10)	752.9(10)	8041.0(9)	41.0(3)
C31	541.4(12)	-2603.7(12)	10759.8(13)	55.6(4)
C32	1350.9(10)	947.9(9)	9829.3(9)	38.6(3)
C33	3423.4(9)	-445.3(9)	9639.4(9)	39.3(3)
C34	3786.0(9)	1303.6(8)	9439.2(8)	34.9(2)
C35	4118.5(10)	1375.1(10)	8806.8(9)	40.7(3)

Atom	x	y	z	U_{eq}
C36	5008.2(10)	1856.6(10)	9046.9(9)	41.7(3)
C37	5578.5(9)	2262.3(8)	9931.1(9)	37.3(2)
C38	5250.3(9)	2190.7(9)	10570.6(9)	38.7(3)
C39	4357.8(9)	1725.1(9)	10324.4(8)	37.1(2)
C40	6790.2(12)	2901.4(14)	9618.3(13)	61.6(4)
F7	-43.4(7)	5951.1(6)	2842.4(6)	55.3(2)
F8	1305.1(9)	5594.4(7)	2808.4(8)	71.1(3)
F9	759.5(8)	6676.0(7)	2396.2(6)	61.5(3)
O5	1638.9(6)	6595.0(7)	4542.0(6)	40.1(2)
O6	2724.2(6)	11344.4(6)	5391.7(6)	39.66(19)
N3	836.5(7)	7756.2(7)	4142.9(7)	36.9(2)
C41	-698.2(9)	6534.5(9)	4886.4(9)	37.0(2)
C42	-1617.9(10)	6297.1(10)	4267.2(10)	44.9(3)
C43	-2361.7(10)	5959.5(10)	4435.2(11)	49.4(3)
C44	-2210.3(11)	5838.8(9)	5219.7(11)	46.2(3)
C45	-1291.9(11)	6056.3(10)	5826.4(10)	47.9(3)
C46	-546.8(10)	6397.0(10)	5664.8(9)	43.4(3)
C47	107.6(9)	6868.3(9)	4703.6(8)	36.2(2)
C48	147.4(9)	7605.7(9)	4490.7(9)	37.0(2)
C49	1366.7(9)	7015.7(9)	3923.3(8)	36.8(2)
C50	832.7(11)	6303.5(10)	2989.5(10)	45.3(3)
C51	-3005.3(13)	5485.3(12)	5422.5(14)	63.1(5)
C52	864.8(9)	6264.6(9)	4715.4(9)	40.2(3)
C53	-526.8(9)	8293.3(9)	4537.8(11)	44.7(3)
C54	1329.2(8)	8672.9(8)	4456.1(8)	35.0(2)
C55	1635.0(9)	9250.0(9)	5343.6(9)	36.9(2)
C56	2093.6(9)	10134.8(9)	5628.8(8)	36.9(2)
C57	2268.6(8)	10455.5(8)	5032.9(9)	34.9(2)
C58	1984.9(10)	9881.6(9)	4150.1(9)	40.6(3)
C59	1509.3(10)	8995.2(9)	3867.1(9)	40.4(3)
C60	2997.2(13)	11684.0(11)	4833.3(12)	55.7(4)
F10	2513.1(8)	5386.8(6)	667.5(8)	63.5(3)
F11	1531.6(8)	4412.1(8)	-548.4(7)	66.9(3)
F12	1218.2(8)	4775.5(7)	632.9(8)	65.9(3)
O7	2938.9(7)	3456.4(6)	-3.4(6)	41.2(2)
O8	1728.9(8)	1564.6(7)	2742.6(7)	47.3(2)
N4	2816.0(8)	4011.3(8)	1418.6(7)	40.3(2)
C61	5144.9(10)	5153.2(9)	1606.5(8)	39.1(3)
C62	5923.3(11)	4817.3(9)	1418.5(9)	43.9(3)
C63	6745.5(11)	5403.9(10)	1650.5(10)	46.1(3)
C64	6824.1(10)	6349.2(10)	2076.6(9)	42.5(3)
C65	6055.6(11)	6686.4(10)	2280.8(11)	50.3(3)
C66	5229.5(11)	6104.9(10)	2044.4(11)	48.7(3)
C67	4234.9(10)	4536.8(9)	1288.9(8)	39.4(3)
C68	3763.8(10)	4473.9(9)	1801.8(9)	40.5(3)
C69	2534.9(10)	3383.3(9)	1754.4(8)	38.6(3)
C70	1613.3(10)	3289.4(10)	1734.2(9)	43.4(3)
C71	1307.5(10)	2677.4(10)	2041.0(9)	43.8(3)
C72	1942.3(10)	2171.4(9)	2400.3(8)	39.9(3)
C73	2865.8(10)	2259.1(9)	2421.8(9)	40.8(3)
C74	3158.0(10)	2855.2(9)	2093.7(8)	39.3(3)
C75	7700.1(12)	6987.9(12)	2280.8(10)	53.2(4)
C76	3820.6(10)	4044.6(9)	305.0(9)	41.8(3)
C77	2356.0(10)	3787.9(9)	488.8(8)	39.6(3)
C78	1914.6(11)	4601.0(10)	315.8(10)	47.4(3)
C79	4124.5(12)	4941.2(10)	2789.4(9)	48.6(3)
C80	766.0(12)	1376.5(12)	2641.4(12)	56.4(4)

Table S2: Anisotropic Displacement Parameters (×10[4]) for **5af.** The anisotropic displacement factor exponent

takes the form: $-2\pi^2[h^2a^{*2} \times U_{11} + ... + 2hka^* \times b^* \times U_{12}]$

Atom	U_{11}	U_{22}	U_{33}	U_{23}	U_{13}	U_{12}
F1	38.6(4)	51.0(4)	49.1(4)	13.9(4)	17.8(3)	3.0(3)
F2	59.7(5)	81.6(7)	35.7(4)	-3.7(4)	19.0(4)	14.9(5)
F3	52.8(5)	46.6(4)	74.8(6)	24.8(4)	27.0(4)	15.6(4)
O1	38.5(4)	41.7(5)	30.0(4)	11.8(4)	6.2(3)	0.0(4)
O2	50.6(5)	44.2(5)	42.1(5)	14.9(4)	20.9(4)	0.1(4)
N1	42.4(5)	34.3(5)	29.0(5)	9.0(4)	13.6(4)	2.2(4)
C1	36.7(6)	37.4(6)	29.9(5)	11.5(5)	12.1(5)	4.3(5)
C2	33.8(6)	42.7(6)	38.3(6)	16.2(5)	9.3(5)	4.2(5)
C3	40.8(6)	39.7(6)	39.7(6)	17.4(5)	14.1(5)	7.2(5)
C4	39.2(6)	39.4(6)	33.9(6)	9.5(5)	15.8(5)	2.3(5)
C5	33.4(6)	45.4(7)	45.7(7)	14.0(6)	8.8(5)	3.6(5)
C6	37.9(6)	41.5(7)	43.6(7)	16.7(5)	8.5(5)	7.3(5)
C7	36.2(6)	37.0(6)	29.8(5)	10.9(5)	10.5(5)	5.0(5)
C8	38.9(6)	33.5(6)	30.3(6)	8.9(5)	12.6(5)	4.4(5)
C9	42.3(6)	35.7(6)	29.5(5)	10.7(5)	13.0(5)	3.4(5)
C10	52.4(7)	34.8(6)	42.1(7)	13.8(5)	21.9(6)	9.5(5)
C11	55.7(8)	33.7(6)	40.2(6)	13.8(5)	19.9(6)	3.8(5)
C12	44.1(6)	38.7(6)	29.1(5)	10.6(5)	12.3(5)	0.0(5)
C13	45.3(7)	40.3(6)	40.2(6)	15.6(5)	18.2(5)	8.8(5)
C14	46.4(7)	35.8(6)	38.3(6)	16.7(5)	15.7(5)	7.5(5)
C15	44.9(7)	45.2(7)	52.1(8)	14.9(6)	20.2(6)	-0.5(6)
C16	42.0(6)	40.7(6)	30.2(6)	12.7(5)	10.1(5)	1.7(5)
C17	37.4(6)	36.8(6)	29.3(5)	8.7(5)	10.9(5)	3.0(5)
C18	41.9(6)	41.1(6)	33.5(6)	6.8(5)	13.7(5)	4.5(5)
C19	47.6(7)	39.0(6)	29.3(6)	10.2(5)	12.7(5)	3.3(5)
C20	74.9(11)	48.2(8)	67.3(10)	24.1(7)	39.6(9)	1.7(7)
F4	52.8(5)	48.6(4)	43.2(4)	14.2(4)	8.2(4)	-7.9(4)
F5	56.0(5)	74.1(6)	50.3(5)	24.7(5)	3.1(4)	22.2(5)
F6	63.0(5)	76.9(6)	34.2(4)	14.5(4)	19.4(4)	-5.0(5)
O3	47.0(5)	33.9(4)	38.0(4)	16.1(4)	18.5(4)	11.7(4)
O4	40.1(5)	43.2(5)	51.9(5)	19.0(4)	17.8(4)	4.2(4)
N2	38.0(5)	35.2(5)	37.2(5)	17.1(4)	14.7(4)	7.4(4)
C21	34.4(5)	39.4(6)	34.8(6)	17.4(5)	12.3(5)	8.6(5)
C22	38.6(6)	41.9(6)	36.0(6)	15.9(5)	14.2(5)	8.4(5)
C23	39.1(6)	54.9(8)	40.3(6)	25.1(6)	17.2(5)	11.1(6)
C24	34.1(6)	51.5(7)	48.9(7)	30.3(6)	12.6(5)	9.3(5)
C25	50.3(7)	38.1(6)	43.8(7)	17.8(5)	13.9(6)	4.8(5)
C26	52.7(7)	40.0(6)	36.5(6)	16.7(5)	18.0(6)	7.7(6)
C27	39.1(6)	34.5(6)	32.5(6)	13.8(5)	13.1(5)	7.7(5)
C28	38.7(6)	32.4(5)	34.0(6)	14.0(5)	12.4(5)	5.8(5)
C29	39.7(6)	34.4(6)	33.7(6)	14.9(5)	13.1(5)	6.8(5)
C30	42.2(6)	45.6(7)	35.9(6)	17.3(5)	13.0(5)	5.3(5)
C31	51.8(8)	61.5(9)	68.0(10)	41.4(8)	21.5(7)	7.0(7)
C32	45.0(6)	38.3(6)	40.4(6)	18.9(5)	20.7(5)	12.4(5)
C33	39.3(6)	37.3(6)	46.0(7)	19.3(5)	17.2(5)	10.1(5)
C34	38.3(6)	33.8(6)	36.0(6)	14.8(5)	15.8(5)	7.9(5)
C35	44.1(7)	46.7(7)	32.6(6)	15.8(5)	14.7(5)	6.2(5)
C36	45.6(7)	47.0(7)	39.9(6)	19.8(6)	20.9(6)	8.0(5)
C37	38.8(6)	32.7(6)	43.9(7)	16.4(5)	17.2(5)	8.5(5)
C38	43.7(6)	35.2(6)	35.0(6)	11.7(5)	13.1(5)	6.4(5)
C39	44.3(6)	37.3(6)	33.4(6)	14.8(5)	17.3(5)	8.3(5)
C40	43.9(8)	82.7(12)	72.8(11)	45.6(10)	22.3(7)	2.0(8)
F7	55.9(5)	53.9(5)	44.0(4)	9.2(4)	14.8(4)	-16.3(4)
F8	86.0(7)	53.4(5)	62.5(6)	2.2(5)	33.8(6)	17.2(5)
F9	75.8(6)	64.6(6)	38.5(4)	17.9(4)	17.0(4)	-15.8(5)
O5	35.3(4)	45.2(5)	45.8(5)	21.6(4)	17.2(4)	9.9(4)
O6	39.6(4)	36.0(4)	45.4(5)	17.3(4)	16.0(4)	2.8(4)
N3	34.5(5)	35.5(5)	42.1(6)	15.5(4)	14.8(4)	3.6(4)
C41	37.6(6)	34.3(6)	40.2(6)	12.6(5)	16.9(5)	5.6(5)

Atom	U_{11}	U_{22}	U_{33}	U_{23}	U_{13}	U_{12}
C42	40.0(6)	49.0(7)	45.8(7)	20.6(6)	13.1(6)	2.4(6)
C43	38.5(7)	45.8(7)	59.7(9)	16.3(6)	17.1(6)	0.9(6)
C44	51.2(7)	32.3(6)	60.2(8)	11.1(6)	33.3(7)	4.9(5)
C45	58.7(8)	45.6(7)	46.9(7)	15.9(6)	30.3(7)	7.4(6)
C46	45.1(7)	46.9(7)	38.8(7)	14.7(5)	17.7(5)	5.2(6)
C47	34.4(6)	38.6(6)	36.3(6)	14.3(5)	13.4(5)	4.9(5)
C48	31.4(5)	37.2(6)	39.8(6)	12.7(5)	12.0(5)	3.2(5)
C49	37.1(6)	37.3(6)	37.3(6)	13.5(5)	15.5(5)	4.3(5)
C50	53.4(8)	41.5(7)	41.4(7)	12.3(6)	21.7(6)	-0.5(6)
C51	65.7(10)	48.8(8)	85.2(13)	17.1(8)	49.4(10)	2.0(7)
C52	40.3(6)	44.0(7)	45.0(7)	22.4(6)	19.8(5)	10.0(5)
C53	34.1(6)	38.7(6)	61.6(8)	18.9(6)	17.6(6)	6.1(5)
C54	33.1(5)	35.6(6)	38.5(6)	16.2(5)	13.3(5)	5.0(5)
C55	36.3(6)	41.6(6)	37.3(6)	18.6(5)	14.8(5)	4.5(5)
C56	36.0(6)	40.5(6)	34.3(6)	13.8(5)	13.3(5)	3.7(5)
C57	30.8(5)	35.0(6)	41.7(6)	17.2(5)	13.7(5)	6.4(4)
C58	46.8(7)	41.8(6)	38.9(6)	19.8(5)	17.6(5)	5.0(5)
C59	46.3(7)	40.5(6)	34.2(6)	13.9(5)	14.5(5)	4.0(5)
C60	69.9(10)	45.6(8)	57.5(9)	24.7(7)	25.5(8)	-4.1(7)
F10	68.1(6)	38.9(4)	79.7(7)	27.8(4)	16.8(5)	5.4(4)
F11	77.4(7)	71.6(6)	50.8(5)	33.7(5)	10.4(5)	21.0(5)
F12	69.0(6)	61.3(6)	84.8(7)	37.1(5)	38.4(6)	26.6(5)
O7	48.6(5)	36.6(4)	30.8(4)	7.3(3)	11.1(4)	1.1(4)
O8	53.1(6)	49.5(5)	40.7(5)	19.2(4)	17.0(4)	-2.1(4)
N4	49.8(6)	35.3(5)	31.9(5)	10.8(4)	12.4(5)	0.6(4)
C61	46.8(7)	34.9(6)	32.2(6)	12.8(5)	10.3(5)	4.0(5)
C62	54.5(8)	34.8(6)	42.6(7)	14.3(5)	18.0(6)	7.0(5)
C63	49.5(7)	47.1(7)	46.0(7)	21.4(6)	18.5(6)	9.1(6)
C64	46.7(7)	45.0(7)	33.3(6)	19.5(5)	7.0(5)	1.0(6)
C65	54.6(8)	35.1(6)	50.3(8)	10.5(6)	12.1(6)	1.9(6)
C66	48.6(7)	37.1(7)	52.4(8)	10.2(6)	15.9(6)	6.5(6)
C67	48.2(7)	32.5(6)	32.2(6)	10.0(5)	10.7(5)	3.3(5)
C68	50.1(7)	32.2(6)	33.1(6)	9.2(5)	11.2(5)	1.1(5)
C69	48.0(7)	34.5(6)	30.3(6)	8.7(5)	14.6(5)	2.3(5)
C70	47.8(7)	42.6(7)	39.8(7)	15.6(5)	15.8(6)	8.7(6)
C71	43.8(7)	47.4(7)	39.6(7)	14.1(6)	17.2(5)	4.2(6)
C72	49.0(7)	38.2(6)	29.4(5)	9.2(5)	14.5(5)	-0.5(5)
C73	46.0(7)	38.6(6)	34.7(6)	12.3(5)	12.5(5)	3.8(5)
C74	43.2(6)	37.1(6)	34.9(6)	10.2(5)	14.8(5)	2.8(5)
C75	55.5(8)	57.1(8)	44.2(7)	25.0(7)	9.2(6)	-5.4(7)
C76	47.7(7)	39.6(6)	33.2(6)	11.1(5)	11.9(5)	1.7(5)
C77	46.7(7)	34.8(6)	33.1(6)	11.3(5)	11.4(5)	3.1(5)
C78	51.4(8)	43.4(7)	46.9(7)	19.4(6)	14.8(6)	6.3(6)
C79	62.3(8)	42.3(7)	31.6(6)	7.9(5)	12.5(6)	-5.6(6)
C80	57.8(9)	55.9(9)	60.0(9)	22.5(7)	26.9(8)	-2.3(7)

Table S3: Bond Lengths in Å for **5af**.

Atom	Atom	Length/Å
F1	C18	1.3335(16)
F2	C18	1.3366(16)
F3	C18	1.3348(18)
O1	C16	1.4314(15)
O1	C17	1.4083(16)
O2	C12	1.3731(16)
O2	C20	1.4174(19)
N1	C8	1.4278(16)
N1	C9	1.4376(16)
N1	C17	1.4441(15)
C1	C2	1.3912(19)

Atom	Atom	Length/Å
C1	C6	1.3964(18)
C1	C7	1.4870(17)
C2	C3	1.3888(18)
C3	C4	1.3900(19)
C4	C5	1.387(2)
C4	C15	1.5044(18)
C5	C6	1.3914(19)
C7	C8	1.3345(18)
C7	C16	1.5099(17)
C8	C19	1.5035(17)
C9	C10	1.3857(18)

Atom	Atom	Length/Å
C9	C14	1.3910(19)
C10	C11	1.3923(19)
C11	C12	1.387(2)
C12	C13	1.3905(19)
C13	C14	1.3845(19)
C17	C18	1.5283(18)
F4	C30	1.3337(16)
F5	C30	1.3320(17)
F6	C30	1.3352(16)
O3	C29	1.4097(15)
O3	C32	1.4323(15)
O4	C37	1.3671(16)
O4	C40	1.4210(19)
N2	C28	1.4216(15)
N2	C29	1.4391(16)
N2	C34	1.4365(16)
C21	C22	1.3959(18)
C21	C26	1.3949(19)
C21	C27	1.4844(17)
C22	C23	1.3899(19)
C23	C24	1.389(2)
C24	C25	1.388(2)
C24	C31	1.5077(19)
C25	C26	1.3901(19)
C27	C28	1.3411(18)
C27	C32	1.5037(17)
C28	C33	1.4996(17)
C29	C30	1.5308(18)
C34	C35	1.3884(17)
C34	C39	1.3914(18)
C35	C36	1.3890(19)
C36	C37	1.388(2)
C37	C38	1.3959(18)
C38	C39	1.3801(18)
F7	C50	1.3346(17)
F8	C50	1.3358(19)
F9	C50	1.3308(17)
O5	C49	1.4078(16)
O5	C52	1.4341(15)
O6	C57	1.3704(15)
O6	C60	1.4213(17)
N3	C48	1.4216(16)
N3	C49	1.4386(17)
N3	C54	1.4362(16)
C41	C42	1.3946(19)

Atom	Atom	Length/Å
C41	C46	1.3907(19)
C41	C47	1.4885(16)
C42	C43	1.393(2)
C43	C44	1.388(2)
C44	C45	1.387(2)
C44	C51	1.5089(19)
C45	C46	1.3899(19)
C47	C48	1.3400(19)
C47	C52	1.5020(18)
C48	C53	1.5007(18)
C49	C50	1.5357(19)
C54	C55	1.3940(18)
C54	C59	1.3872(18)
C55	C56	1.3812(18)
C56	C57	1.3935(17)
C57	C58	1.3883(19)
C58	C59	1.3937(19)
F10	C78	1.3330(17)
F11	C78	1.3362(19)
F12	C78	1.3351(19)
O7	C76	1.4334(16)
O7	C77	1.4099(17)
O8	C72	1.3717(16)
O8	C80	1.4227(19)
N4	C68	1.4210(18)
N4	C69	1.4314(16)
N4	C77	1.4383(17)
C61	C62	1.391(2)
C61	C66	1.3984(19)
C61	C67	1.4869(18)
C62	C63	1.387(2)
C63	C64	1.388(2)
C64	C65	1.388(2)
C64	C75	1.5098(19)
C65	C66	1.386(2)
C67	C68	1.340(2)
C67	C76	1.5026(17)
C68	C79	1.5041(18)
C69	C70	1.389(2)
C69	C74	1.391(2)
C70	C71	1.389(2)
C71	C72	1.389(2)
C72	C73	1.3904(19)
C73	C74	1.3875(18)
C77	C78	1.529(2)

Table S4: Bond Angles in ° for **5af**.

Atom	Atom	Atom	Angle/°
C17	O1	C16	114.54(9)
C12	O2	C20	117.06(12)
C8	N1	C9	118.29(10)
C8	N1	C17	115.45(10)
C9	N1	C17	114.06(10)
C2	C1	C6	117.48(12)
C2	C1	C7	120.55(11)
C6	C1	C7	121.84(12)
C3	C2	C1	121.52(12)
C2	C3	C4	121.02(13)
C3	C4	C15	121.31(13)

Atom	Atom	Atom	Angle/°
C5	C4	C3	117.59(12)
C5	C4	C15	121.09(12)
C4	C5	C6	121.72(12)
C5	C6	C1	120.66(13)
C1	C7	C16	113.52(11)
C8	C7	C1	124.70(11)
C8	C7	C16	121.63(11)
N1	C8	C19	114.33(11)
C7	C8	N1	120.51(11)
C7	C8	C19	124.97(11)
C10	C9	N1	119.06(12)

Atom	Atom	Atom	Angle/°
C10	C9	C14	118.86(12)
C14	C9	N1	122.03(11)
C9	C10	C11	121.15(13)
C12	C11	C10	119.44(12)
O2	C12	C11	124.70(12)
O2	C12	C13	115.49(12)
C11	C12	C13	119.81(12)
C14	C13	C12	120.27(13)
C13	C14	C9	120.47(12)
O1	C16	C7	113.45(10)
O1	C17	N1	114.27(10)
O1	C17	C18	111.78(10)
N1	C17	C18	110.18(10)
F1	C18	F2	106.41(11)
F1	C18	F3	106.93(11)
F1	C18	C17	115.11(11)
F2	C18	C17	110.55(11)
F3	C18	F2	107.19(12)
F3	C18	C17	110.27(11)
C29	O3	C32	114.05(9)
C37	O4	C40	116.93(12)
C28	N2	C29	115.82(10)
C28	N2	C34	118.89(10)
C34	N2	C29	115.02(10)
C22	C21	C27	121.19(12)
C26	C21	C22	117.72(12)
C26	C21	C27	120.94(11)
C23	C22	C21	120.97(13)
C24	C23	C22	121.36(12)
C23	C24	C31	121.40(14)
C25	C24	C23	117.58(12)
C25	C24	C31	121.03(14)
C24	C25	C26	121.63(13)
C25	C26	C21	120.74(12)
C21	C27	C32	113.77(10)
C28	C27	C21	124.43(12)
C28	C27	C32	121.51(11)
N2	C28	C33	114.80(10)
C27	C28	N2	120.41(11)
C27	C28	C33	124.69(11)
O3	C29	N2	114.55(10)
O3	C29	C30	111.38(11)
N2	C29	C30	110.39(11)
F4	C30	F6	106.33(12)
F4	C30	C29	114.88(11)
F5	C30	F4	106.81(11)
F5	C30	F6	106.83(12)
F5	C30	C29	111.34(12)
F6	C30	C29	110.22(11)
O3	C32	C27	113.45(10)
C35	C34	N2	119.62(12)
C35	C34	C39	118.99(12)
C39	C34	N2	121.38(11)
C34	C35	C36	120.96(12)
C37	C36	C35	119.55(12)
O4	C37	C36	124.67(12)
O4	C37	C38	115.51(12)
C36	C37	C38	119.81(12)
C39	C38	C37	120.06(12)
C38	C39	C34	120.60(11)
C49	O5	C52	113.83(10)
C57	O6	C60	117.59(11)

Atom	Atom	Atom	Angle/°
C48	N3	C49	115.86(10)
C48	N3	C54	118.69(11)
C54	N3	C49	116.47(10)
C42	C41	C47	121.55(12)
C46	C41	C42	117.63(12)
C46	C41	C47	120.71(12)
C43	C42	C41	121.05(14)
C44	C43	C42	121.12(14)
C43	C44	C51	122.17(16)
C45	C44	C43	117.73(12)
C45	C44	C51	120.09(16)
C44	C45	C46	121.46(14)
C45	C46	C41	120.99(14)
C41	C47	C52	114.05(11)
C48	C47	C41	124.29(12)
C48	C47	C52	121.54(11)
N3	C48	C53	114.60(11)
C47	C48	N3	120.05(12)
C47	C48	C53	125.25(12)
O5	C49	N3	114.07(10)
O5	C49	C50	111.38(11)
N3	C49	C50	110.76(11)
F7	C50	F8	106.76(12)
F7	C50	C49	115.18(11)
F8	C50	C49	110.38(13)
F9	C50	F7	106.67(13)
F9	C50	F8	107.12(12)
F9	C50	C49	110.34(11)
O5	C52	C47	113.53(11)
C55	C54	N3	121.41(11)
C59	C54	N3	119.65(12)
C59	C54	C55	118.94(11)
C56	C55	C54	120.49(11)
C55	C56	C57	120.25(12)
O6	C57	C56	114.97(11)
O6	C57	C58	125.18(11)
C58	C57	C56	119.85(11)
C57	C58	C59	119.41(12)
C54	C59	C58	121.03(12)
C77	O7	C76	114.45(10)
C72	O8	C80	117.16(12)
C68	N4	C69	119.66(11)
C68	N4	C77	116.00(11)
C69	N4	C77	115.94(10)
C62	C61	C66	117.19(13)
C62	C61	C67	121.33(12)
C66	C61	C67	121.29(13)
C63	C62	C61	121.29(13)
C62	C63	C64	121.41(14)
C63	C64	C65	117.53(13)
C63	C64	C75	121.09(14)
C65	C64	C75	121.35(14)
C66	C65	C64	121.32(13)
C65	C66	C61	121.24(14)
C61	C67	C76	113.67(11)
C68	C67	C61	124.64(12)
C68	C67	C76	121.41(12)
N4	C68	C79	114.50(12)
C67	C68	N4	120.31(12)
C67	C68	C79	124.93(13)
C70	C69	N4	119.36(13)
C70	C69	C74	118.89(12)

Atom	Atom	Atom	Angle/˚
C74	C69	N4	121.75(12)
C69	C70	C71	121.19(14)
C72	C71	C70	119.46(13)
O8	C72	C71	124.70(12)
O8	C72	C73	115.54(13)
C71	C72	C73	119.75(12)
C74	C73	C72	120.34(13)
C73	C74	C69	120.33(12)
O7	C76	C67	113.78(11)

Atom	Atom	Atom	Angle/˚
O7	C77	N4	113.93(11)
O7	C77	C78	111.72(11)
N4	C77	C78	110.79(11)
F10	C78	F11	106.53(12)
F10	C78	F12	106.71(13)
F10	C78	C77	114.94(13)
F11	C78	C77	110.83(12)
F12	C78	F11	106.84(13)
F12	C78	C77	110.57(12)

Table S5: Torsion Angles in ˚ for **5af**.

Atom	Atom	Atom	Atom	Angle/˚
O1	C17	C18	F1	-71.52(14)
O1	C17	C18	F2	49.07(15)
O1	C17	C18	F3	167.42(10)
O2	C12	C13	C14	179.06(12)
N1	C9	C10	C11	176.95(12)
N1	C9	C14	C13	-177.28(12)
N1	C17	C18	F1	56.68(15)
N1	C17	C18	F2	177.26(11)
N1	C17	C18	F3	-64.38(14)
C1	C2	C3	C4	0.1(2)
C1	C7	C8	N1	168.65(12)
C1	C7	C8	C19	-6.0(2)
C1	C7	C16	O1	178.95(10)
C2	C1	C6	C5	0.3(2)
C2	C1	C7	C8	129.03(14)
C2	C1	C7	C16	-55.44(16)
C2	C3	C4	C5	0.9(2)
C2	C3	C4	C15	-178.28(13)
C3	C4	C5	C6	-1.2(2)
C4	C5	C6	C1	0.7(2)
C6	C1	C2	C3	-0.70(19)
C6	C1	C7	C8	-55.13(19)
C6	C1	C7	C16	120.39(14)
C7	C1	C2	C3	175.32(12)
C7	C1	C6	C5	-175.64(13)
C8	N1	C9	C10	146.19(13)
C8	N1	C9	C14	-36.29(17)
C8	N1	C17	O1	41.20(15)
C8	N1	C17	C18	-85.62(14)
C8	C7	C16	O1	-5.37(18)
C9	N1	C8	C7	129.24(13)
C9	N1	C8	C19	-55.59(16)
C9	N1	C17	O1	-100.74(12)
C9	N1	C17	C18	132.44(12)
C9	C10	C11	C12	0.4(2)
C10	C9	C14	C13	0.2(2)
C10	C11	C12	O2	-179.43(13)
C10	C11	C12	C13	0.2(2)
C11	C12	C13	C14	-0.6(2)
C12	C13	C14	C9	0.4(2)
C14	C9	C10	C11	-0.6(2)
C15	C4	C5	C6	177.90(13)
C16	O1	C17	N1	-53.96(14)
C16	O1	C17	C18	72.03(13)
C16	C7	C8	N1	-6.53(19)
C16	C7	C8	C19	178.84(12)

Atom	Atom	Atom	Atom	Angle/°
C17	O1	C16	C7	35.16(15)
C17	N1	C8	C7	-11.02(18)
C17	N1	C8	C19	164.15(11)
C17	N1	C9	C10	-73.02(16)
C17	N1	C9	C14	104.49(14)
C20	O2	C12	C11	1.5(2)
C20	O2	C12	C13	-178.18(13)
O3	C29	C30	F4	-73.53(14)
O3	C29	C30	F5	48.03(15)
O3	C29	C30	F6	166.40(11)
O4	C37	C38	C39	179.68(12)
N2	C29	C30	F4	54.90(15)
N2	C29	C30	F5	176.47(10)
N2	C29	C30	F6	-65.17(14)
N2	C34	C35	C36	178.89(12)
N2	C34	C39	C38	179.90(12)
C21	C22	C23	C24	0.2(2)
C21	C27	C28	N2	165.60(11)
C21	C27	C28	C33	-10.6(2)
C21	C27	C32	O3	179.84(10)
C22	C21	C26	C25	-0.6(2)
C22	C21	C27	C28	129.04(14)
C22	C21	C27	C32	-57.14(16)
C22	C23	C24	C25	-0.4(2)
C22	C23	C24	C31	179.40(13)
C23	C24	C25	C26	0.0(2)
C24	C25	C26	C21	0.5(2)
C26	C21	C22	C23	0.30(19)
C26	C21	C27	C28	-55.52(19)
C26	C21	C27	C32	118.30(14)
C27	C21	C22	C23	175.88(12)
C27	C21	C26	C25	-176.22(13)
C28	N2	C29	O3	39.63(15)
C28	N2	C29	C30	-87.06(13)
C28	N2	C34	C35	137.49(13)
C28	N2	C34	C39	-43.80(17)
C28	C27	C32	O3	-6.14(18)
C29	O3	C32	C27	36.42(15)
C29	N2	C28	C27	-8.63(17)
C29	N2	C28	C33	167.89(11)
C29	N2	C34	C35	-78.76(15)
C29	N2	C34	C39	99.95(14)
C31	C24	C25	C26	-179.74(14)
C32	O3	C29	N2	-54.14(14)
C32	O3	C29	C30	72.03(13)
C32	C27	C28	N2	-7.77(19)
C32	C27	C28	C33	176.07(12)
C34	N2	C28	C27	134.84(12)
C34	N2	C28	C33	-48.64(15)
C34	N2	C29	O3	-105.27(12)
C34	N2	C29	C30	128.05(11)
C34	C35	C36	C37	0.8(2)
C35	C34	C39	C38	-1.4(2)
C35	C36	C37	O4	179.08(13)
C35	C36	C37	C38	-0.6(2)
C36	C37	C38	C39	-0.6(2)
C37	C38	C39	C34	1.6(2)
C39	C34	C35	C36	0.2(2)
C40	O4	C37	C36	6.1(2)
C40	O4	C37	C38	-174.23(13)
O5	C49	C50	F7	72.39(16)
O5	C49	C50	F8	-48.58(14)

Atom	Atom	Atom	Atom	Angle/°
O5	C49	C50	F9	-166.80(11)
O6	C57	C58	C59	-178.72(12)
N3	C49	C50	F7	-55.70(16)
N3	C49	C50	F8	-176.68(11)
N3	C49	C50	F9	65.11(15)
N3	C54	C55	C56	-178.90(12)
N3	C54	C59	C58	179.94(12)
C41	C42	C43	C44	-0.7(2)
C41	C47	C48	N3	-166.26(12)
C41	C47	C48	C53	10.0(2)
C41	C47	C52	O5	-179.77(11)
C42	C41	C46	C45	-1.6(2)
C42	C41	C47	C48	57.48(19)
C42	C41	C47	C52	-118.48(14)
C42	C43	C44	C45	-0.7(2)
C42	C43	C44	C51	179.24(14)
C43	C44	C45	C46	0.9(2)
C44	C45	C46	C41	0.2(2)
C46	C41	C42	C43	1.8(2)
C46	C41	C47	C48	-126.40(15)
C46	C41	C47	C52	57.64(17)
C47	C41	C42	C43	178.06(13)
C47	C41	C46	C45	-177.83(13)
C48	N3	C49	O5	-41.05(15)
C48	N3	C49	C50	85.56(13)
C48	N3	C54	C55	40.53(17)
C48	N3	C54	C59	-139.64(13)
C48	C47	C52	O5	4.14(18)
C49	O5	C52	C47	-35.82(15)
C49	N3	C48	C47	8.73(18)
C49	N3	C48	C53	-167.93(11)
C49	N3	C54	C55	-105.36(14)
C49	N3	C54	C59	74.47(16)
C51	C44	C45	C46	-178.98(14)
C52	O5	C49	N3	55.03(14)
C52	O5	C49	C50	-71.26(14)
C52	C47	C48	N3	9.42(19)
C52	C47	C48	C53	-174.30(13)
C54	N3	C48	C47	-137.37(13)
C54	N3	C48	C53	45.97(16)
C54	N3	C49	O5	105.81(12)
C54	N3	C49	C50	-127.57(12)
C54	C55	C56	C57	-1.01(19)
C55	C54	C59	C58	-0.2(2)
C55	C56	C57	O6	179.74(11)
C55	C56	C57	C58	-0.32(19)
C56	C57	C58	C59	1.3(2)
C57	C58	C59	C54	-1.1(2)
C59	C54	C55	C56	1.27(19)
C60	O6	C57	C56	174.56(13)
C60	O6	C57	C58	-5.37(19)
O7	C77	C78	F10	-72.39(16)
O7	C77	C78	F11	48.45(16)
O7	C77	C78	F12	166.73(12)
O8	C72	C73	C74	-179.91(11)
N4	C69	C70	C71	179.01(12)
N4	C69	C74	C73	179.40(11)
N4	C77	C78	F10	55.81(17)
N4	C77	C78	F11	176.65(12)
N4	C77	C78	F12	-65.07(16)
C61	C62	C63	C64	-0.2(2)
C61	C67	C68	N4	167.26(13)

Atom	Atom	Atom	Atom	Angle/°
C61	C67	C68	C79	-6.5(2)
C61	C67	C76	O7	-179.45(11)
C62	C61	C66	C65	-0.1(2)
C62	C61	C67	C68	128.03(16)
C62	C61	C67	C76	-57.97(17)
C62	C63	C64	C65	1.4(2)
C62	C63	C64	C75	-176.48(13)
C63	C64	C65	C66	-1.9(2)
C64	C65	C66	C61	1.3(3)
C66	C61	C62	C63	-0.5(2)
C66	C61	C67	C68	-57.2(2)
C66	C61	C67	C76	116.78(15)
C67	C61	C62	C63	174.51(13)
C67	C61	C66	C65	-175.04(14)
C68	N4	C69	C70	147.82(13)
C68	N4	C69	C74	-32.85(18)
C68	N4	C77	O7	41.57(16)
C68	N4	C77	C78	-85.42(15)
C68	C67	C76	O7	-5.24(19)
C69	N4	C68	C67	135.29(13)
C69	N4	C68	C79	-50.30(17)
C69	N4	C77	O7	-106.60(13)
C69	N4	C77	C78	126.41(13)
C69	C70	C71	C72	2.1(2)
C70	C69	C74	C73	-1.27(19)
C70	C71	C72	O8	178.38(12)
C70	C71	C72	C73	-2.2(2)
C71	C72	C73	C74	0.60(19)
C72	C73	C74	C69	1.15(19)
C74	C69	C70	C71	-0.3(2)
C75	C64	C65	C66	175.94(14)
C76	O7	C77	N4	-53.66(14)
C76	O7	C77	C78	72.84(14)
C76	C67	C68	N4	-6.3(2)
C76	C67	C68	C79	179.92(14)
C77	O7	C76	C67	34.86(16)
C77	N4	C68	C67	-11.63(19)
C77	N4	C68	C79	162.78(12)
C77	N4	C69	C70	-65.24(16)
C77	N4	C69	C74	114.08(14)
C80	O8	C72	C71	6.49(19)
C80	O8	C72	C73	-172.98(12)

Table S6: Hydrogen Fractional Atomic Coordinates (×10[4]) and Equivalent Isotropic Displacement Parameters (Å[2]×10[3]) for **5af.** U_{eq} is defined as 1/3 of the trace of the orthogonalised U_{ij}.

Atom	x	y	z	U_{eq}
H2	5888.05	-729.32	3890.68	47
H3	4975.44	-2114.86	3529.36	47
H5	2651.49	-1205.52	2328.88	53
H6	3556.33	190.9	2700.98	51
H10	6247.18	3653.91	3242.59	50
H11	7425.21	4109.71	2812.91	51
H13	8345.65	1652.73	2539.45	49
H14	7180.62	1212.75	2984.36	47
H15A	3192.75	-3177.58	2184.89	57
H15B	2444.26	-2608.29	2531.58	57
H15C	3305.28	-2866.99	3197.51	57
H15D	2768.78	-2590.99	3091.1	57
H15E	3517.27	-3160.29	2744.41	57

Atom	x	y	z	U_{eq}
H15F	2656.25	-2901.58	2078.48	57
H16A	5429.07	1331.6	4909.57	46
H16B	6311.75	813.27	4829.26	46
H17	6622.55	3169.99	4610.95	43
H19A	4428.98	1107.46	1946	48
H19B	4812.4	159.05	1920.22	48
H19C	5440.47	951.72	1865.5	48
H19D	5358.92	371.36	1875.15	48
H19E	4975.5	1319.77	1900.92	48
H19F	4347.43	527.1	1955.64	48
H20A	8282.57	4065.35	1901.51	89
H20B	9403.23	4077.57	2206.88	89
H20C	8923.16	4478.07	2927.57	89
H22	1666.69	344.83	11304.73	46
H23	1059.04	-782.64	11650.68	50
H25	917.65	-2710.91	9336.8	53
H26	1510.43	-1589.78	8974.58	50
H29	2369.14	1874.09	8901.69	42
H31A	-118.99	-2841.95	10367.91	67
H31B	575.52	-2339.61	11372.2	67
H31C	917.88	-3106.83	10687.32	67
H31D	1035.26	-2683.65	11250.38	67
H31E	340.75	-3185.98	10246.09	67
H31F	-1.61	-2418.76	10930.97	67
H32A	708.28	644.88	9402.97	46
H32B	1305.4	1241.88	10417.75	46
H33A	3786.84	-325.86	10258.99	47
H33B	3854.85	-362.05	9349.35	47
H33C	3085.93	-1078.53	9345.28	47
H33D	3364.9	-851.77	9043.42	47
H33E	3296.9	-815.57	9953.06	47
H33F	4065.82	-99.09	9957.13	47
H35	3731.78	1090.62	8201	49
H36	5225.03	1907.93	8608.8	50
H38	5641.68	2462.86	11175.86	46
H39	4131.89	1692.04	10763.28	44
H40A	6845.66	2312.5	9200.11	92
H40B	7409.68	3294.18	9924.23	92
H40C	6336.83	3208.12	9299.52	92
H42	-1739.05	6366.76	3722.3	54
H43	-2983.17	5809.28	4005.47	59
H45	-1169.21	5970.68	6364.33	57
H46	75.1	6537.95	6092.41	52
H49	1963.12	7290.39	3915.5	44
H51A	-3508.71	5865.39	5352.39	76
H51B	-2765.55	5515.37	6029.9	76
H51C	-3258.07	4845.49	5016.83	76
H51D	-2846.18	4952.11	5580.36	76
H51E	-3589.34	5302.13	4902.85	76
H51F	-3096.82	5972.01	5915.92	76
H52A	1111.49	6217.14	5299.48	48
H52B	580.15	5638.3	4268.69	48
H53A	-203.48	8876.76	5026.85	54
H53B	-1069.05	8058.24	4633.22	54
H53C	-748.23	8394.06	3984.13	54
H53D	-1143.69	8009.28	4069.29	54
H53E	-278.12	8827.8	4462.91	54
H53F	-598.95	8491.98	5112.01	54
H55	1527.33	9033.09	5755.41	44
H56	2290.35	10526.31	6233.47	44
H58	2113.94	10091.24	3742.54	49
H59	1305.28	8605.59	3261.39	49

Atom	x	y	z	U_{eq}
H60A	2430.61	11714.23	4369.53	84
H60B	3382.07	11270.66	4565.06	84
H60C	3367.08	12301.12	5180.42	84
H62	5891.22	4173.58	1125.37	53
H63	7266.02	5153.15	1514.87	55
H65	6097	7328.67	2588.85	60
H66	4710.83	6357.59	2182.35	58
H70	1184.21	3650.12	1506.38	52
H71	669.45	2605.63	2005.33	53
H73	3299.02	1908.95	2662.27	49
H74	3786.82	2902.9	2100.82	47
H75A	8263.97	6730.3	2516.59	64
H75B	7710.02	7591.64	2717.58	64
H75C	7694.94	7056.36	1740.59	64
H75D	7515.32	7521.9	2133.25	64
H75E	8069.27	6660.56	1932.26	64
H75F	8084.35	7195.84	2909.25	64
H76A	3723.48	4507.74	30.72	50
H76B	4280.82	3670.05	109.17	50
H77	1821.39	3278.23	273.92	47
H79A	3730.6	5406.02	2962.18	58
H79B	4780.46	5239	3001.64	58
H79C	4097.6	4482.94	3047.95	58
H79D	4675.18	4679.29	3045.67	58
H79E	3625.31	4846.3	3006.21	58
H79F	4308.17	5602.36	2959.9	58
H80A	368.04	1157.98	2015.58	68
H80B	570.72	1940.04	2967.95	68
H80C	695.89	901.83	2870.8	68
H80D	721.73	1508.59	3220.64	68
H80E	519.05	726.52	2268.27	68
H80F	393.88	1764.73	2365.42	68

Table S7: Atomic Occupancies for all atoms that are not fully occupied in **5af**.

Atom	Occupancy	Atom	Occupancy	Atom	Occupancy
H15A	0.72(2)	H33A	0.108(18)	H75A	0.77(2)
H15B	0.72(2)	H33B	0.108(18)	H75B	0.77(2)
H15C	0.72(2)	H33C	0.108(18)	H75C	0.77(2)
H15D	0.28(2)	H33D	0.892(18)	H75D	0.23(2)
H15E	0.28(2)	H33E	0.892(18)	H75E	0.23(2)
H15F	0.28(2)	H33F	0.892(18)	H75F	0.23(2)
H19A	0.910(18)	H51A	0.19(3)	H79A	0.86(2)
H19B	0.910(18)	H51B	0.19(3)	H79B	0.86(2)
H19C	0.910(18)	H51C	0.19(3)	H79C	0.86(2)
H19D	0.090(18)	H51D	0.81(3)	H79D	0.14(2)
H19E	0.090(18)	H51E	0.81(3)	H79E	0.14(2)
H19F	0.090(18)	H51F	0.81(3)	H79F	0.14(2)
H31A	0.40(2)	H53A	0.823(19)	H80A	0.92(2)
H31B	0.40(2)	H53B	0.823(19)	H80B	0.92(2)
H31C	0.40(2)	H53C	0.823(19)	H80C	0.92(2)
H31D	0.60(2)	H53D	0.177(19)	H80D	0.08(2)
H31E	0.60(2)	H53E	0.177(19)	H80E	0.08(2)
H31F	0.60(2)	H53F	0.177(19)	H80F	0.08(2)

(*E*)-4-(1-(*p*-Tolyl)ethylidene)-3-tosyl-2-(trifluoromethyl)oxazolidine (4aff)

CCDC deposition Number 2359063

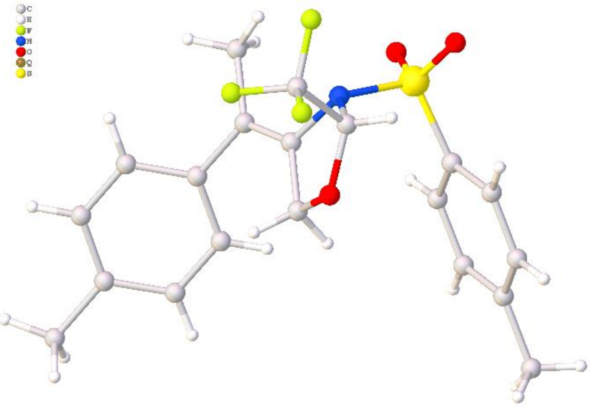

Crystals of compound **4aff** were collected upon crystallization via vapour diffusion with Et_2O/pentane at 5 °C.

Experimental. Single clear pale colourless prism-shaped crystals of **4aff** were used as supplied. A suitable crystal with dimensions 0.58 × 0.23 × 0.17 mm^3 was selected and mounted on a SuperNova, Dual, Cu at home/near, AtlasS2 diffractometer. The crystal was kept at a steady T = 140.00(10) K during data collection. The structure was solved with the ShelXT (Sheldrick, 2015) solution program using dual methods and by using Olex2 1.5 (Dolomanov et al., 2009) as the graphical interface. The model was refined with ShelXL 2019/3 (Sheldrick, 2015) using full matrix least squares minimisation on $\boldsymbol{F^2}$.

Crystal Data. $C_{20}H_{20}NO_3F_3S$, M_r = 411.43, triclinic, P-1 (No. 2), a = 10.5083(3) Å, b = 10.6382(3) Å, c = 10.6680(3) Å, α = 60.550(3)°, β = 68.701(3)°, γ = 79.050(2)°, V = 967.45(6) $Å^3$, T = 140.00(10) K, Z = 2, Z' = 1, μ(Mo K_α) = 0.216, 31233 reflections measured, 6721 unique (R_{int} = 0.0351) which were used in all calculations. The final wR_2 was 0.1098 (all data) and R_1 was 0.0417 (I≥2 σ(I)).

Compound	4aff
Formula	$C_{20}H_{20}NO_3F_3S$
$D_{calc.}$/ g cm^{-3}	1.412
μ/mm^{-1}	0.216
Formula Weight	411.43
Colour	clear pale colourless
Shape	prism
Size/mm^3	0.58×0.23×0.17
T/K	140.00(10)
Crystal System	triclinic
Space Group	P-1
a/Å	10.5083(3)
b/Å	10.6382(3)
c/Å	10.6680(3)
α/°	60.550(3)
β/°	68.701(3)
γ/°	79.050(2)
V/Å^3	967.45(6)
Z	2
Z'	1
Wavelength/Å	0.71073
Radiation type	Mo K_α
Θ_{min}/°	3.006
Θ_{max}/°	32.865
Measured Refl's.	31233
Indep't Refl's	6721
Refl's I≥2 σ(I)	5550
R_{int}	0.0351
Parameters	333
Restraints	0
Largest Peak	0.392
Deepest Hole	-0.405
GooF	1.043
wR_2 (all data)	0.1098
wR_2	0.1026
R_1 (all data)	0.0535
R_1	0.0417

Structure Quality Indicators

**Reflections:**	d min (MoKα) 2Θ=65.7° 0.65	I/σ(I) 31.0	Rint m=4.65 3.51%	Full 50.5° 93% to 65.7° 99.8
**Refinement:**	Shift 0.000	Max Peak 0.4	Min Peak -0.4	GooF 1.043

A clear pale colourless prism-shaped crystal with dimensions 0.58 × 0.23 × 0.17 mm^3 was mounted. Data were collected using a SuperNova, Dual, Cu at home/near, AtlasS2 diffractometer operating at T = 140.00(10) K.

Data were measured using ω scans with Mo K_α radiation. The diffraction pattern was indexed and the total number of runs and images was based on the strategy calculation from the program CrysAlisPro system (CCD 43.95a 64-bit (release 03-11-2023)). The maximum resolution that was achieved was Θ = 32.865° (0.65 Å).

The unit cell was refined using CrysAlisPro 1.171.43.94a (Rigaku OD, 2023) on 12009 reflections, 38% of the observed reflections.

Data reduction, scaling and absorption corrections were performed using CrysAlisPro 1.171.43.94a

(Rigaku OD, 2023). The final completeness is 99.80 % out to 32.865° in *Θ*. A gaussian absorption correction was performed using CrysAlisPro 1.171.43.94a (Rigaku Oxford Diffraction, 2023). The numerical absorption correction based on gaussian integration over a multifaceted crystal model. The empirical absorption correction was done using spherical harmonics, implemented in SCALE3 ABSPACK scaling algorithm. The absorption coefficient μ of this crystal is 0.216 mm^{-1} at this wavelength (λ = 0.71073Å) and the minimum and maximum transmissions are 0.519 and 1.000.

The structure was solved and the space group *P*-1 (# 2) determined by the ShelXT (Sheldrick, 2015) structure solution program using dual methods and refined by full matrix least squares minimisation on $\boldsymbol{F^2}$ using version 2019/3 of ShelXL (Sheldrick, 2015). All non-hydrogen atoms were refined anisotropically. Hydrogen atom positions were calculated geometrically and refined using freely.

There is a single formula unit in the asymmetric unit, which is represented by the reported sum formula. In other words: Z is 2 and Z' is 1. The moiety formula is C20 H20 F3 N O3 S.

Data Plots: Diffraction Data

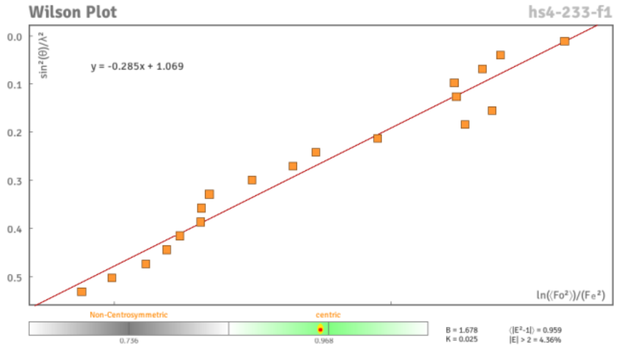

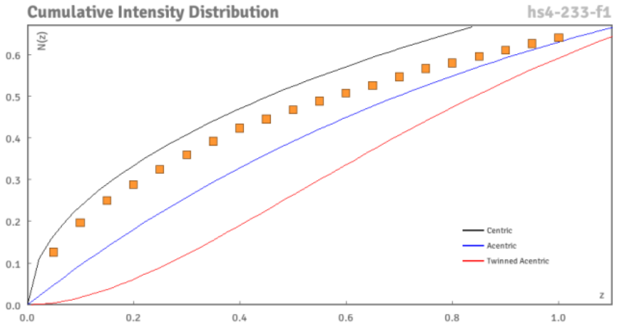

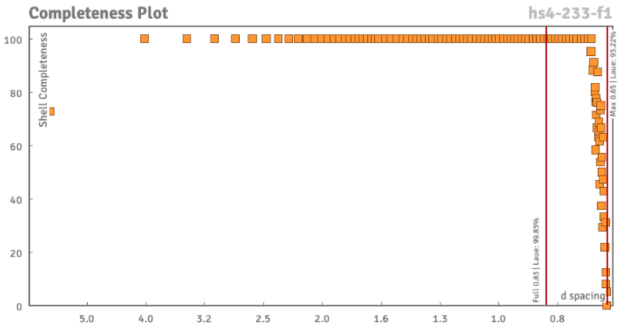

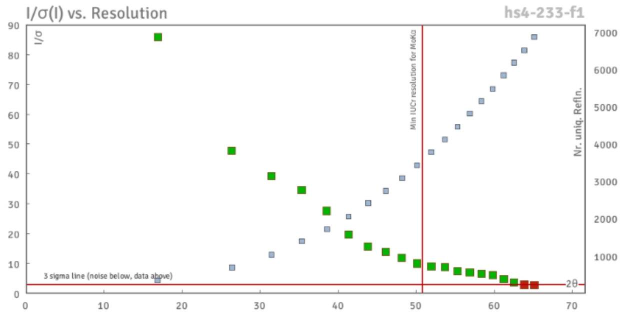

Data Plots: Refinement and Data

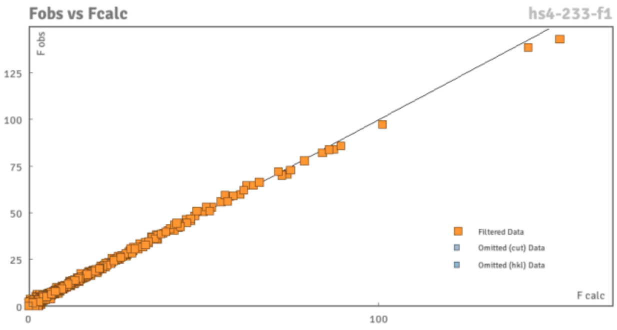

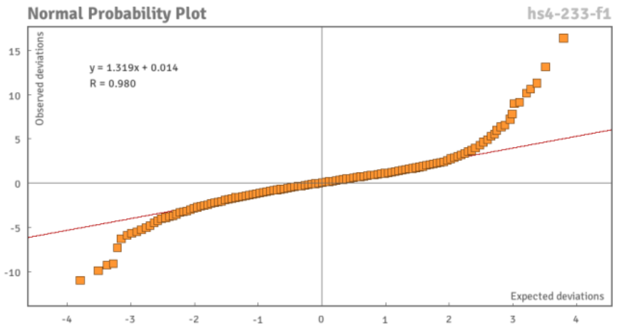

Reflection Statistics

Total reflections (after filtering)	31233	Unique reflections	6721
Completeness	0.932	Mean I/σ	19.29
hkl$_{max}$ collected	(15, 16, 16)	hkl$_{min}$ collected	(-16, -15, -15)

hkl_{max} used	(16, 16, 16)	hkl_{min} used	(-14, -12, 0)
Lim d_{max} collected	100.0	Lim d_{min} collected	0.36
d_{max} used	6.78	d_{min} used	0.65
Friedel pairs	5972	Friedel pairs merged	1
Inconsistent equivalents	1	R_{int}	0.0351
R_{sigma}	0.0323	Intensity transformed	0
Omitted reflections	0	Omitted by user (OMIT hkl)	0
Multiplicity	(3906, 3755, 2551, 1367, 528, 274, 161, 88, 50, 12, 1)	Maximum multiplicity	19
Removed systematic absences	0	Filtered off (Shel/OMIT)	0

Table S8: Fractional Atomic Coordinates (×10^4) and Equivalent Isotropic Displacement Parameters (Å^2×10^3) for **4aff.** U_{eq} is defined as 1/3 of the trace of the orthogonalised U_{ij}.

Atom	x	y	z	U_{eq}
S1	7004.8(3)	9264.8(3)	1230.3(3)	19.04(7)
F1	6838.8(11)	7867.2(10)	-1493.9(10)	40.8(2)
F2	5172.4(11)	6376.2(11)	-114.1(13)	49.7(3)
F3	7179.3(10)	5699.1(9)	102.9(11)	41.1(2)
O1	5335.7(9)	6134.6(10)	2535.6(11)	27.5(2)
O2	8316.9(9)	9517.3(10)	1166.8(11)	26.53(19)
O3	6401.9(9)	10291.3(9)	95.3(10)	24.53(18)
N1	7155.2(9)	7722.7(10)	1104.2(11)	18.85(18)
C1	5914.8(12)	7292.9(13)	1103.5(14)	21.9(2)
C2	6306.9(12)	5595.7(13)	3365.9(14)	22.3(2)
C3	7581.4(11)	6447.6(12)	2268.1(13)	19.3(2)
C4	8879.5(11)	6056.0(13)	2215.7(14)	22.5(2)
C5	9145.5(12)	4683.9(14)	3480.8(14)	23.5(2)
C6	10098.4(13)	3675.2(16)	3183.2(16)	30.7(3)
C7	10271.3(14)	2345.2(15)	4353.3(17)	31.5(3)
C8	9506.0(13)	1963.5(14)	5856.8(16)	27.4(3)
C9	8588.9(14)	2985.7(15)	6160.3(16)	29.0(3)
C10	8414.7(13)	4324.5(15)	4996.9(15)	26.8(2)
C11	9630.9(16)	479.9(16)	7105.7(19)	35.0(3)
C12	10072.1(13)	6915.8(17)	913.0(17)	32.3(3)
C13	6285.6(15)	6800.9(15)	-108.6(17)	30.3(3)
C14	5846.8(11)	8903.9(12)	3033.7(13)	19.1(2)
C15	4447.3(12)	9039.3(13)	3244.0(14)	21.2(2)
C16	3544.6(12)	8658.0(14)	4694.8(15)	24.3(2)
C17	4005.9(13)	8141.0(13)	5936.1(14)	23.2(2)
C18	5411.7(13)	8013.7(14)	5695.2(15)	25.5(2)
C19	6337.5(12)	8390.9(14)	4253.3(15)	23.4(2)
C20	2998.6(16)	7722.1(17)	7499.6(17)	32.2(3)

Table S9: Anisotropic Displacement Parameters (×10^4) for **4aff.** The anisotropic displacement factor exponent takes the form: $-2\pi^2[h^2a^{*2} \times U_{11} + ... + 2hka^* \times b^* \times U_{12}]$

Atom	U_{11}	U_{22}	U_{33}	U_{23}	U_{13}	U_{12}
S1	18.92(12)	17.72(13)	18.46(14)	-6.35(11)	-6.03(9)	-1.57(9)
F1	67.5(6)	31.6(5)	24.5(4)	-11.2(4)	-19.3(4)	1.3(4)
F2	62.6(6)	49.7(6)	63.1(7)	-34.8(6)	-33.1(5)	-4.6(5)
F3	60.3(6)	28.2(4)	36.3(5)	-20.2(4)	-17.4(4)	15.6(4)
O1	20.7(4)	29.5(5)	27.9(5)	-8.6(4)	-7.0(3)	-5.6(3)
O2	21.5(4)	28.0(5)	28.9(5)	-11.0(4)	-6.9(3)	-6.0(3)
O3	28.0(4)	19.7(4)	21.4(4)	-5.4(3)	-9.7(3)	0.9(3)
N1	18.0(4)	18.6(4)	18.8(5)	-7.8(4)	-6.3(3)	0.9(3)
C1	21.7(5)	20.8(5)	25.9(6)	-11.9(5)	-9.9(4)	1.7(4)
C2	19.9(5)	20.8(5)	22.0(6)	-7.9(5)	-3.8(4)	-2.9(4)
C3	19.0(5)	18.4(5)	15.6(5)	-5.6(4)	-3.2(4)	-0.6(4)

Atom	U_{11}	U_{22}	U_{33}	U_{23}	U_{13}	U_{12}
C4	19.3(5)	22.7(5)	18.1(5)	-6.2(5)	-3.5(4)	1.1(4)
C5	20.0(5)	24.0(6)	20.6(6)	-6.6(5)	-6.3(4)	0.9(4)
C6	24.3(6)	31.7(7)	24.3(6)	-8.9(6)	-4.2(5)	5.8(5)
C7	26.7(6)	27.6(7)	31.7(7)	-9.7(6)	-9.7(5)	6.9(5)
C8	25.9(6)	25.7(6)	28.2(6)	-6.5(5)	-14.2(5)	-0.9(4)
C9	32.2(6)	30.7(7)	20.3(6)	-7.2(5)	-10.7(5)	-0.7(5)
C10	28.6(6)	27.7(6)	22.0(6)	-10.5(5)	-9.4(5)	3.8(5)
C11	39.0(7)	28.5(7)	32.8(8)	-4.8(6)	-19.8(6)	-0.6(6)
C12	18.9(5)	32.4(7)	24.7(6)	-2.9(6)	-0.2(5)	0.3(5)
C13	42.6(7)	22.8(6)	31.7(7)	-14.1(6)	-18.4(6)	4.2(5)
C14	21.3(5)	17.1(5)	18.7(5)	-7.8(4)	-6.3(4)	-1.0(4)
C15	22.4(5)	21.5(5)	21.3(6)	-10.3(5)	-8.9(4)	1.4(4)
C16	22.6(5)	25.1(6)	25.8(6)	-13.6(5)	-5.9(4)	0.1(4)
C17	31.0(6)	17.1(5)	20.2(5)	-8.9(4)	-5.1(4)	-2.6(4)
C18	33.8(6)	24.2(6)	21.3(6)	-10.0(5)	-12.4(5)	-0.2(5)
C19	23.9(5)	24.8(6)	24.9(6)	-11.8(5)	-10.3(4)	-1.2(4)
C20	38.3(7)	30.4(7)	22.4(6)	-12.4(6)	-1.3(5)	-4.6(5)

Table S10: Bond Lengths in Å for **4aff**.

Atom	Atom	Length/Å
S1	O2	1.4258(9)
S1	O3	1.4332(9)
S1	N1	1.6844(10)
S1	C14	1.7550(12)
F1	C13	1.3367(17)
F2	C13	1.3345(17)
F3	C13	1.3351(16)
O1	C1	1.4075(15)
O1	C2	1.4456(15)
N1	C1	1.4614(14)
N1	C3	1.4489(15)
C1	C13	1.5231(18)
C2	C3	1.5048(16)
C3	C4	1.3376(16)
C4	C5	1.4873(17)

Atom	Atom	Length/Å
C4	C12	1.5030(17)
C5	C6	1.3968(18)
C5	C10	1.3975(18)
C6	C7	1.387(2)
C7	C8	1.389(2)
C8	C9	1.392(2)
C8	C11	1.505(2)
C9	C10	1.3893(19)
C14	C15	1.3948(15)
C14	C19	1.3924(17)
C15	C16	1.3864(17)
C16	C17	1.3927(18)
C17	C18	1.3961(18)
C17	C20	1.5056(18)
C18	C19	1.3884(18)

Table S11: Bond Angles in ° for **4aff**

Atom	Atom	Atom	Angle/°
O2	S1	O3	120.32(6)
O2	S1	N1	106.19(5)
O2	S1	C14	108.97(6)
O3	S1	N1	105.20(5)
O3	S1	C14	109.27(5)
N1	S1	C14	105.90(5)
C1	O1	C2	109.20(9)
C1	N1	S1	114.77(7)
C3	N1	S1	116.44(8)
C3	N1	C1	104.61(9)
O1	C1	N1	108.34(9)
O1	C1	C13	109.23(10)
N1	C1	C13	109.18(10)
O1	C2	C3	104.95(10)
N1	C3	C2	106.08(9)
C4	C3	N1	124.90(10)
C4	C3	C2	128.43(11)
C3	C4	C5	118.21(11)

Atom	Atom	Atom	Angle/°
C3	C4	C12	122.82(11)
C5	C4	C12	118.94(10)
C6	C5	C4	120.61(11)
C6	C5	C10	117.63(12)
C10	C5	C4	121.70(11)
C7	C6	C5	120.84(13)
C6	C7	C8	121.57(13)
C7	C8	C9	117.66(12)
C7	C8	C11	121.10(13)
C9	C8	C11	121.21(13)
C10	C9	C8	121.17(13)
C9	C10	C5	121.04(12)
F1	C13	C1	111.52(11)
F2	C13	F1	107.42(11)
F2	C13	F3	107.48(11)
F2	C13	C1	110.40(12)
F3	C13	F1	107.05(12)
F3	C13	C1	112.72(11)

Atom	Atom	Atom	Angle/°
C15	C14	S1	119.54(9)
C19	C14	S1	119.34(9)
C19	C14	C15	120.97(11)
C16	C15	C14	118.81(11)
C15	C16	C17	121.48(11)
C16	C17	C18	118.58(11)
C16	C17	C20	120.16(12)
C18	C17	C20	121.26(12)
C19	C18	C17	121.10(12)
C18	C19	C14	119.07(11)

Table S12: Torsion Angles in ° for **4aff**.

Atom	Atom	Atom	Atom	Angle/°
S1	N1	C1	O1	-104.93(9)
S1	N1	C1	C13	136.20(9)
S1	N1	C3	C2	101.72(9)
S1	N1	C3	C4	-86.46(13)
S1	C14	C15	C16	175.75(9)
S1	C14	C19	C18	-175.63(9)
O1	C1	C13	F1	179.87(10)
O1	C1	C13	F2	60.54(13)
O1	C1	C13	F3	-59.67(14)
O1	C2	C3	N1	19.22(12)
O1	C2	C3	C4	-152.21(13)
O2	S1	N1	C1	-178.27(8)
O2	S1	N1	C3	59.07(9)
O2	S1	C14	C15	161.25(9)
O2	S1	C14	C19	-23.16(11)
O3	S1	N1	C1	-49.70(9)
O3	S1	N1	C3	-172.35(8)
O3	S1	C14	C15	27.97(11)
O3	S1	C14	C19	-156.44(9)
N1	S1	C14	C15	-84.89(10)
N1	S1	C14	C19	90.70(10)
N1	C1	C13	F1	-61.82(14)
N1	C1	C13	F2	178.85(10)
N1	C1	C13	F3	58.64(15)
N1	C3	C4	C5	-177.83(11)
N1	C3	C4	C12	0.1(2)
C1	O1	C2	C3	-4.47(12)
C1	N1	C3	C2	-26.09(11)
C1	N1	C3	C4	145.73(12)
C2	O1	C1	N1	-11.94(12)
C2	O1	C1	C13	106.89(11)
C2	C3	C4	C5	-7.9(2)
C2	C3	C4	C12	170.01(13)
C3	N1	C1	O1	23.89(12)
C3	N1	C1	C13	-94.97(11)
C3	C4	C5	C6	131.60(14)
C3	C4	C5	C10	-45.35(18)
C4	C5	C6	C7	-174.75(13)
C4	C5	C10	C9	174.29(12)
C5	C6	C7	C8	0.2(2)
C6	C5	C10	C9	-2.7(2)
C6	C7	C8	C9	-2.4(2)
C6	C7	C8	C11	175.63(14)
C7	C8	C9	C10	1.9(2)
C8	C9	C10	C5	0.6(2)
C10	C5	C6	C7	2.3(2)
C11	C8	C9	C10	-176.06(13)
C12	C4	C5	C6	-46.38(18)
C12	C4	C5	C10	136.68(14)
C14	S1	N1	C1	65.96(9)
C14	S1	N1	C3	-56.69(9)

Atom	Atom	Atom	Atom	Angle/°
C14	C15	C16	C17	-0.27(18)
C15	C14	C19	C18	-0.10(18)
C15	C16	C17	C18	0.18(18)
C15	C16	C17	C20	-179.38(12)
C16	C17	C18	C19	-0.04(18)
C17	C18	C19	C14	0.00(19)
C19	C14	C15	C16	0.23(18)
C20	C17	C18	C19	179.51(12)

Table S13: Hydrogen Fractional Atomic Coordinates (×10[4]) and Equivalent Isotropic Displacement Parameters (Å[2]×10[3]) for **4aff.** U_{eq} is defined as 1/3 of the trace of the orthogonalised U_{ij}.

Atom	x	y	z	U_{eq}
H1	5286(16)	8068(18)	881(18)	25(4)
H2A	6448(16)	4581(18)	3693(18)	26(4)
H2B	5903(15)	5746(17)	4255(18)	24(4)
H6	10619(19)	3940(20)	2130(20)	41(5)
H7	10900(20)	1650(20)	4120(20)	50(6)
H9	8090(20)	2780(20)	7150(20)	47(5)
H10	7835(19)	5000(20)	5210(20)	41(5)
H11A	8760(20)	-110(20)	7590(30)	59(6)
H11B	9860(20)	530(20)	7880(30)	60(6)
H11C	10430(20)	-90(30)	6710(30)	67(7)
H12A	10740(20)	6250(20)	670(20)	58(6)
H12B	9835(18)	7590(20)	30(20)	35(4)
H12C	10530(20)	7440(20)	1180(30)	60(6)
H15	4133(16)	9367(17)	2399(19)	26(4)
H16	2578(19)	8746(19)	4830(20)	38(5)
H18	5758(17)	7664(18)	6520(20)	31(4)
H19	7259(19)	8280(20)	4130(20)	38(5)
H20A	2510(20)	8570(30)	7530(30)	62(6)
H20B	3450(30)	7360(30)	8190(30)	71(7)
H20C	2340(20)	7030(30)	7730(30)	68(7)

H. References

[1] S. S. Zalesskiy, V. P. Ananikov, *Organometallics* **2012**, *31*, 2302–2309.
[2] L. Ingrassia, M. Mulliez, *Synthesis* **1999**, *1999*, 1731–1738.
[3] H. Mimura, K. Kawada, T. Yamashita, T. Sakamoto, Y. Kikugawa, *J. Fluor. Chem.* **2010**, *131*, 477–486.
[4] I. Kumadaki, S. Jonoshita, A. Harada, M. Omote, A. Ando, *J. Fluor. Chem.* **1999**, *97*, 61–63.
[5] G.-Y. Lin, C.-Y. Yang, R.-S. Liu, *J. Org. Chem.* **2007**, *72*, 6753–6757.
[6] N. Cabrera-Lobera, P. Rodríguez-Salamanca, J. C. Nieto-Carmona, E. Buñuel, D. J. Cárdenas, *Chem. – Eur. J.* **2018**, *24*, 784–788.
[7] N. Chaisan, S. Ruengsangtongkul, C. Thongsornkleeb, J. Tummatorn, S. Ruchirawat, *Synlett* **2022**, *33*, 1426–1430.
[8] R. S. Kim, L. V. Dinh-Nguyen, K. W. Shimkin, D. A. Watson, *Org. Lett.* **2020**, *22*, 8106–8110.
[9] A. Hamze, O. Provot, J.-D. Brion, M. Alami, *J. Org. Chem.* **2007**, *72*, 3868–3874.
[10] F. Y. Kwong, Y. M. Li, W. H. Lam, L. Qiu, H. W. Lee, C. H. Yeung, K. S. Chan, A. S. C. Chan, *Chem. – Eur. J.* **2005**, *11*, 3872–3880.
[11] R. Iioka, K. Yorozu, Y. Sakai, R. Kawai, N. Hatae, K. Takashima, G. Tanabe, H. Wasada, M. Yoshimatsu, *Eur. J. Org. Chem.* **2021**, *2021*, 1553–1558.
[12] B.-N. Lin, S.-H. Huang, W.-Y. Wu, C.-Y. Mou, F.-Y. Tsai, *Molecules* **2010**, *15*, 9157–9173.
[13] F. Emmetiere, R. Ratnayake, H. A. M. Schares, K. F. M. Jones, E. Bevan–Smith, H. Luesch, D. A. Harki, A. J. Grenning, *Chem. Weinh. Bergstr. Ger.* **2021**, *27*, 5564–5571.
[14] A. Köpfer, B. Breit, *Angew. Chem. Int. Ed.* **2015**, *54*, 6913–6917.
[15] T. K. Roy, T. Liu, Y. Qian, C. A. Sojdak, M. C. Kozlowski, M. I. Lester, *Chem. Sci.* **2023**, *14*, 10471–10477.
[16] T. D. R. Morgan, L. M. LeBlanc, G. H. Ardagh, R. J. Boyd, D. J. Burnell, *J. Org. Chem.* **2015**, *80*, 1042–1051.
[17] W. Gong, Z. Zhou, J. Shi, B. Wu, B. Huang, W. Yi, *Org. Lett.* **2018**, *20*, 182–185.
[18] S. M. Rummelt, K. Radkowski, D.-A. Roşca, A. Fürstner, *J. Am. Chem. Soc.* **2015**, *137*, 5506–5519.
[19] B. R. Blank, I. P. Andrews, O. Kwon, *ChemCatChem* **2020**, *12*, 4352–4372.
[20] M. Puriņš, L. Eichenberger, J. Waser, *Chem. Commun.* **2023**, DOI 10.1039/D3CC01847D.
[21] E. C. Juenge, P. Spangler, *J. Org. Chem.* **1964**, *29*, 226–228.
[22] W. Zeinyeh, Z. Mahiout, S. Radix, T. Lomberget, A. Dumoulin, R. Barret, C. Grenot, L. Rocheblave, E.-L. Matera, C. Dumontet, N. Walchshofer, *Steroids* **2012**, *77*, 1177–1191.
[23] S. Nicolai, C. Piemontesi, J. Waser, *Angew. Chem. Int. Ed.* **2011**, *50*, 4680–4683.
[24] R. B. Dateer, B. S. Shaibu, R.-S. Liu, *Angew. Chem. Int. Ed.* **2012**, *51*, 113–117.
[25] M. Chuchmareva, C. Strauch, S. Schröder, A. Collong, M. Niggemann, *Tetrahedron Lett.* **2021**, *74*, 153173.
[26] K. Osowska, T. Lis, S. Szafert, *Eur. J. Org. Chem.* **2008**, *2008*, 4598–4606.
[27] G. Suez, V. Bloch, G. Nisnevich, M. Gandelman, *Eur. J. Org. Chem.* **2012**, *2012*, 2118–2122.
[28] L. Buzzetti, M. Puriņš, P. D. G. Greenwood, J. Waser, *J. Am. Chem. Soc.* **2020**, *142*, 17334–17339.
[29] M. Amongero, T. S. Kaufman, *Tetrahedron Lett.* **2013**, *54*, 1924–1927.

I. NMR Spectra

I.1. 6-*endo-dig* carboamination products

3-(4-Methoxyphenyl)-4-methyl-2-(trifluoromethyl)-5-((triisopropylsilyl)ethynyl)-3,6-dihydro-2*H*-1,3-oxazine (5aa)

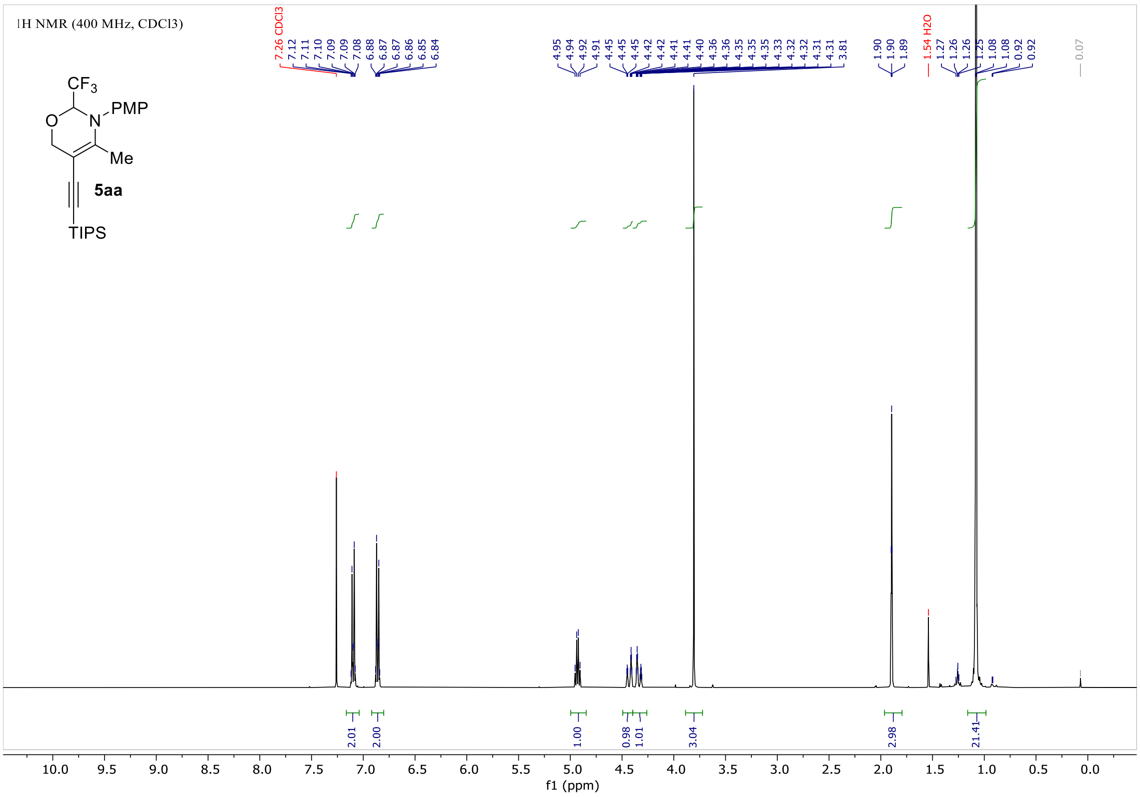

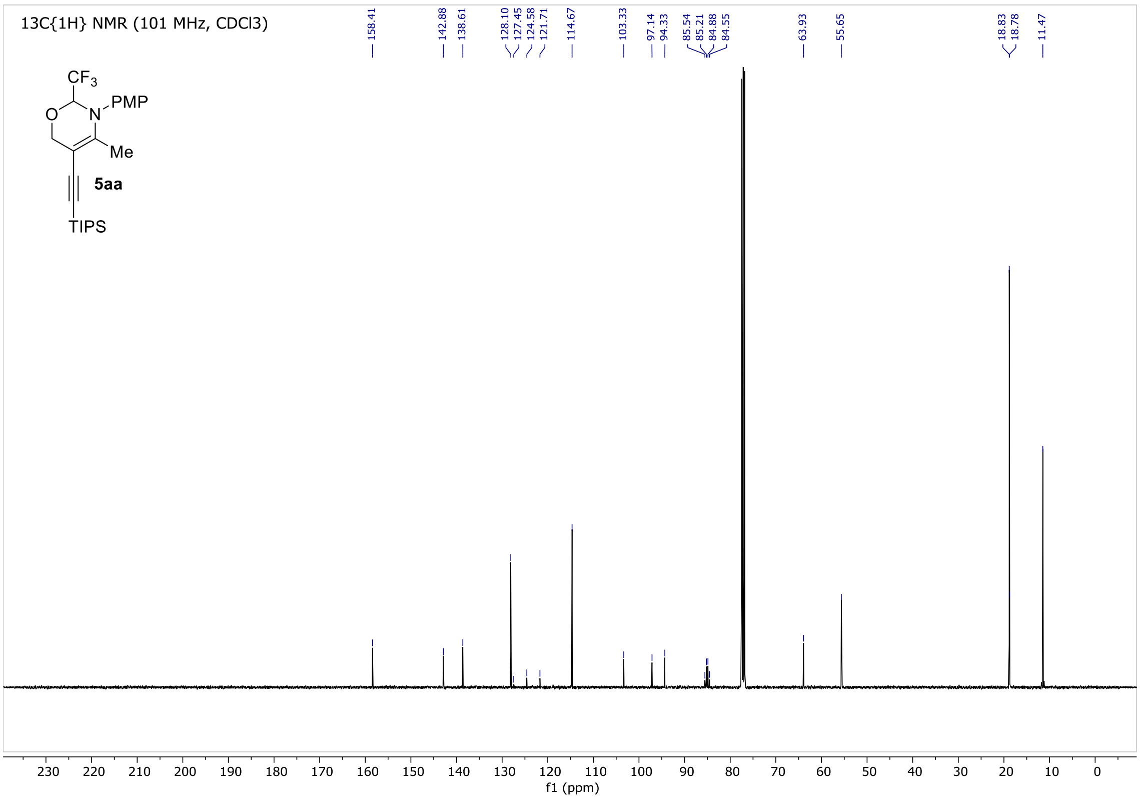

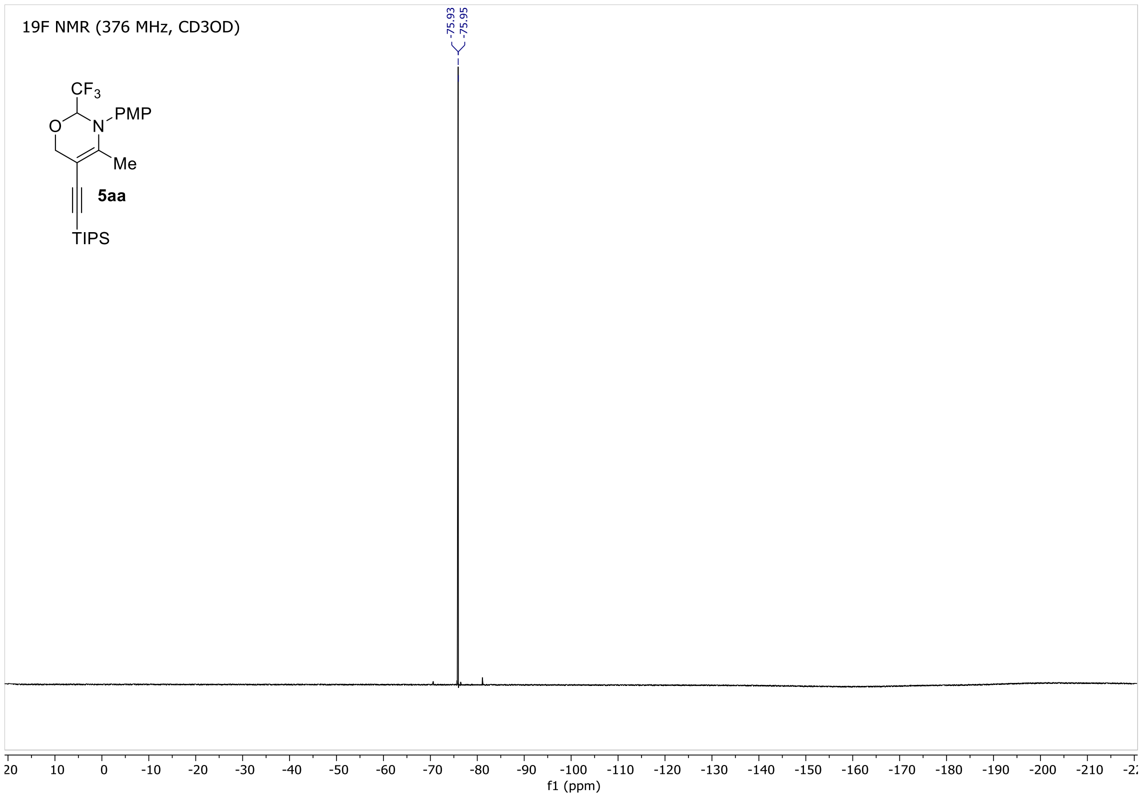

3-(4-Methoxyphenyl)-4-phenyl-2-(trifluoromethyl)-5-((triisopropylsilyl)ethynyl)-3,6-dihydro-2*H*-1,3-oxazine (5ba)

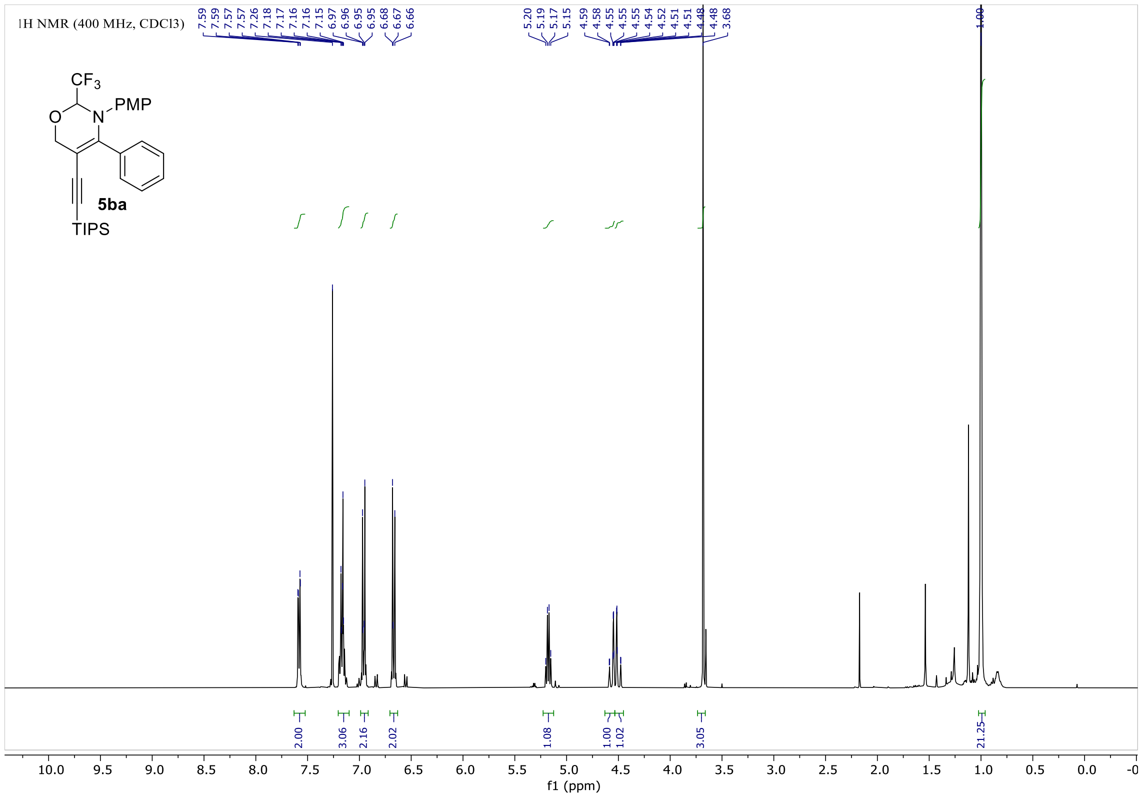

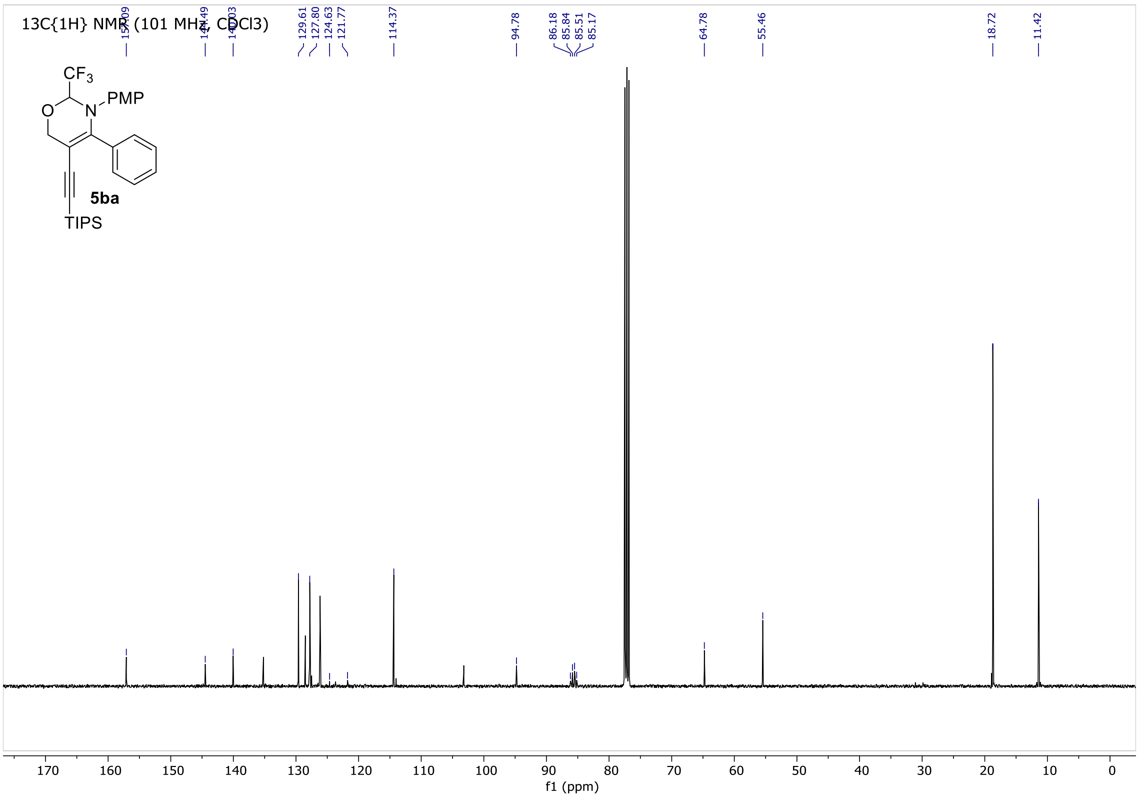
13C{1H} NMR (101 MHz, CDCl3)
CF3
PMP
5ba
TIPS
129.61
127.80
124.63
121.77
114.37
94.78
86.18
85.84
85.51
85.17
64.78
55.46
18.72
11.42
f1 (ppm)

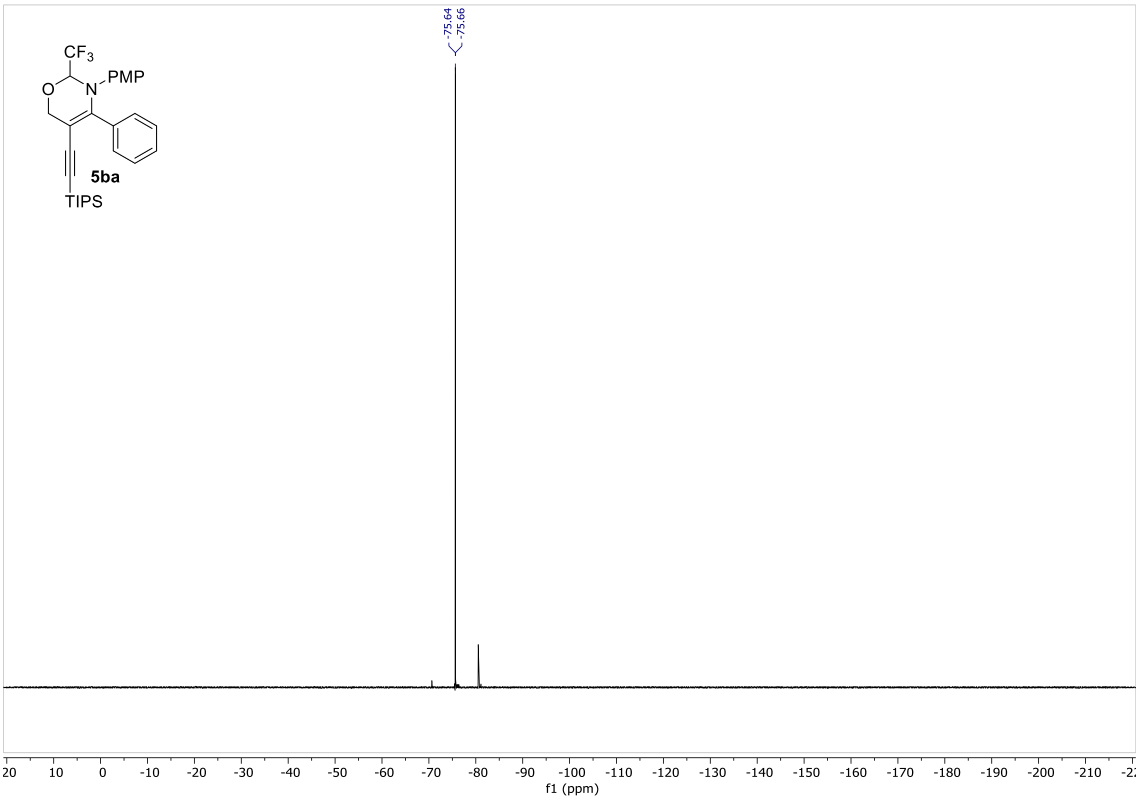
-75.64
-75.66
CF3
PMP
5ba
TIPS
f1 (ppm)

3,4-Bis(4-methoxyphenyl)-2-(trifluoromethyl)-5-((triisopropylsilyl)ethynyl)-3,6-dihydro-2*H*-1,3-oxazine (5ca)

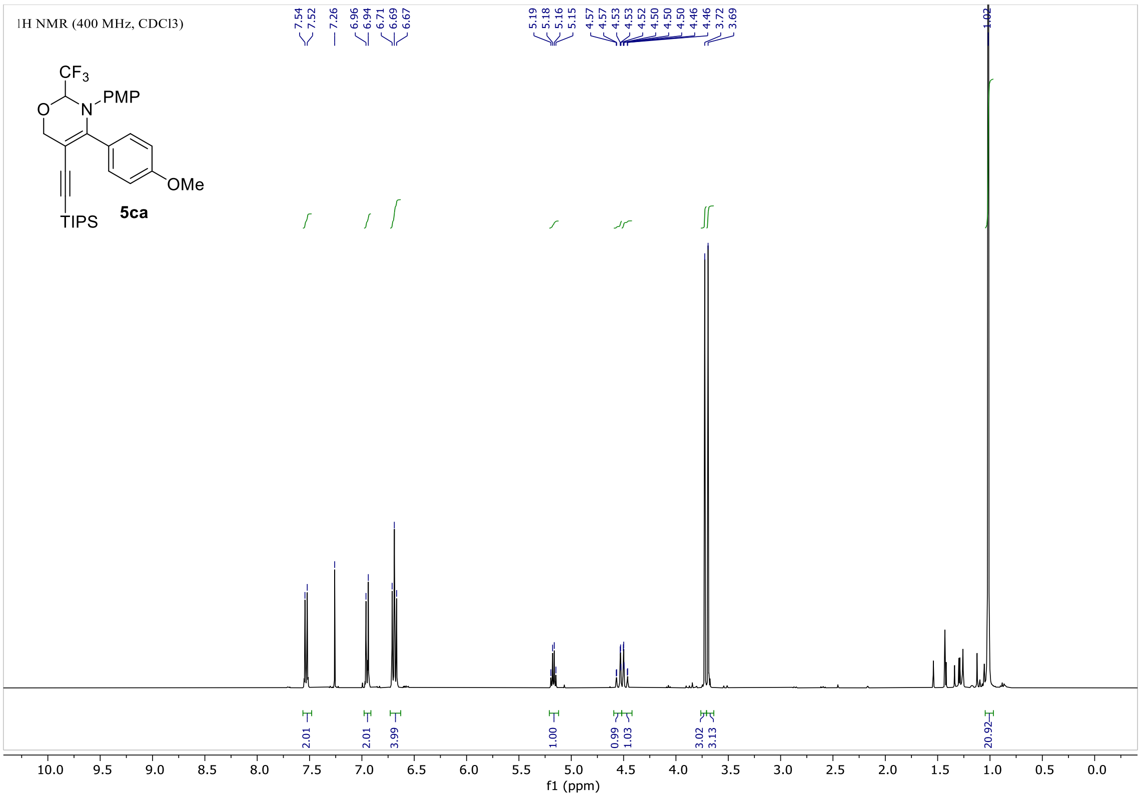

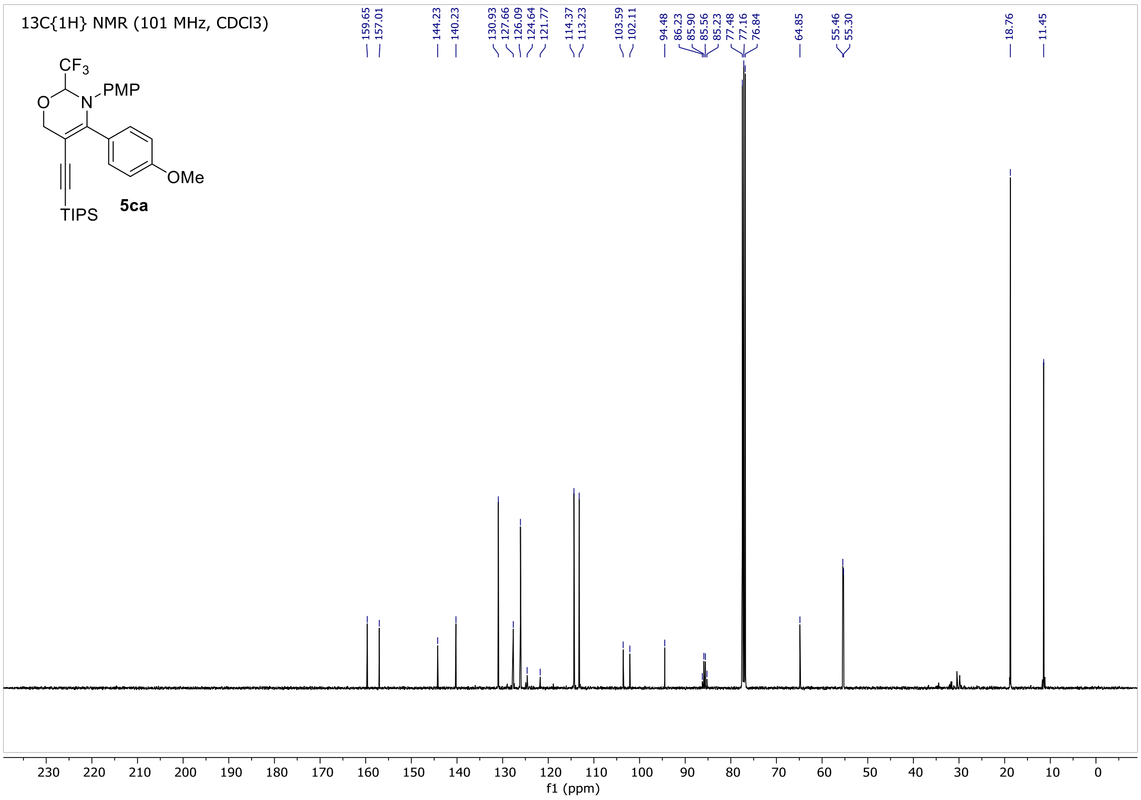

3-(4-Methoxyphenyl)-4-(4-fluorophenyl)-2-(trifluoromethyl)-5-((triisopropylsilyl)ethynyl)-3,6-dihydro-2*H*-1,3-oxazine (5da)

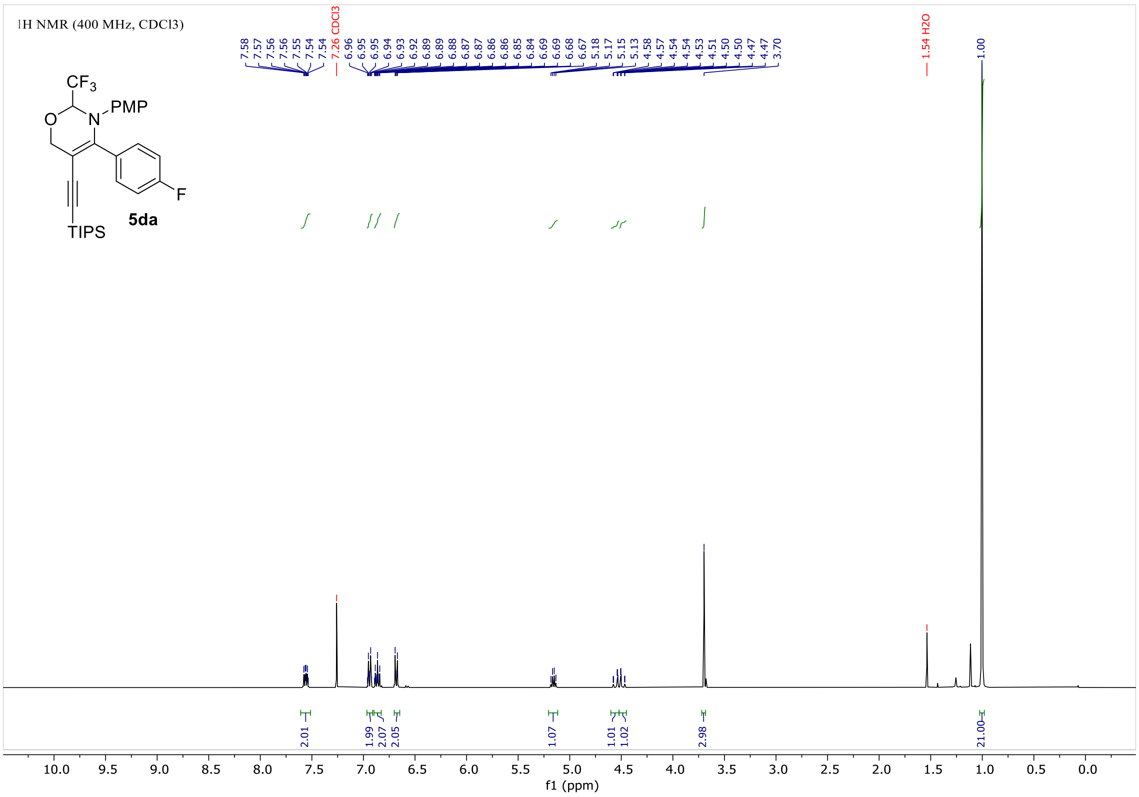

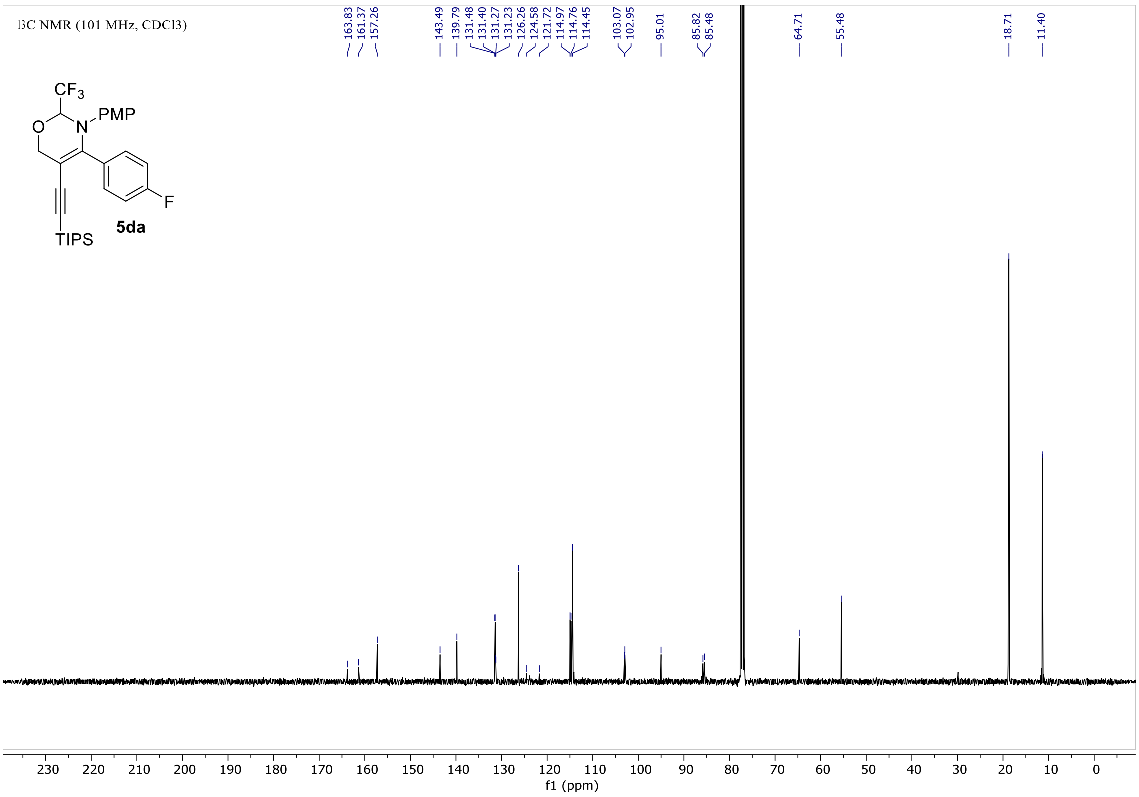

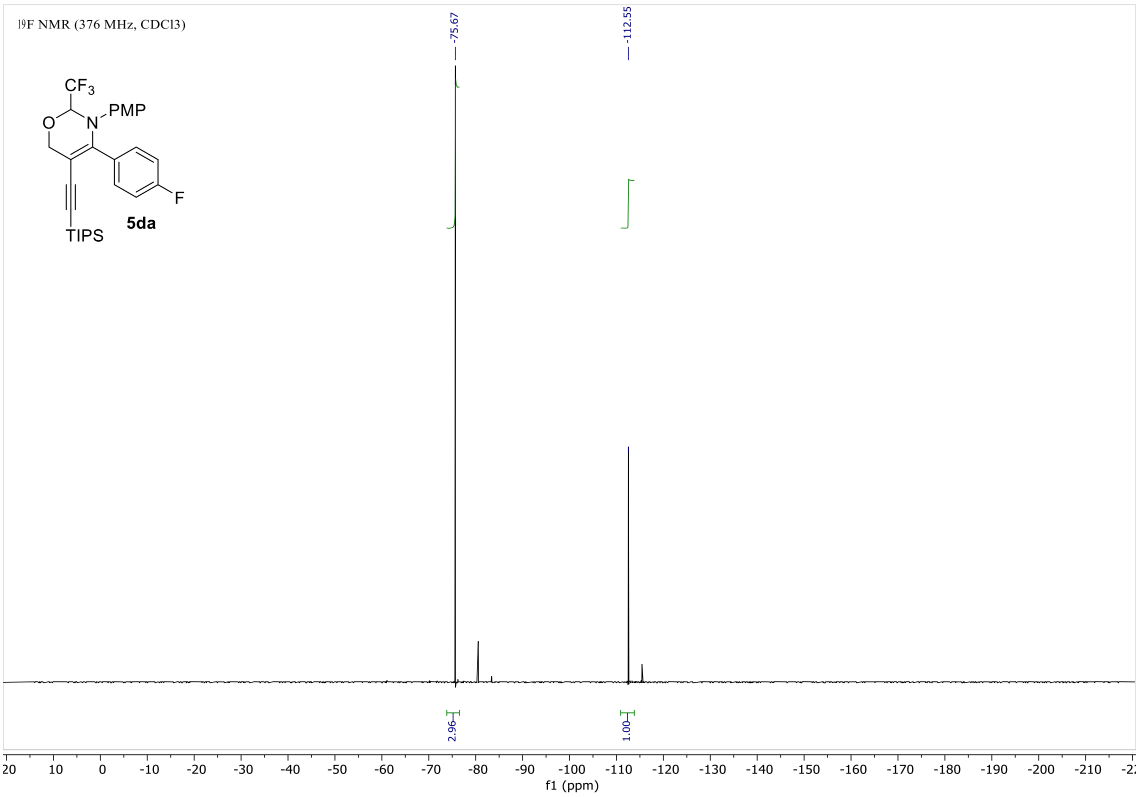

3-(4-Methoxyphenyl)-4-(4-chlorophenyl)-2-(trifluoromethyl)-5-((triisopropylsilyl)ethynyl)-3,6-dihydro-2*H*-1,3-oxazine (5ea)

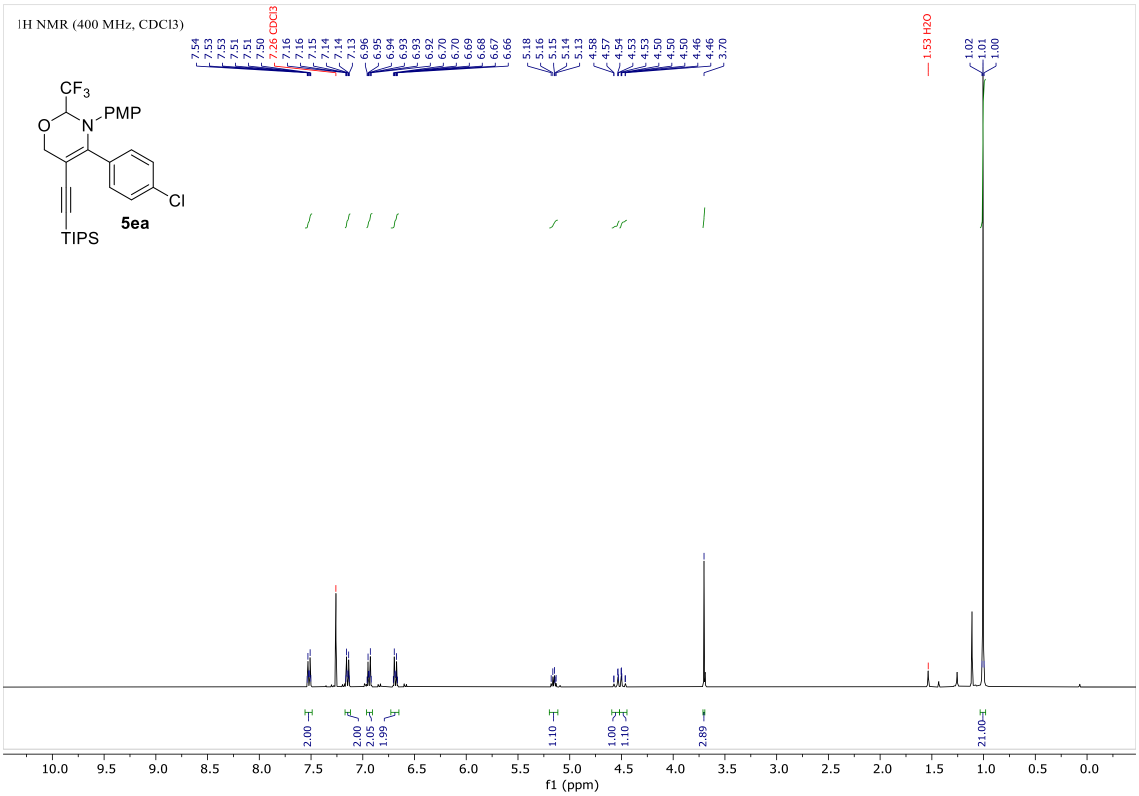

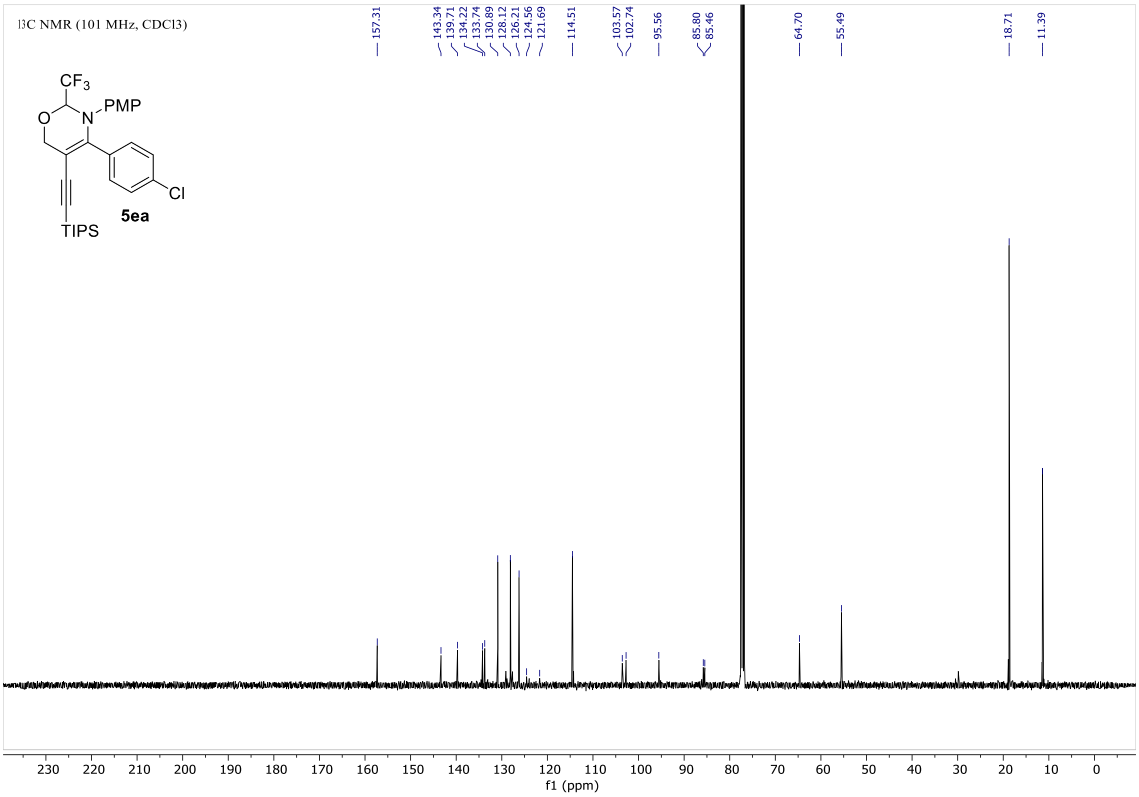
13C NMR (101 MHz, CDCl3)
157.31
143.34
139.71
134.22
133.74
130.89
128.12
126.21
124.56
121.69
114.51
103.57
102.74
95.56
85.80
85.46
64.70
55.49
18.71
11.39
CF3
O
N
PMP
Cl
TIPS
5ea
230 220 210 200 190 180 170 160 150 140 130 120 110 100 90 80 70 60 50 40 30 20 10 0
f1 (ppm)

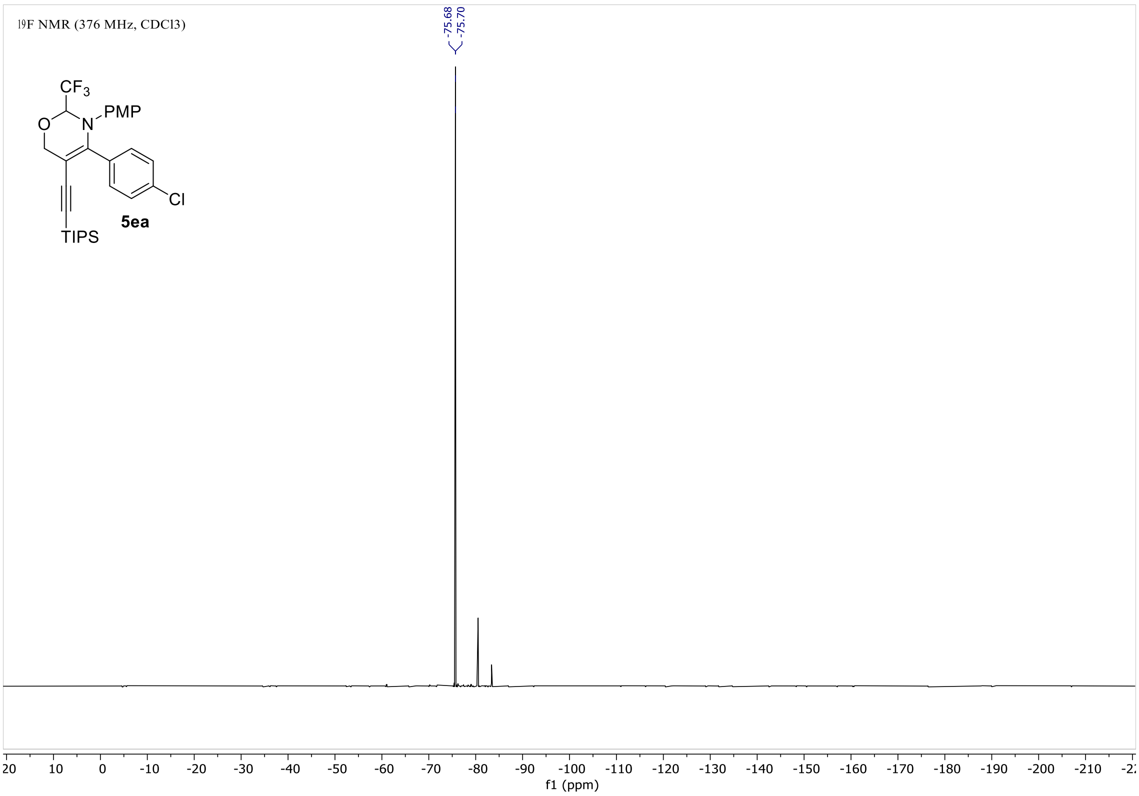
19F NMR (376 MHz, CDCl3)
-75.68
-75.70
CF3
O
N
PMP
Cl
TIPS
5ea
20 10 0 -10 -20 -30 -40 -50 -60 -70 -80 -90 -100 -110 -120 -130 -140 -150 -160 -170 -180 -190 -200 -210
f1 (ppm)

3-(4-Methoxyphenyl)-4-(4-(trifluoromethyl)phenyl)-2-(trifluoromethyl)-5-((triisopropylsilyl)ethynyl)-3,6-dihydro-2*H*-1,3-oxazine (5fa)

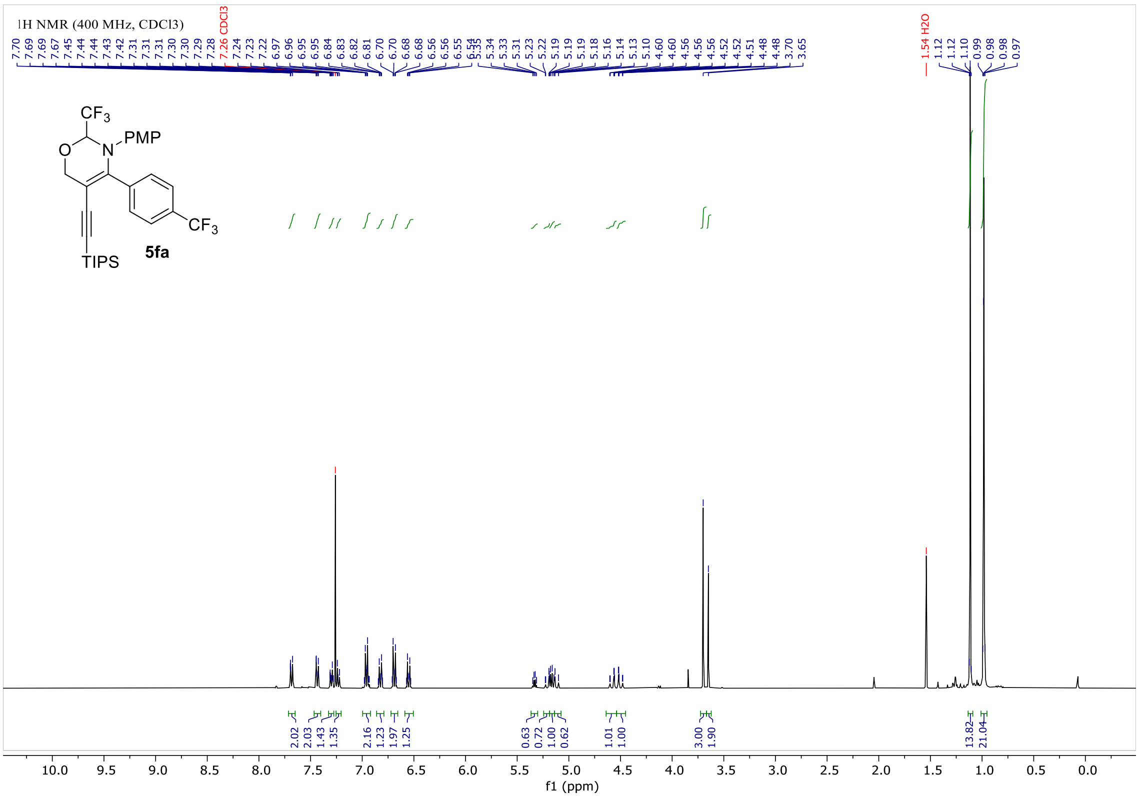

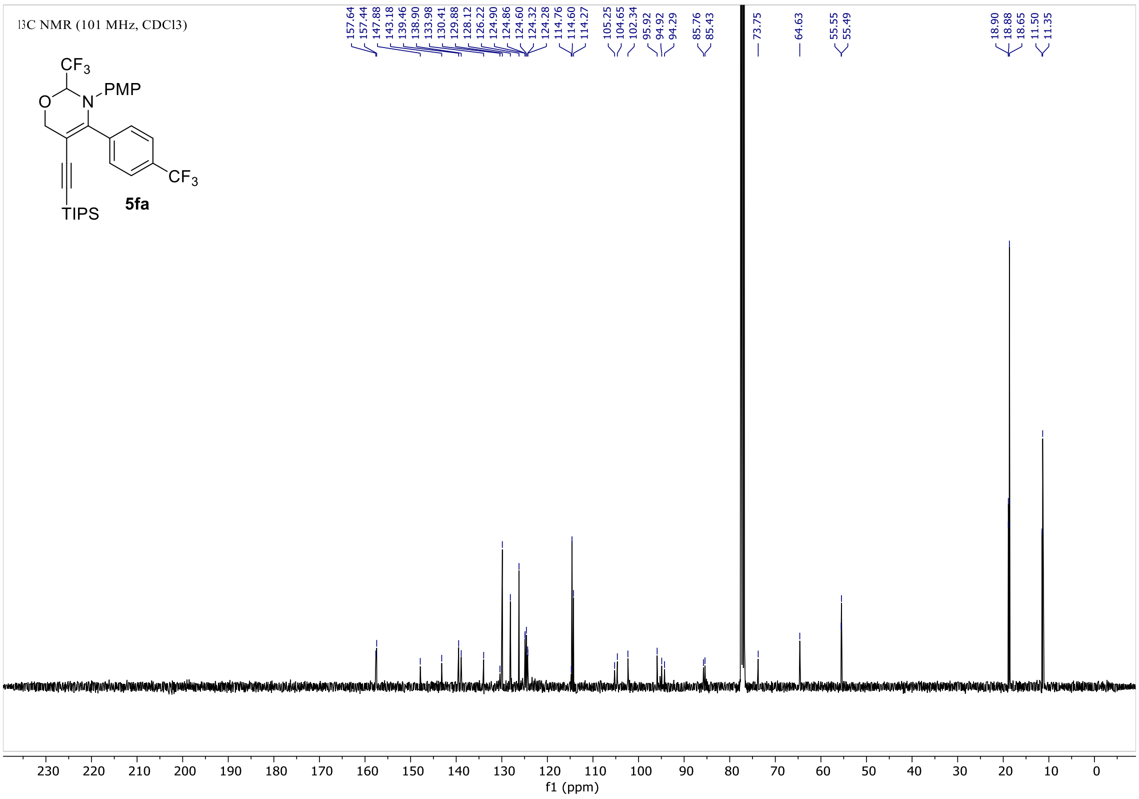

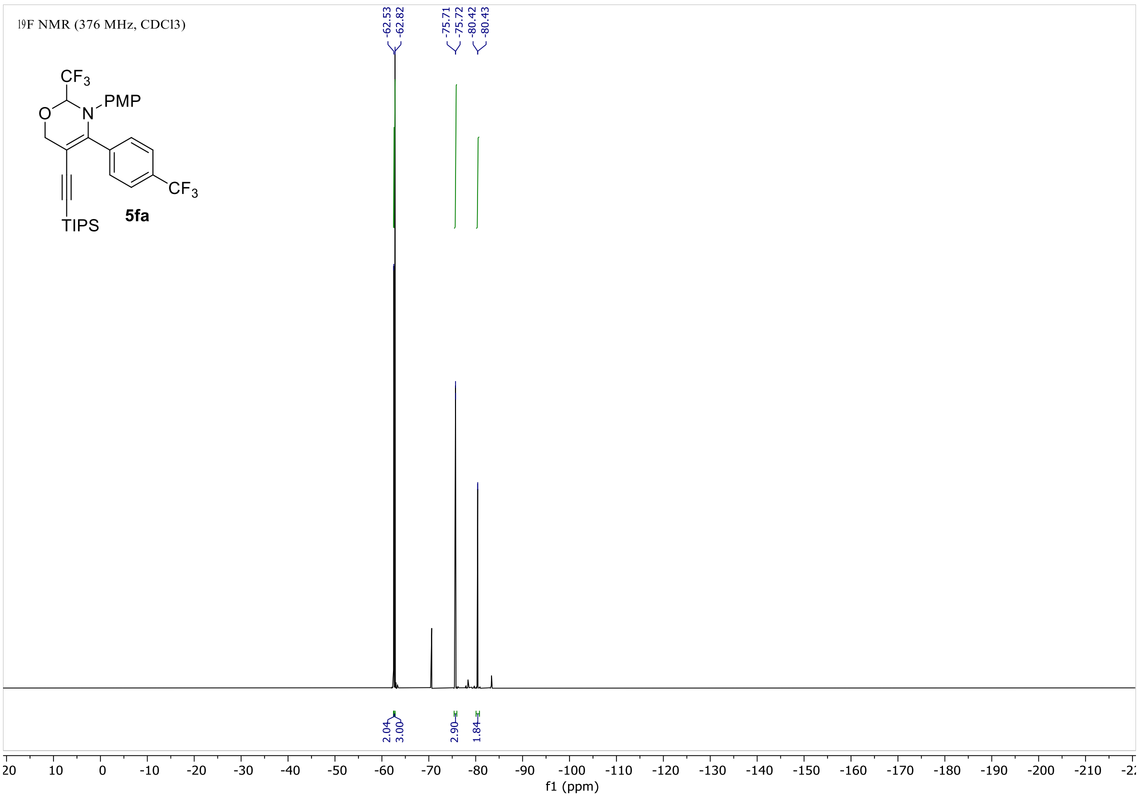

3-(4-Methoxyphenyl)-4-(3-fluorophenyl)-2-(trifluoromethyl)-5-((triisopropylsilyl)ethynyl)-3,6-dihydro-2*H*-1,3-oxazine (5ga)

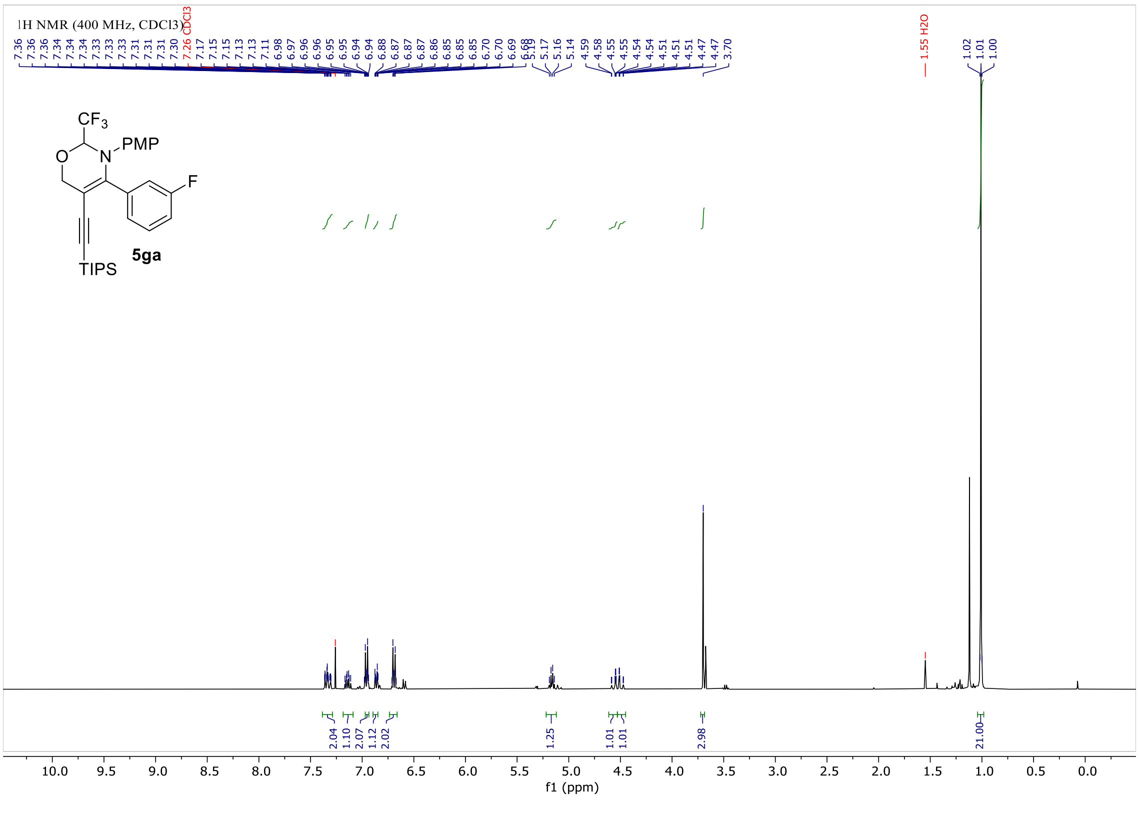

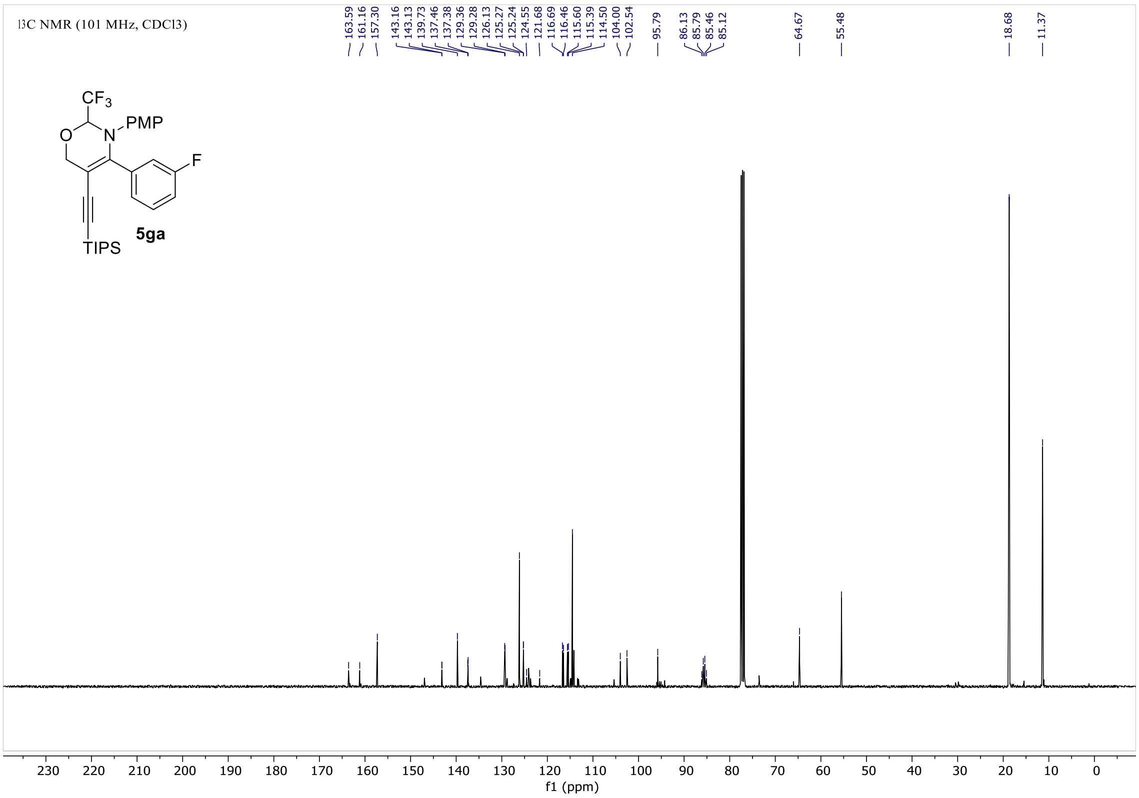
13C NMR (101 MHz, CDCl3)
163.59
161.16
157.30
143.16
143.13
139.73
137.46
137.38
129.36
129.28
126.13
125.27
125.24
124.55
121.68
116.69
116.46
115.60
115.39
114.50
104.00
102.54
95.79
86.13
85.79
85.46
85.12
64.67
55.48
18.68
11.37
CF3
PMP
O
N
F
TIPS
5ga
f1 (ppm)

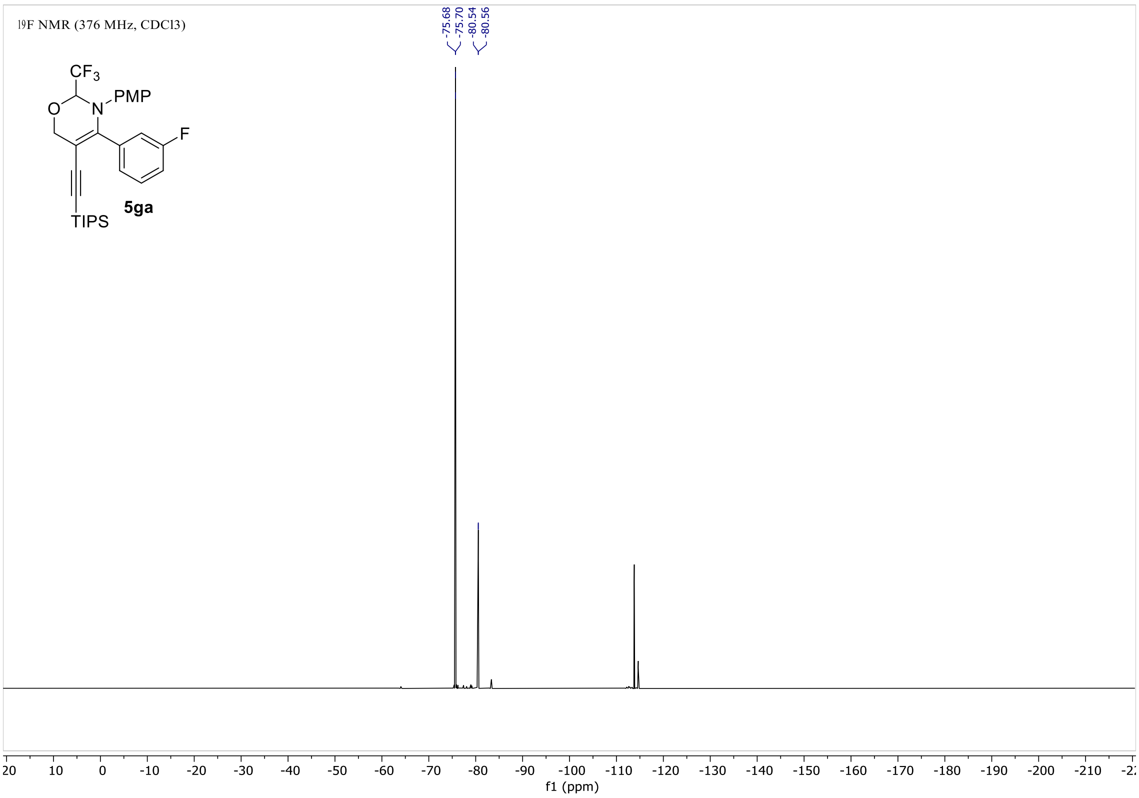
19F NMR (376 MHz, CDCl3)
-75.68
-75.70
-80.54
-80.56
CF3
PMP
O
N
F
TIPS
5ga
f1 (ppm)

3-(4-Methoxyphenyl)-4-(5-methylthiophen-2-yl)-2-(trifluoromethyl)-5-((triisopropylsilyl)ethynyl)-3,6-dihydro-2*H*-1,3-oxazine (5ha)

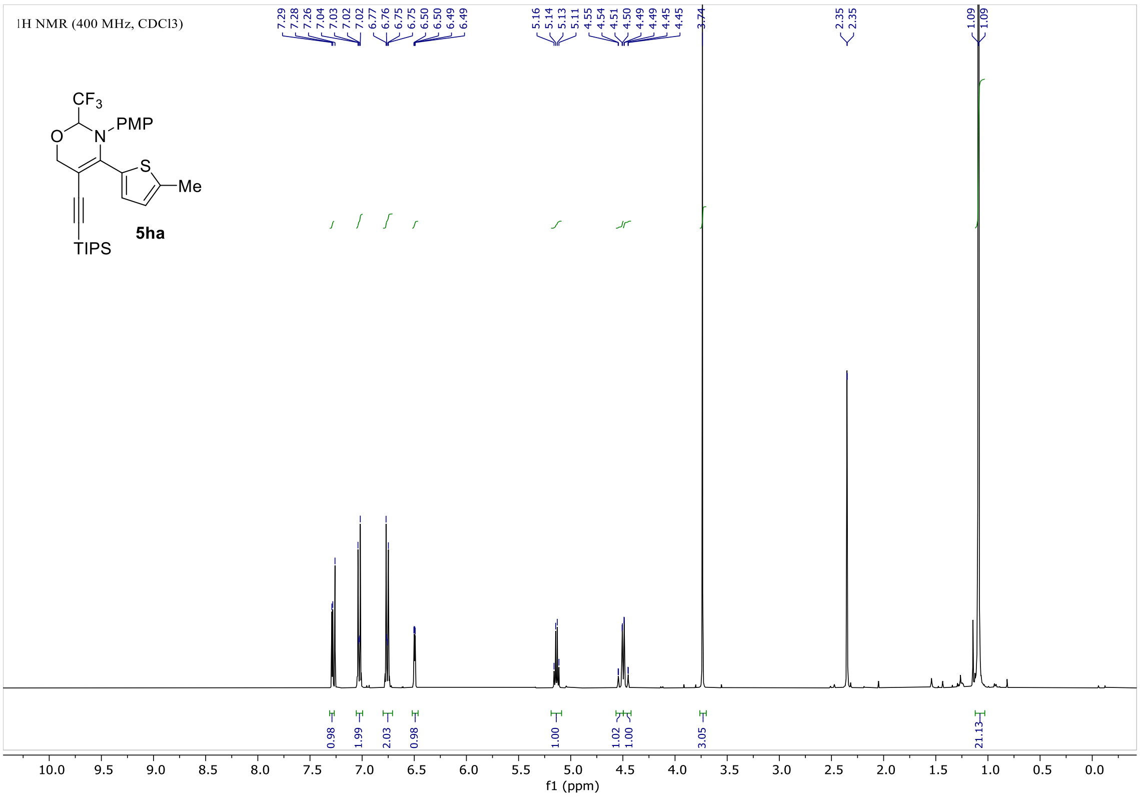

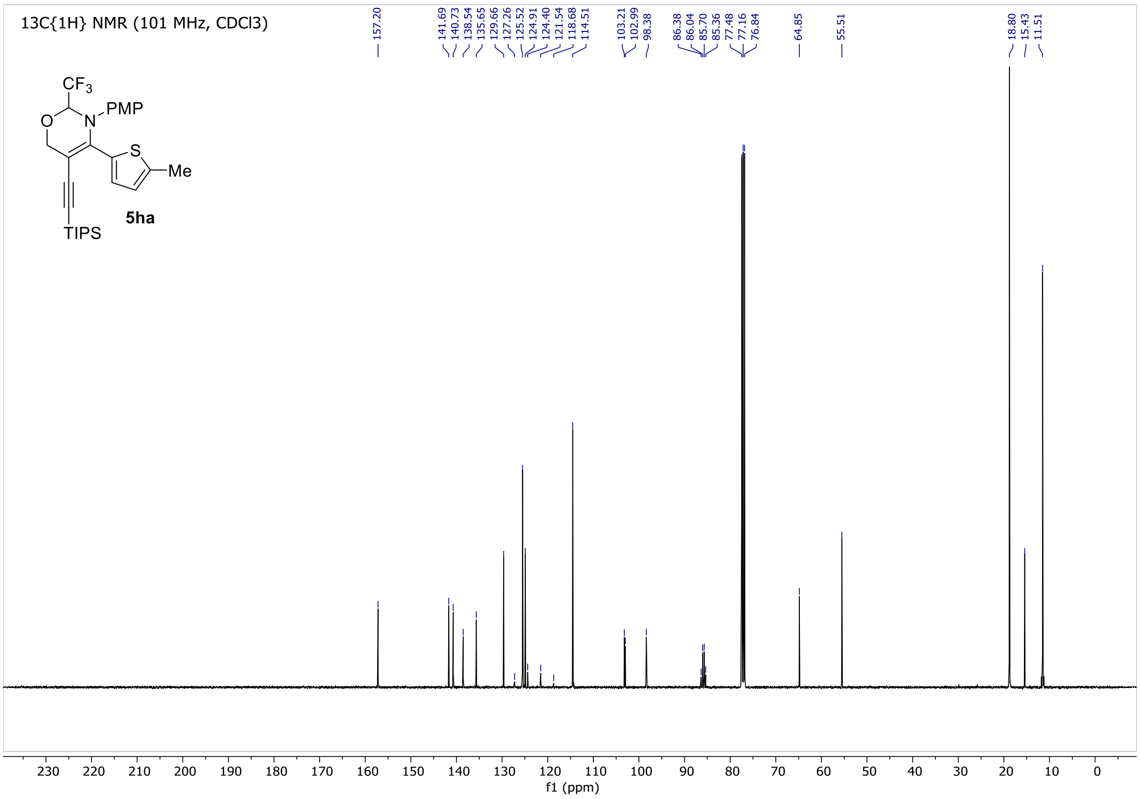

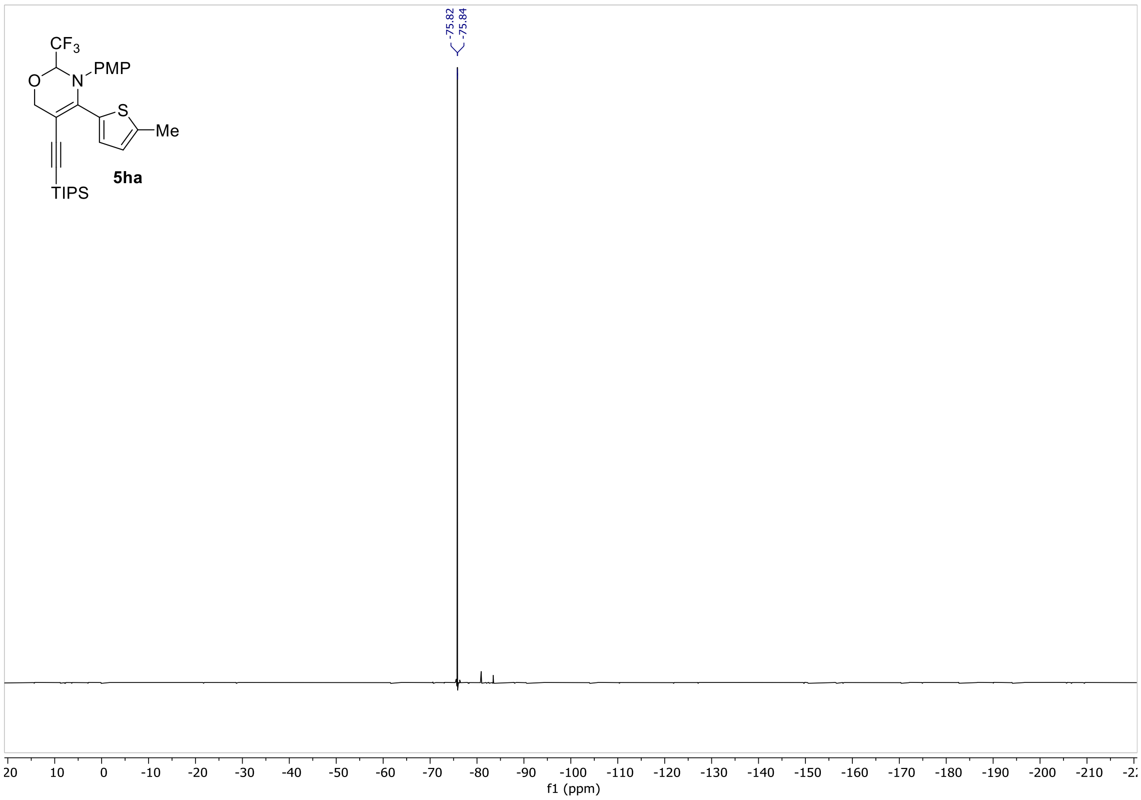

4-Cyclopropyl-3-(4-methoxyphenyl)-2-(trifluoromethyl)-5-((triisopropylsilyl)ethynyl)-3,6-dihydro-2*H*-1,3-oxazine (5ia)

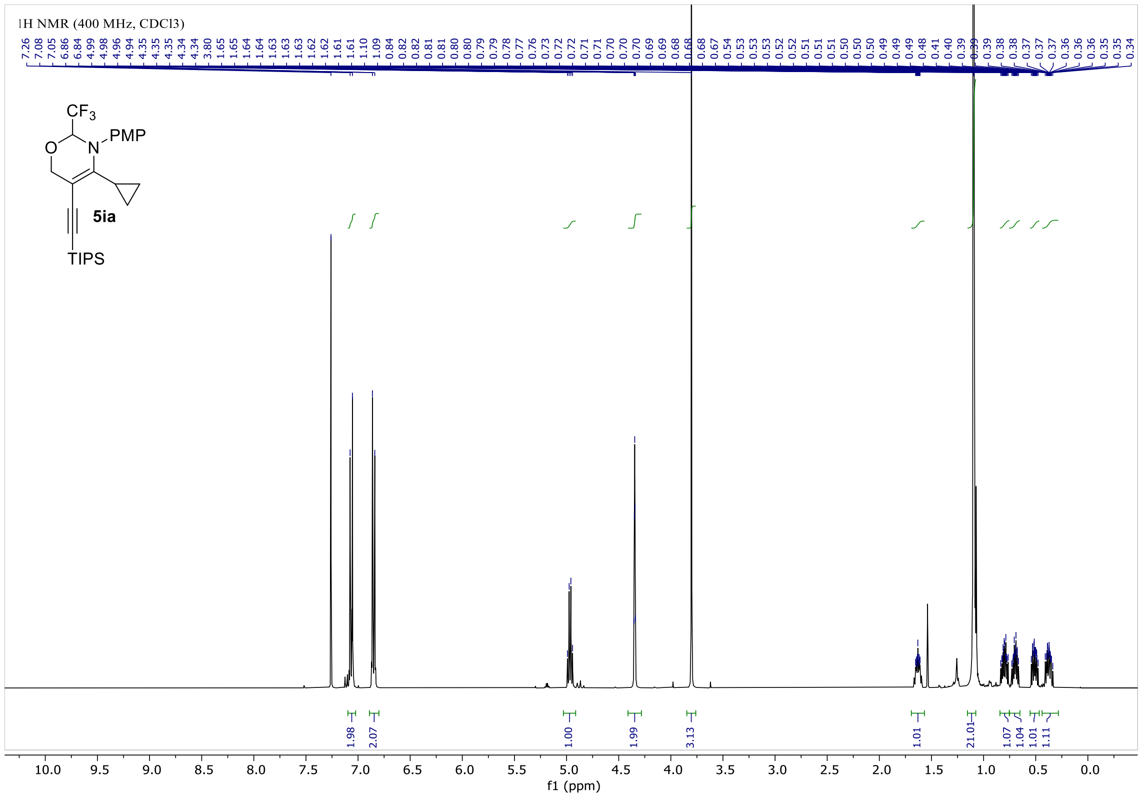

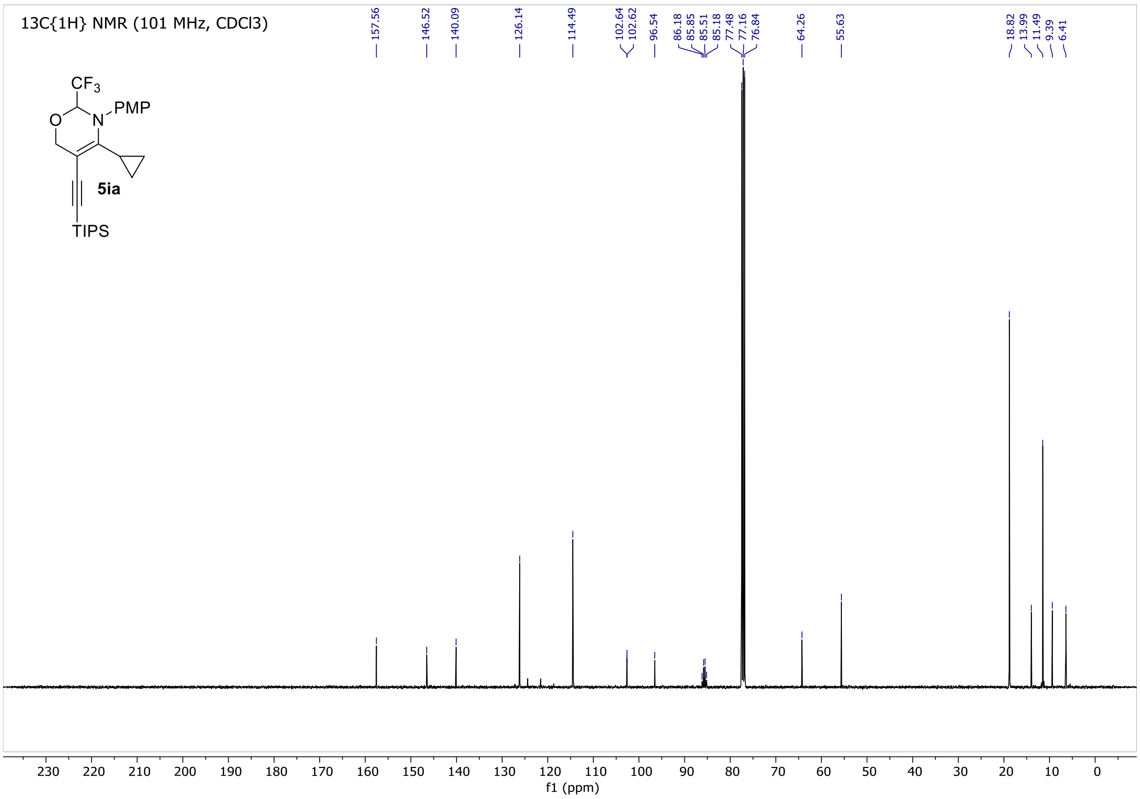
13C{1H} NMR (101 MHz, CDCl3)
157.56
146.52
140.09
126.14
114.49
102.64
102.62
96.54
86.18
85.85
85.51
85.18
77.48
77.16
76.84
64.26
55.63
18.82
13.99
11.49
9.39
6.41
CF3
PMP
O
N
5ia
TIPS
230 220 210 200 190 180 170 160 150 140 130 120 110 100 90 80 70 60 50 40 30 20 10 0
f1 (ppm)

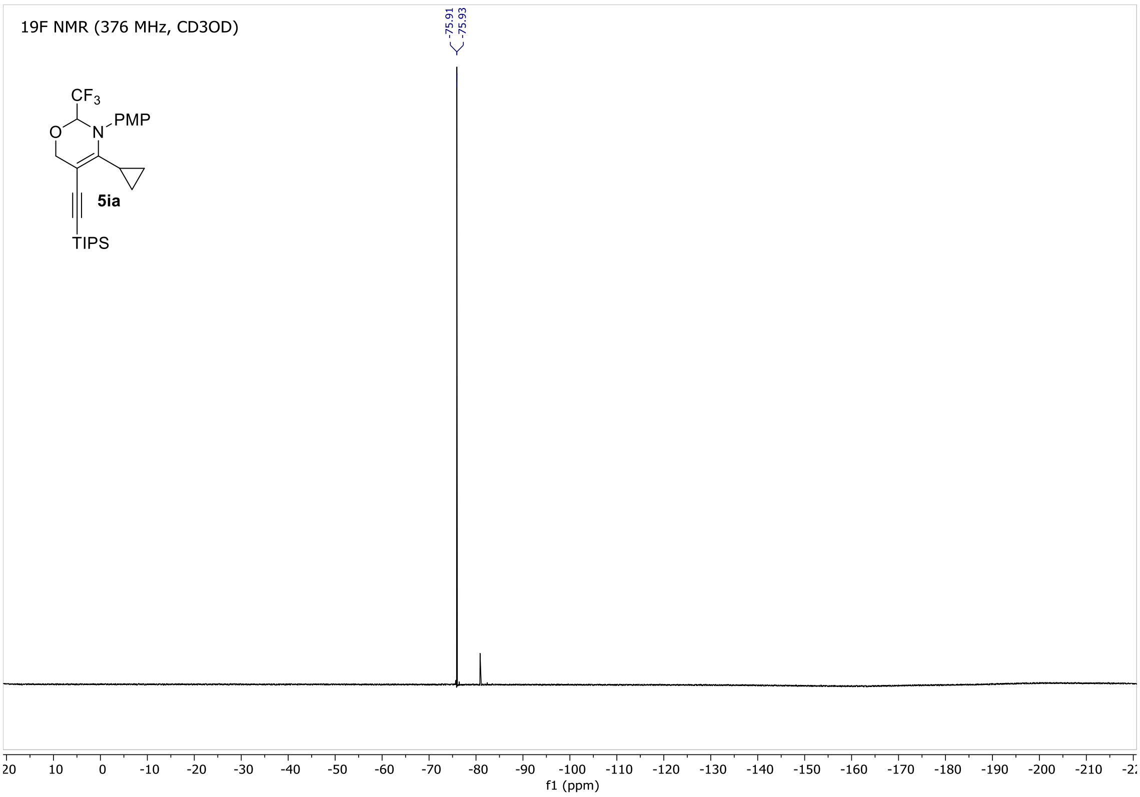
19F NMR (376 MHz, CD3OD)
-75.91
-75.93
CF3
PMP
O
N
5ia
TIPS
20 10 0 -10 -20 -30 -40 -50 -60 -70 -80 -90 -100 -110 -120 -130 -140 -150 -160 -170 -180 -190 -200 -210
f1 (ppm)

4-(((*tert*-Butyldimethylsilyl)oxy)methyl)-3-(4-methoxyphenyl)-2-(trifluoromethyl)-5-((triisopropylsilyl)ethynyl)-3,6-dihydro-2*H*-1,3-oxazine (5ja)

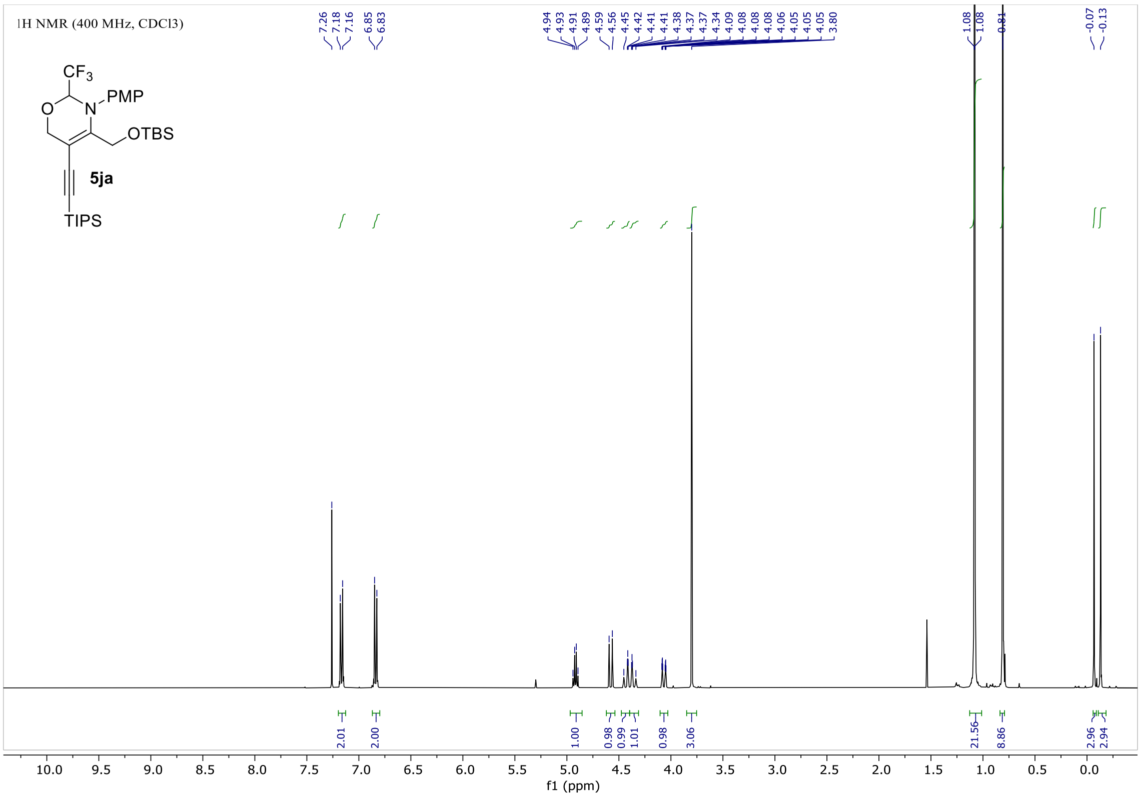

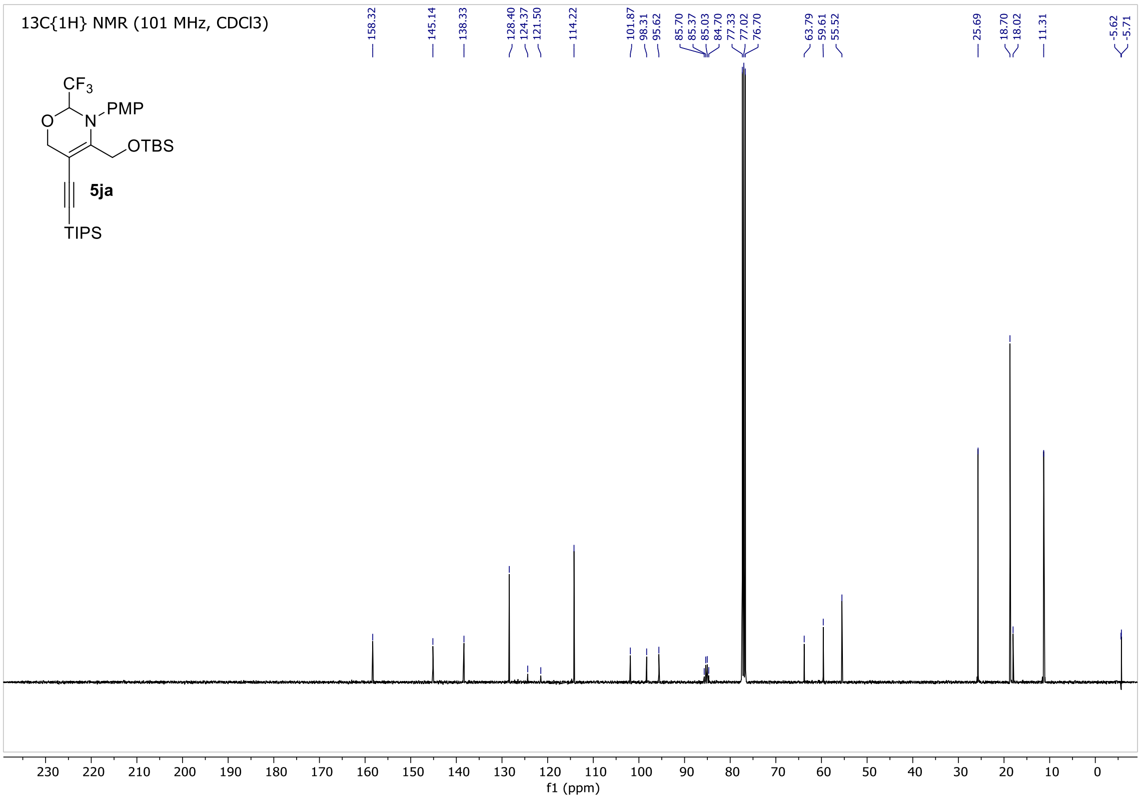

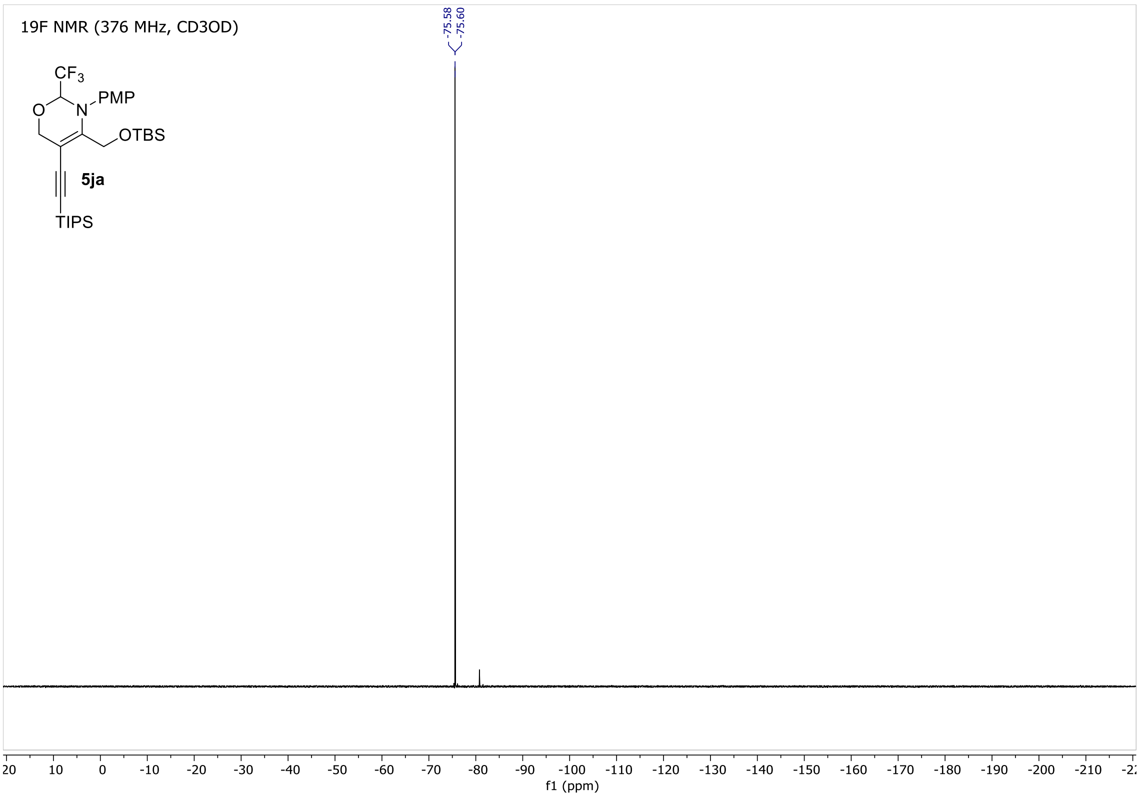

3-(4-Methoxyphenyl)-2-(trifluoromethyl)-5-((triisopropylsilyl)ethynyl)-4-vinyl-3,6-dihydro-2*H*-1,3-oxazine (5ka)

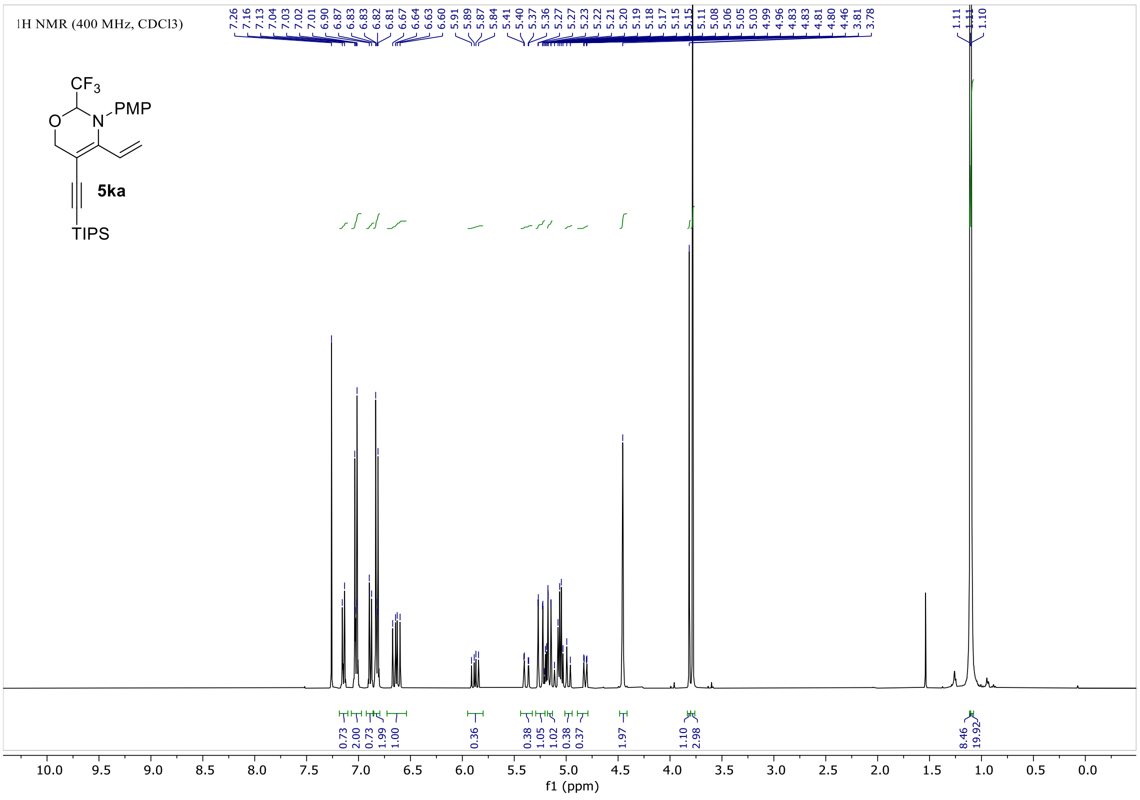

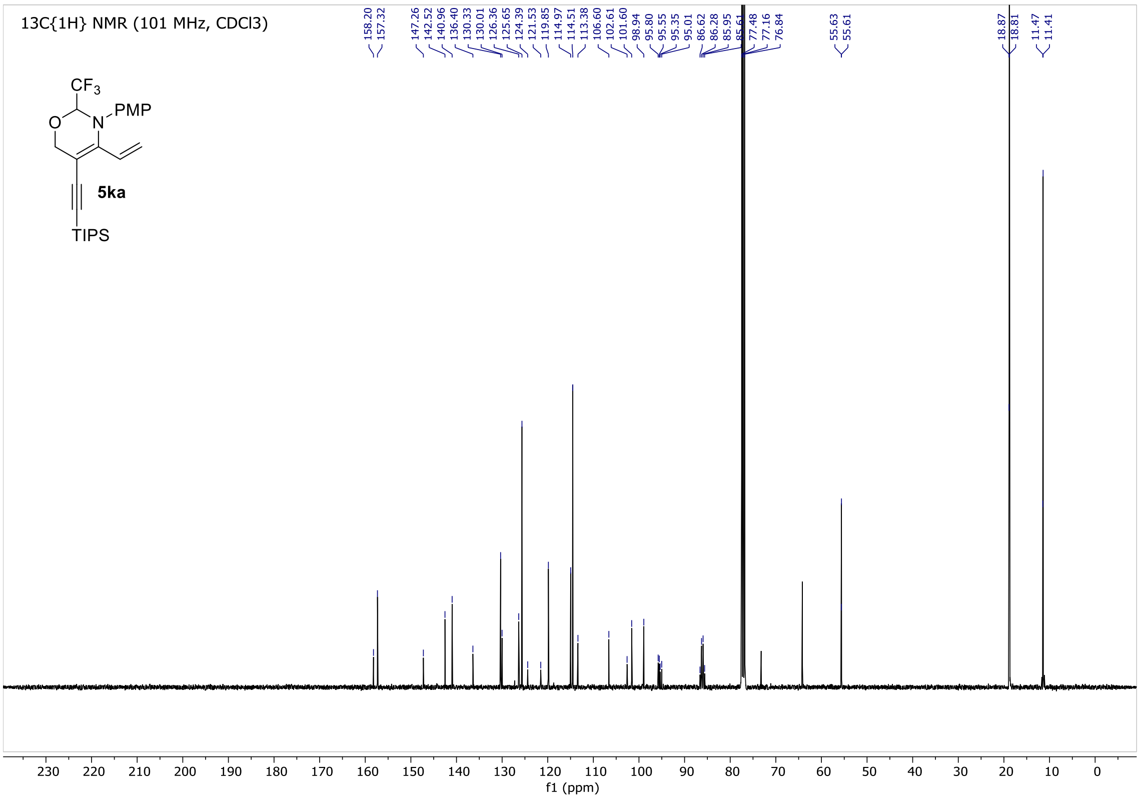
13C{1H} NMR (101 MHz, CDCl3)
158.20
157.32
147.26
142.52
140.96
136.40
130.33
130.01
126.36
125.65
124.39
121.53
119.85
114.97
114.51
113.38
106.60
102.61
101.60
98.94
95.80
95.55
95.35
95.01
86.62
86.28
85.95
85.61
77.48
77.16
76.84
55.63
55.61
18.87
18.81
11.47
11.41
CF3
O
N
PMP
5ka
TIPS
230
220
210
200
190
180
170
160
150
140
130
120
110
100
90
80
70
60
50
40
30
20
10
0
f1 (ppm)

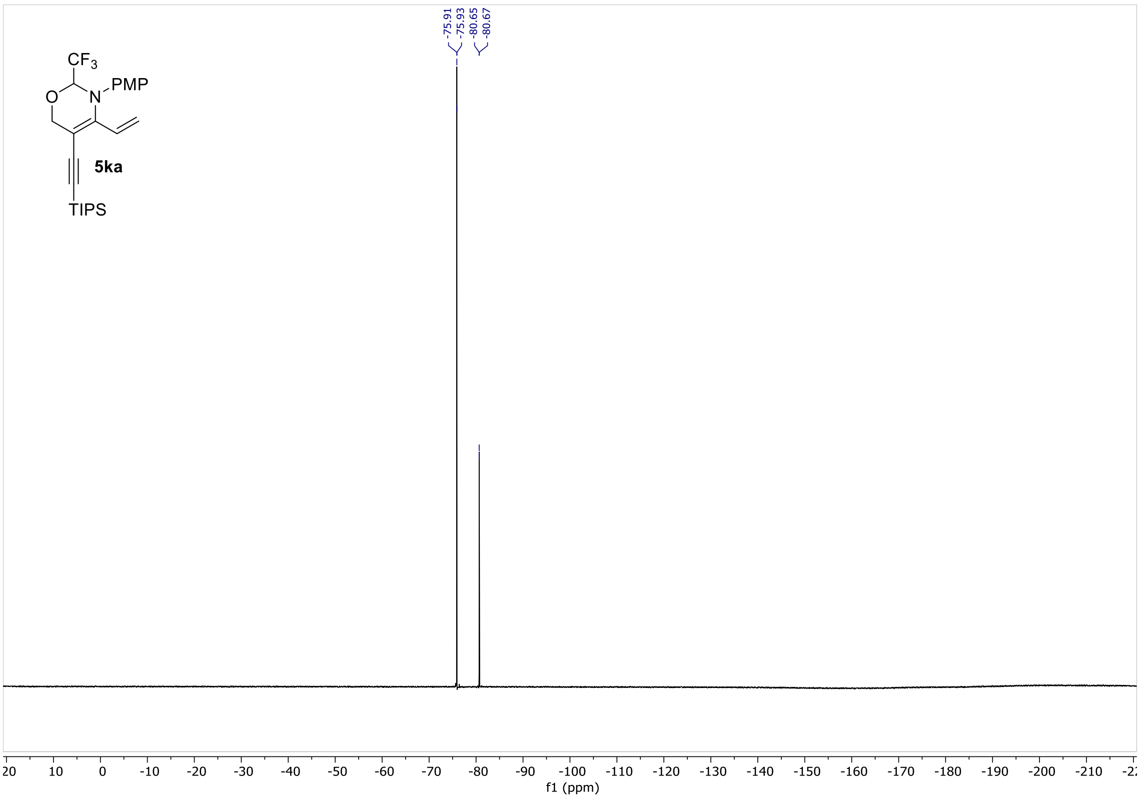
-75.91
-75.93
-80.65
-80.67
CF3
O
N
PMP
5ka
TIPS
20
10
0
-10
-20
-30
-40
-50
-60
-70
-80
-90
-100
-110
-120
-130
-140
-150
-160
-170
-180
-190
-200
-210
f1 (ppm)

3-(4-Methoxyphenyl)-4,6-dimethyl-2-(trifluoromethyl)-5-((triisopropylsilyl)ethynyl)-3,6-dihydro-2*H*-1,3-oxazine (5la)

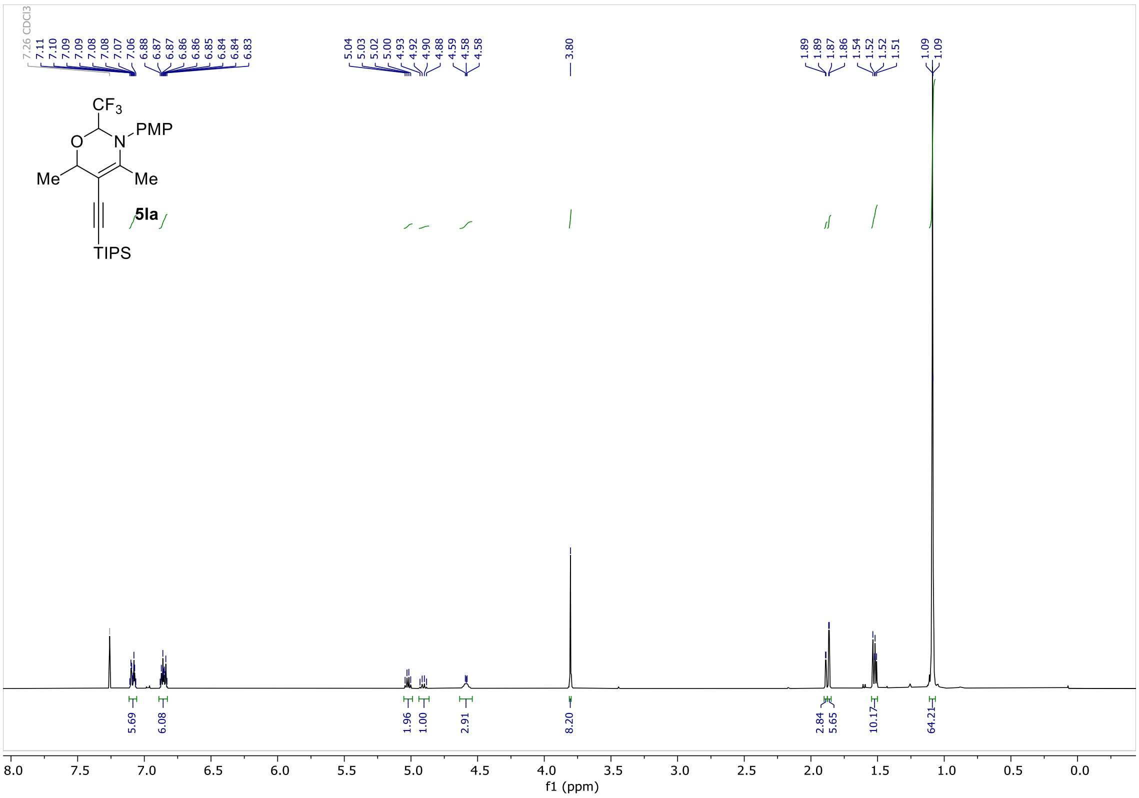

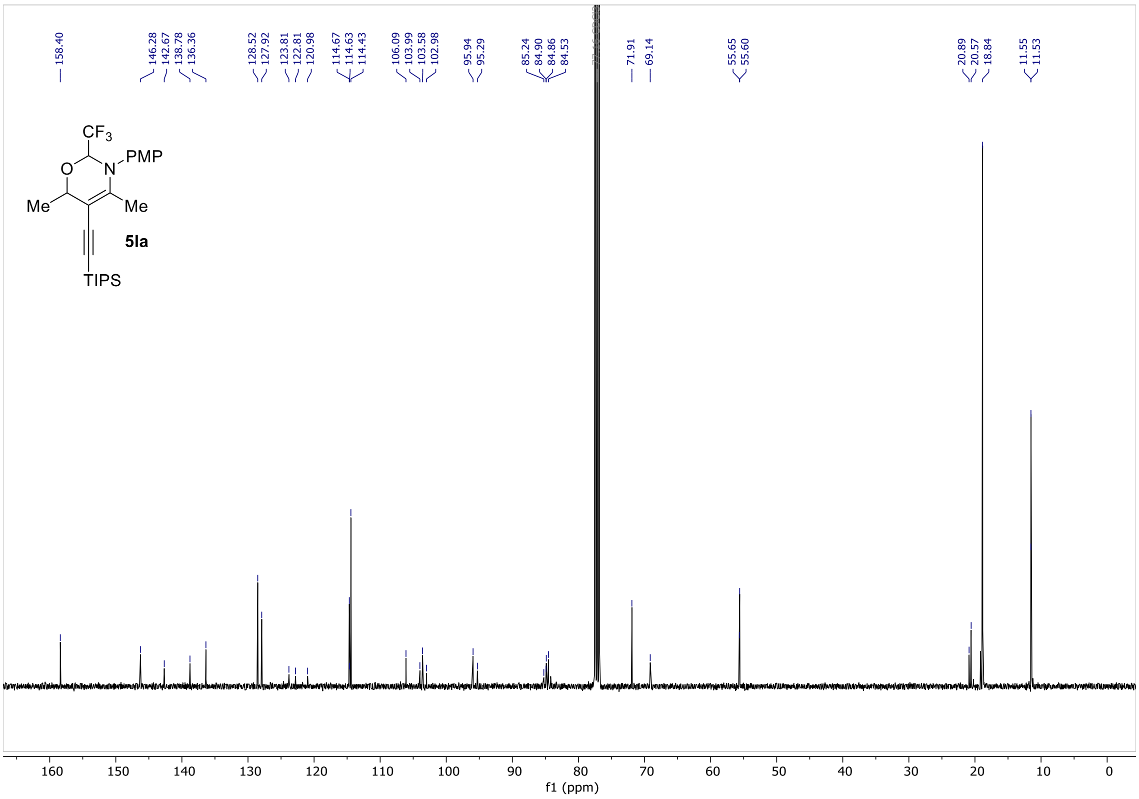

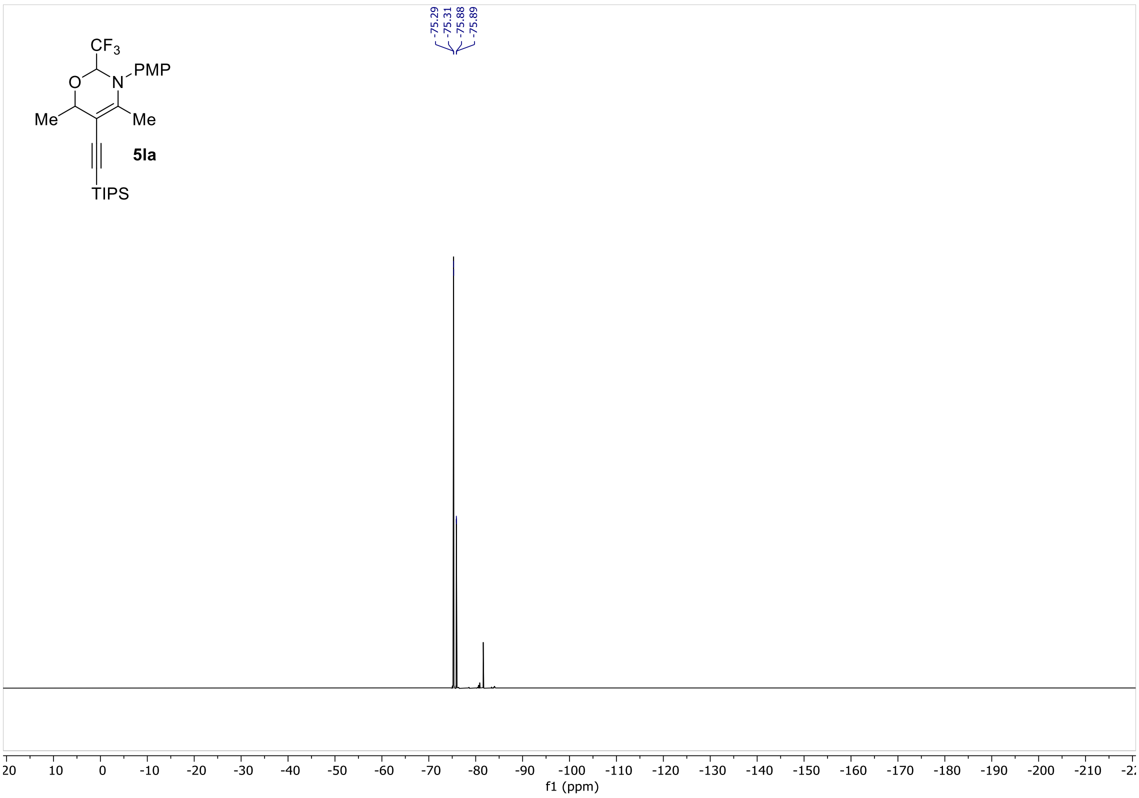

3-(4-Methoxyphenyl)-4,6,6-trimethyl-2-(trifluoromethyl)-5-((triisopropylsilyl)ethynyl)-3,6-dihydro-2*H*-1,3-oxazine (5ma)

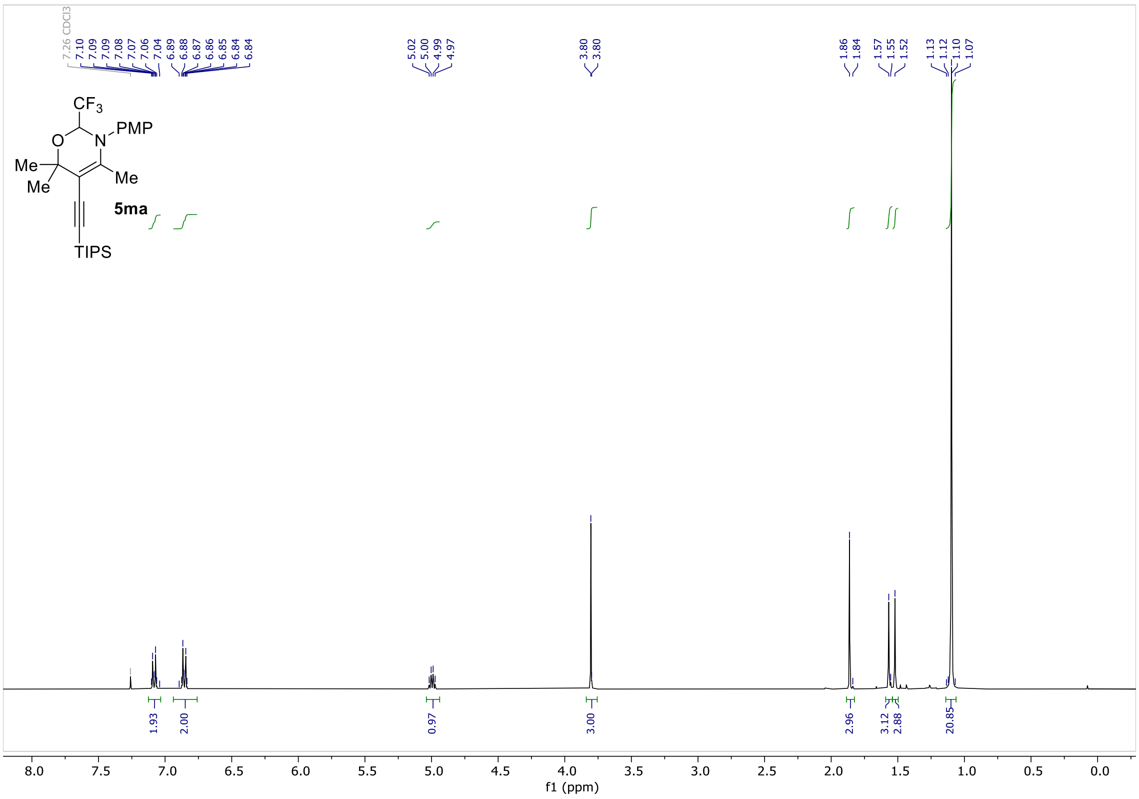

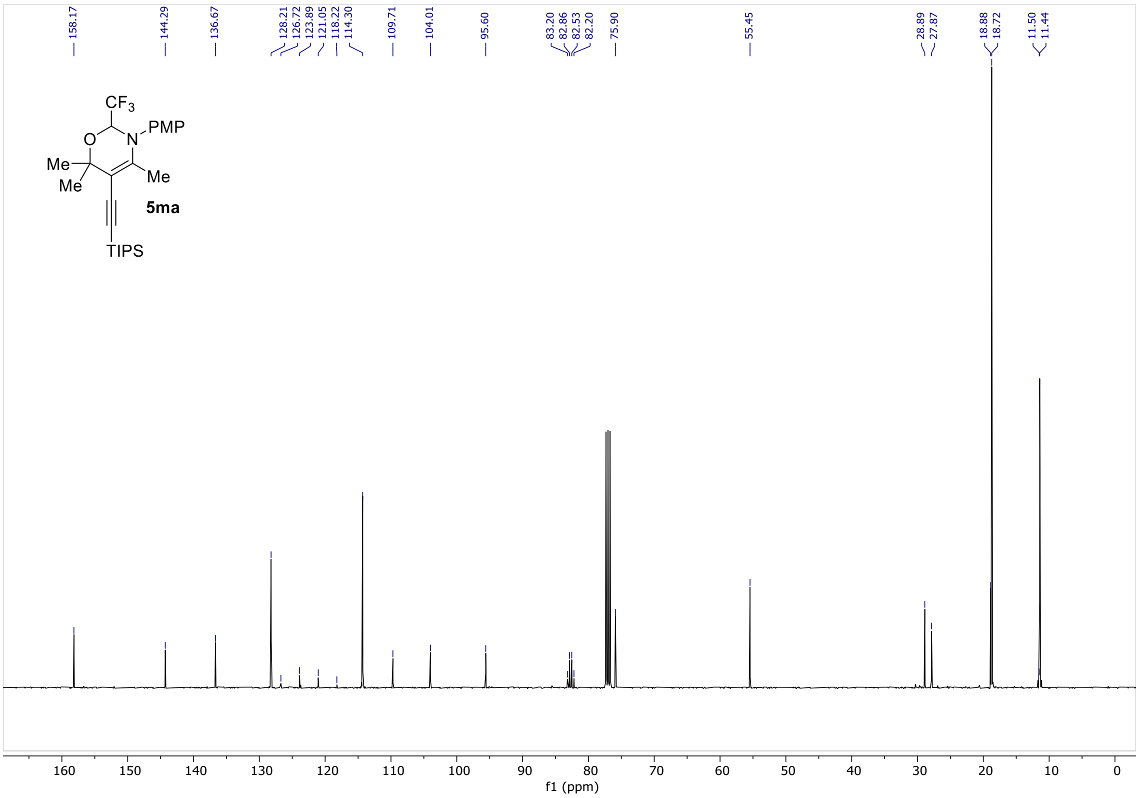
158.17
144.29
136.67
128.21
126.72
123.89
121.05
118.22
114.30
109.71
104.01
95.60
83.20
82.86
82.53
82.20
75.90
55.45
28.89
27.87
18.88
18.72
11.50
11.44
CF3
O
N
PMP
Me
Me
Me
5ma
TIPS
160 150 140 130 120 110 100 90 80 70 60 50 40 30 20 10 0
f1 (ppm)

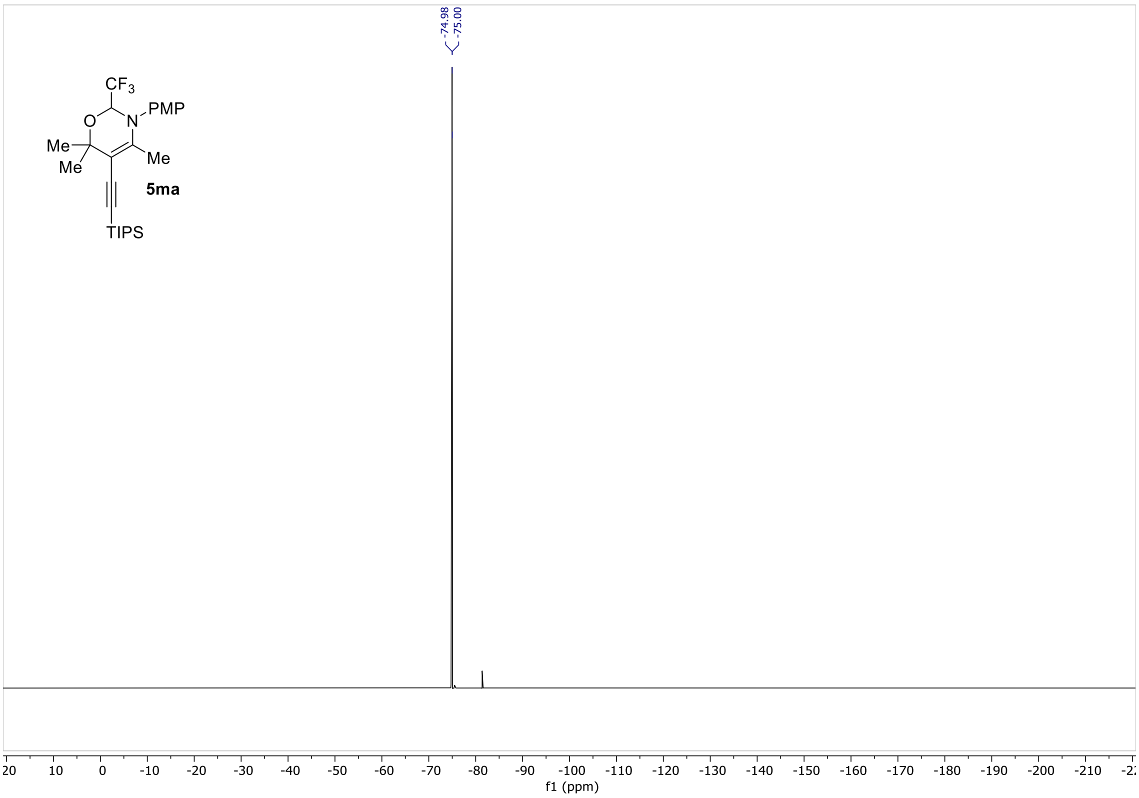
-74.98
-75.00
CF3
O
N
PMP
Me
Me
Me
5ma
TIPS
20 10 0 -10 -20 -30 -40 -50 -60 -70 -80 -90 -100 -110 -120 -130 -140 -150 -160 -170 -180 -190 -200 -210
f1 (ppm)

3-(4-Methoxyphenyl)-4-methyl-2-(trifluoromethyl)-5-(phenylethynyl)-3,6-dihydro-2*H*-1,3-oxazine (5ab)

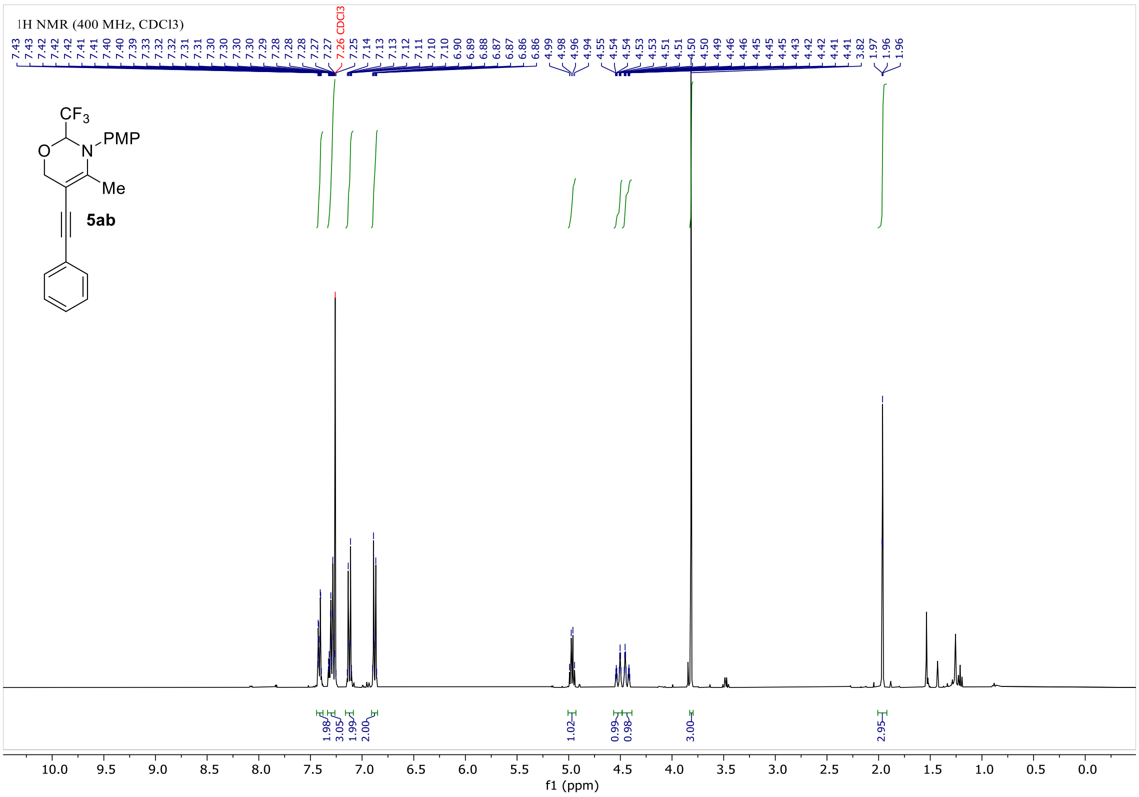

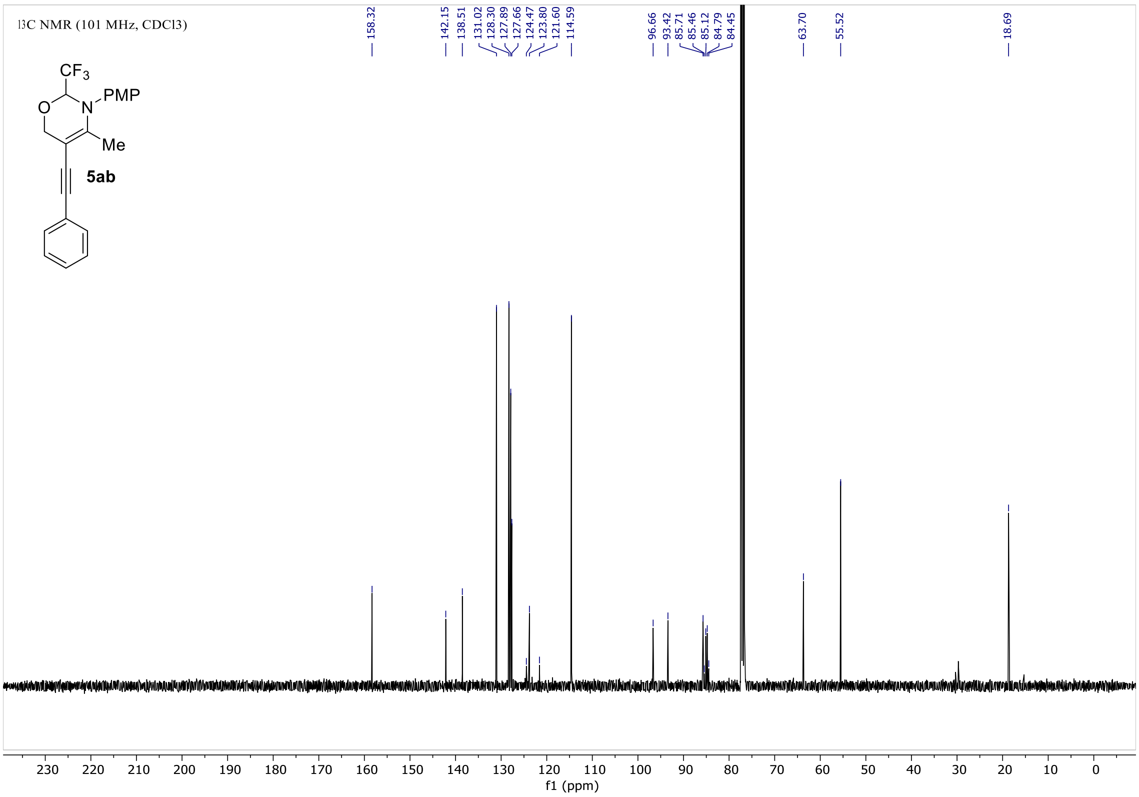

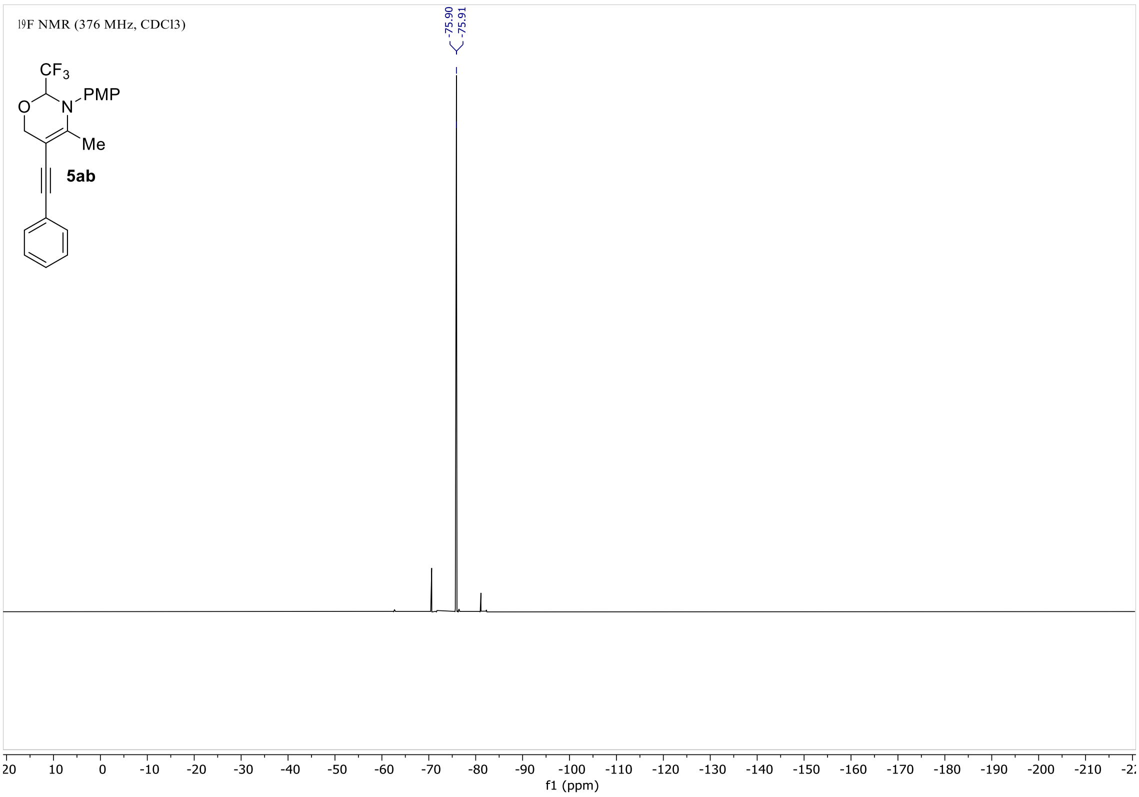

3-(4-Methoxyphenyl)-4-methyl-2-(trifluoromethyl)-5-((4-(trifluoromethyl)phenyl)ethynyl)-3,6-dihydro-2*H*-1,3-oxazine (5ac)

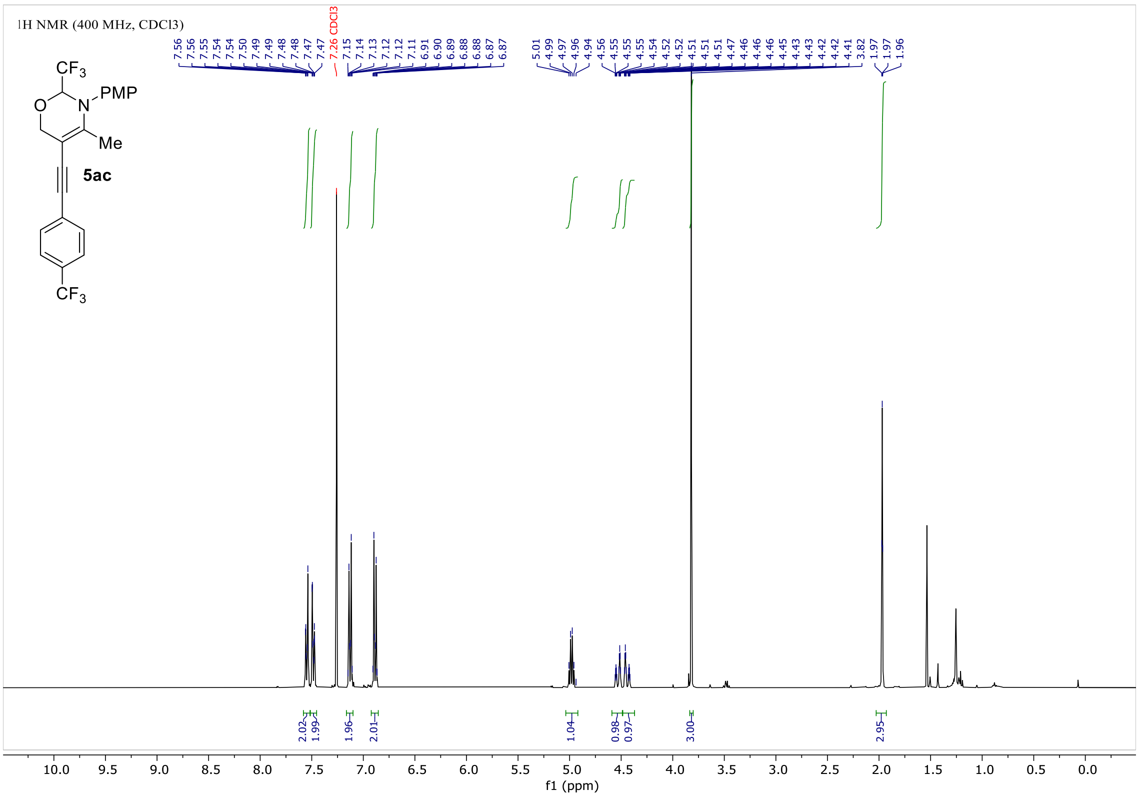

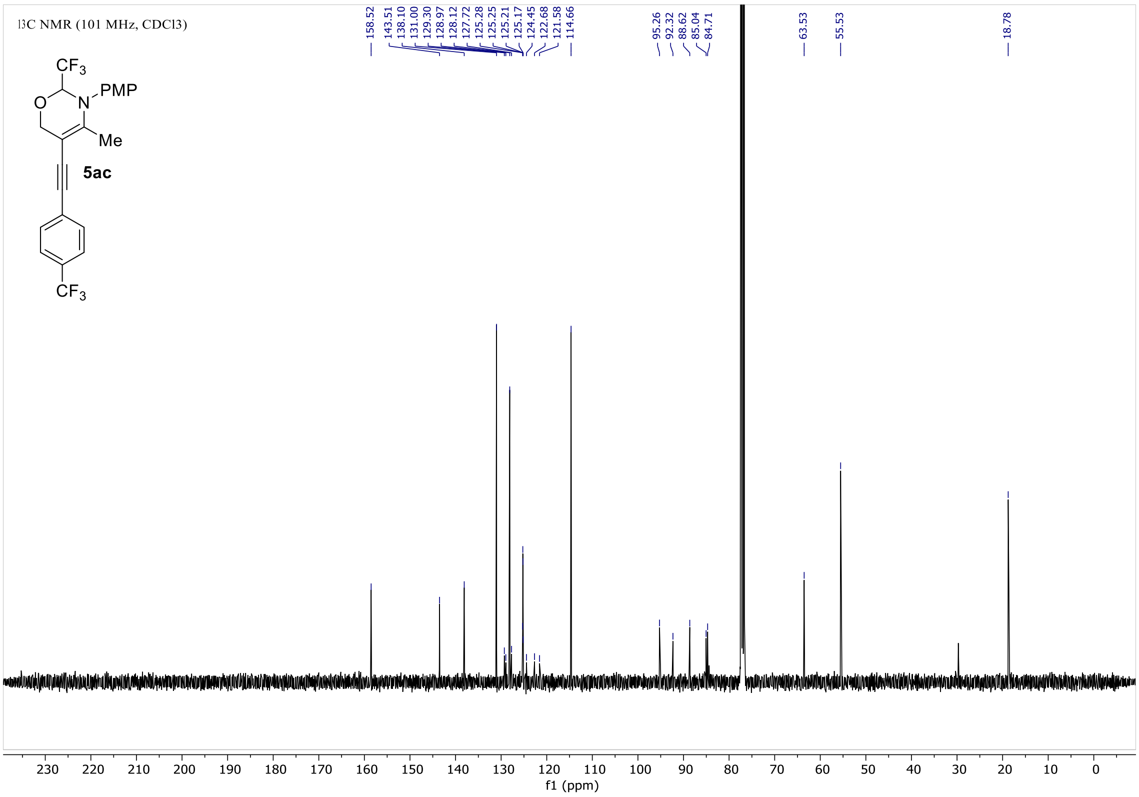
13C NMR (101 MHz, CDCl3)
158.52
143.51
138.10
131.00
129.30
128.97
128.12
127.72
125.28
125.25
125.21
125.17
124.45
122.68
121.58
114.66
95.26
92.32
88.62
85.04
84.71
63.53
55.53
18.78
CF3
PMP
N
O
Me
5ac
CF3
f1 (ppm)

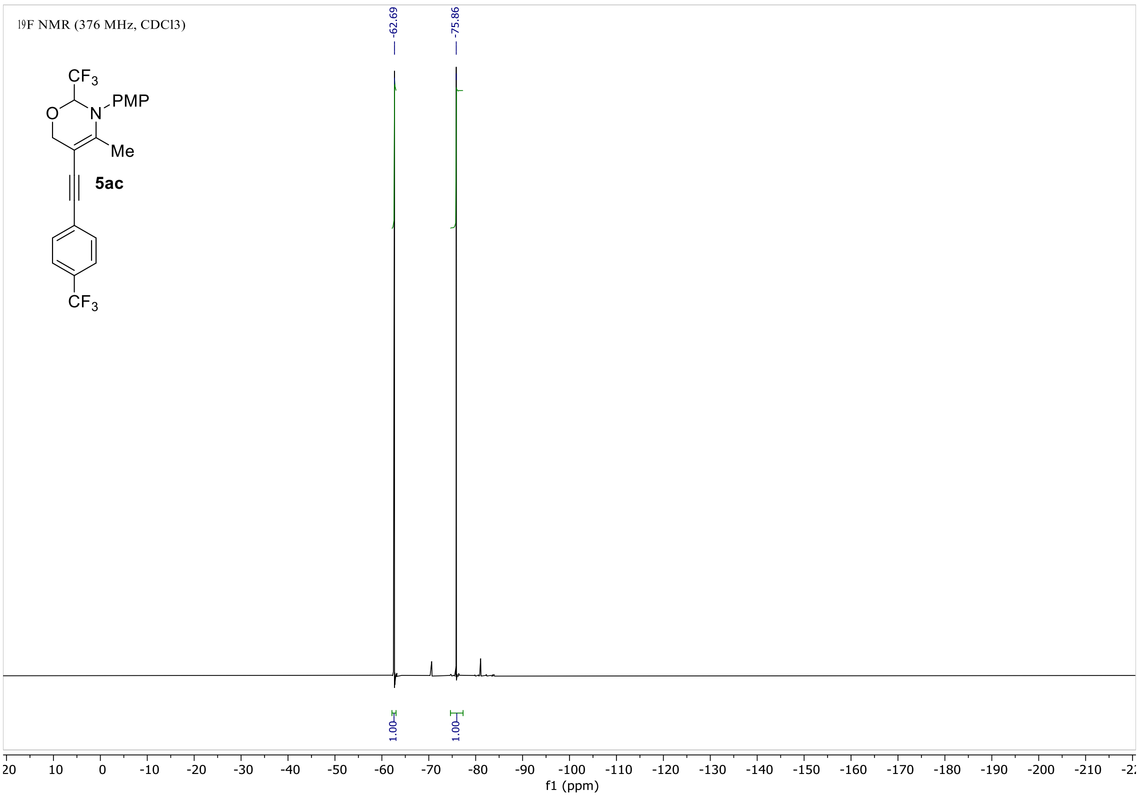
19F NMR (376 MHz, CDCl3)
-62.69
-75.86
CF3
PMP
N
O
Me
5ac
CF3
1.00
1.00
f1 (ppm)

1-(4-((3-(4-Methoxyphenyl)-4-methyl-2-(trifluoromethyl)-3,6-dihydro-2*H*-1,3-oxazin-5-yl)ethynyl)phenyl)ethan-1-one (5ad)

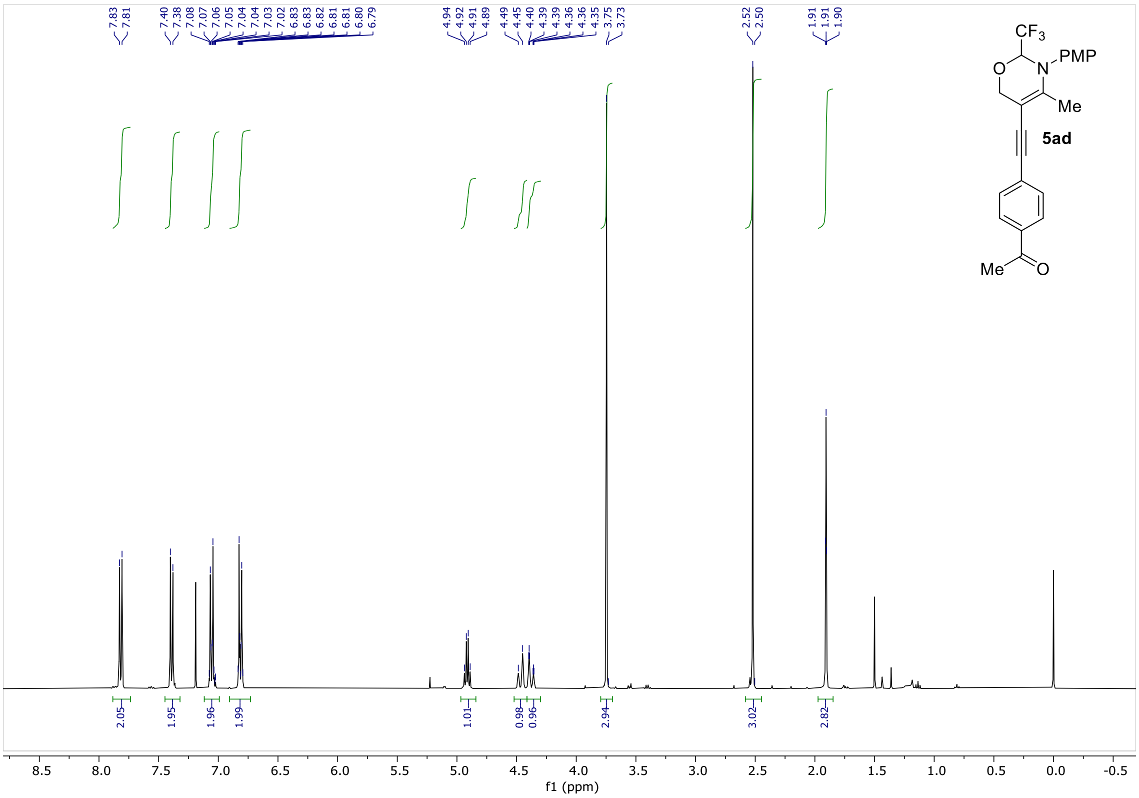

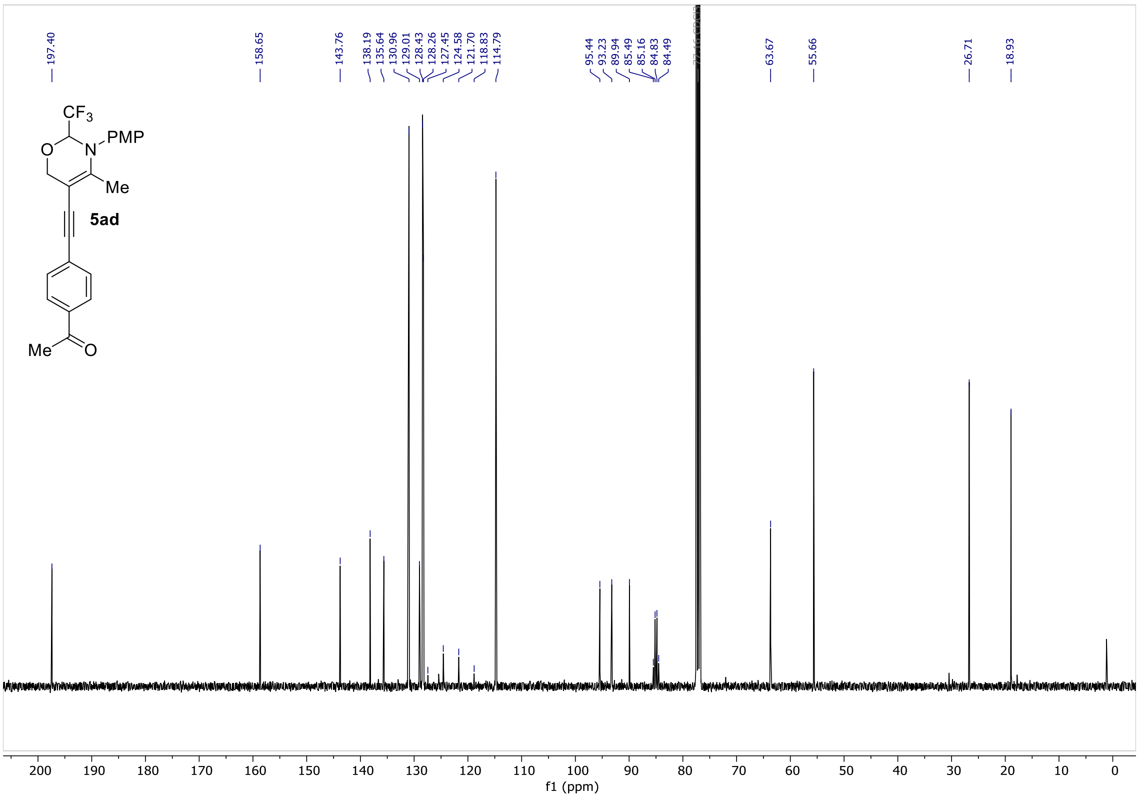

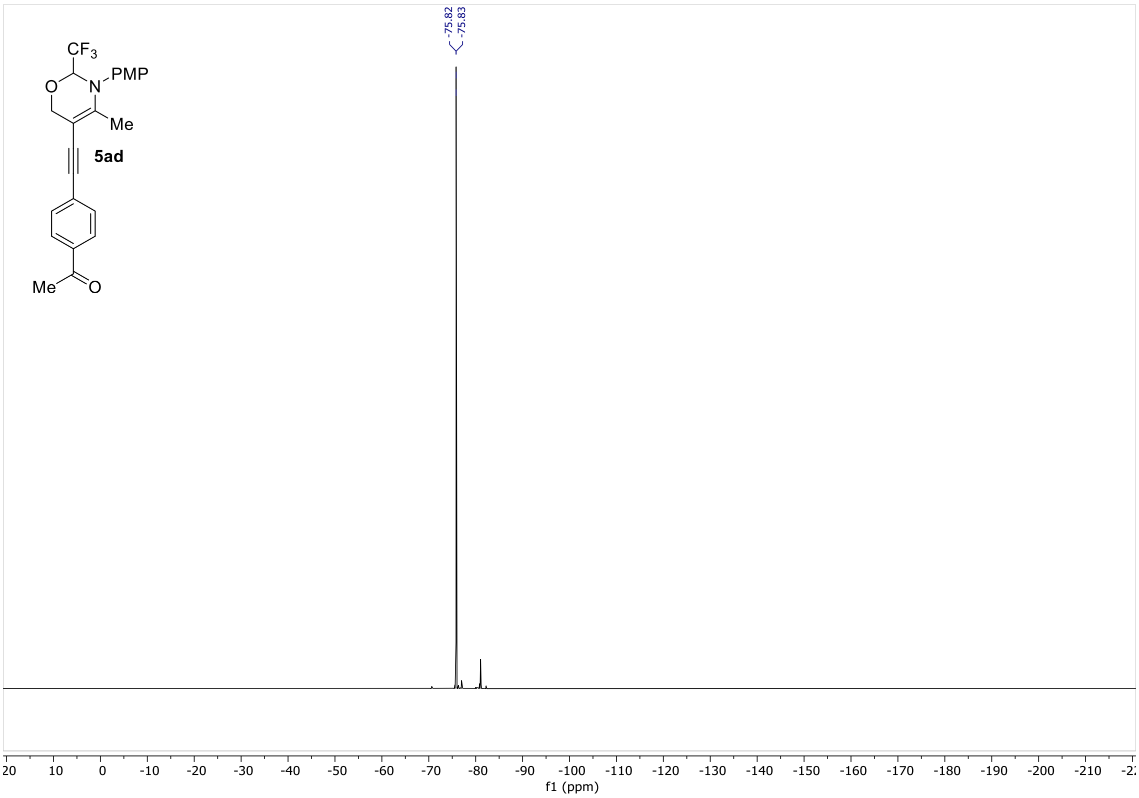

3-(4-Methoxyphenyl)-4-methyl-2-(trifluoromethyl)-5-(3,3,3-trifluoroprop-1-en-2-yl)-3,6-dihydro-2*H*-1,3-oxazine (5ae)

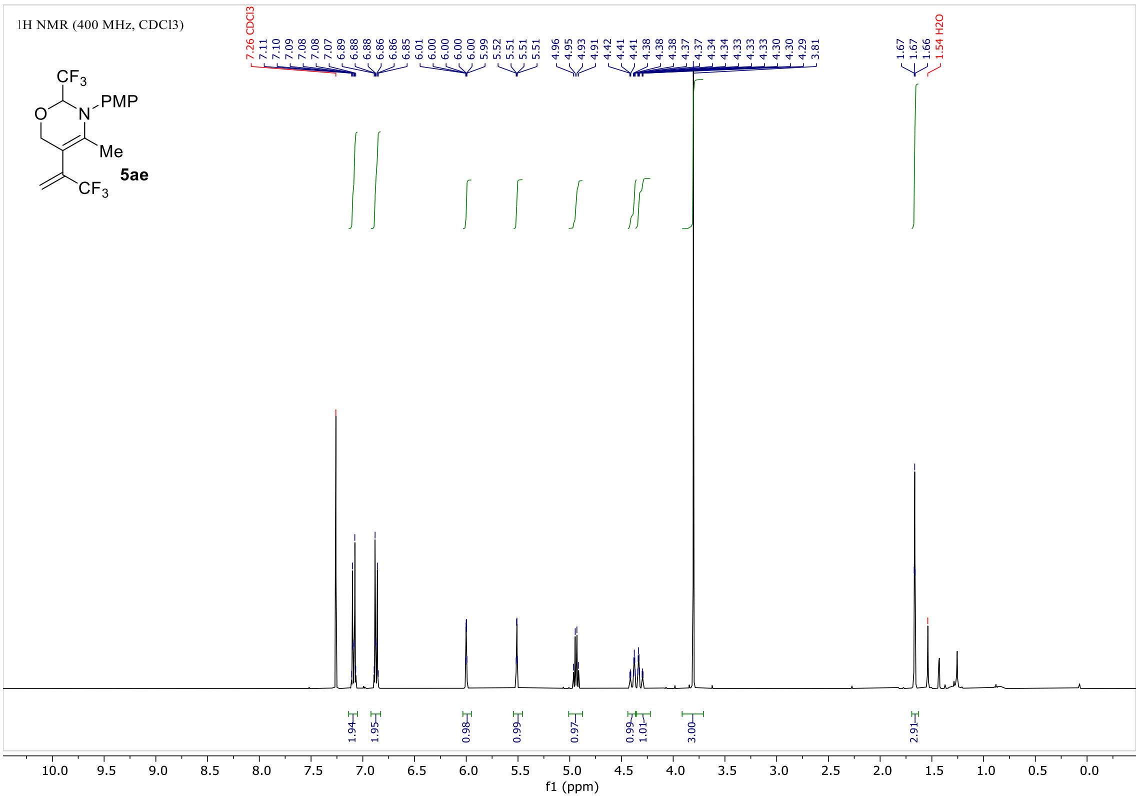

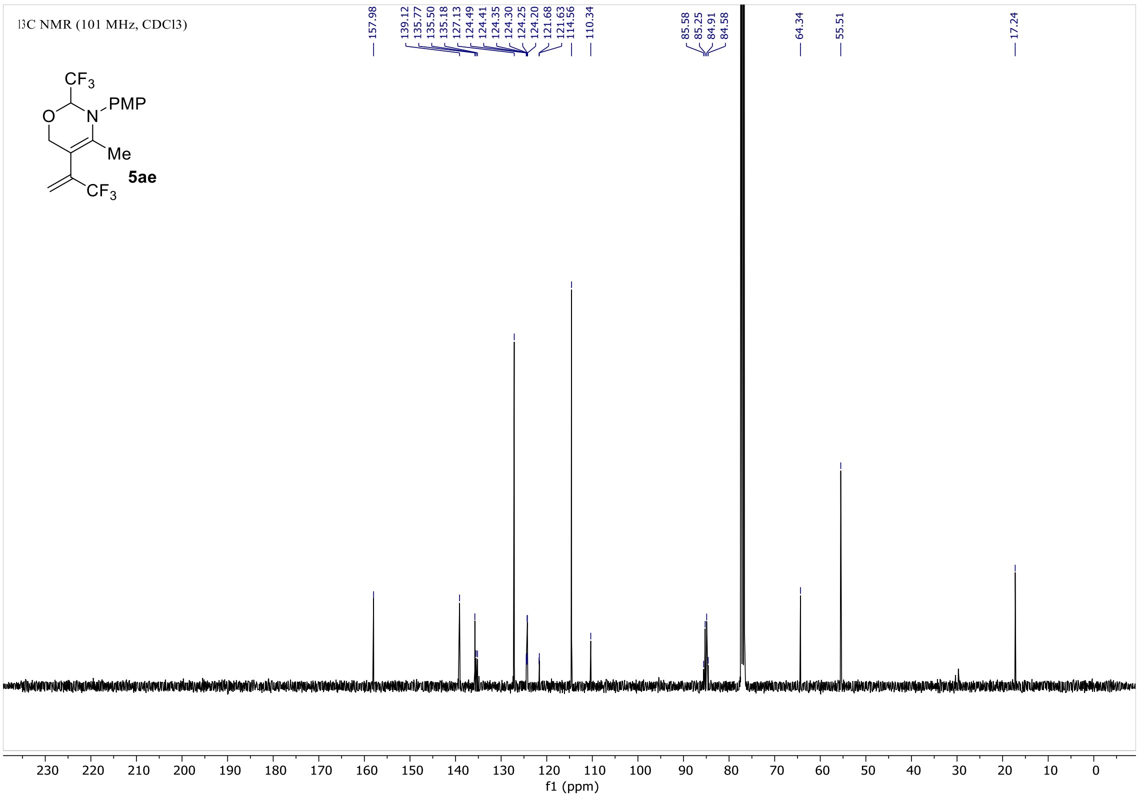
13C NMR (101 MHz, CDCl3)
157.98
139.12
135.77
135.50
135.18
127.13
124.49
124.41
124.35
124.30
124.25
124.20
121.68
121.63
114.56
110.34
85.58
85.25
84.91
84.58
64.34
55.51
17.24
CF3
O
N
PMP
Me
5ae
CF3
230 220 210 200 190 180 170 160 150 140 130 120 110 100 90 80 70 60 50 40 30 20 10 0
f1 (ppm)

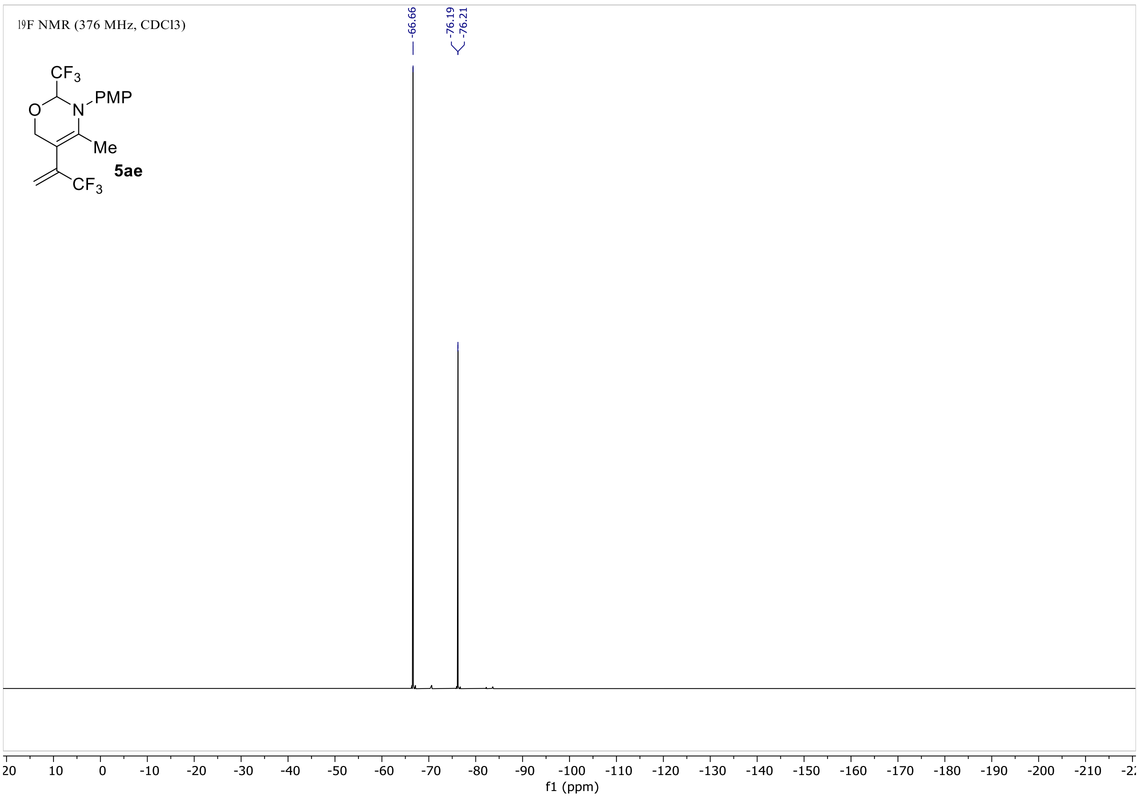
19F NMR (376 MHz, CDCl3)
-66.66
-76.19
-76.21
CF3
O
N
PMP
Me
5ae
CF3
20 10 0 -10 -20 -30 -40 -50 -60 -70 -80 -90 -100 -110 -120 -130 -140 -150 -160 -170 -180 -190 -200 -210
f1 (ppm)

3-(4-Methoxyphenyl)-4-methyl-5-(p-tolyl)-2-(trifluoromethyl)-3,6-dihydro-2*H*-1,3-oxazine (5af)

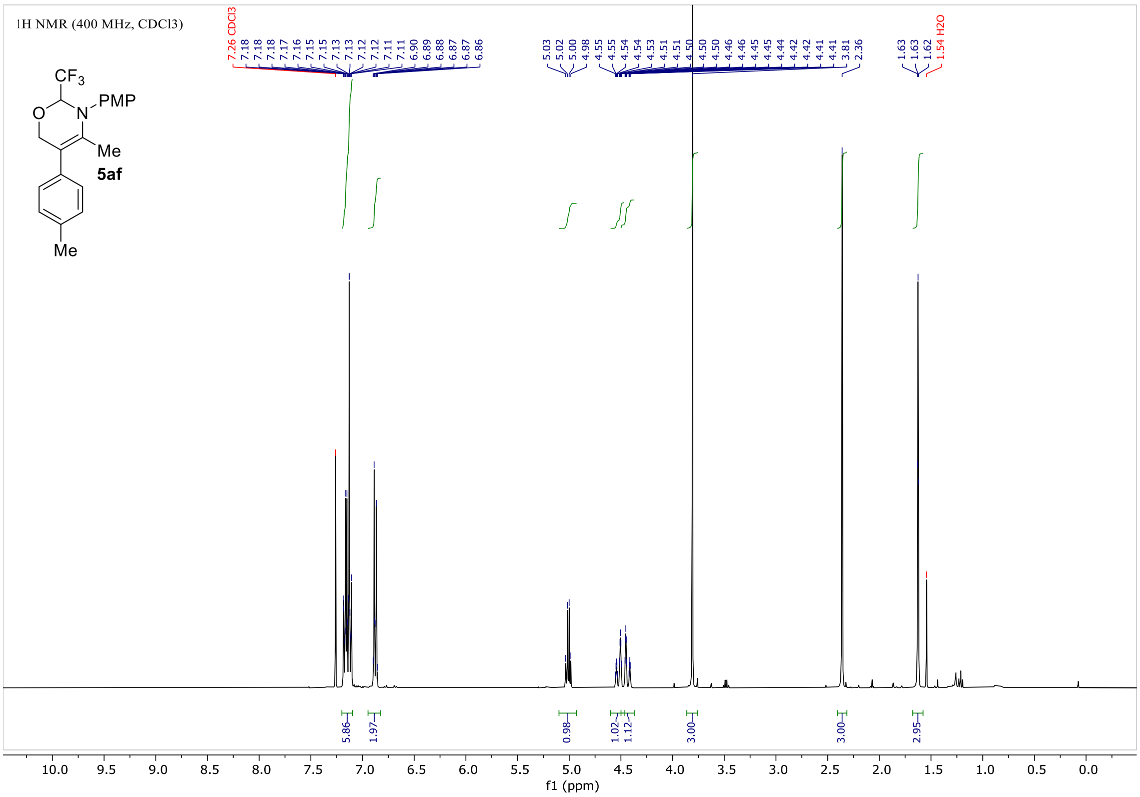

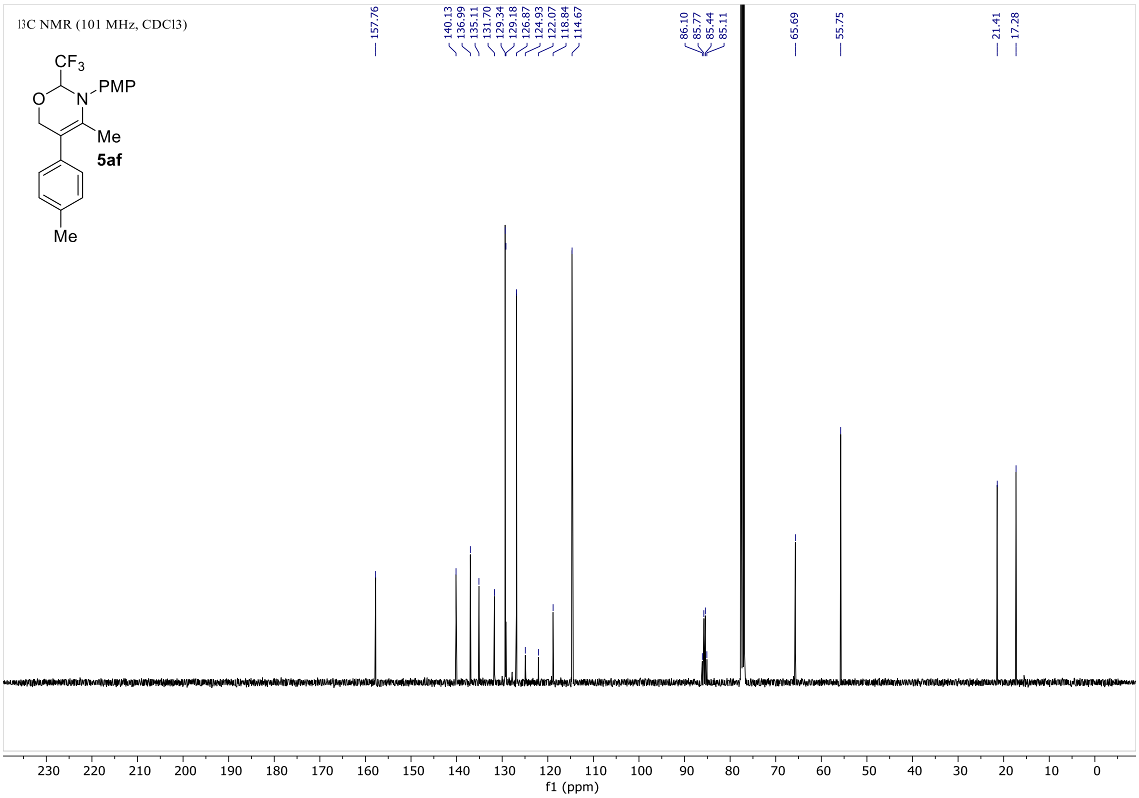

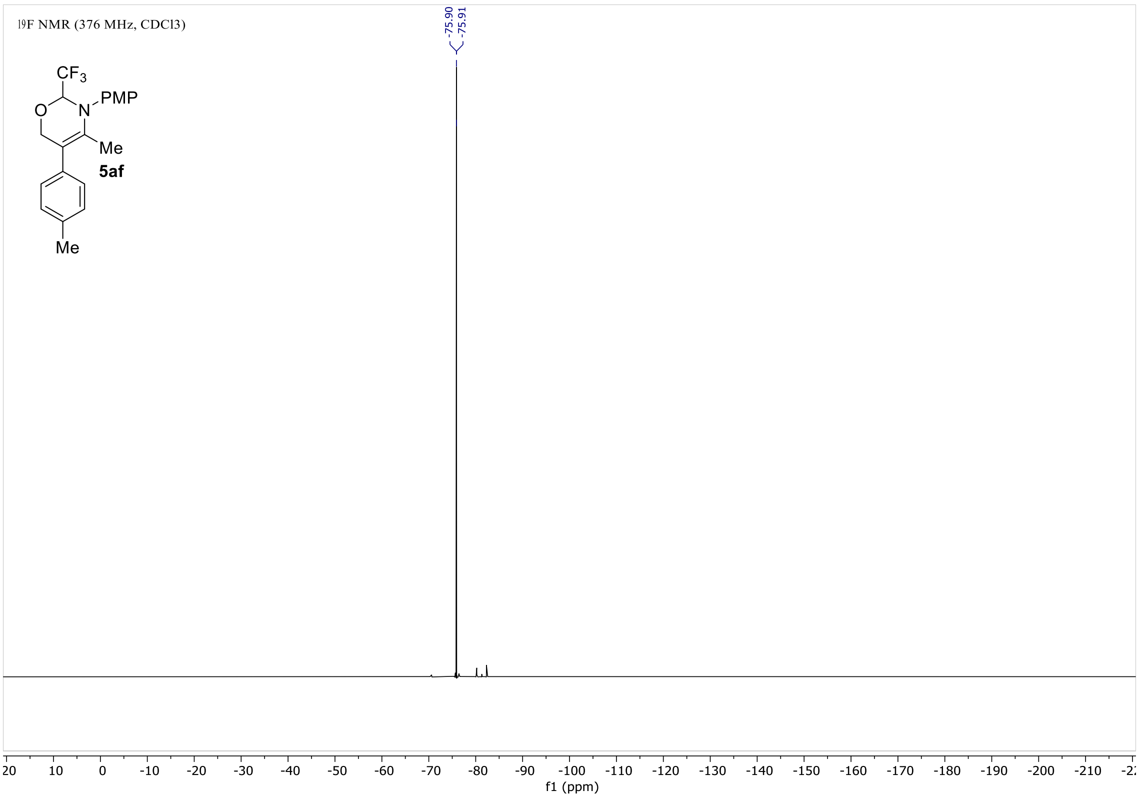

3-(4-Methoxyphenyl)-4-methyl-2-(trifluoromethyl)-5-(4-methoxyphenyl)-3,6-dihydro-2*H*-1,3-oxazine (5ag)

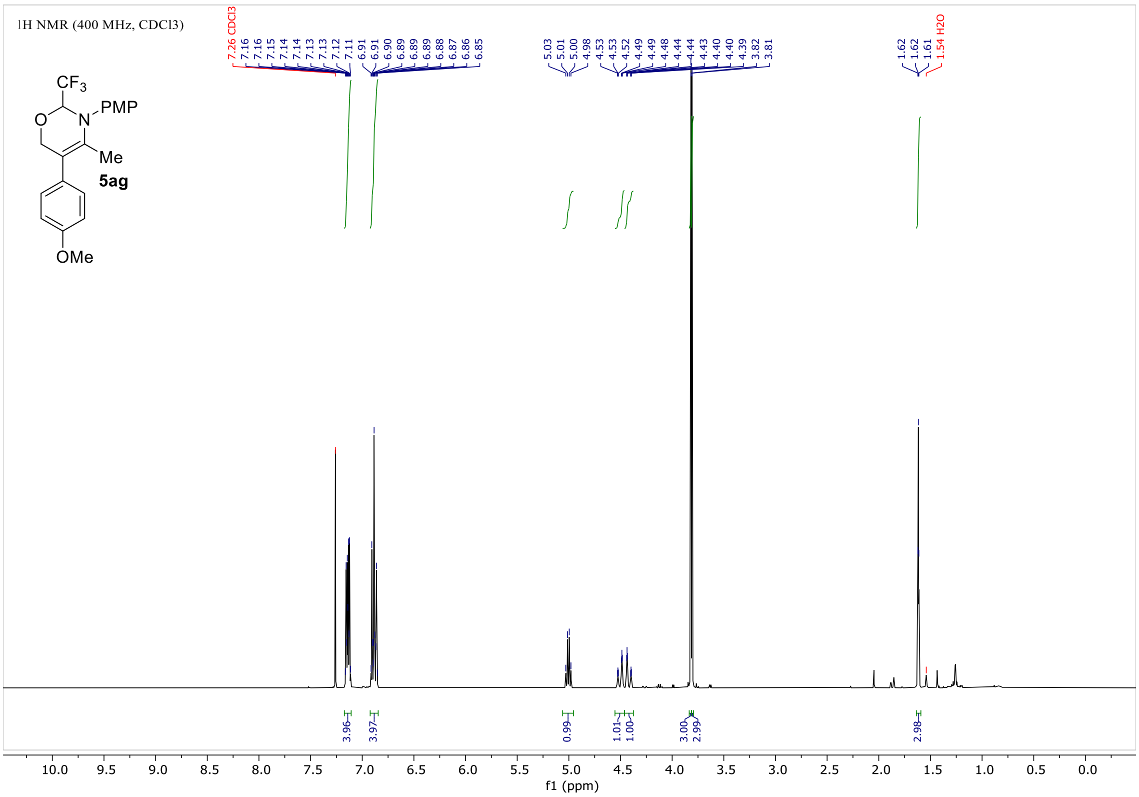

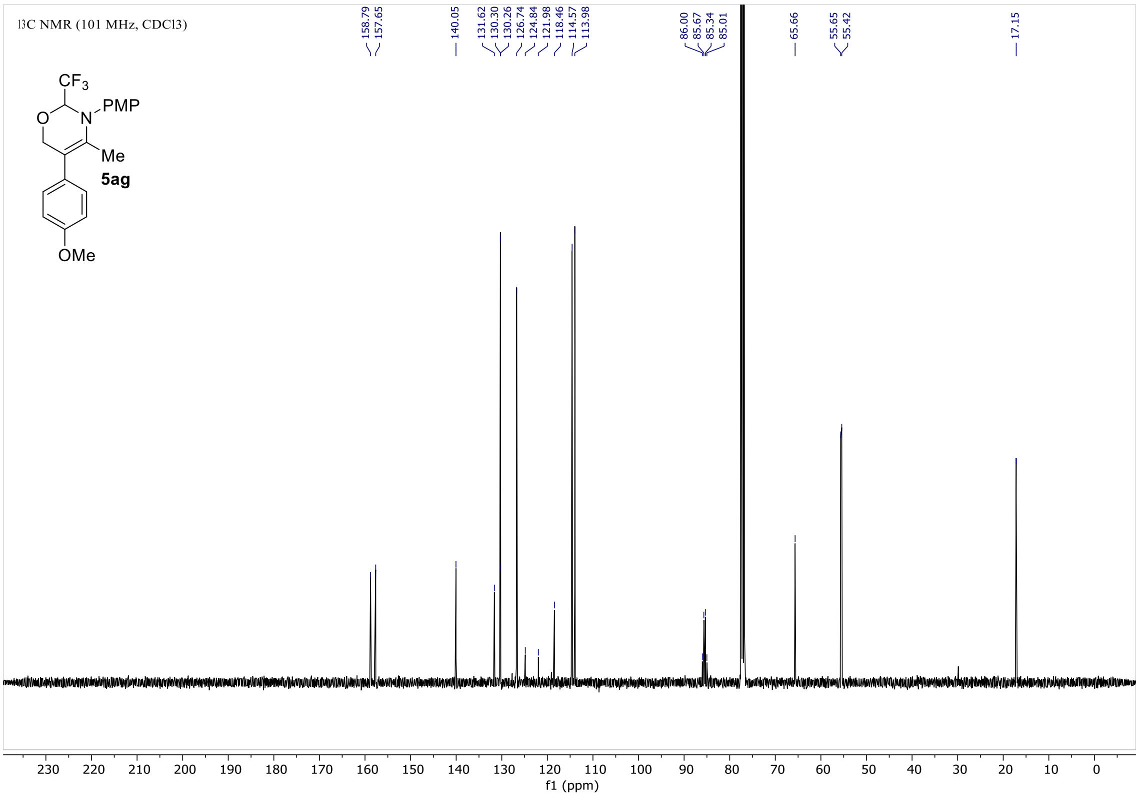
13C NMR (101 MHz, CDCl3)
158.79
157.65
140.05
131.62
130.30
130.26
126.74
124.84
121.98
118.46
114.57
113.98
86.00
85.67
85.34
85.01
65.66
55.65
55.42
17.15
CF3
O
N
PMP
Me
5ag
OMe
f1 (ppm)

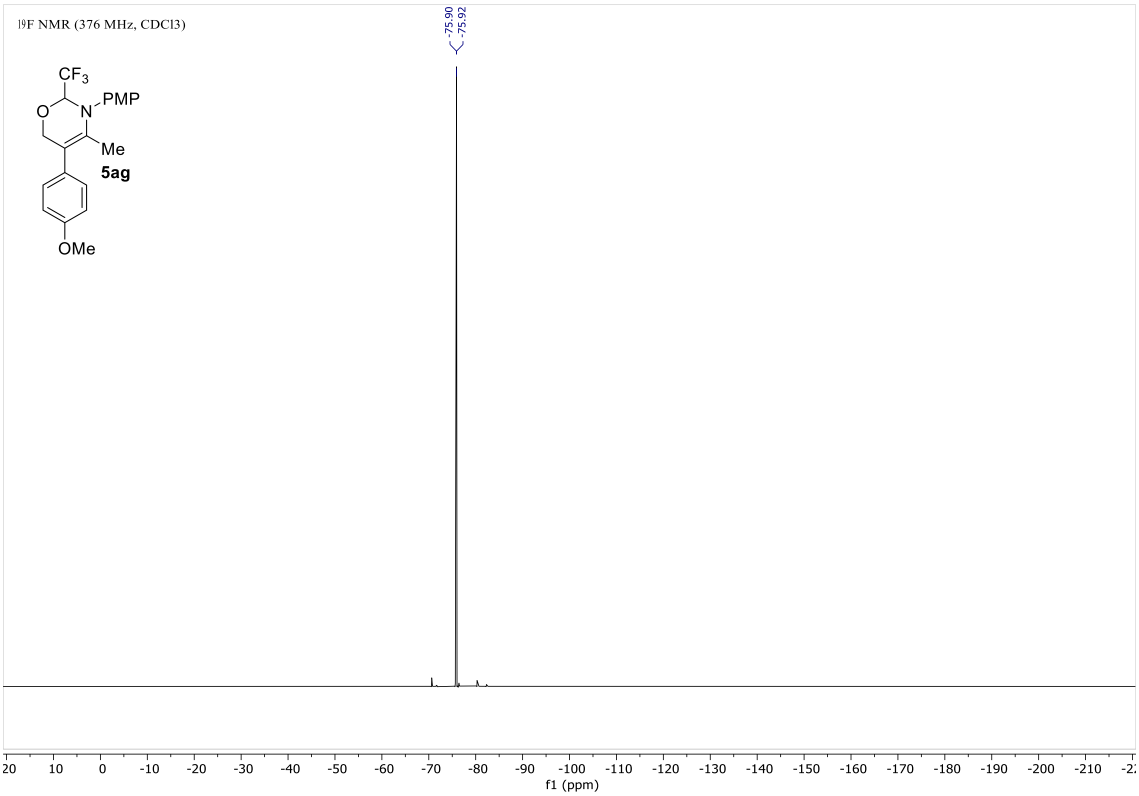
19F NMR (376 MHz, CDCl3)
-75.90
-75.92
CF3
O
N
PMP
Me
5ag
OMe
f1 (ppm)

3-(4-Methoxyphenyl)-4-methyl-2-(trifluoromethyl)-5-(4-trifluoromethylphenyl)-3,6-dihydro-2*H*-1,3-oxazine (5ah)

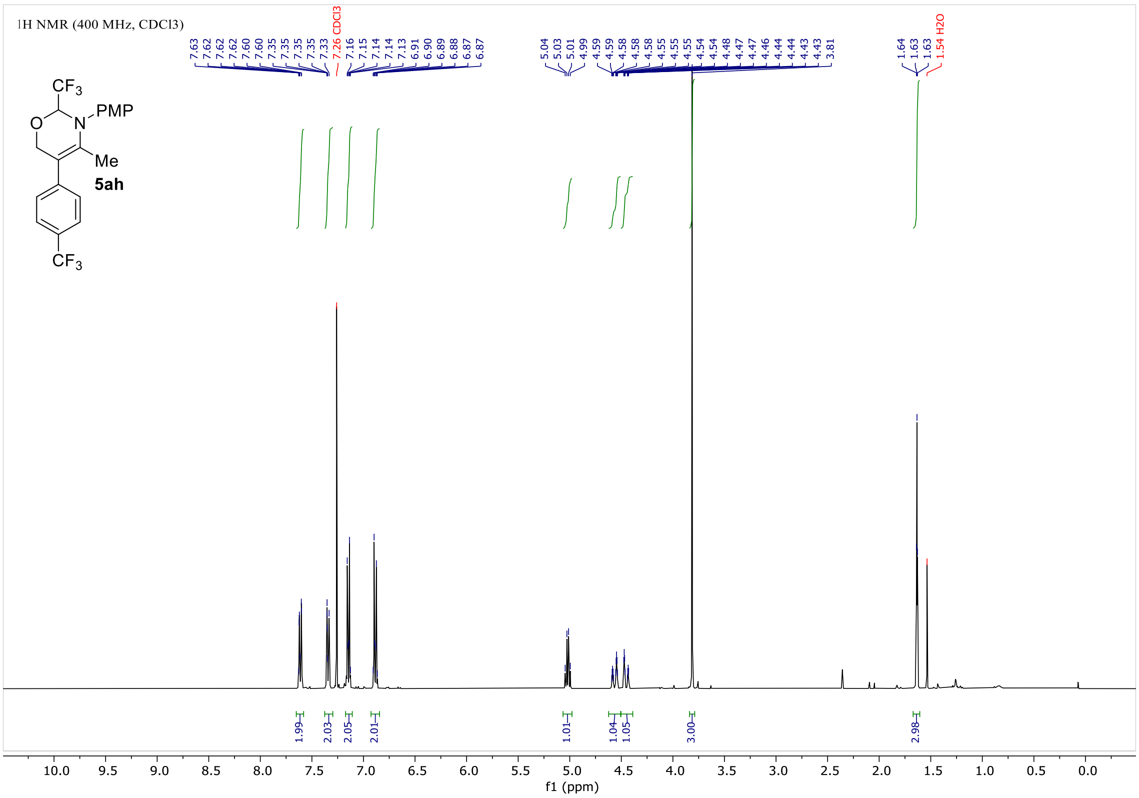

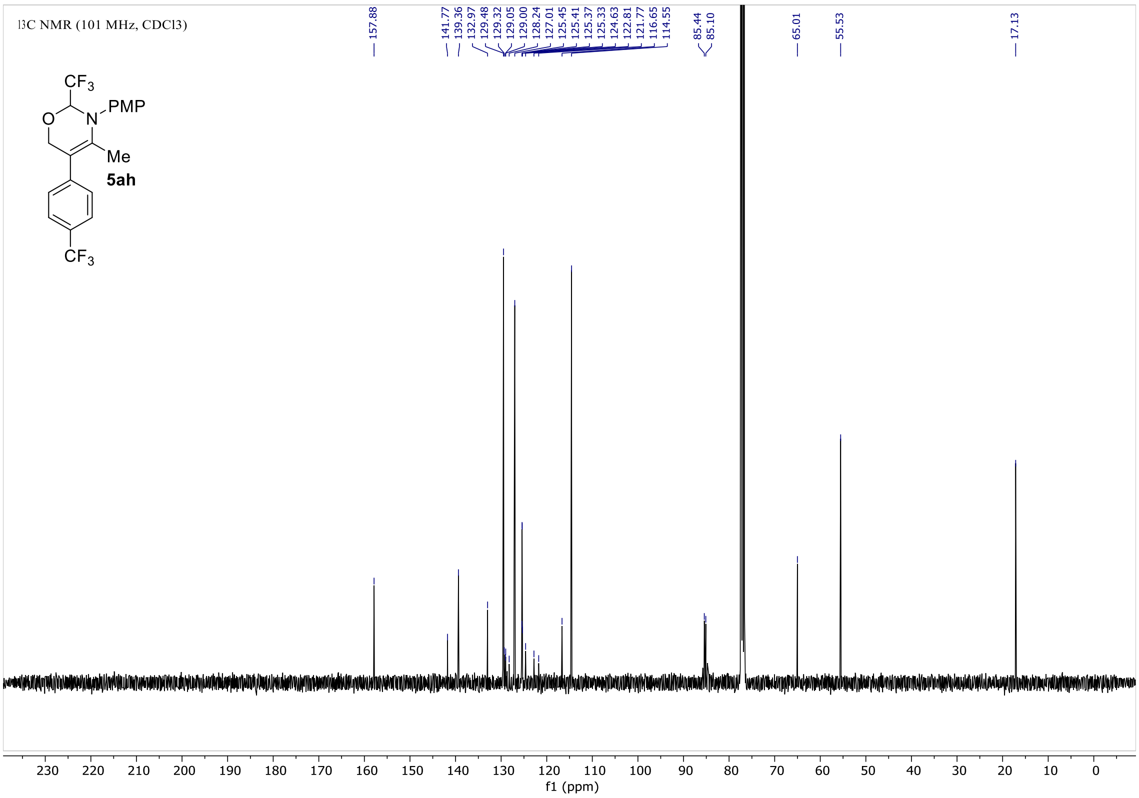

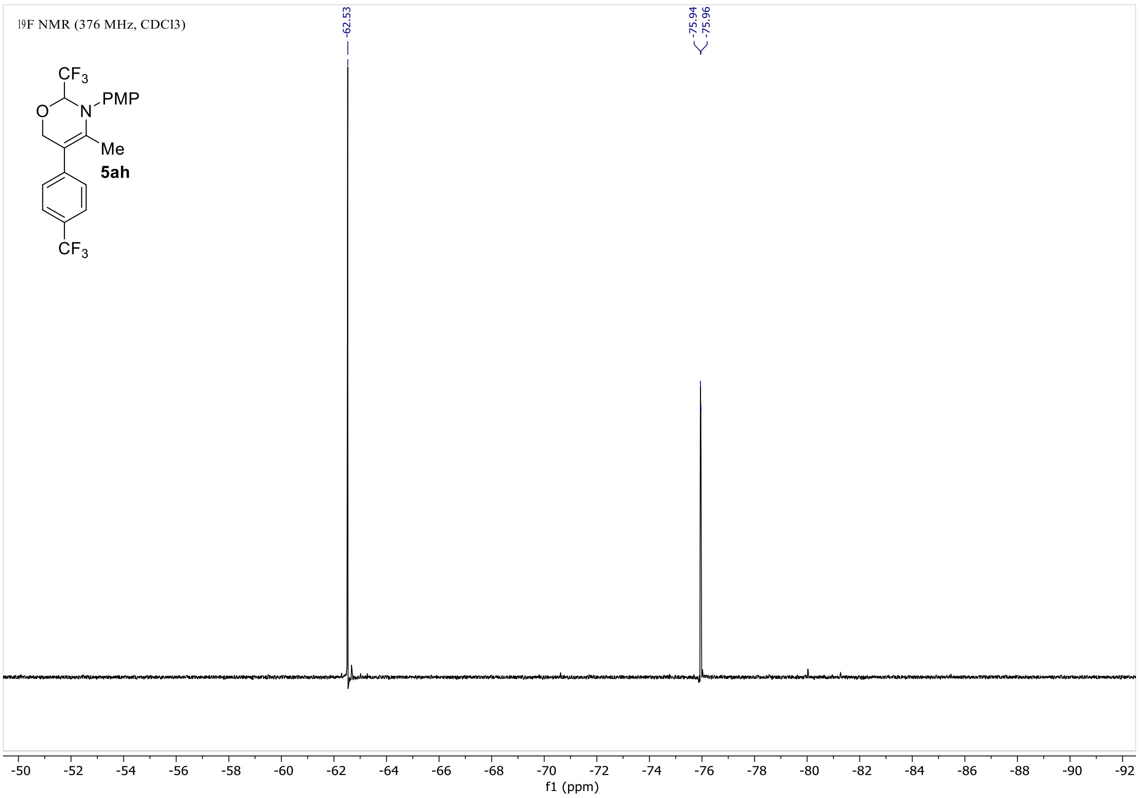

5-(4-Chlorophenyl)-3-(4-methoxyphenyl)-4-methyl-2-(trifluoromethyl)-3,6-dihydro-2*H*-1,3-oxazine (5ai)

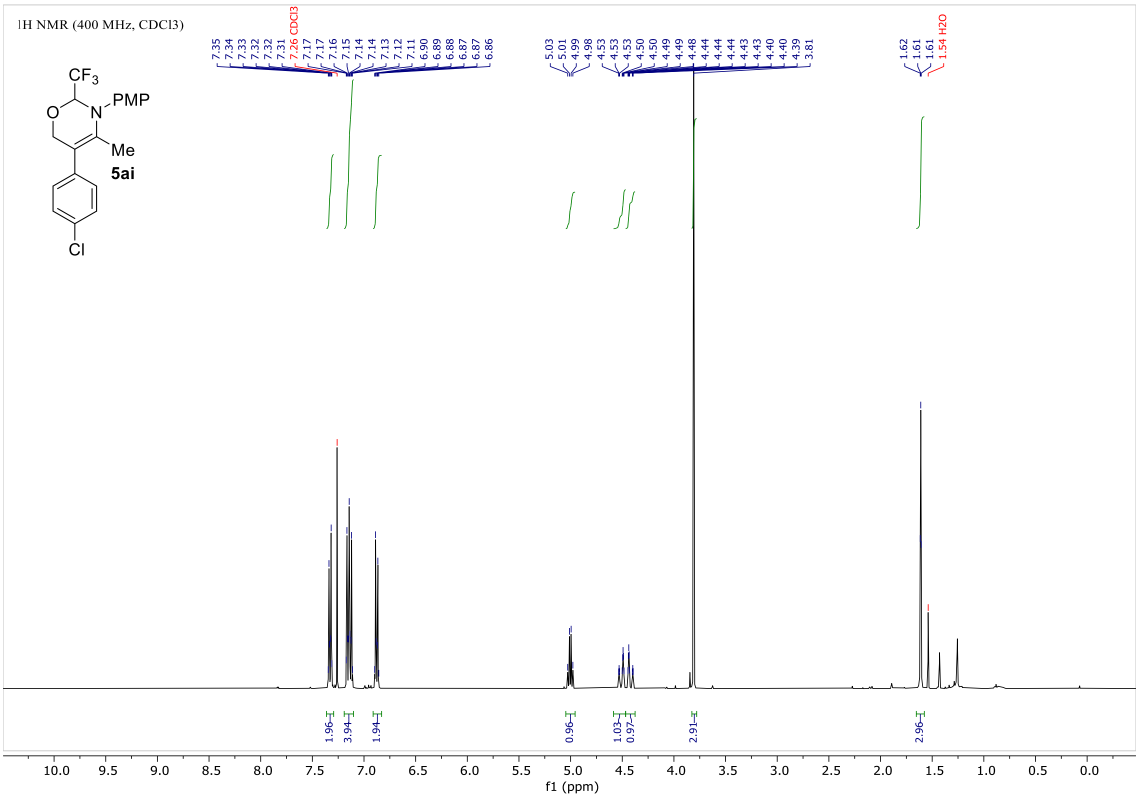

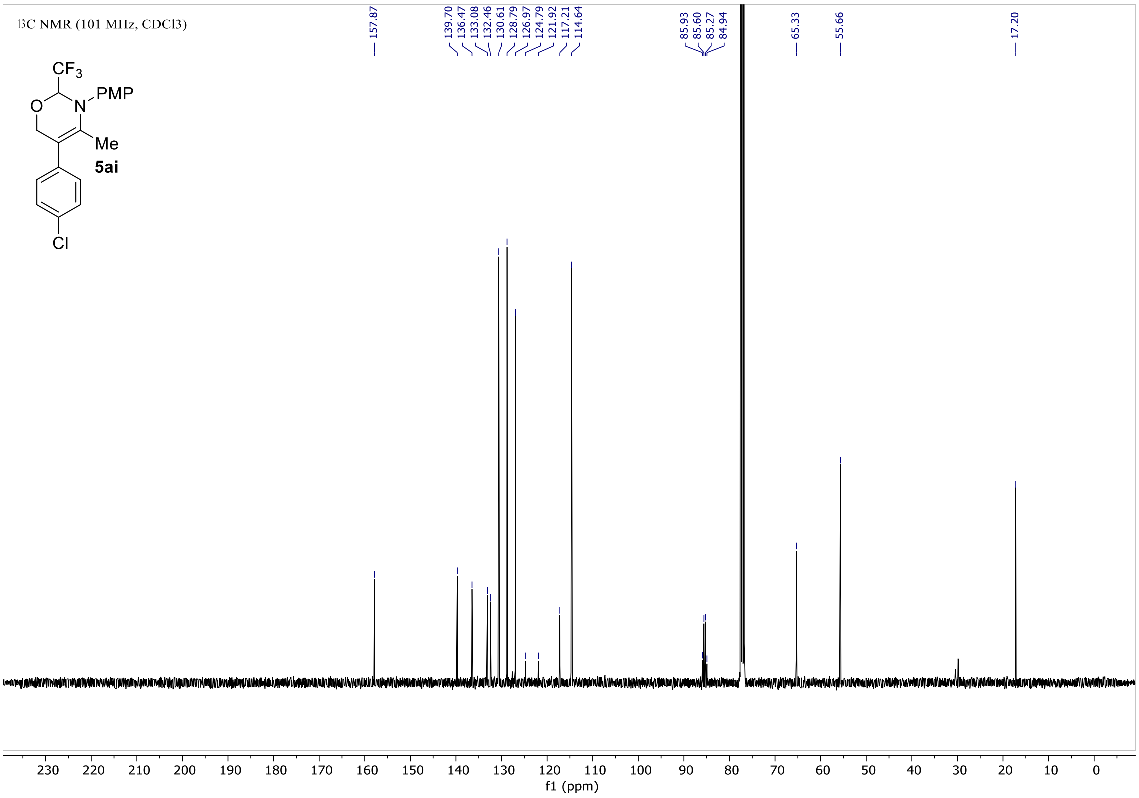
13C NMR (101 MHz, CDCl3)
157.87
139.70
136.47
133.08
132.46
130.61
128.79
126.97
124.79
121.92
117.21
114.64
85.93
85.60
85.27
84.94
65.33
55.66
17.20
CF3
O
N
PMP
Me
5ai
Cl
f1 (ppm)

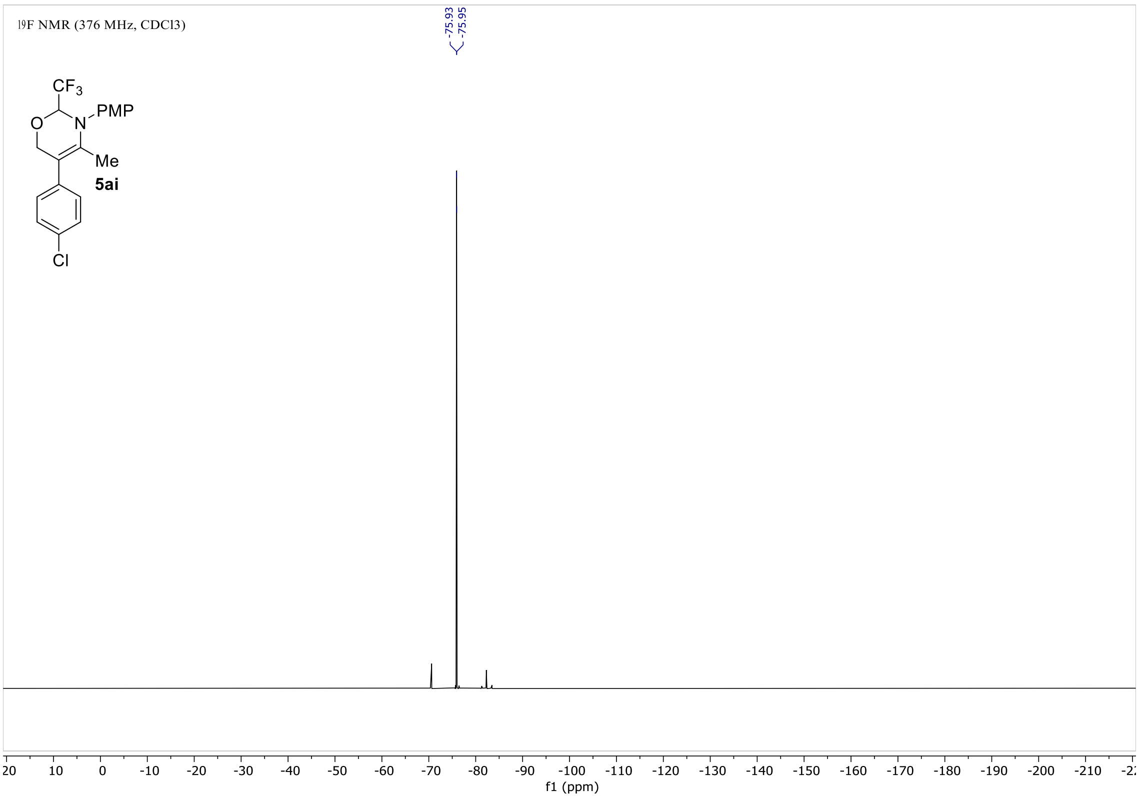
19F NMR (376 MHz, CDCl3)
-75.93
-75.95
CF3
O
N
PMP
Me
5ai
Cl
f1 (ppm)

5-(4-Fluorophenyl)-3-(4-methoxyphenyl)-4-methyl-2-(trifluoromethyl)-3,6-dihydro-2*H*-1,3-oxazine (5aj)

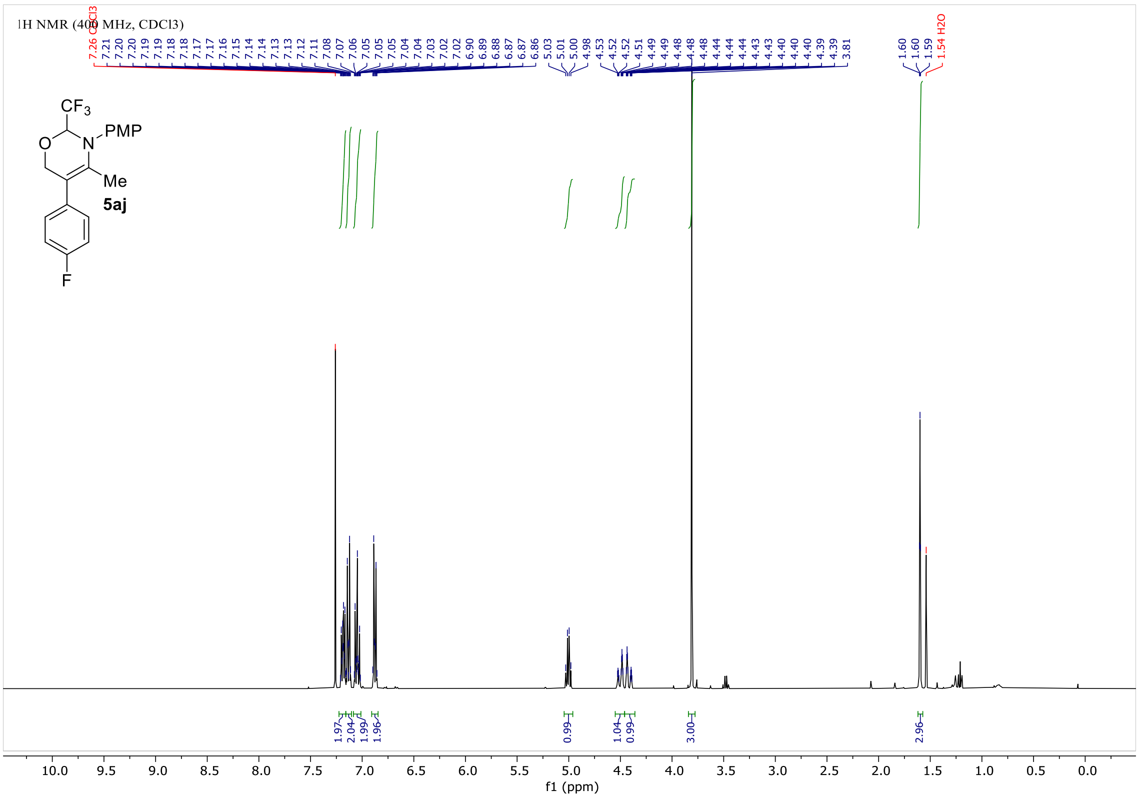

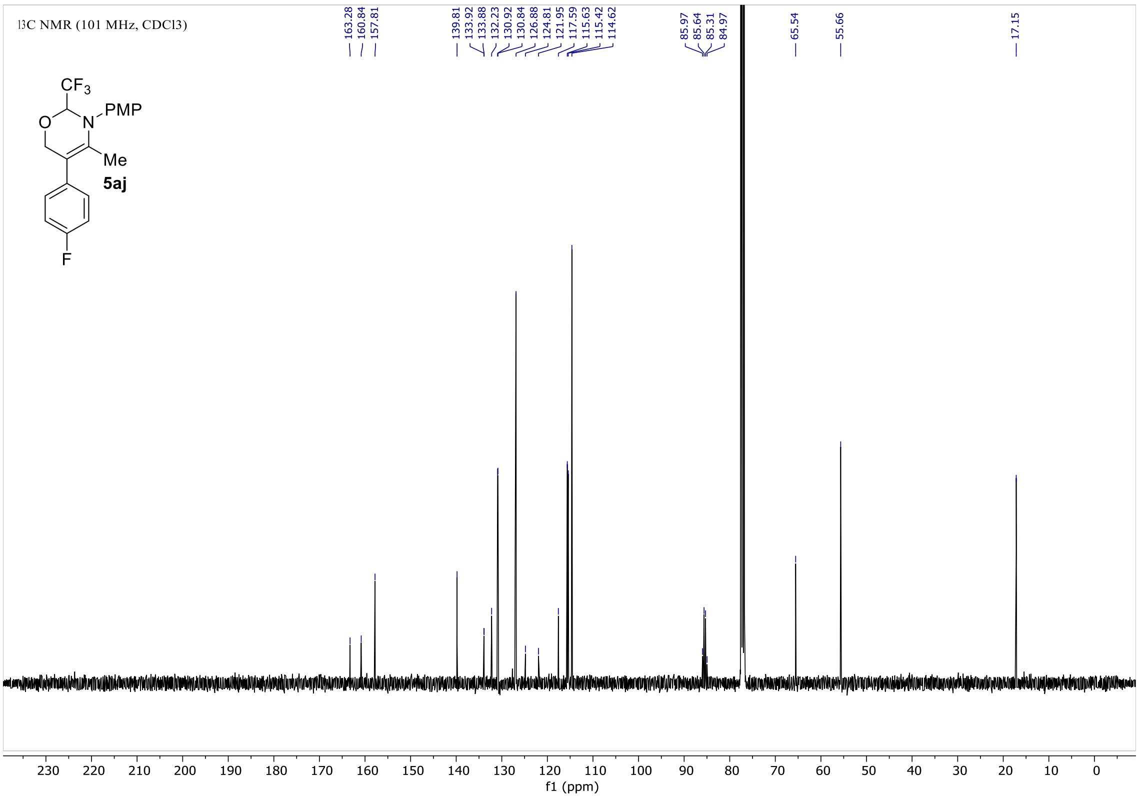

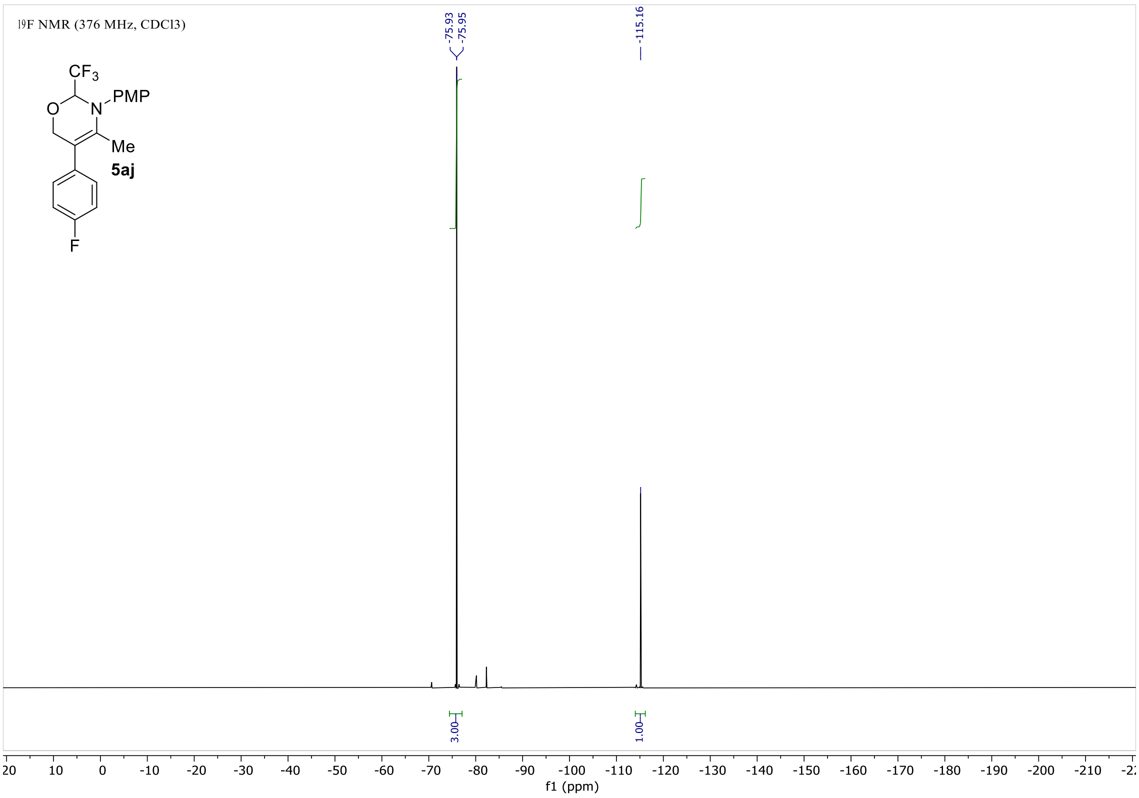

5-(3-Fluorophenyl)-3-(4-methoxyphenyl)-4-methyl-2-(trifluoromethyl)-3,6-dihydro-2*H*-1,3-oxazine (5ak)

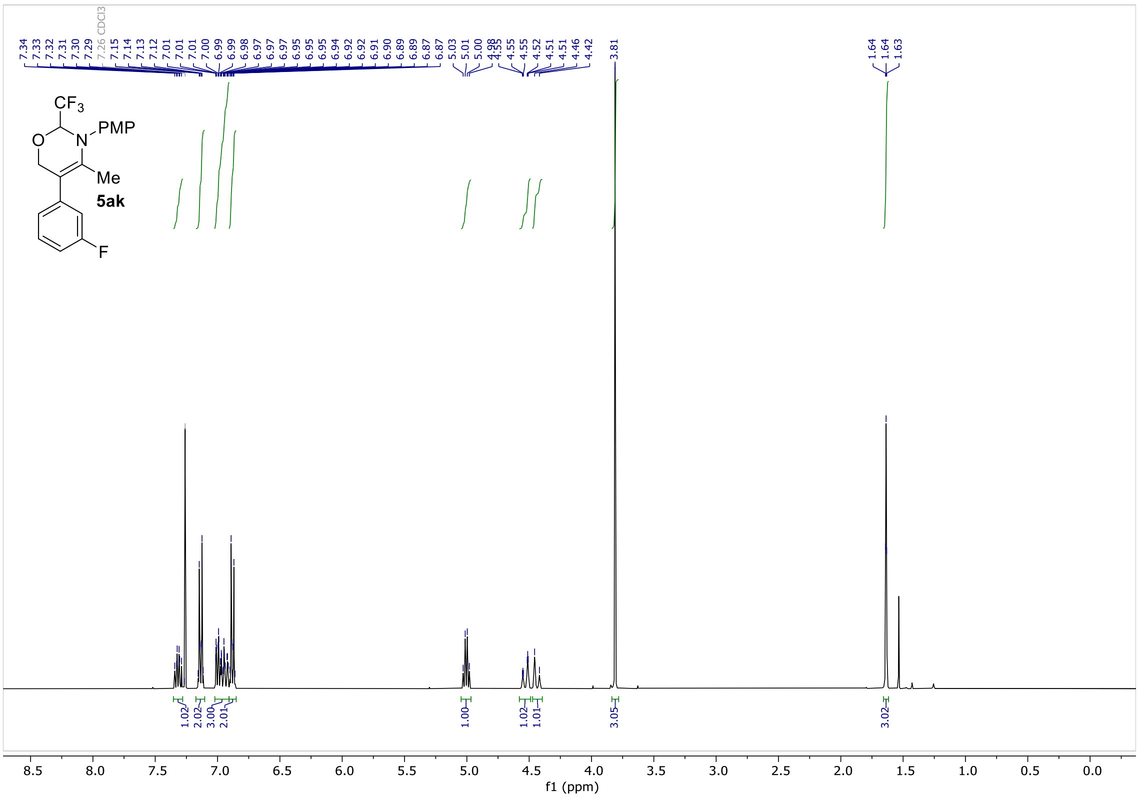

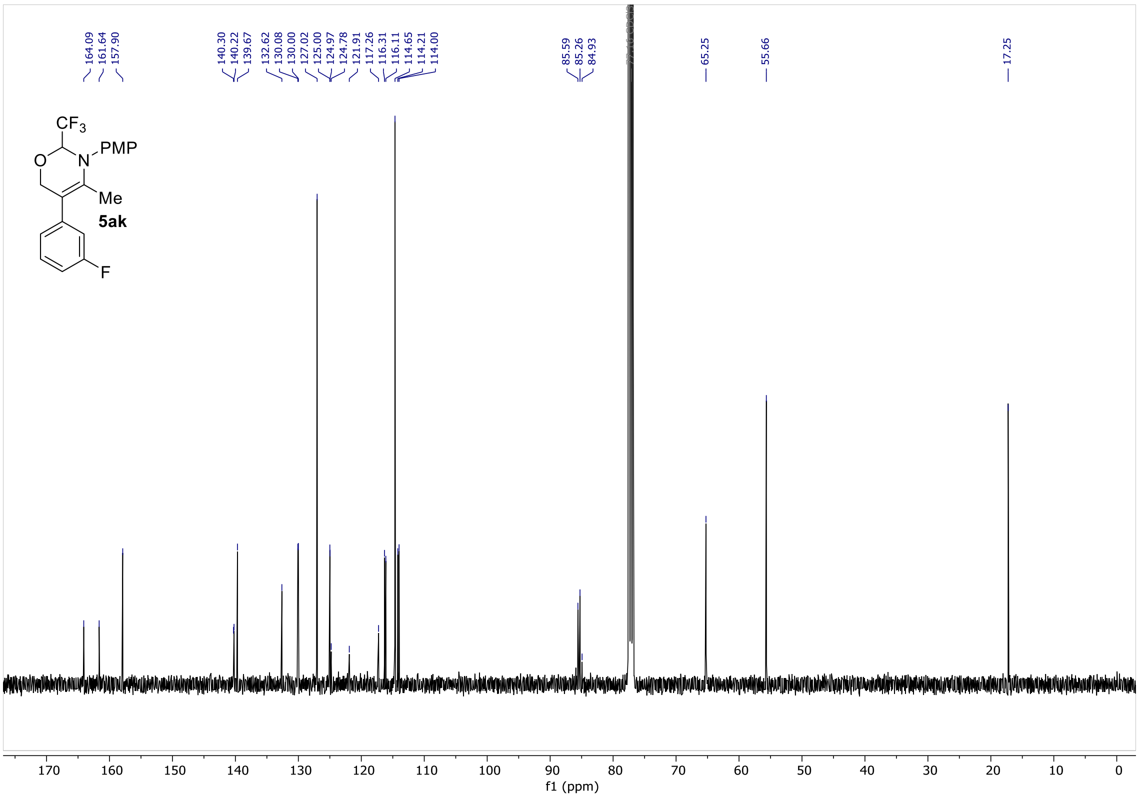
164.09
161.64
157.90
140.30
140.22
139.67
132.62
130.08
130.00
127.02
125.00
124.97
124.78
121.91
117.26
116.31
116.11
114.65
114.21
114.00
85.59
85.26
84.93
65.25
55.66
17.25
CF3
PMP
O
N
Me
5ak
F
f1 (ppm)

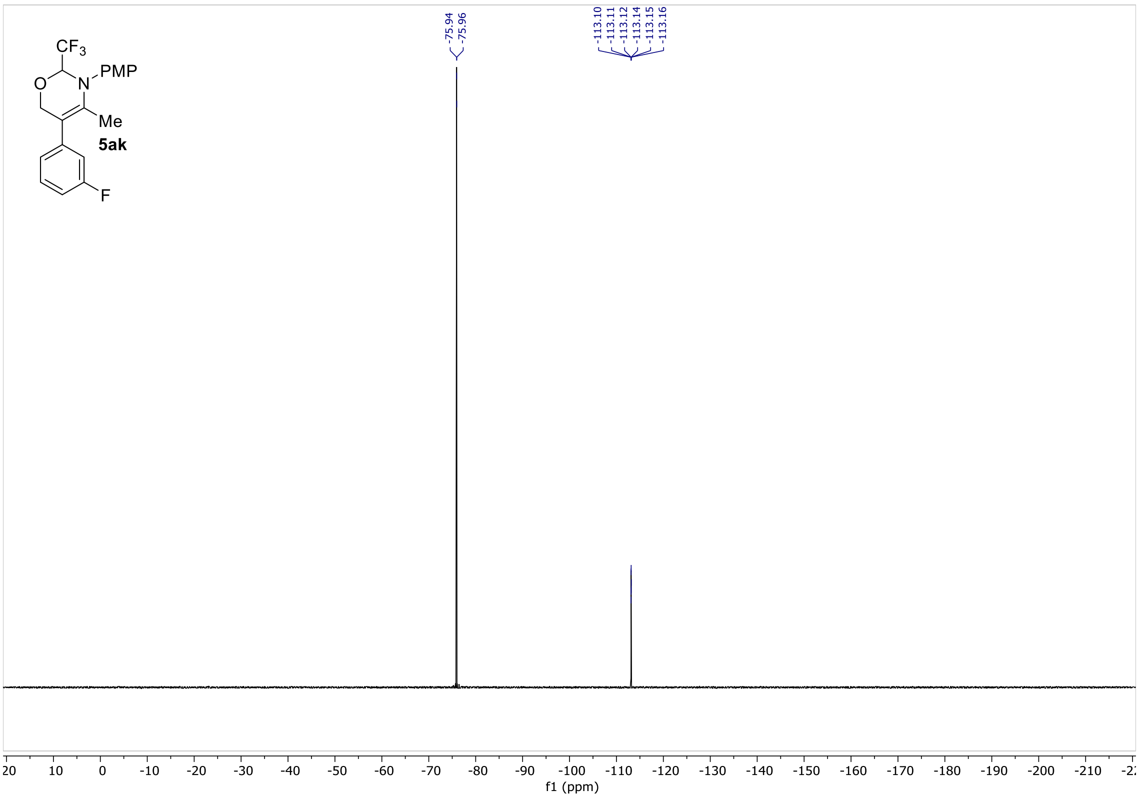
-75.94
-75.96
-113.10
-113.11
-113.12
-113.14
-113.15
-113.16
CF3
PMP
O
N
Me
5ak
F
f1 (ppm)

5-(2-Fluorophenyl)-3-(4-methoxyphenyl)-4-methyl-2-(trifluoromethyl)-3,6-dihydro-2*H*-1,3-oxazine (5al)

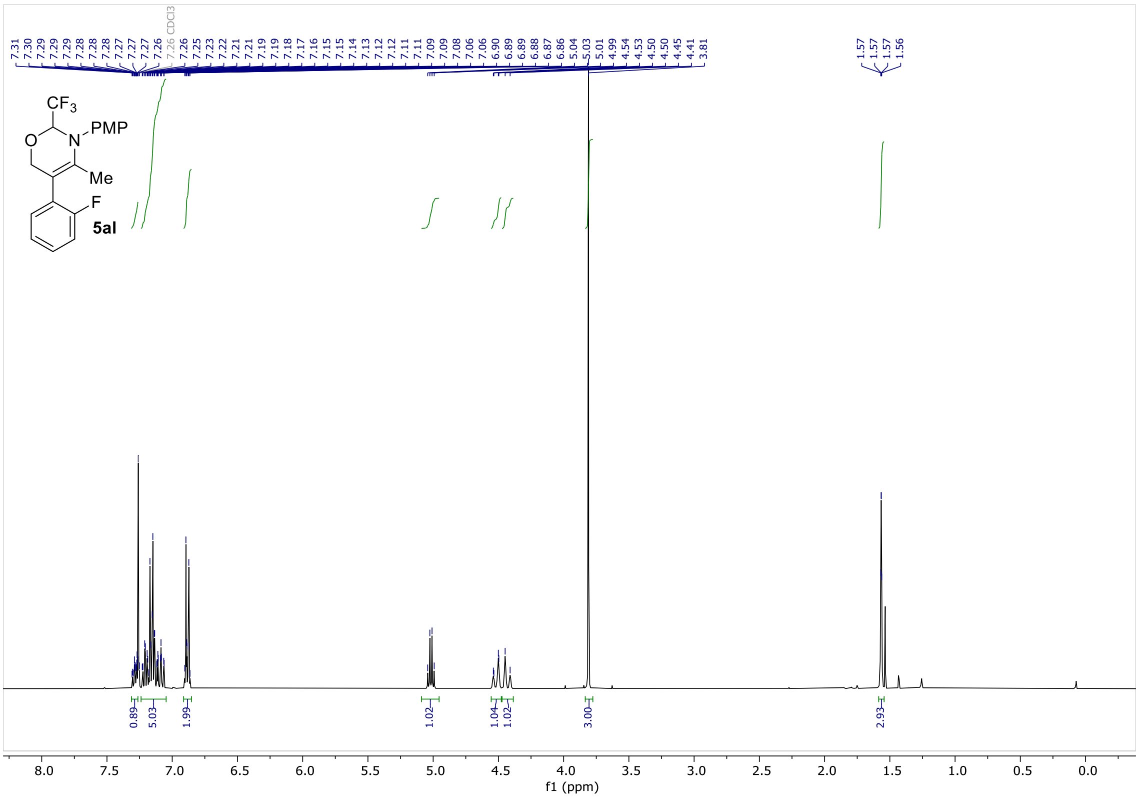

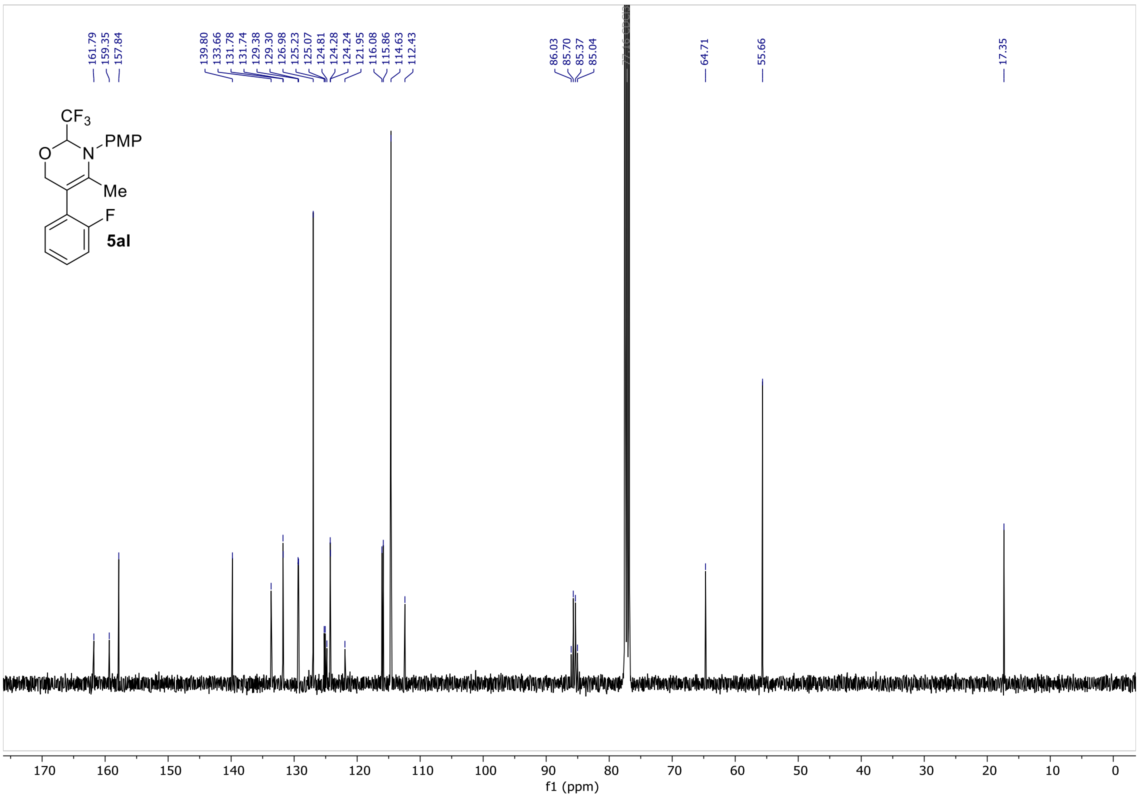

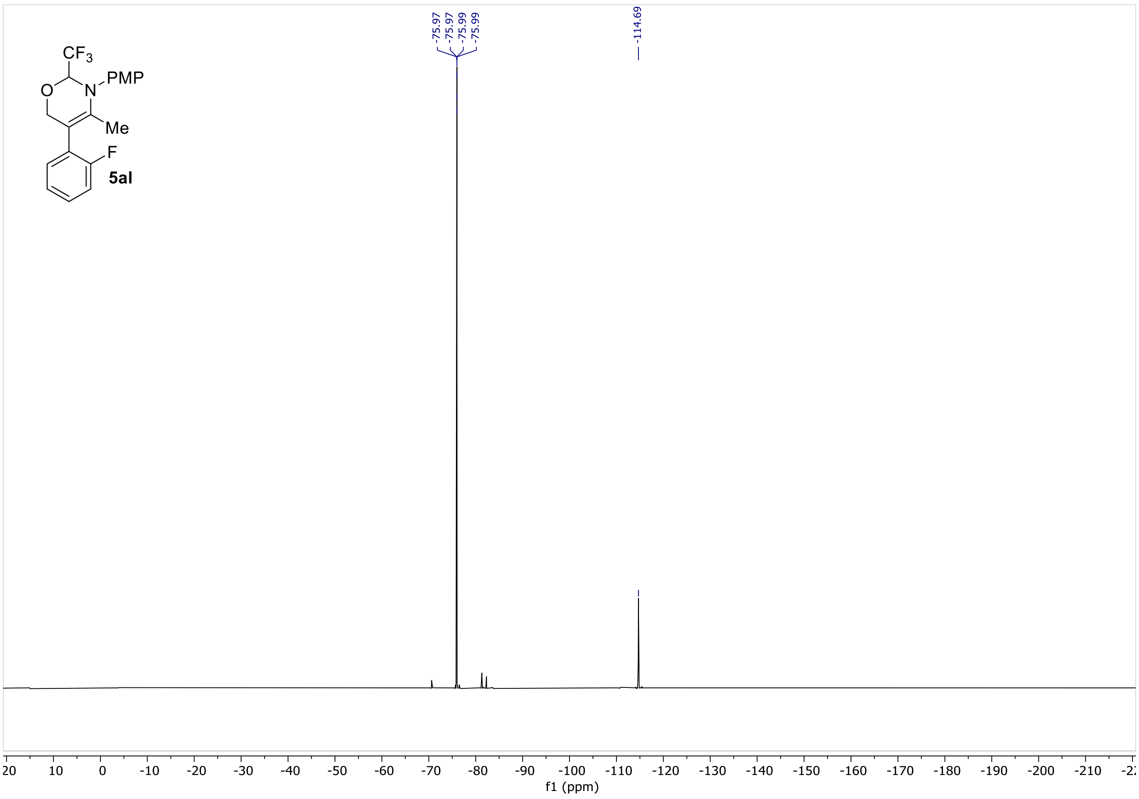

5-(2-Fluoropyridin-4-yl)-3-(4-methoxyphenyl)-4-methyl-2-(trifluoromethyl)-3,6-dihydro-2*H*-1,3-oxazine (5am)

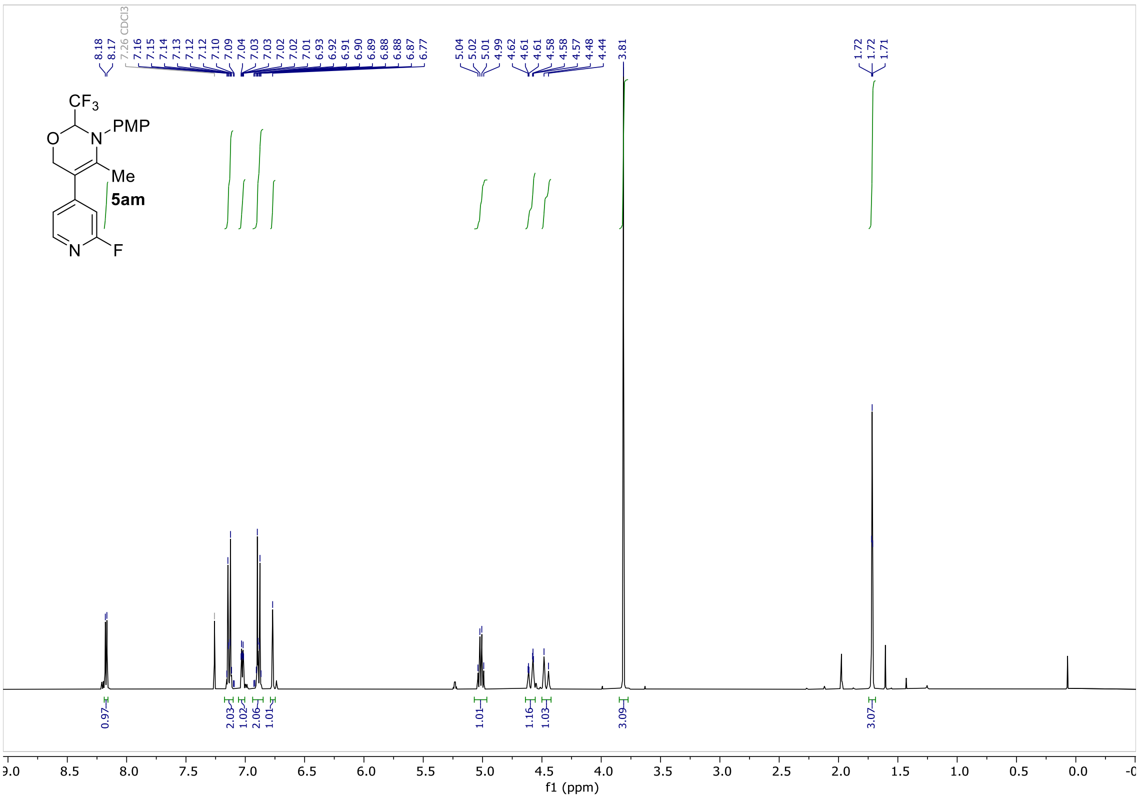

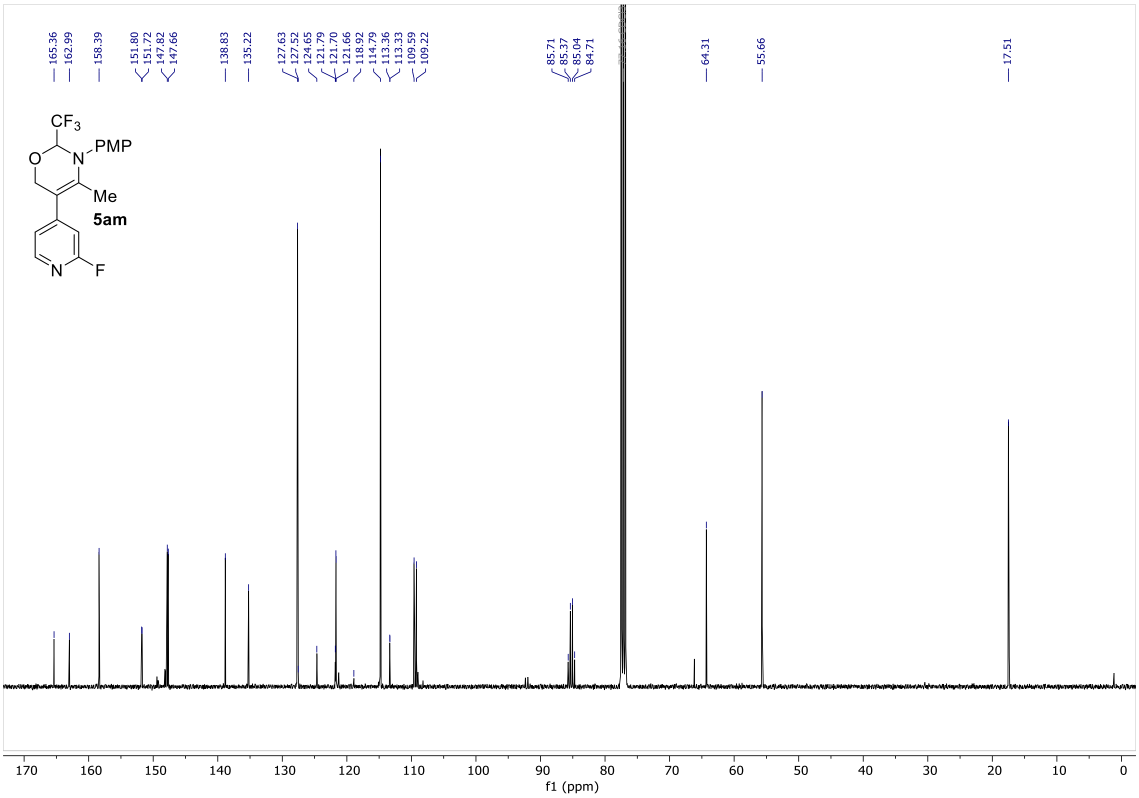

165.36
162.99
158.39
151.80
151.72
147.82
147.66
138.83
135.22
127.63
127.52
124.65
121.79
121.70
121.66
118.92
114.79
113.36
113.33
109.59
109.22
85.71
85.37
85.04
84.71
64.31
55.66
17.51
CF3
O
N
PMP
Me
5am
N
F
170 160 150 140 130 120 110 100 90 80 70 60 50 40 30 20 10 0
f1 (ppm)

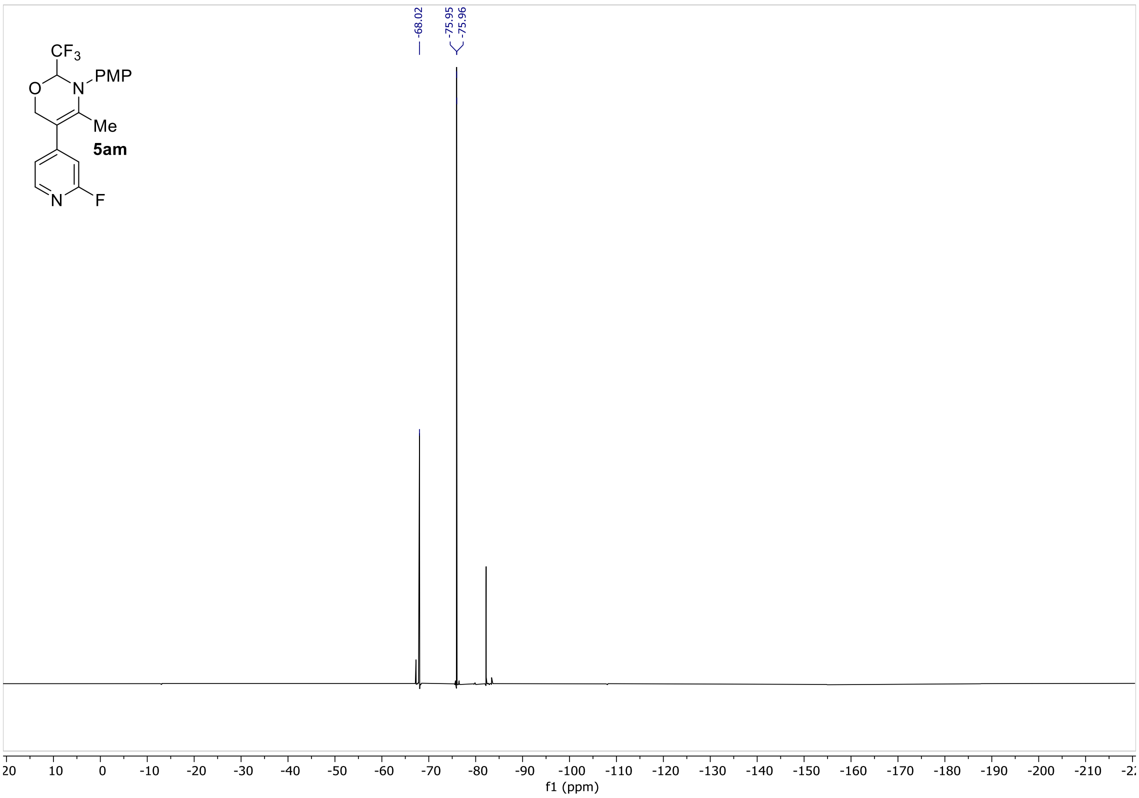

-68.02
-75.95
-75.96
CF3
O
N
PMP
Me
5am
N
F
20 10 0 -10 -20 -30 -40 -50 -60 -70 -80 -90 -100 -110 -120 -130 -140 -150 -160 -170 -180 -190 -200 -210
f1 (ppm)

3,4-Bis(4-methoxyphenyl)-5-(*p*-tolyl)-2-(trifluoromethyl)-3,6-dihydro-2*H*-1,3-oxazine (5cf)

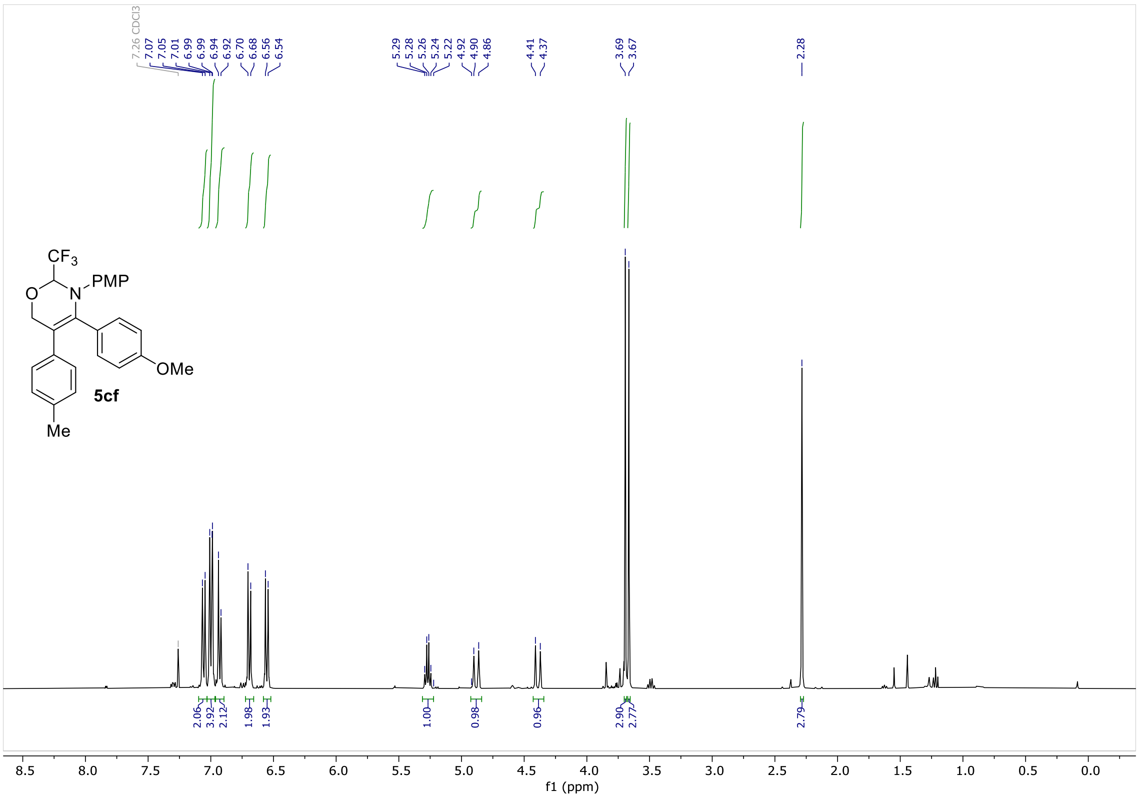

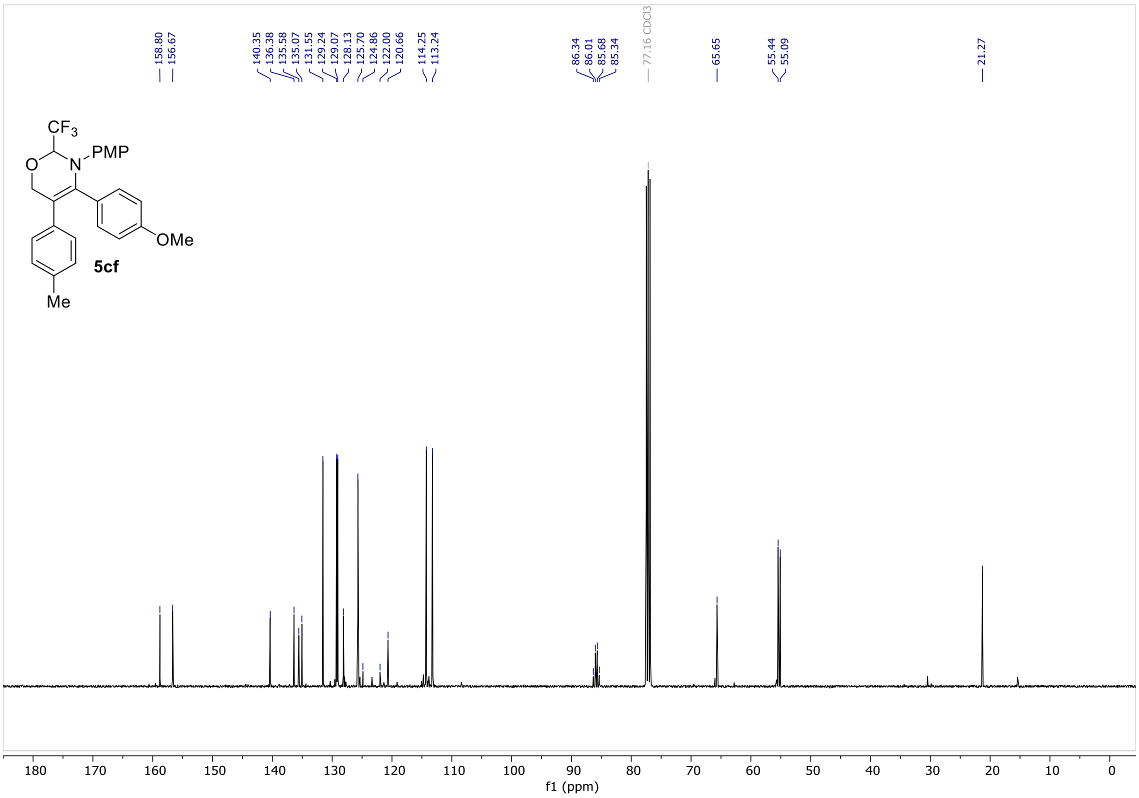

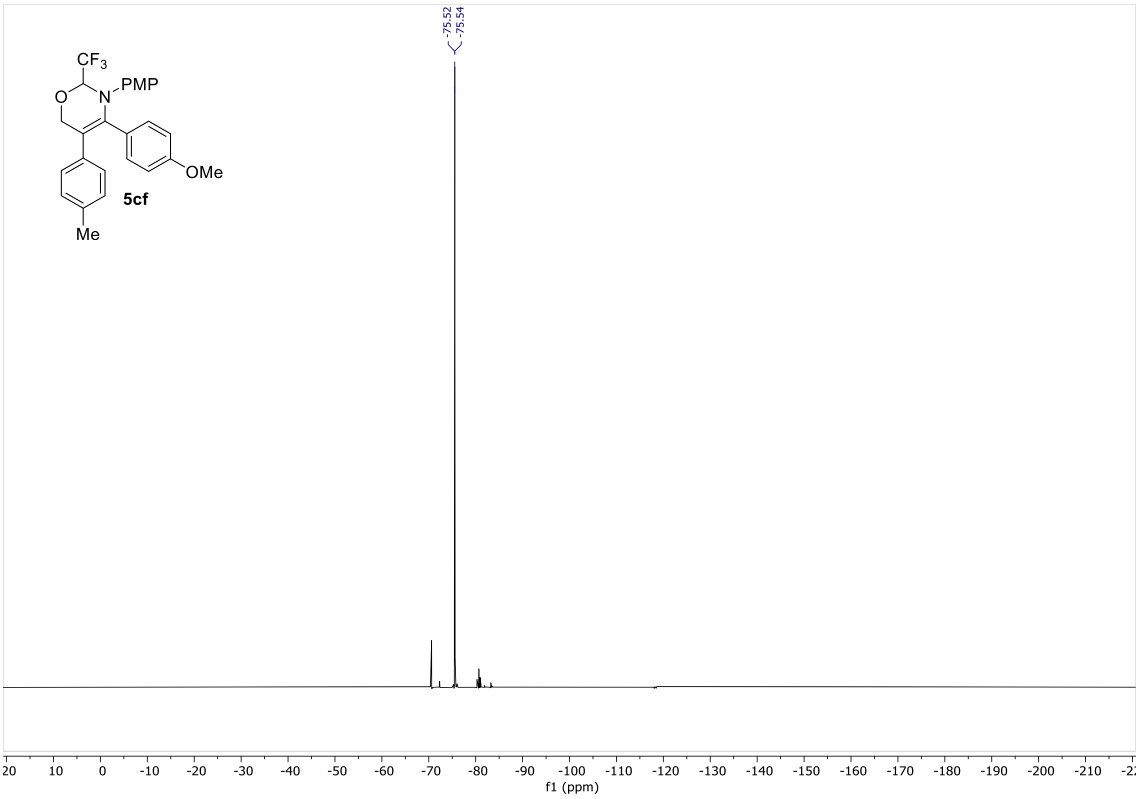

I.2. 5-*exo-dig* carboamination products

(*E*)-3-Tosyl-2-(trifluoromethyl)-4-(4-(triisopropylsilyl)but-3-yn-2-ylidene)oxazolidine (4aaf)

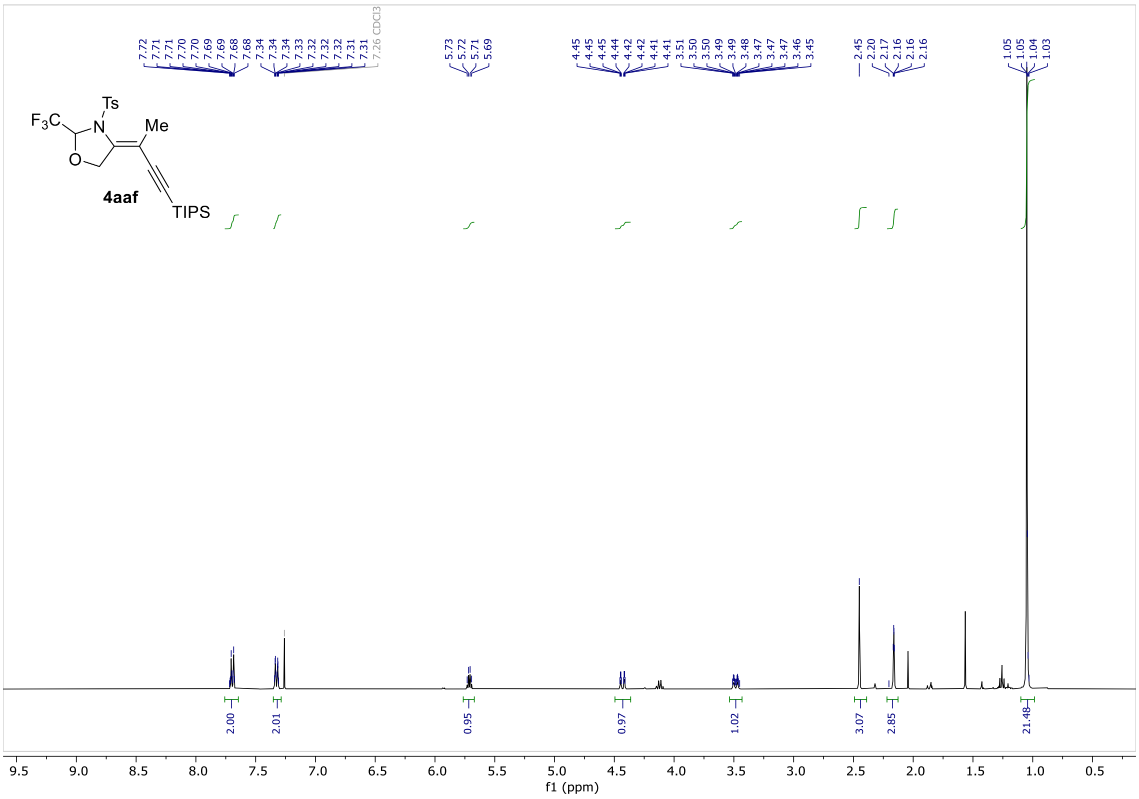

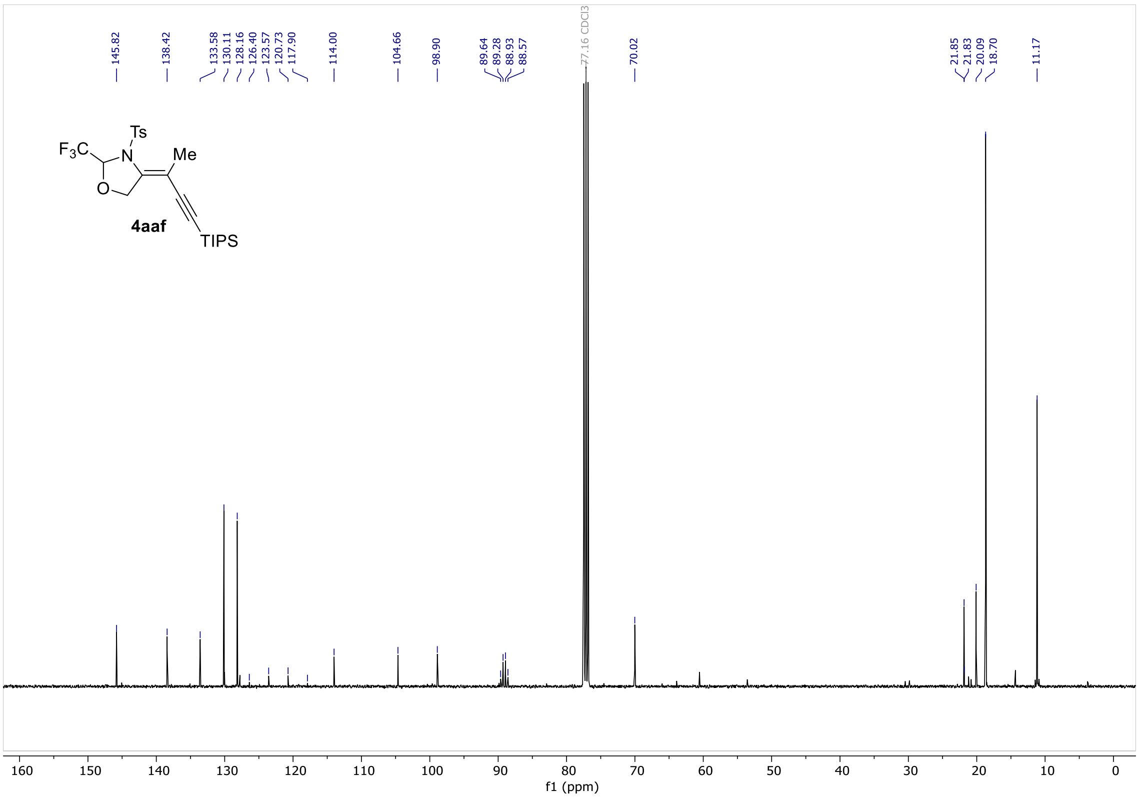
145.82
138.42
133.58
130.11
128.16
126.40
123.57
120.73
117.90
114.00
104.66
98.90
89.64
89.28
88.93
88.57
77.16 CDCl3
70.02
21.85
21.83
20.09
18.70
11.17
Ts
F3C
N
Me
O
4aaf
TIPS
f1 (ppm)

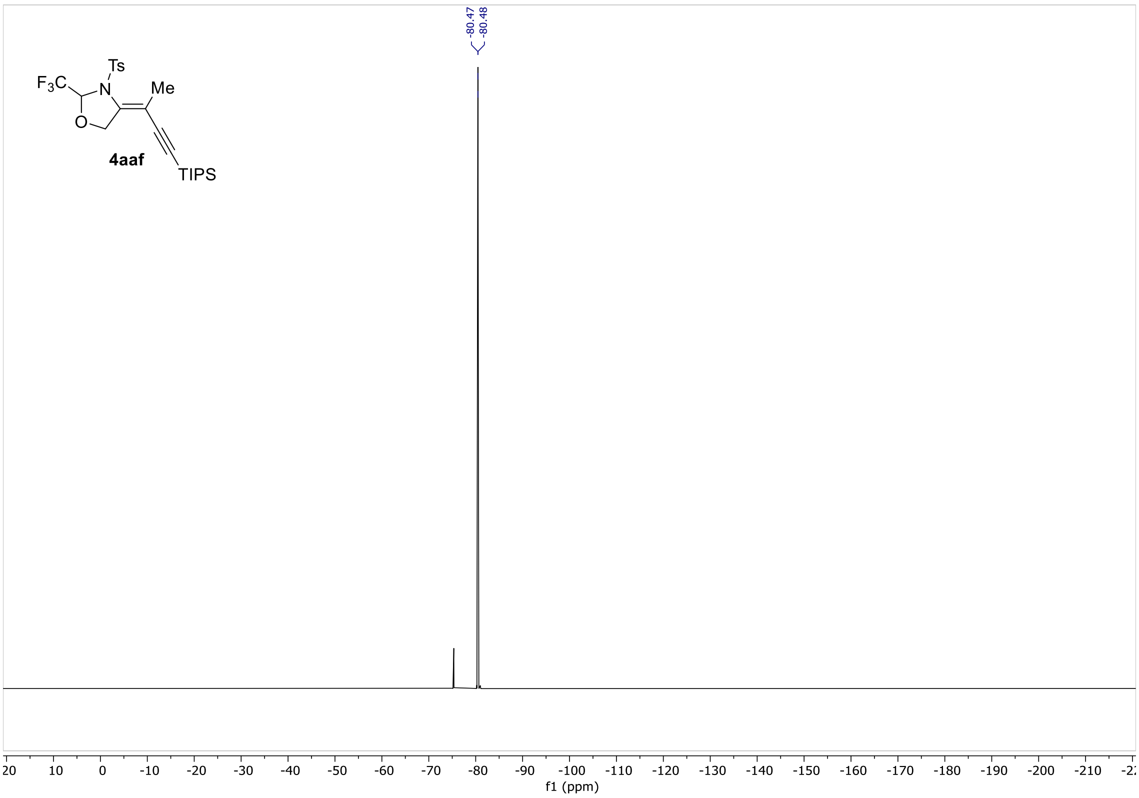
-80.47
-80.48
Ts
F3C
N
Me
O
4aaf
TIPS
f1 (ppm)

(*E*)-3-((4-Nitrophenyl)sulfonyl)-2-(trifluoromethyl)-4-(4-(triisopropylsilyl)but-3-yn-2-ylidene)oxazolidine (4aag)

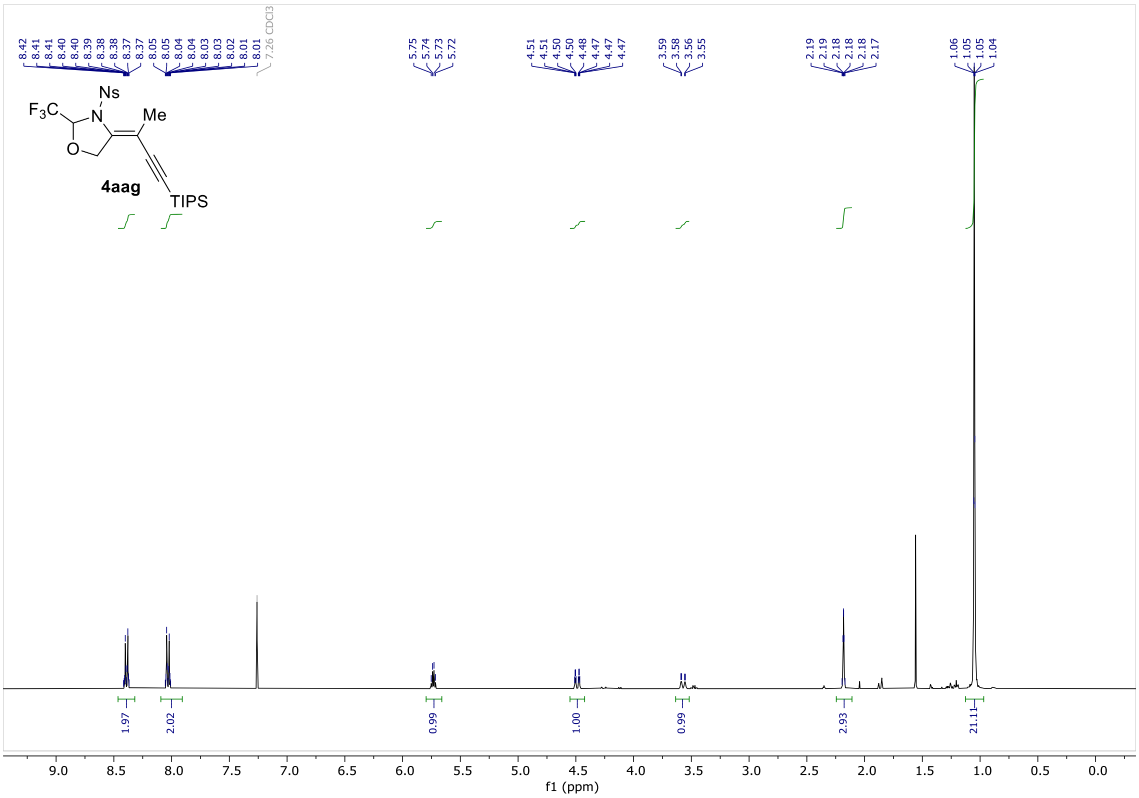

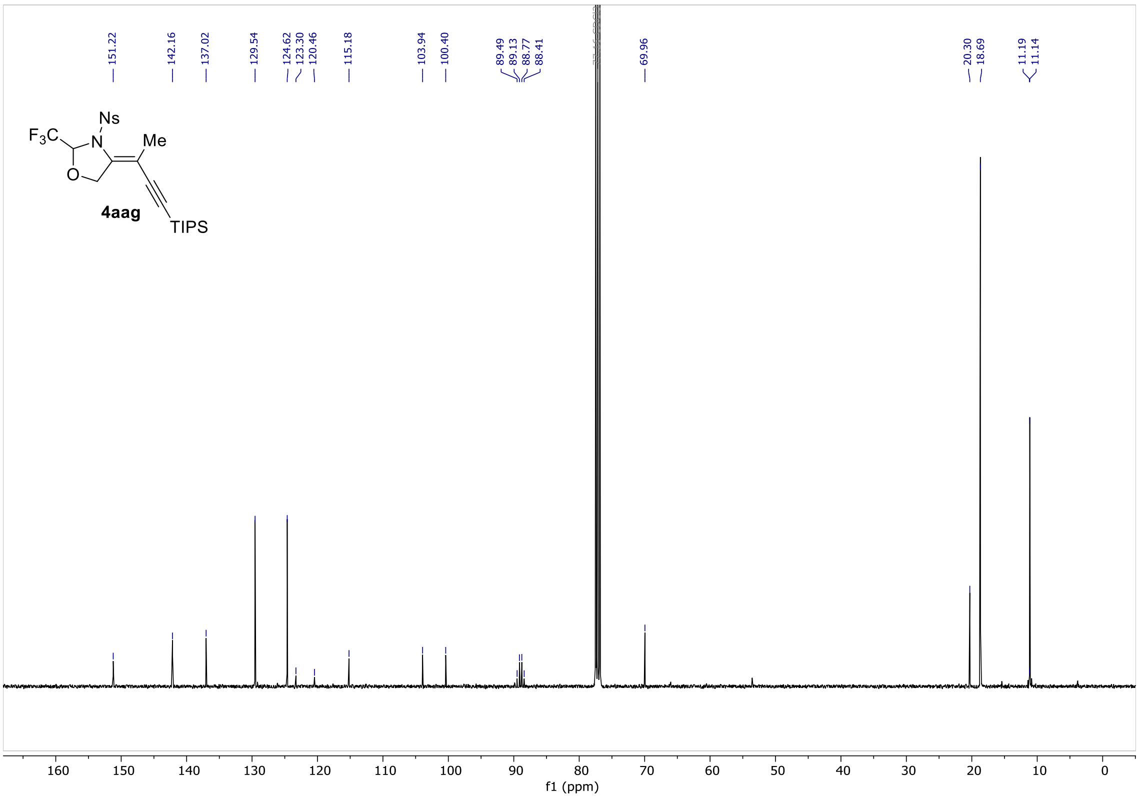

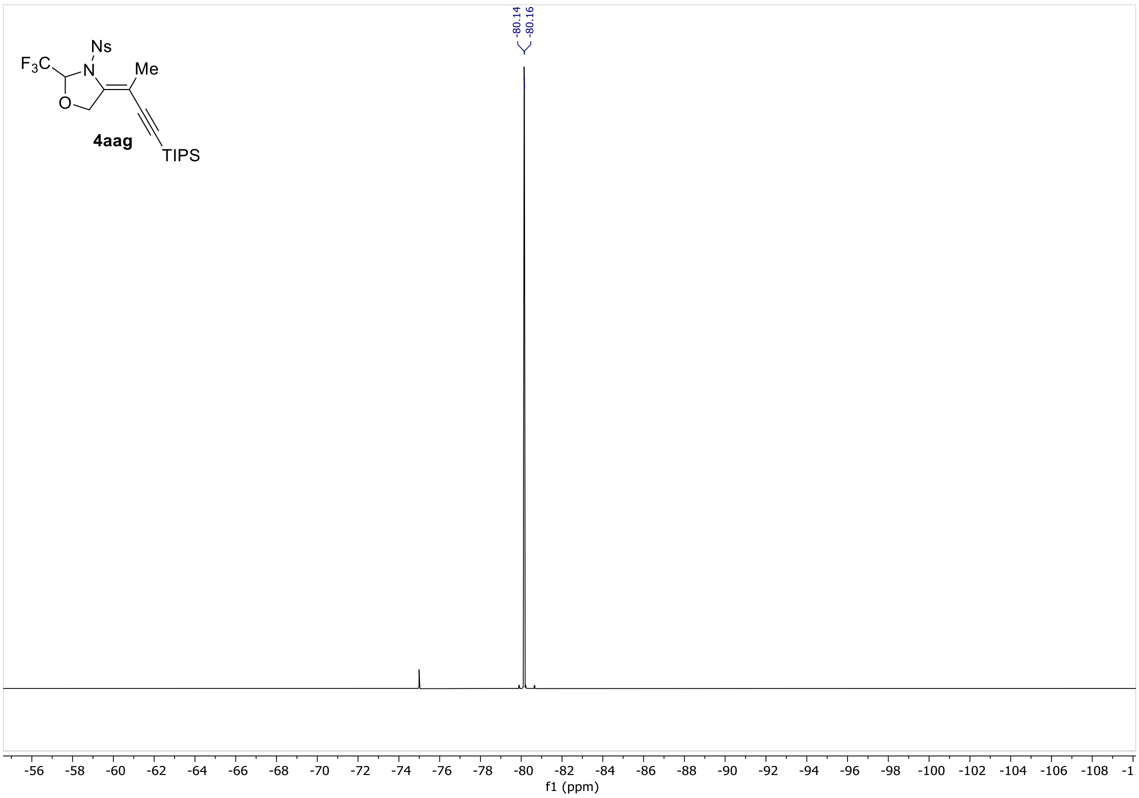

(*E*)-4-(1-Phenyl-3-(triisopropylsilyl)prop-2-yn-1-ylidene)-3-tosyl-2-(trifluoromethyl)oxazolidine (4baf)

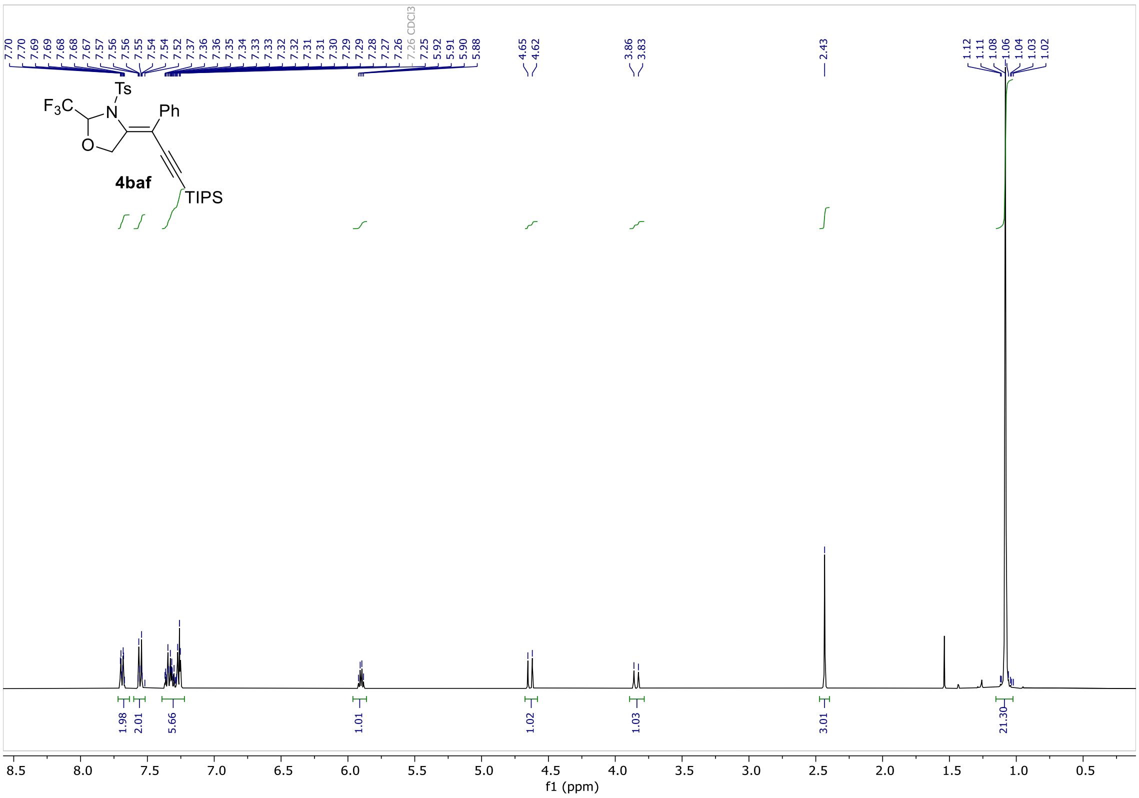

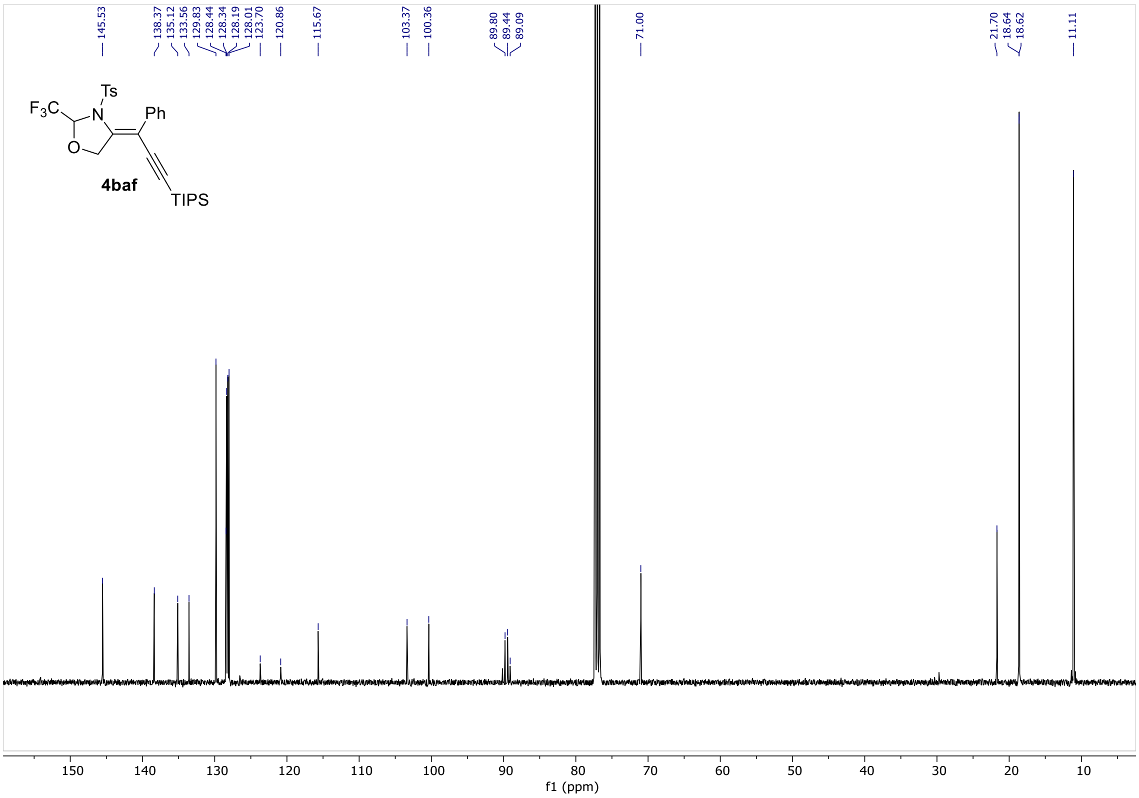
145.53
138.37
135.12
133.56
129.83
128.44
128.34
128.19
128.01
123.70
120.86
115.67
103.37
100.36
89.80
89.44
89.09
71.00
21.70
18.64
18.62
11.11
F3C
N
Ts
Ph
O
4baf
TIPS
150
140
130
120
110
100
90
80
70
60
50
40
30
20
10
f1 (ppm)

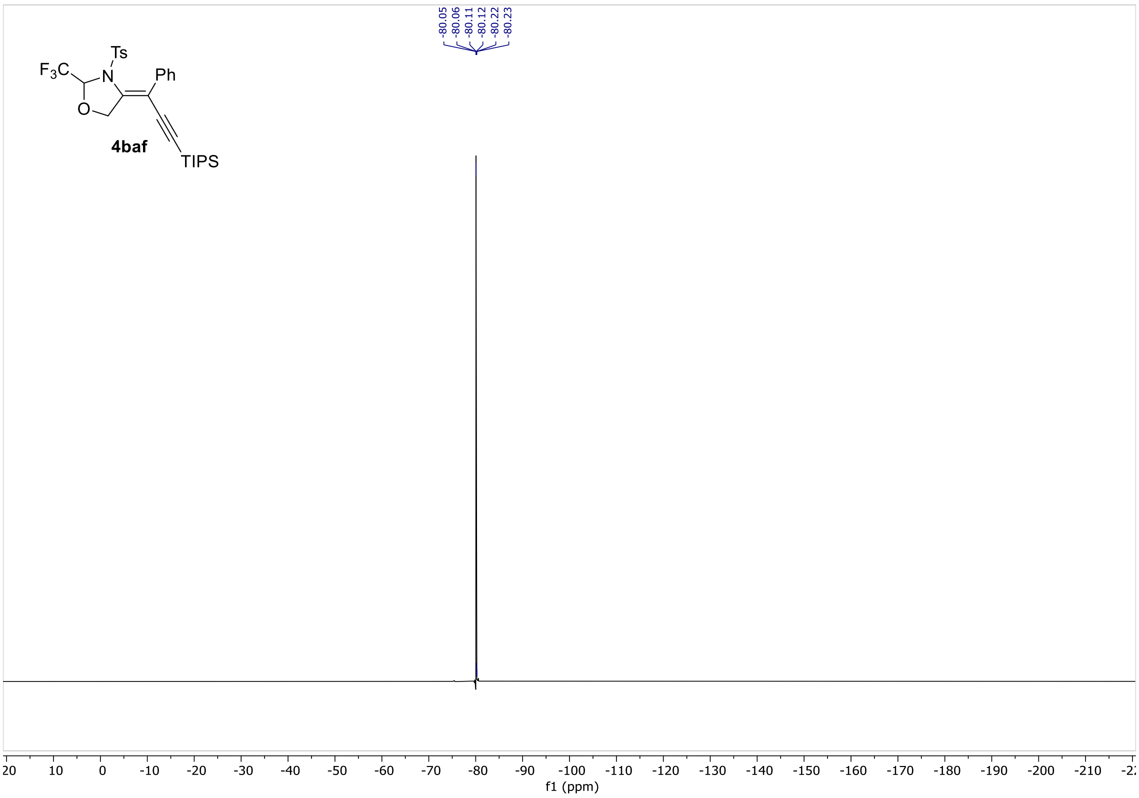
-80.05
-80.06
-80.11
-80.12
-80.22
-80.23
F3C
N
Ts
Ph
O
4baf
TIPS
20
10
0
-10
-20
-30
-40
-50
-60
-70
-80
-90
-100
-110
-120
-130
-140
-150
-160
-170
-180
-190
-200
-210
f1 (ppm)

(*E*)-4-(1-(4-Methoxyphenyl)-3-(triisopropylsilyl)prop-2-yn-1-ylidene)-3-tosyl-2-(trifluoromethyl)oxazolidine (4caf)

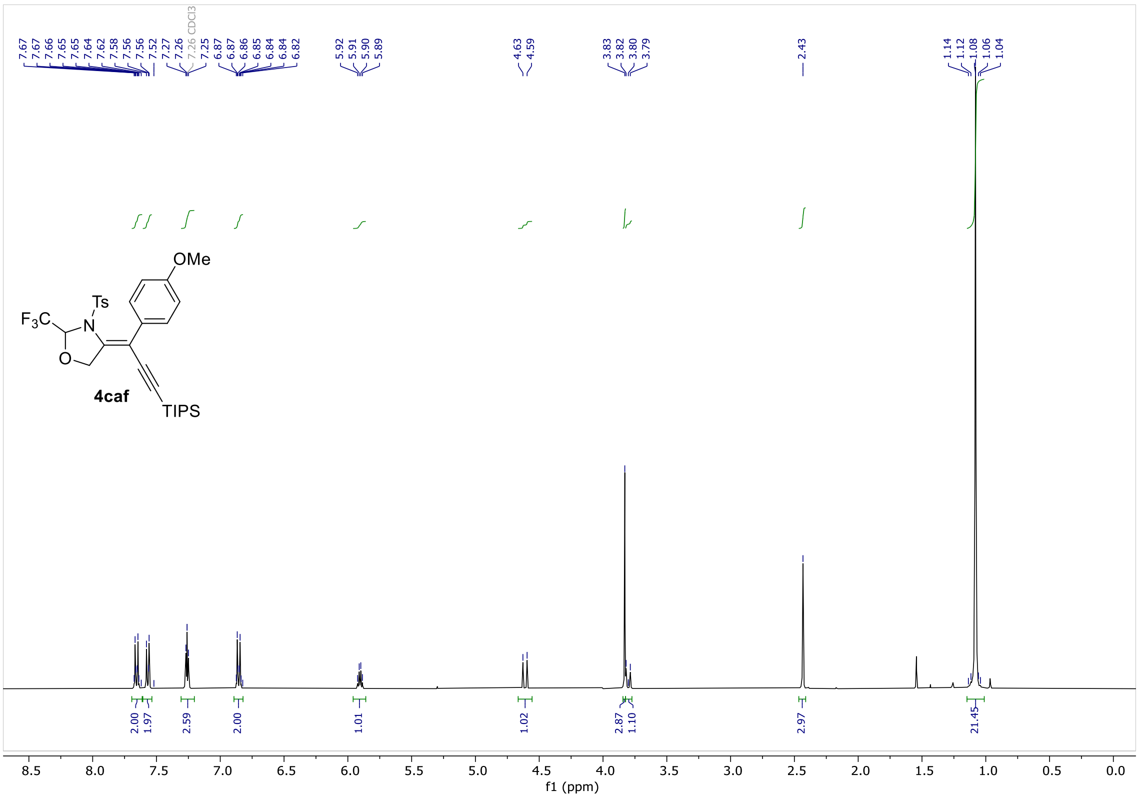

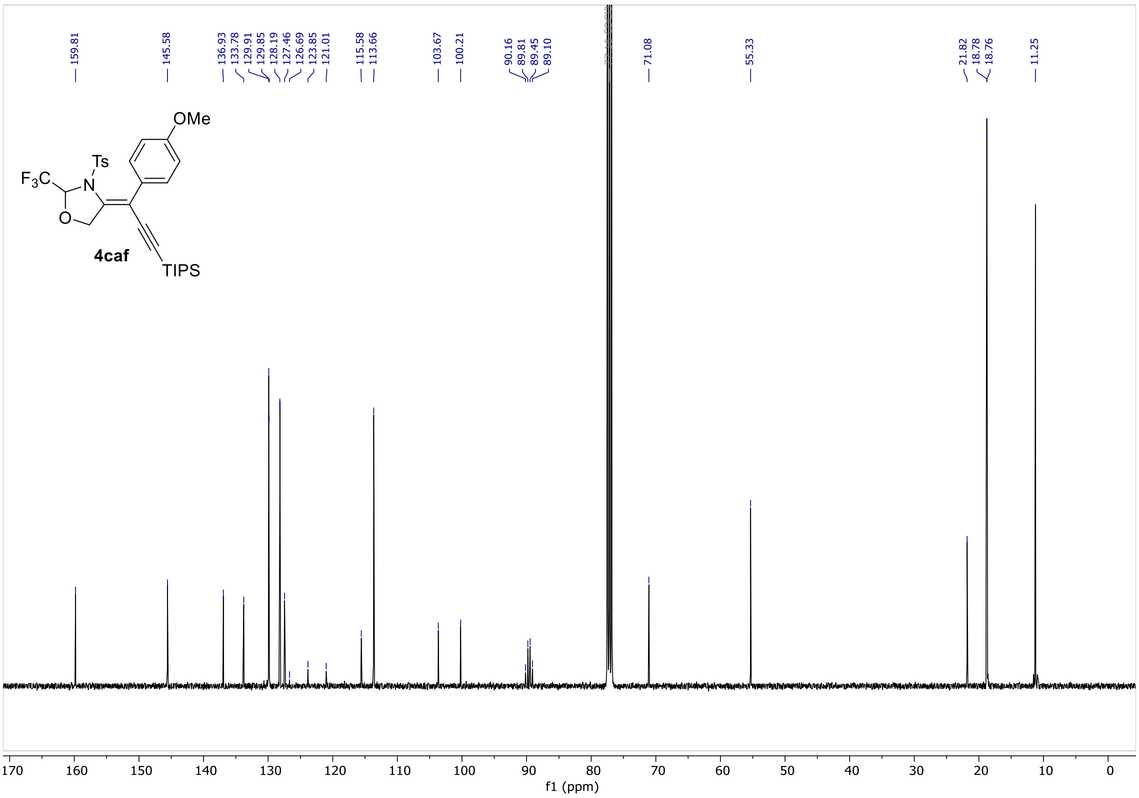

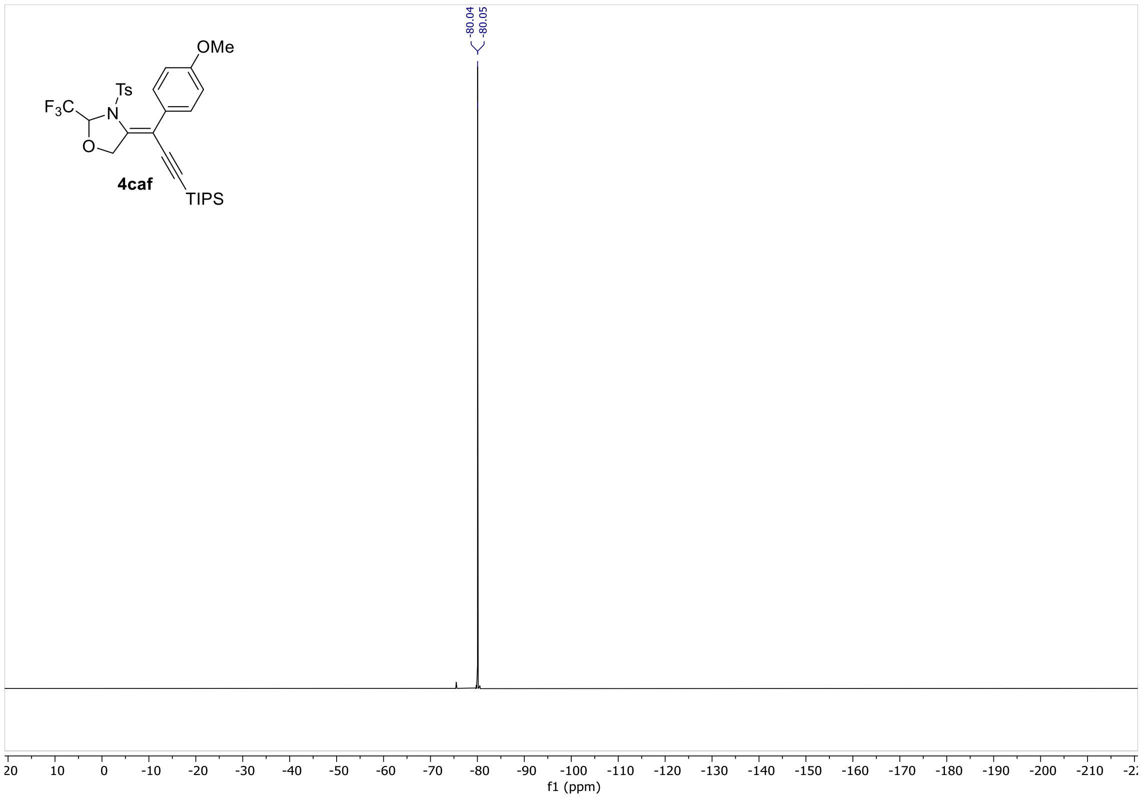

(*E*)-3-Tosyl-2-(trifluoromethyl)-4-(1-(4-(trifluoromethyl)phenyl)-3-(triisopropylsilyl)prop-2-yn-1-ylidene)oxazolidine (4faf)

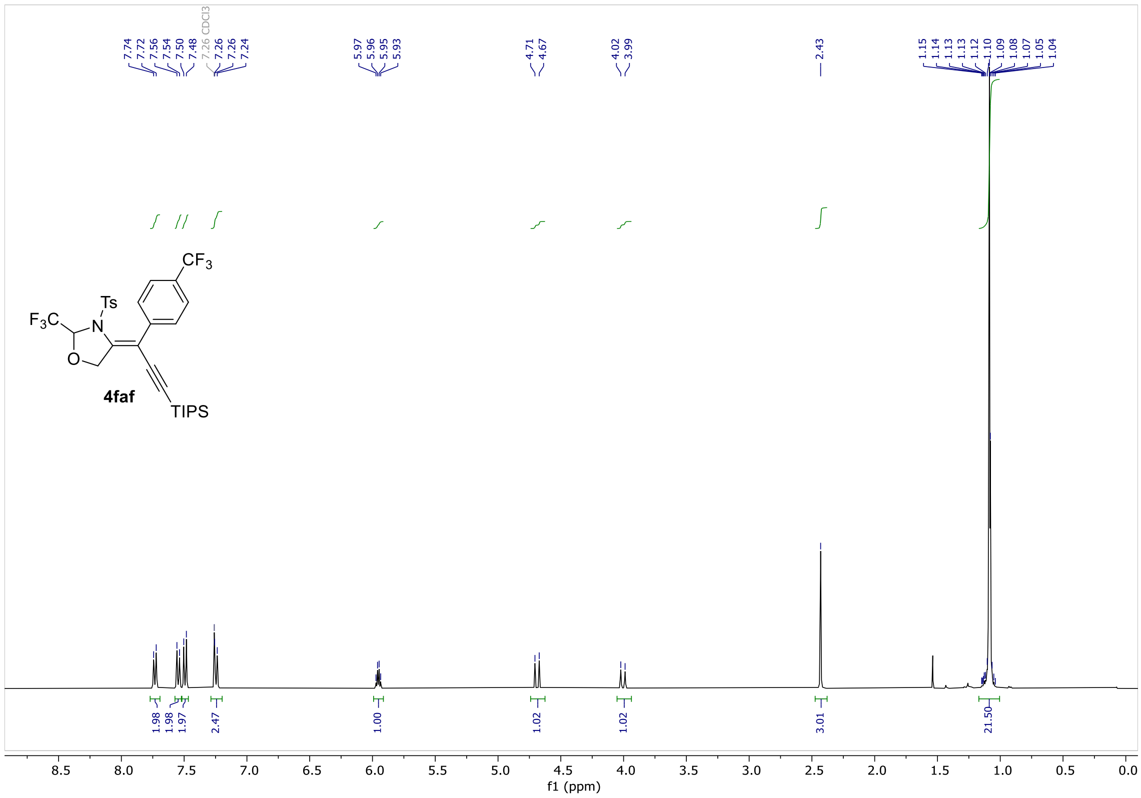

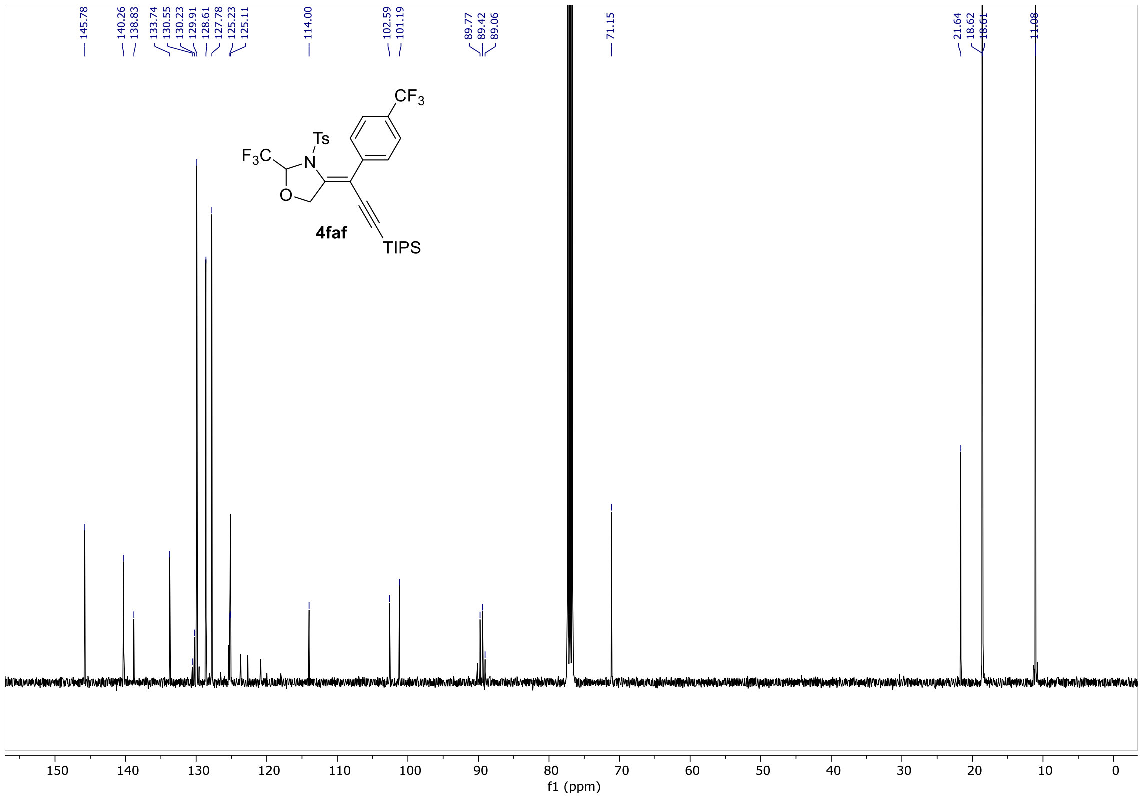

145.78
140.26
138.83
133.74
130.55
130.23
129.91
128.61
127.78
125.23
125.11
114.00
102.59
101.19
89.77
89.42
89.06
71.15
21.64
18.62
18.61
11.08
CF3
Ts
F3C
N
O
4faf
TIPS
150 140 130 120 110 100 90 80 70 60 50 40 30 20 10 0
f1 (ppm)

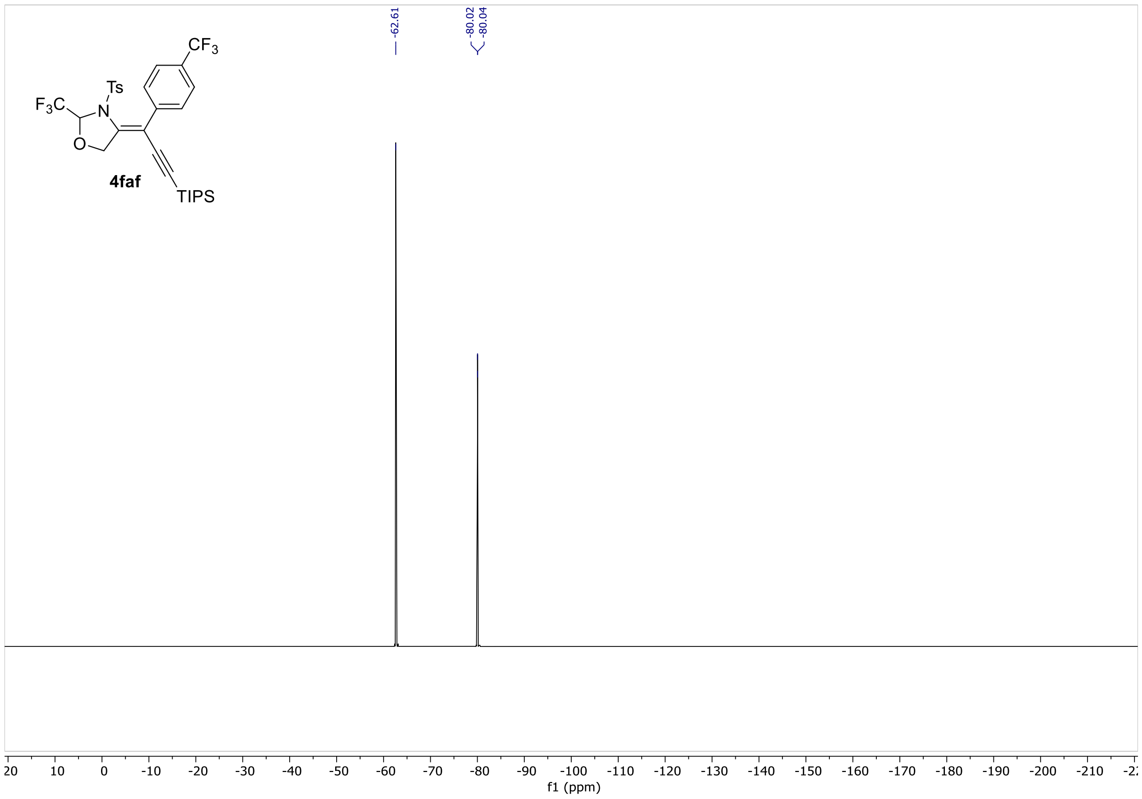

-62.61
-80.02
-80.04
CF3
Ts
F3C
N
O
4faf
TIPS
20 10 0 -10 -20 -30 -40 -50 -60 -70 -80 -90 -100 -110 -120 -130 -140 -150 -160 -170 -180 -190 -200 -210
f1 (ppm)

(*E*)-4-(1-(2-Methoxyphenyl)-3-(triisopropylsilyl)prop-2-yn-1-ylidene)-3-tosyl-2-(trifluoromethyl)oxazolidine (4naf)

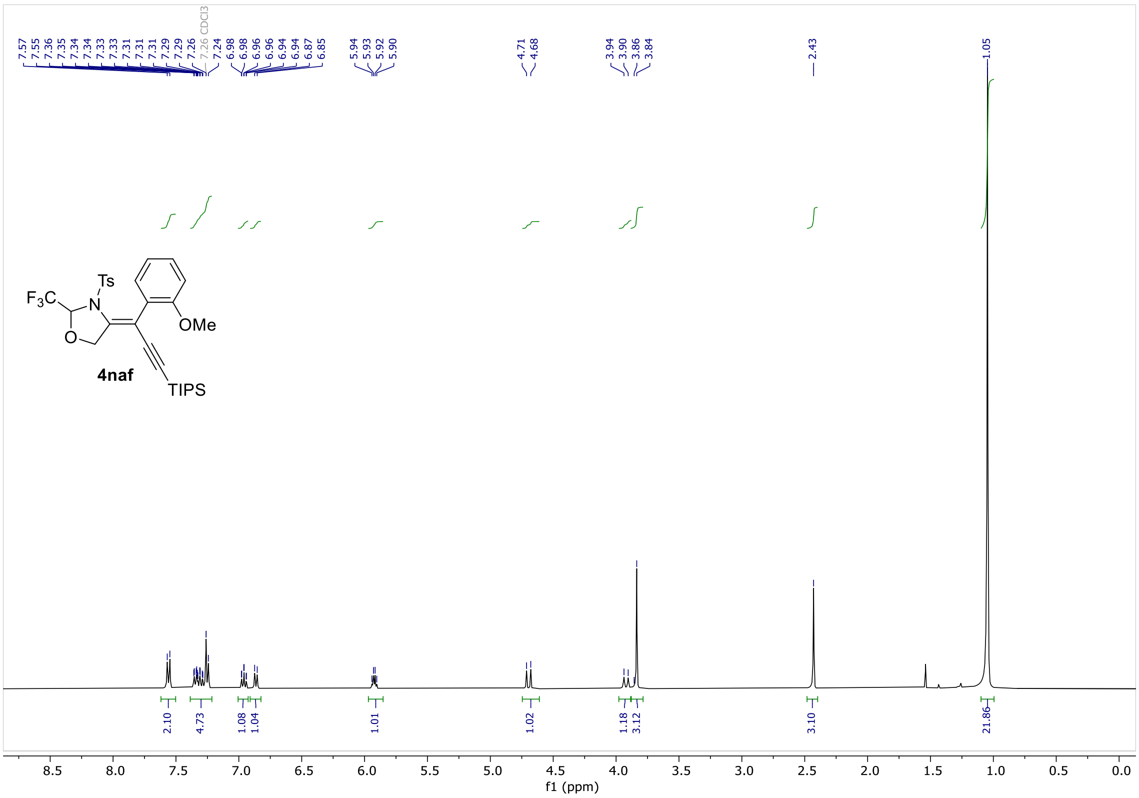

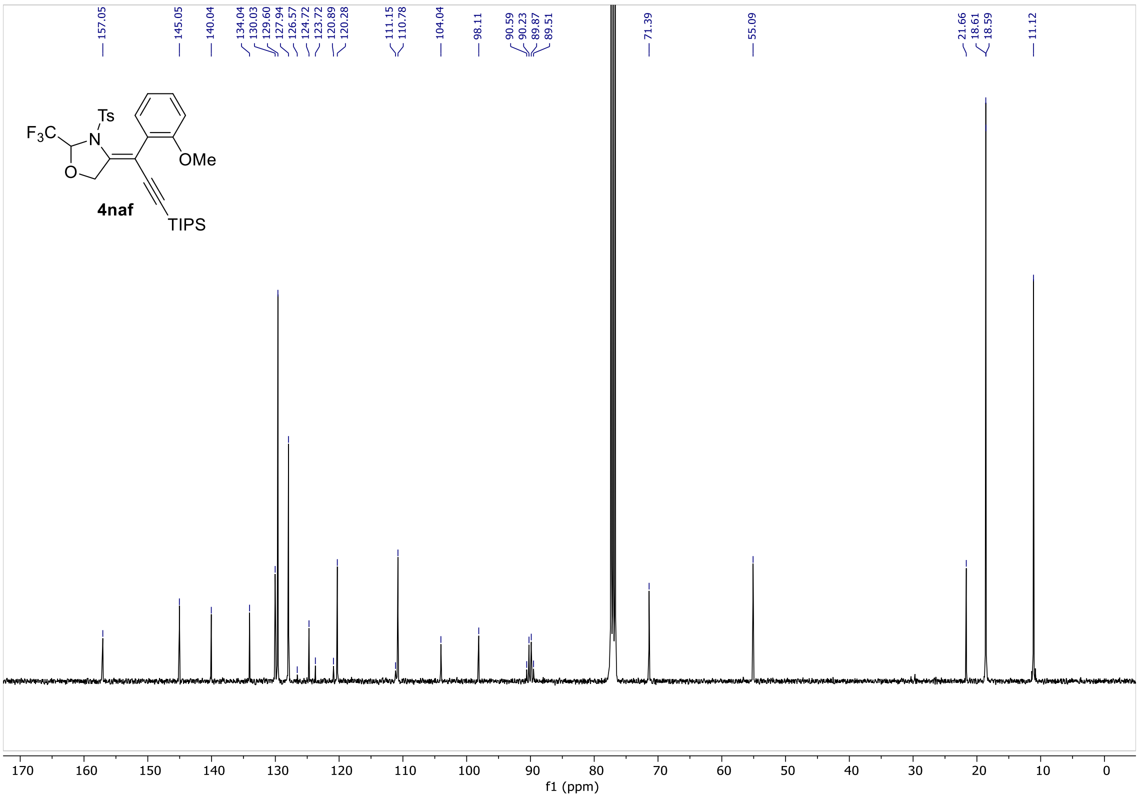

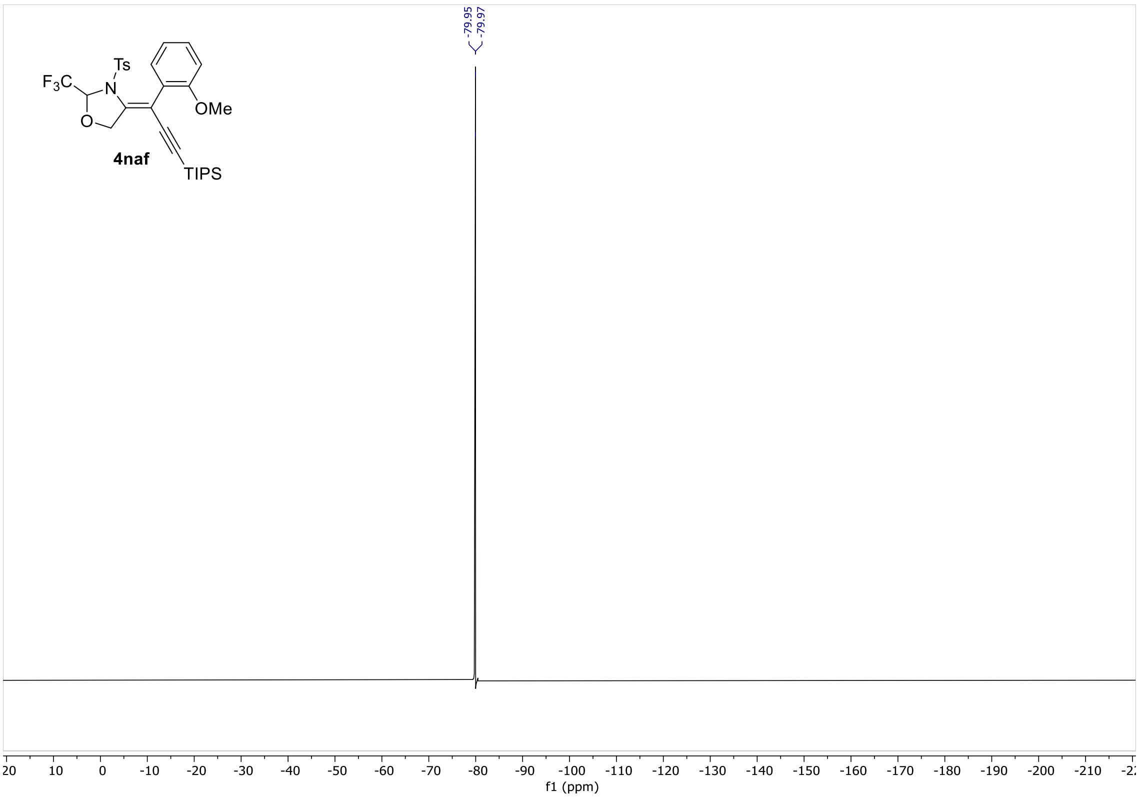

(*Z*)-4-(1-((*tert*-Butyldimethylsilyl)oxy)-4-(triisopropylsilyl)but-3-yn-2-ylidene)-3-tosyl-2-(trifluoromethyl)oxazolidine (4jaf)

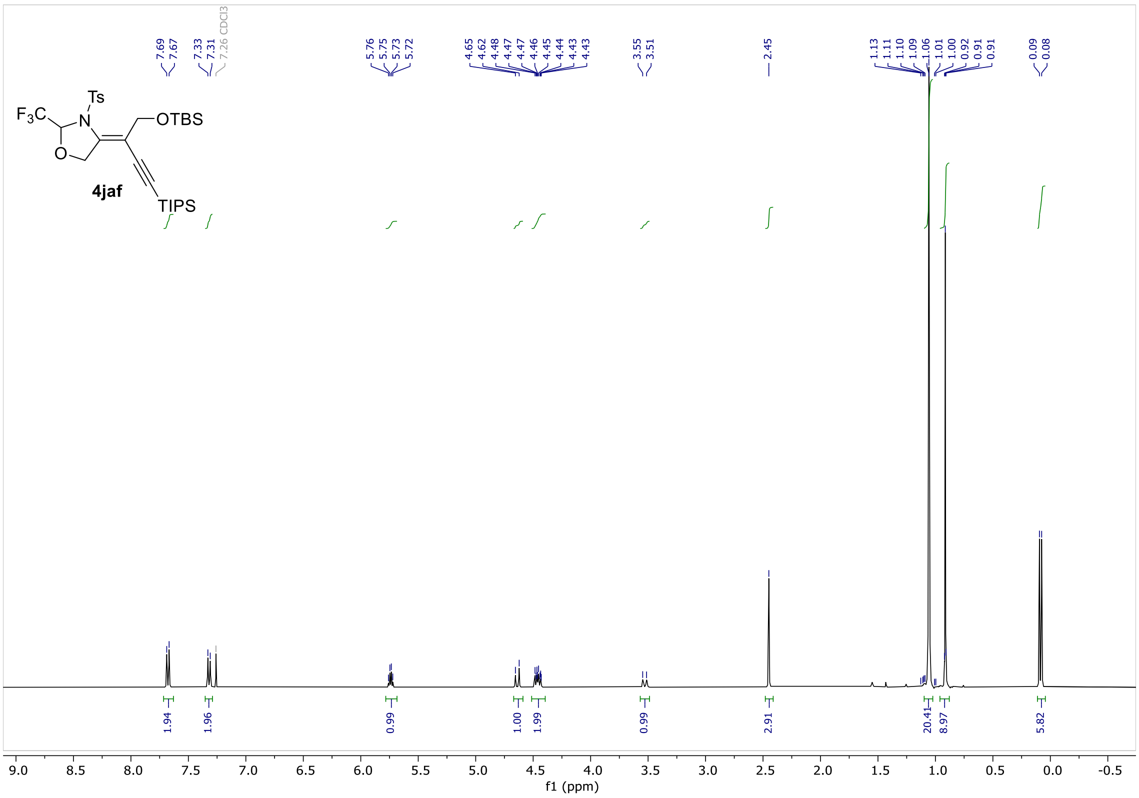

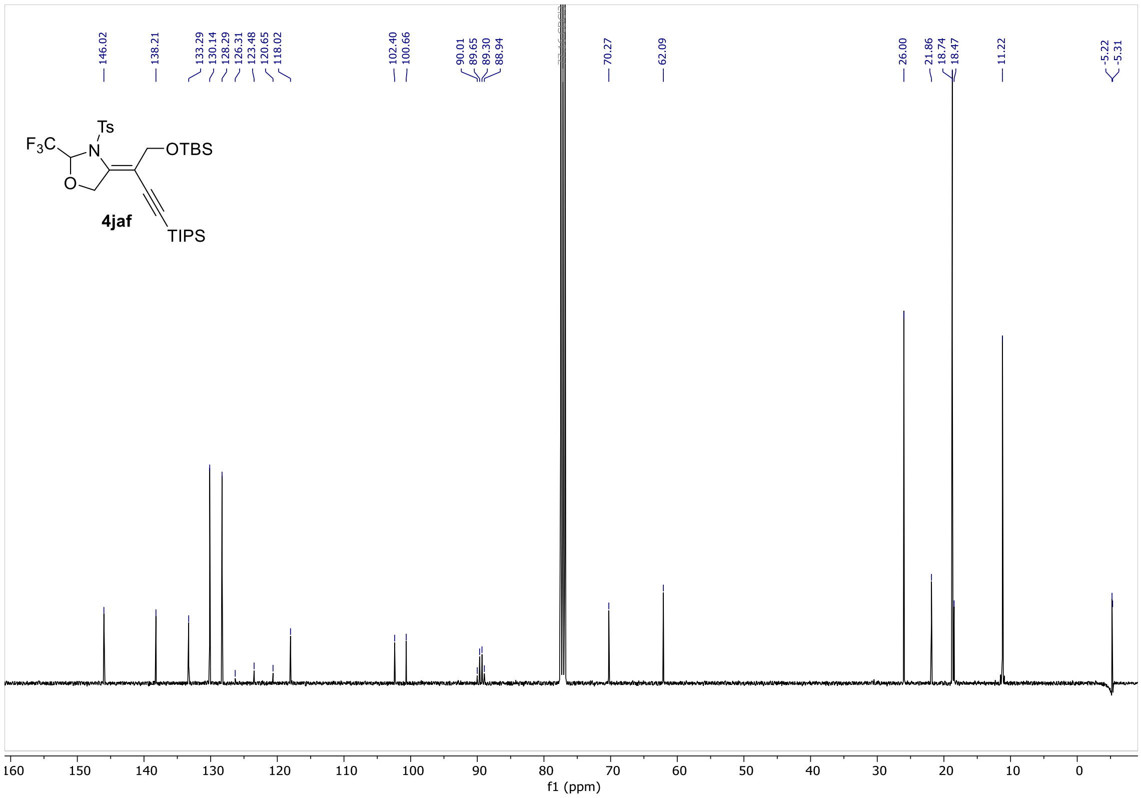
146.02
138.21
133.29
130.14
128.29
126.31
123.48
120.65
118.02
102.40
100.66
90.01
89.65
89.30
88.94
70.27
62.09
26.00
21.86
18.74
18.47
11.22
-5.22
-5.31
Ts
F3C
N
OTBS
O
4jaf
TIPS
f1 (ppm)

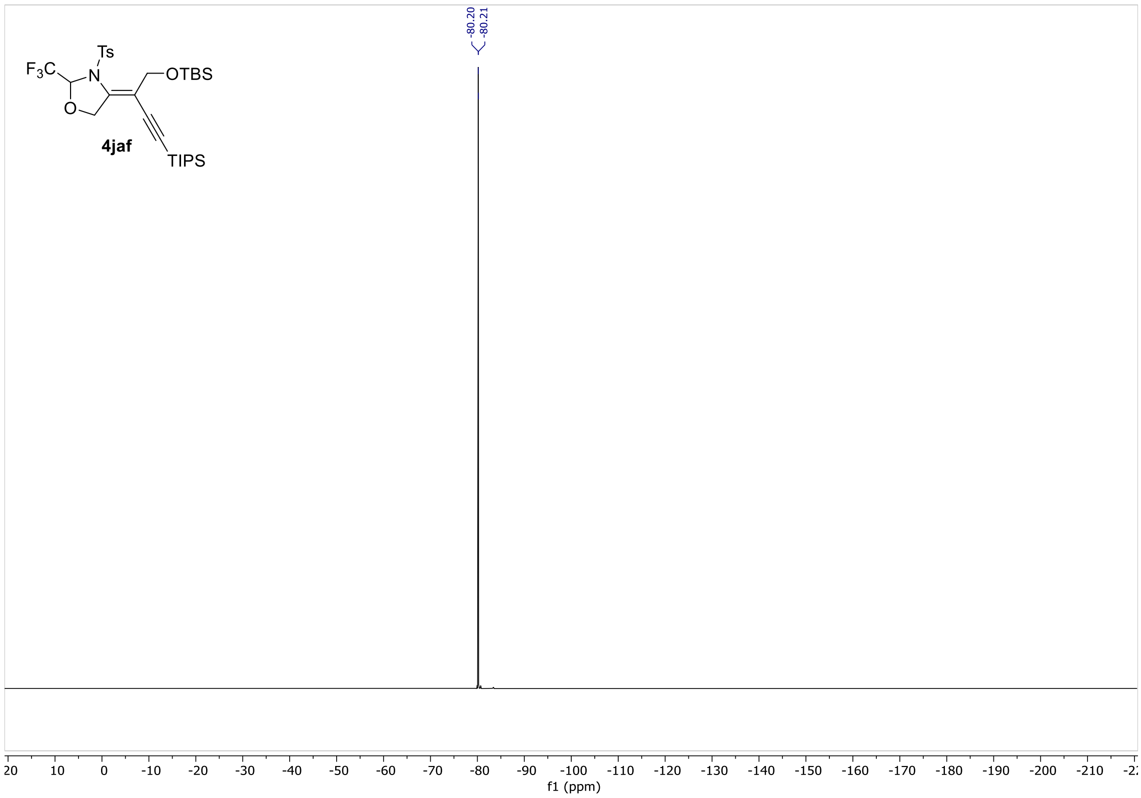
-80.20
-80.21
Ts
F3C
N
OTBS
O
4jaf
TIPS
f1 (ppm)

(*E*)-5-Methyl-3-tosyl-2-(trifluoromethyl)-4-(4-(triisopropylsilyl)but-3-yn-2-ylidene)oxazolidine (4laf)

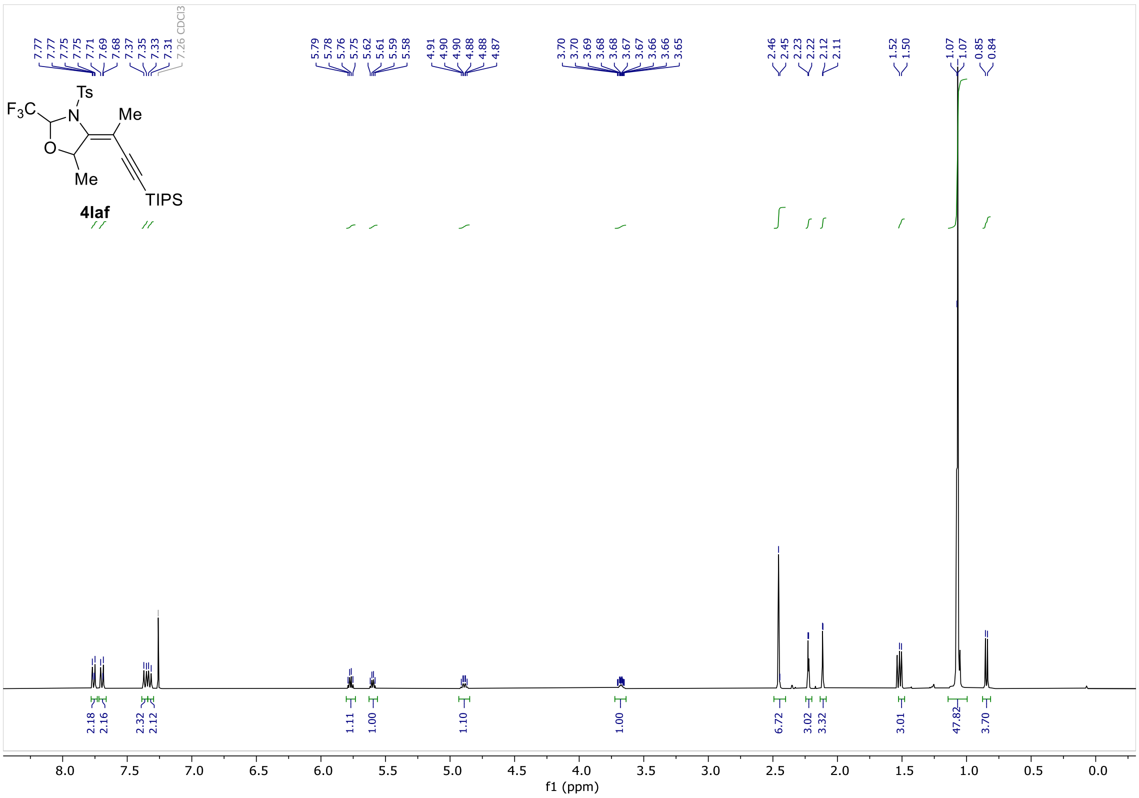

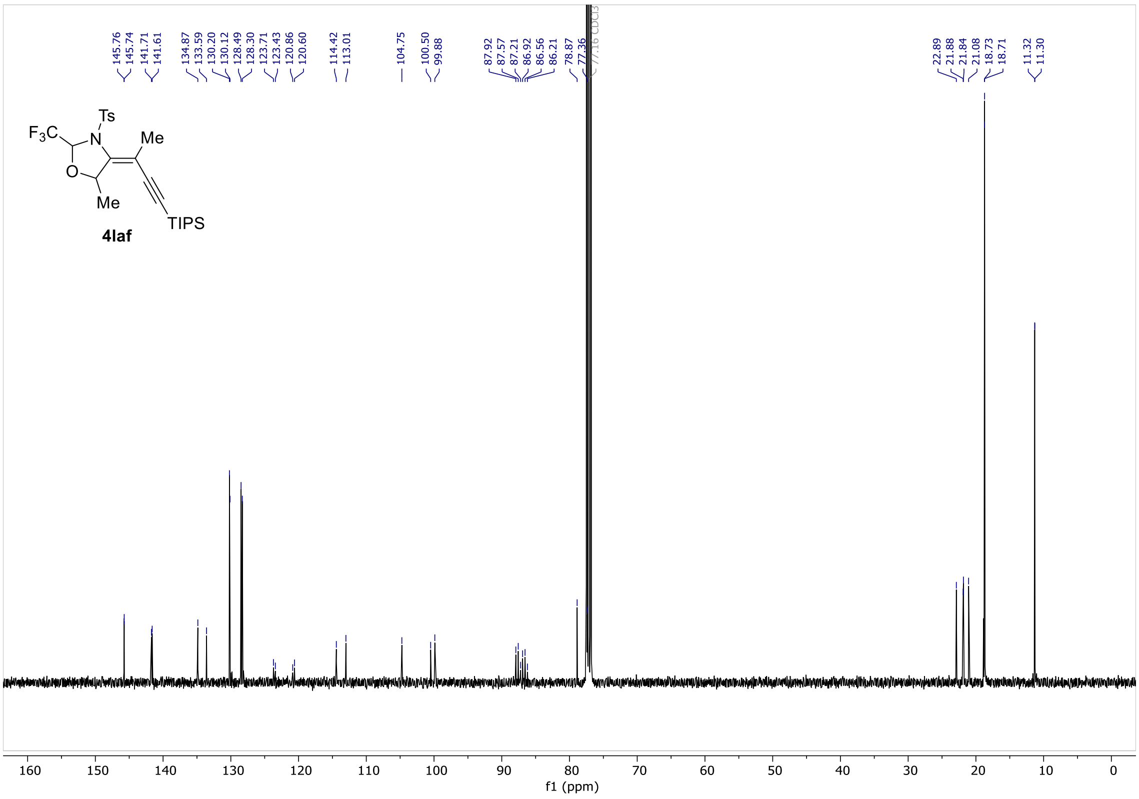

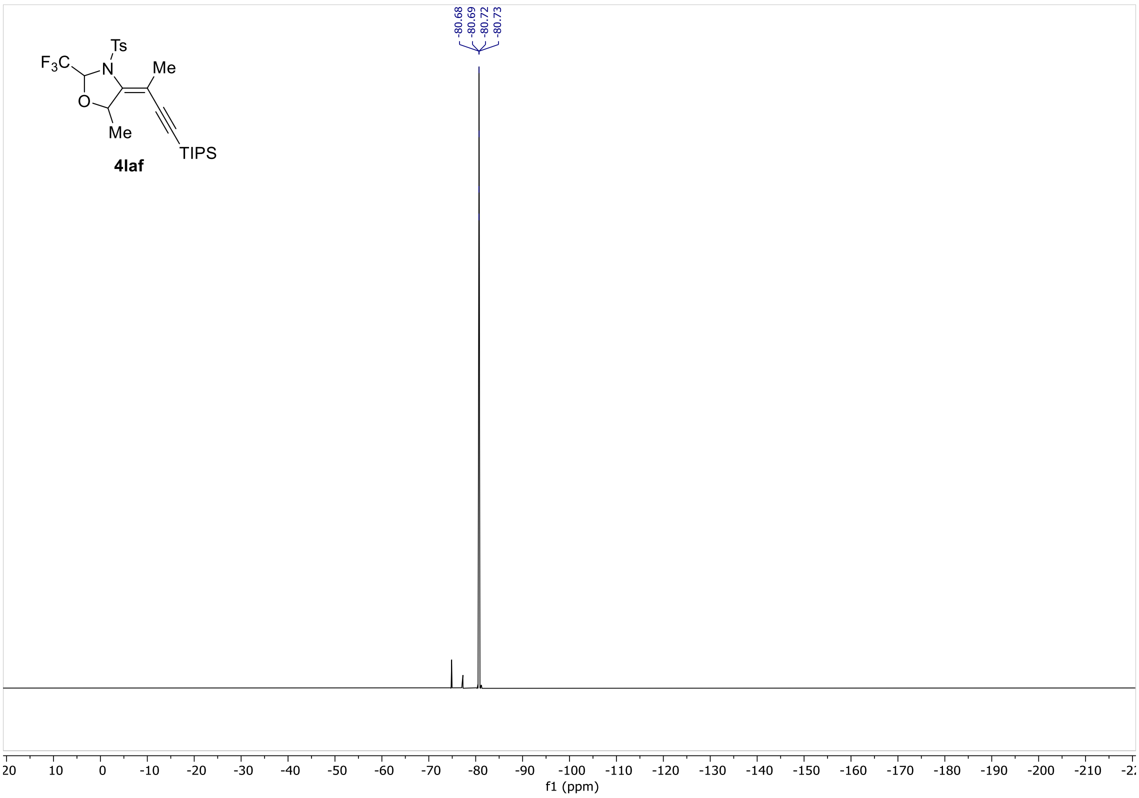

(*E*)-4-(1-(*p*-Tolyl)ethylidene)-3-tosyl-2-(trifluoromethyl)oxazolidine (4aff)

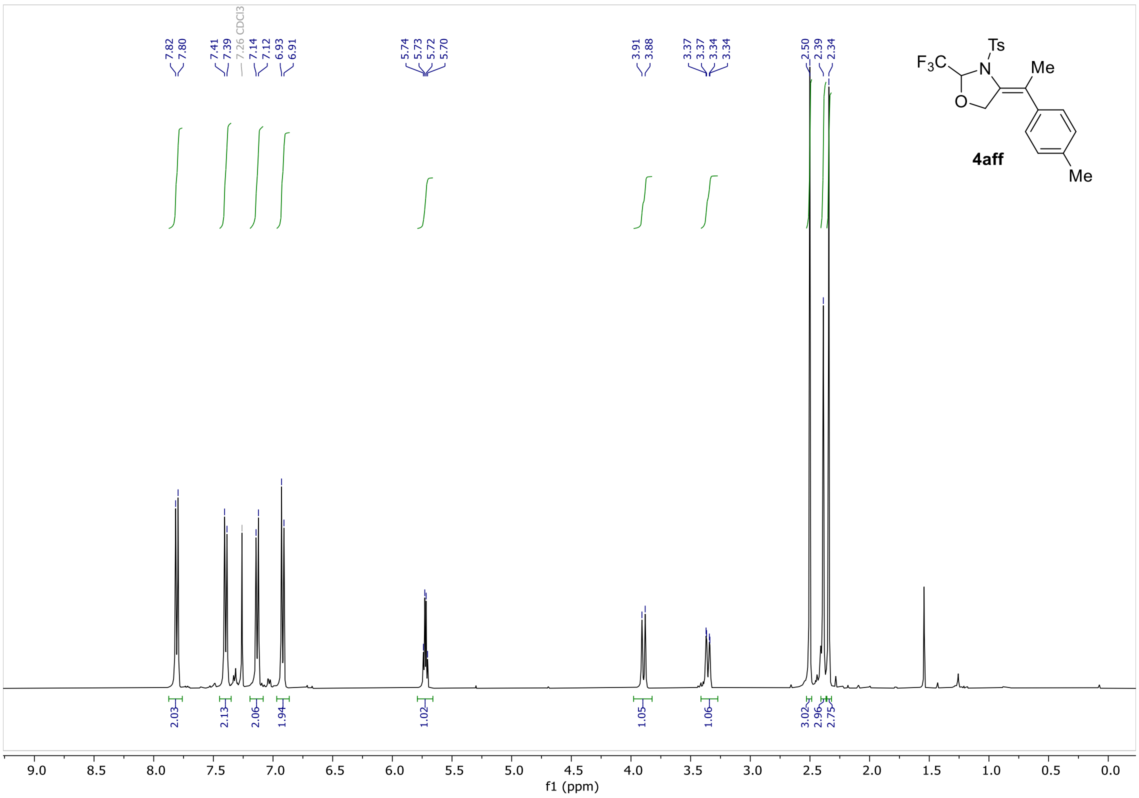

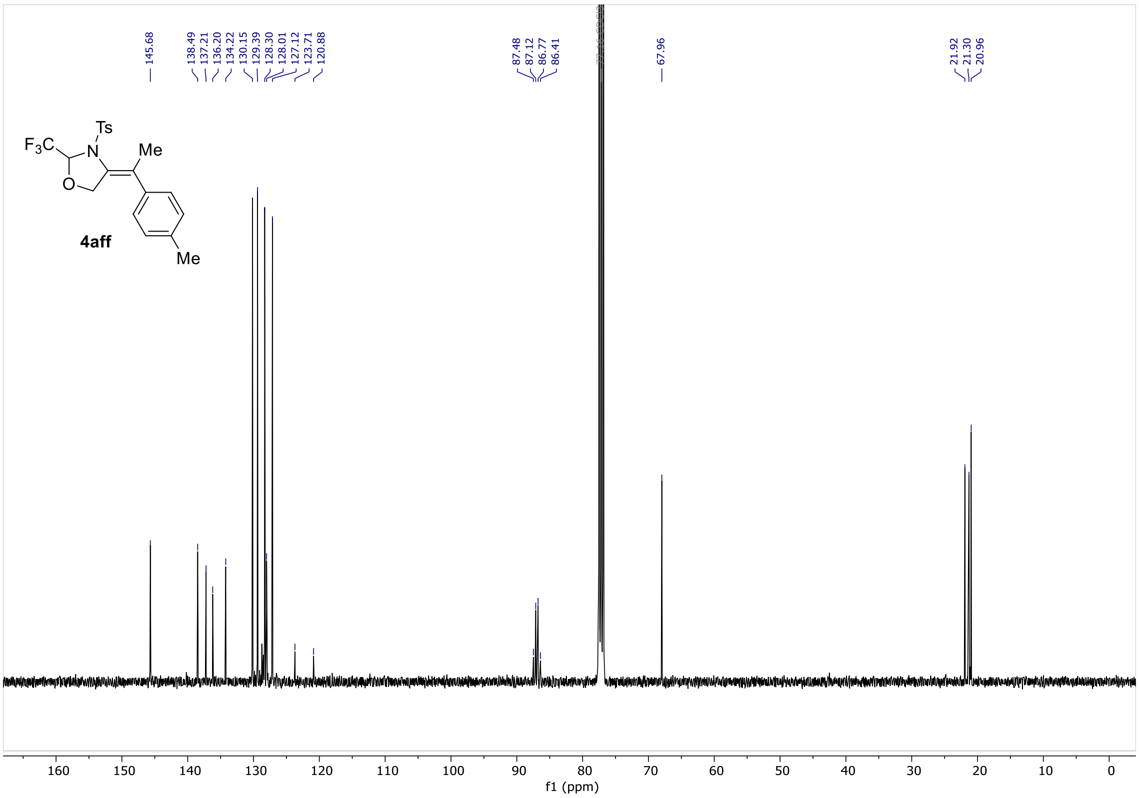
145.68
138.49
137.21
136.20
134.22
130.15
129.39
128.30
128.01
127.12
123.71
120.88
87.48
87.12
86.77
86.41
67.96
21.92
21.30
20.96
Ts
F_3C
N
Me
O
4aff
Me
160 150 140 130 120 110 100 90 80 70 60 50 40 30 20 10 0
f1 (ppm)

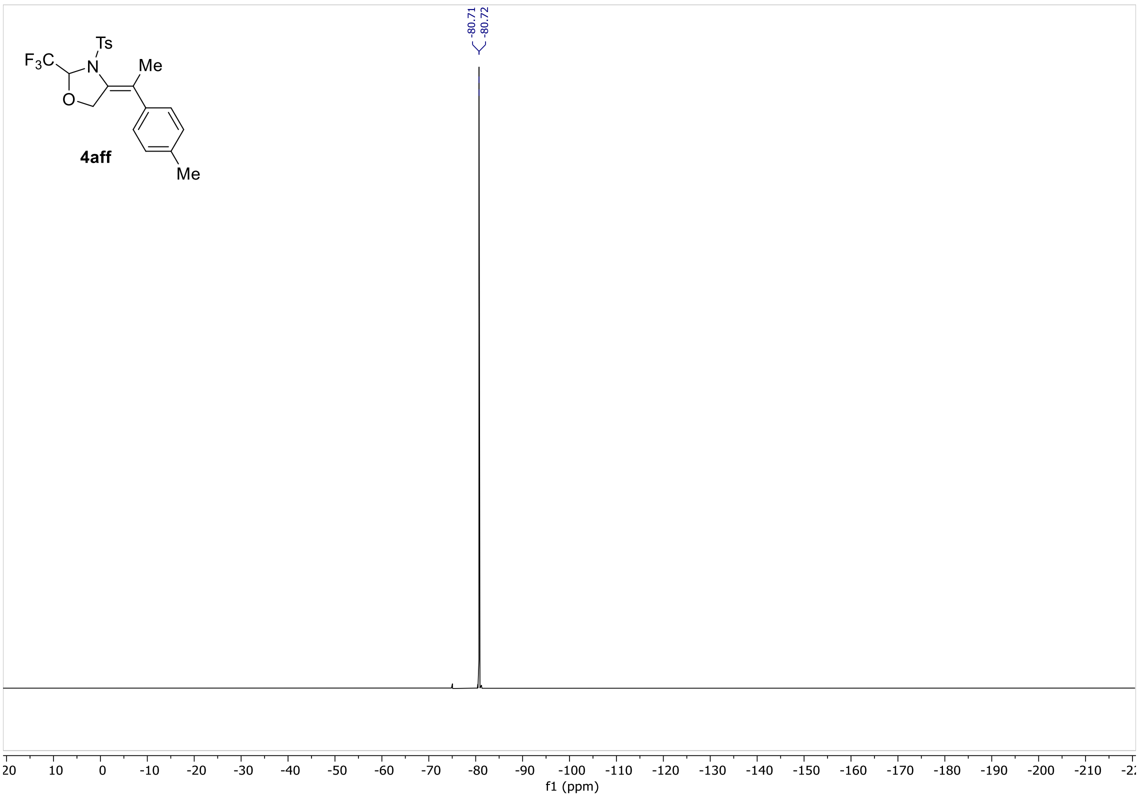
-80.71
-80.72
Ts
F_3C
N
Me
O
4aff
Me
20 10 0 -10 -20 -30 -40 -50 -60 -70 -80 -90 -100 -110 -120 -130 -140 -150 -160 -170 -180 -190 -200 -210
f1 (ppm)

(***E***)**-3-Tosyl-2-(trifluoromethyl)-4-(1-(4-(trifluoromethyl)phenyl)ethylidene)oxazolidine (4ahf)**

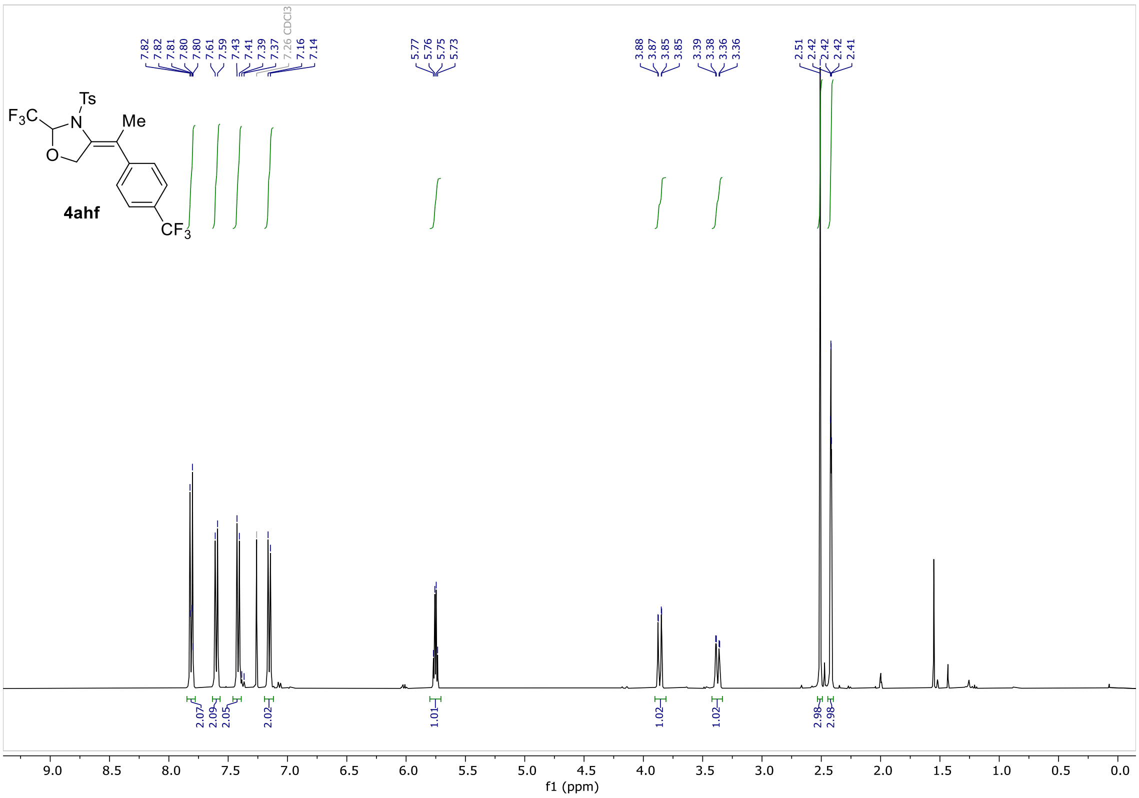

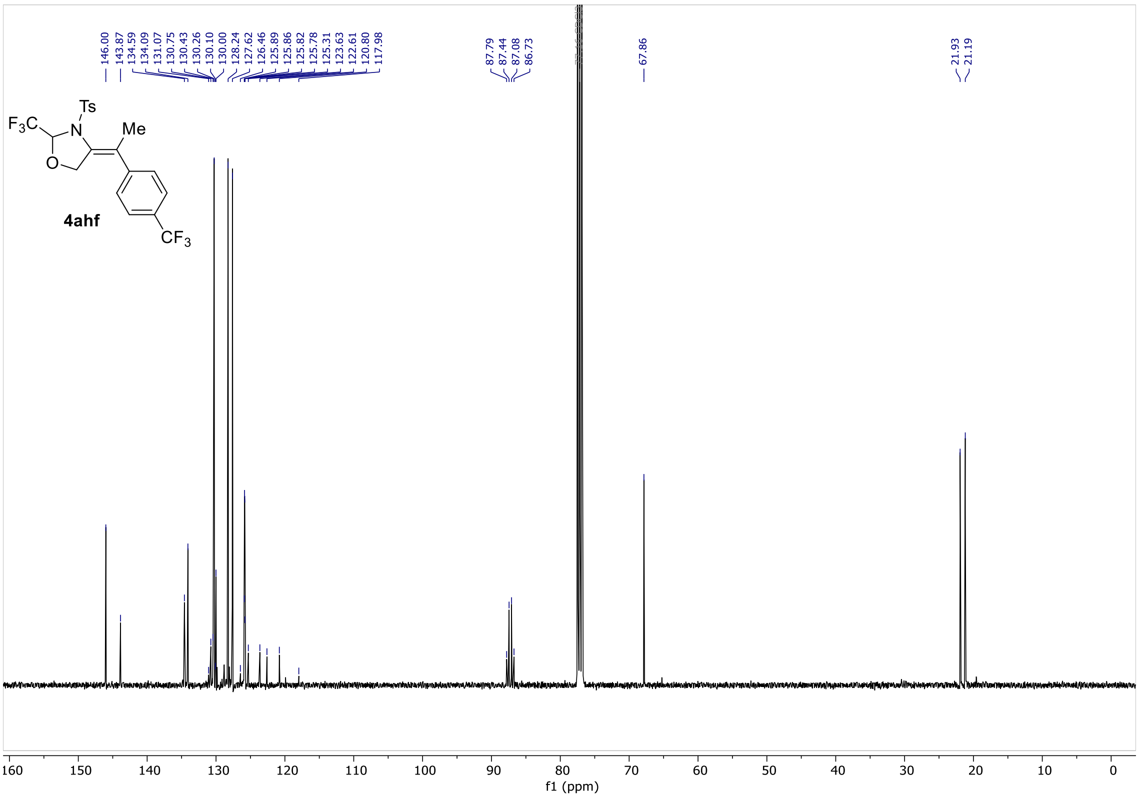

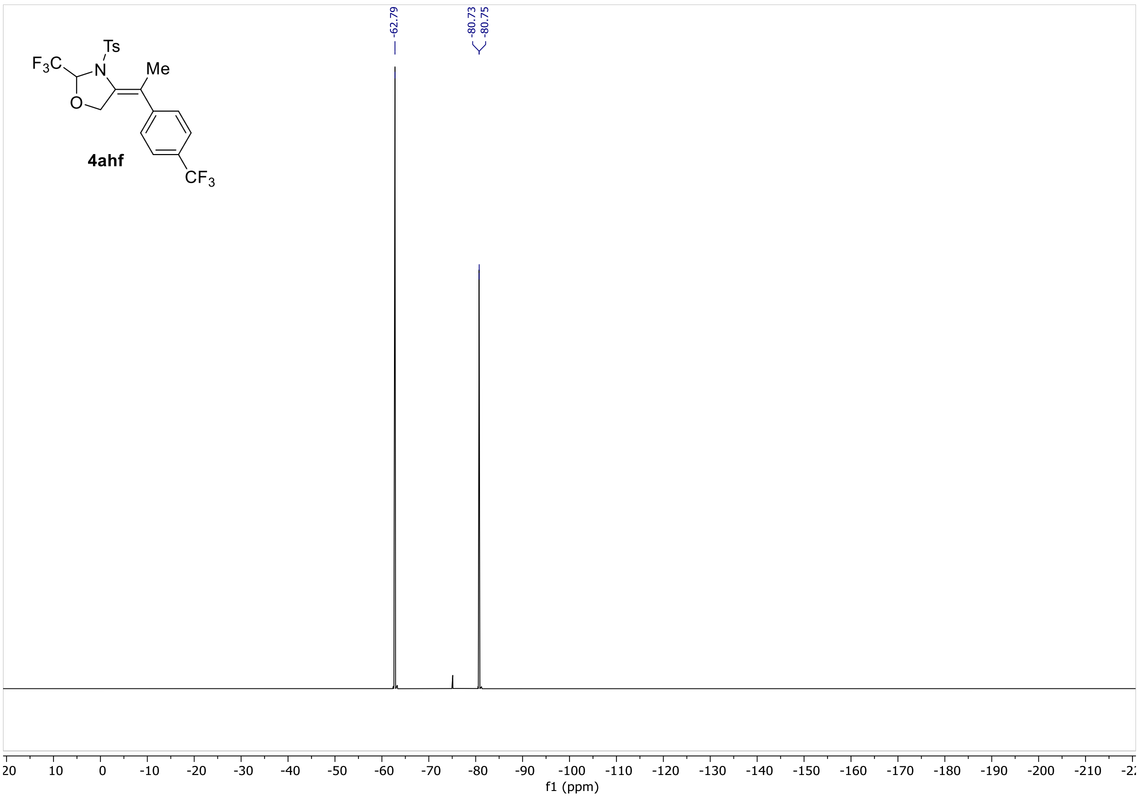

(*E*)-4-(Phenyl(4-(trifluoromethyl)phenyl)methylene)-3-tosyl-2-(trifluoromethyl)oxazolidine (4bhf)

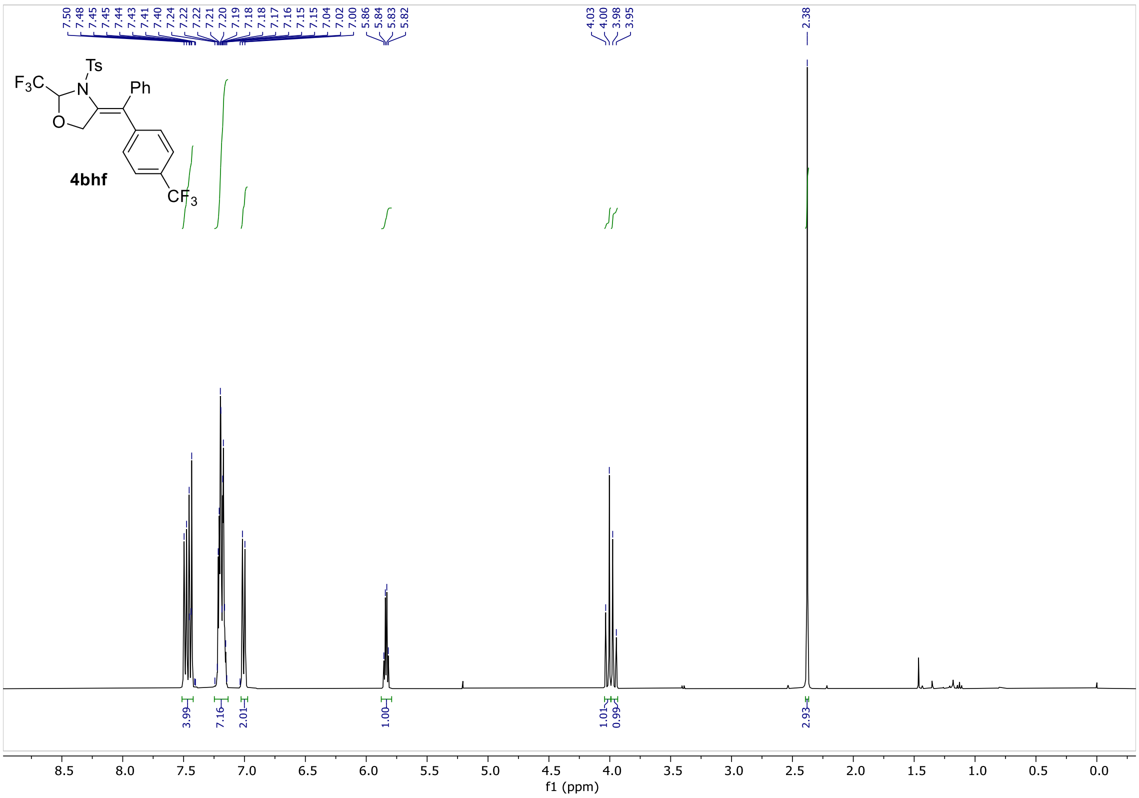

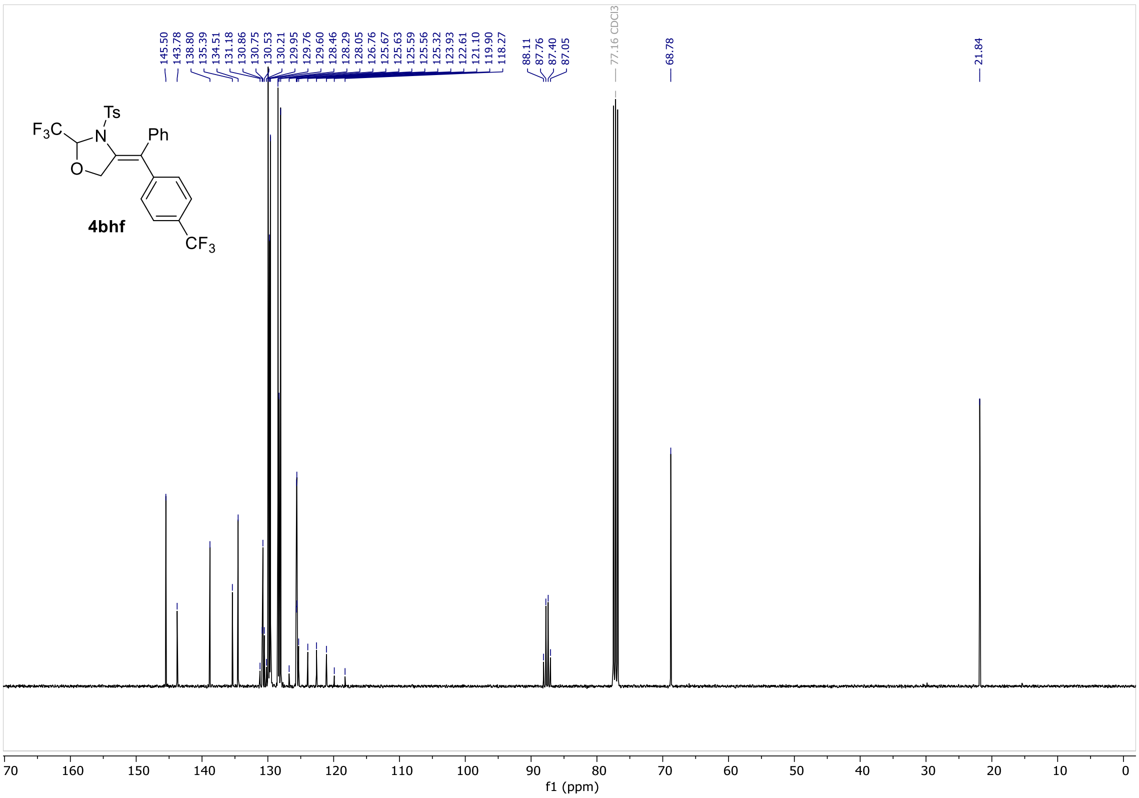
145.50
143.78
138.80
135.39
134.51
131.18
130.86
130.75
130.53
130.21
129.95
129.76
129.60
128.46
128.29
128.05
126.76
125.67
125.63
125.59
125.56
125.32
123.93
122.61
121.10
119.90
118.27
88.11
87.76
87.40
87.05
77.16 CDCl3
68.78
21.84
Ts
F3C
N
Ph
O
4bhf
CF3
f1 (ppm)

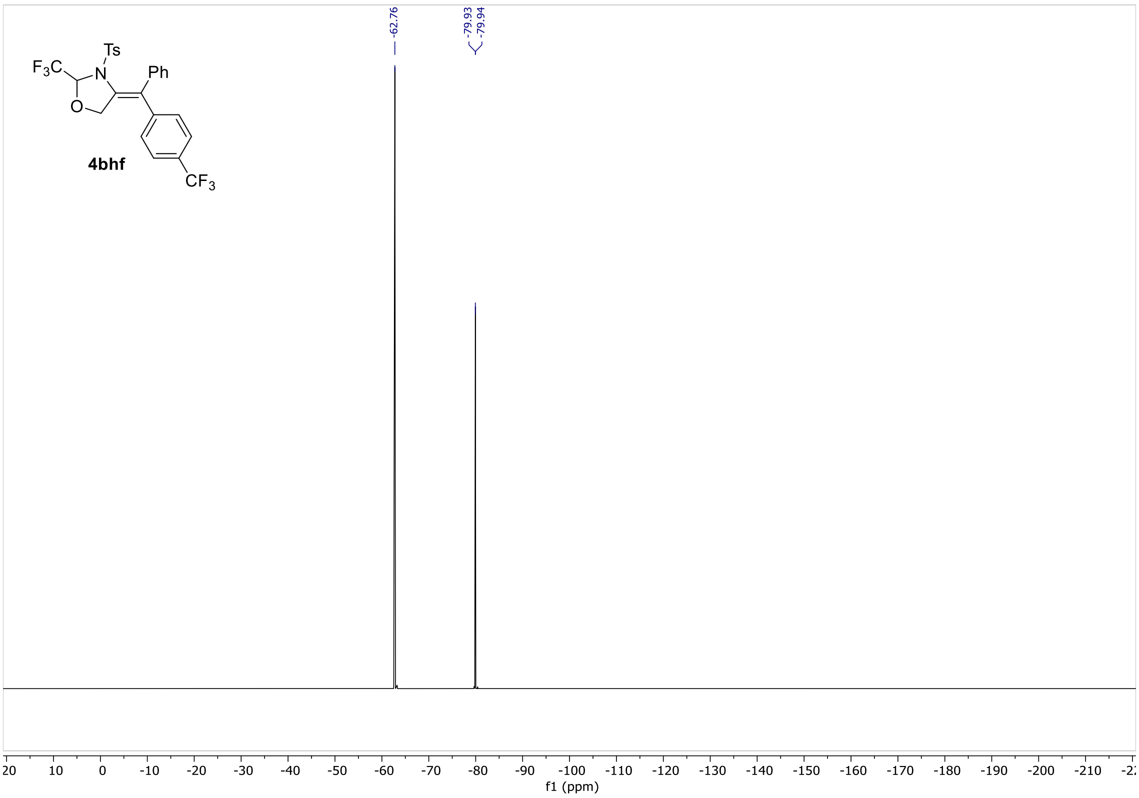
-62.76
-79.93
-79.94
Ts
F3C
N
Ph
O
4bhf
CF3
f1 (ppm)

(*E*)-3-((4-Nitrophenyl)sulfonyl)-4-(phenyl(4-(trifluoromethyl)phenyl)methylene)-2-(trifluoromethyl)oxazolidine (4bhg)

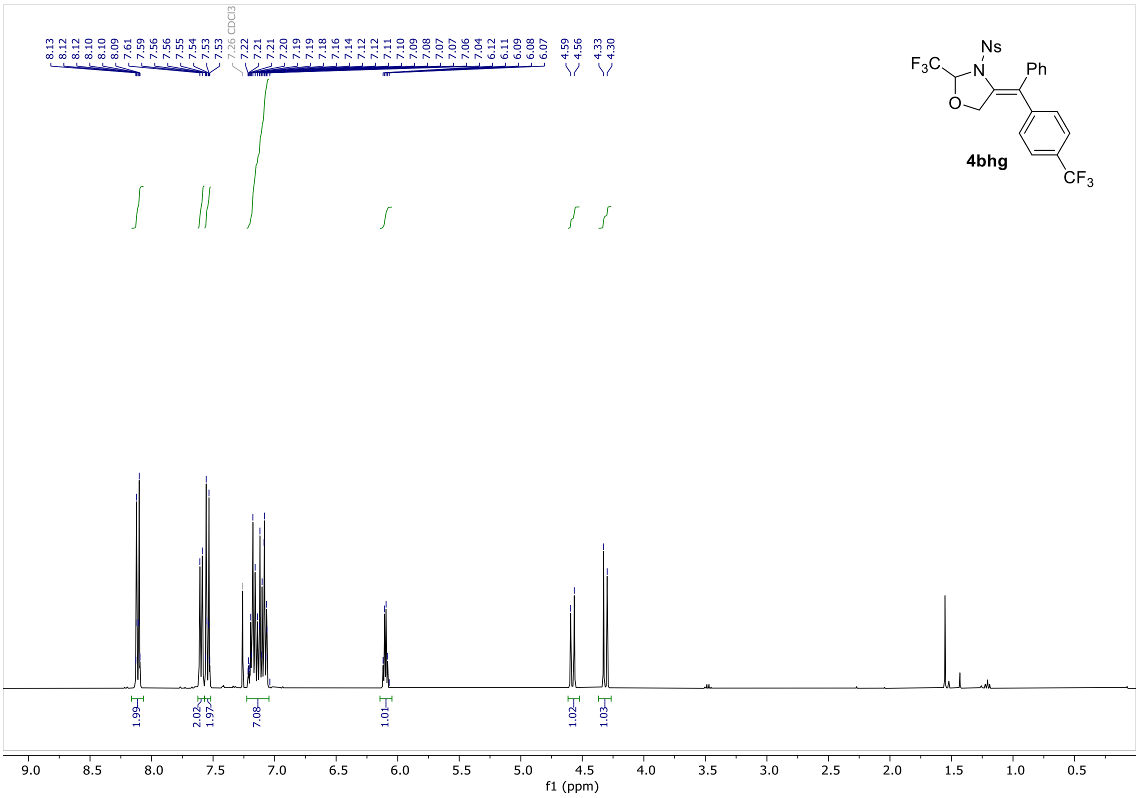

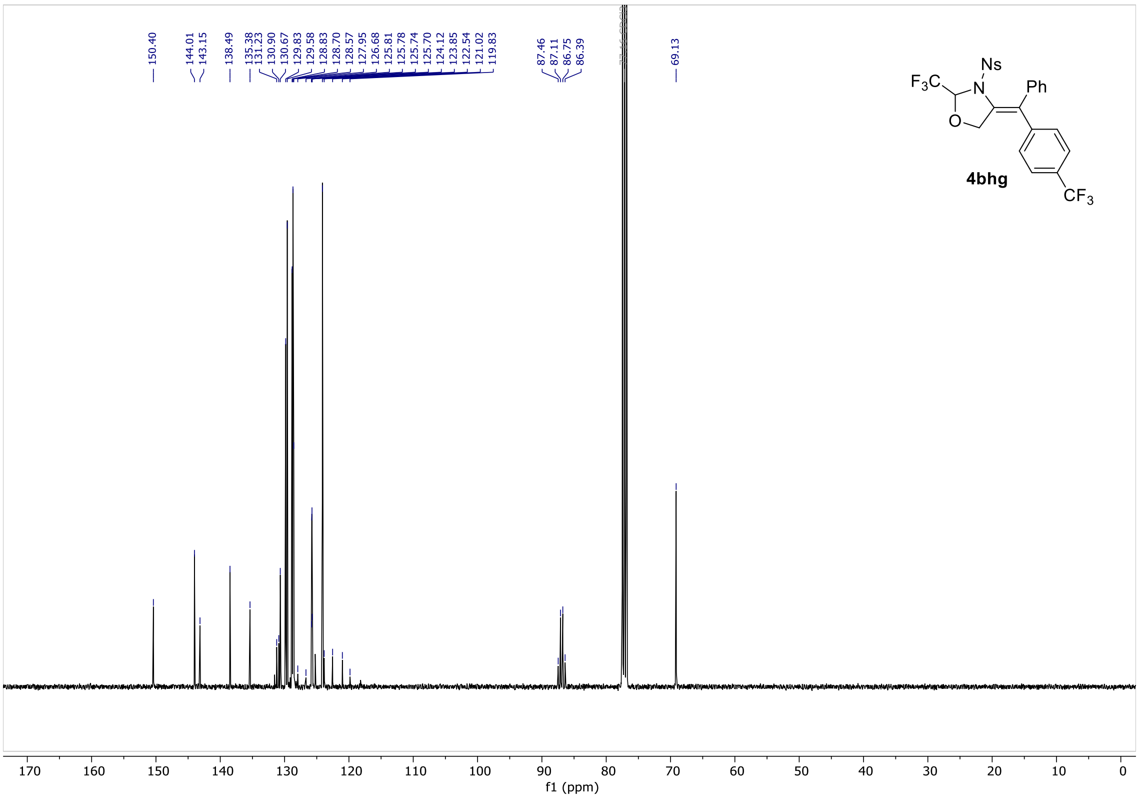

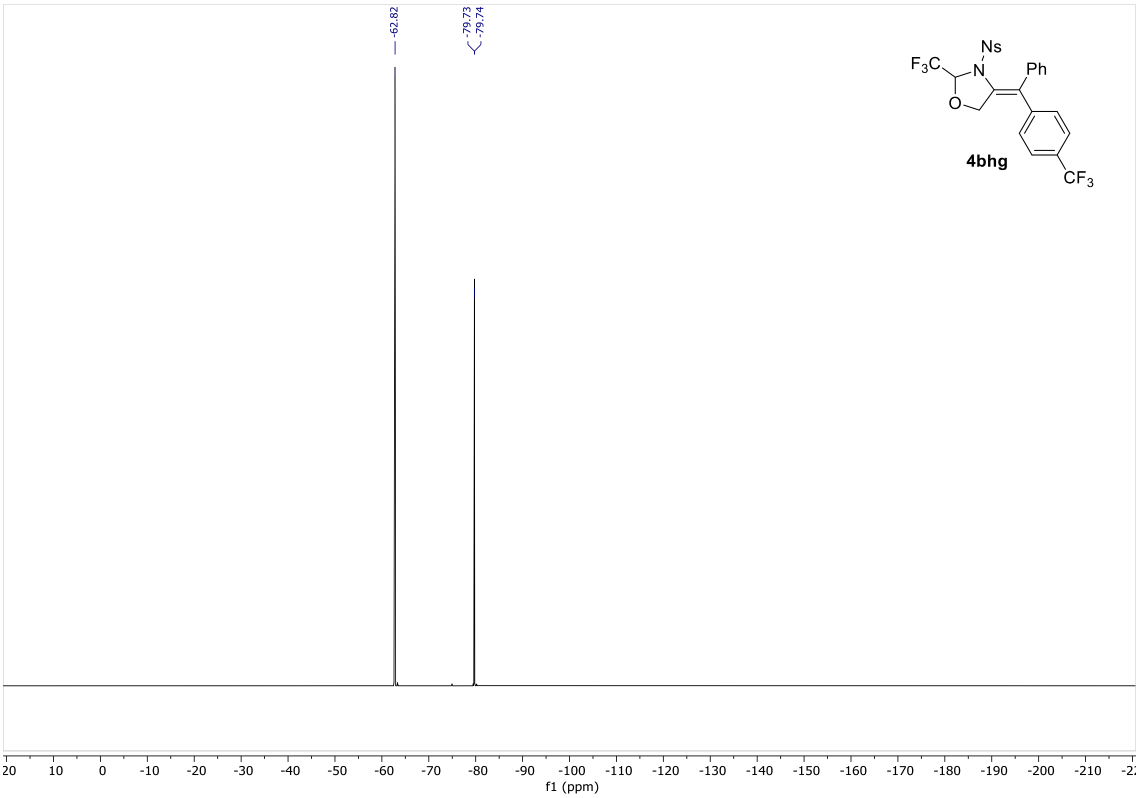

(*E*)-3-((4-Nitrophenyl)sulfonyl)-2-(trifluoromethyl)-4-(1-(4-(trifluoromethyl)phenyl)ethylidene)oxazolidine (4ahg)

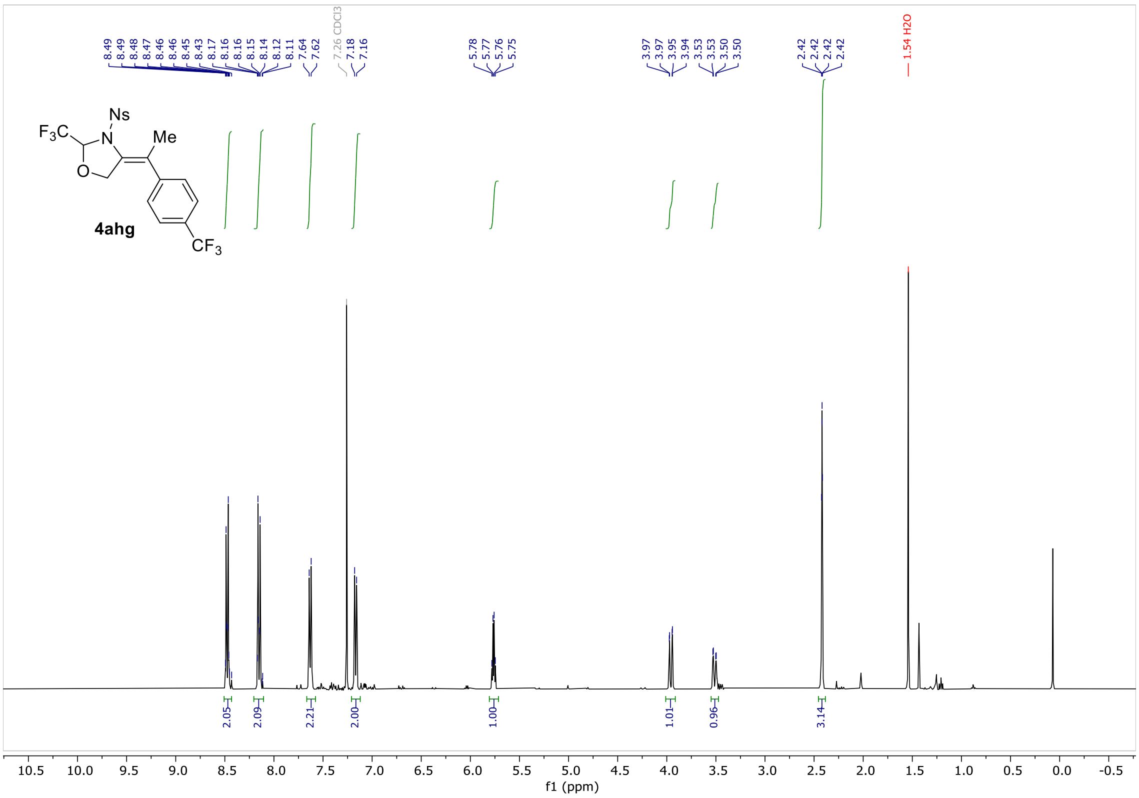

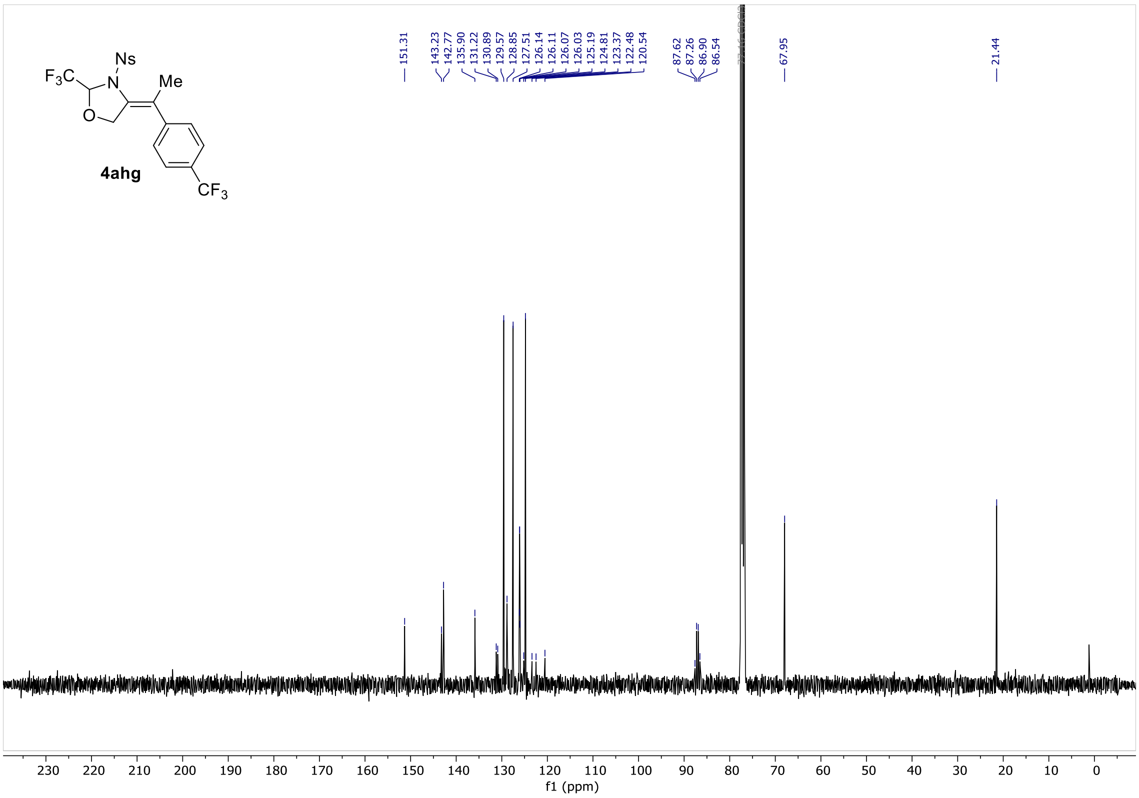
Ns
F3C
N
Me
O
4ahg
CF3
151.31
143.23
142.77
135.90
131.22
130.89
129.57
128.85
127.51
126.14
126.11
126.07
126.03
125.19
124.81
123.37
122.48
120.54
87.62
87.26
86.90
86.54
67.95
21.44
f1 (ppm)

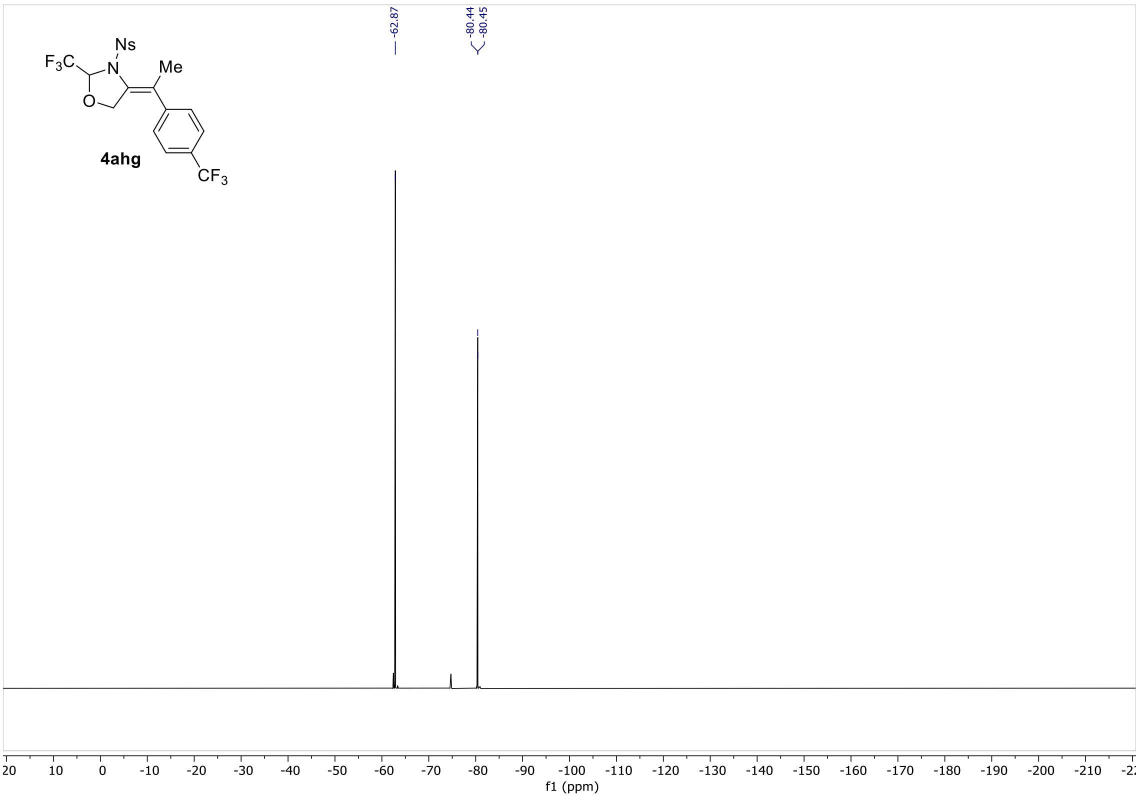
Ns
F3C
N
Me
O
4ahg
CF3
-62.87
-80.44
-80.45
f1 (ppm)

Methyl (*E*)-4-(1-(3-tosyl-2-(trifluoromethyl)oxazolidin-4-ylidene)ethyl)benzoate (4anf)

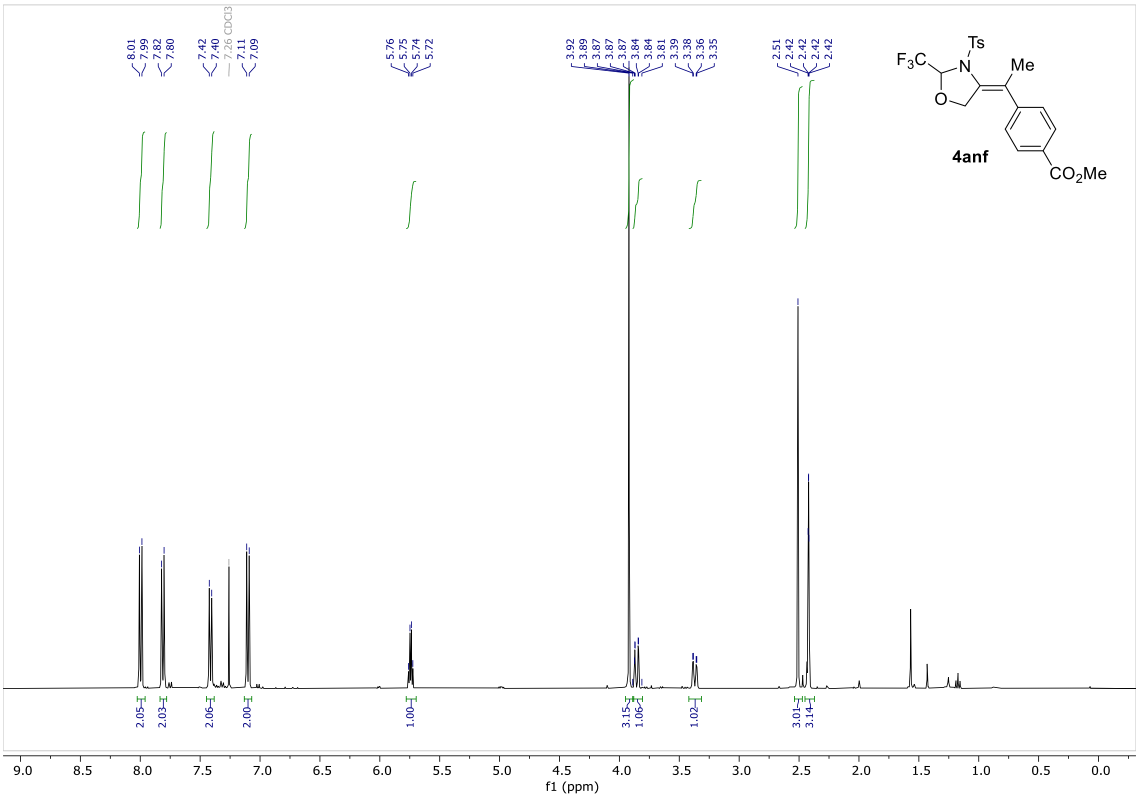

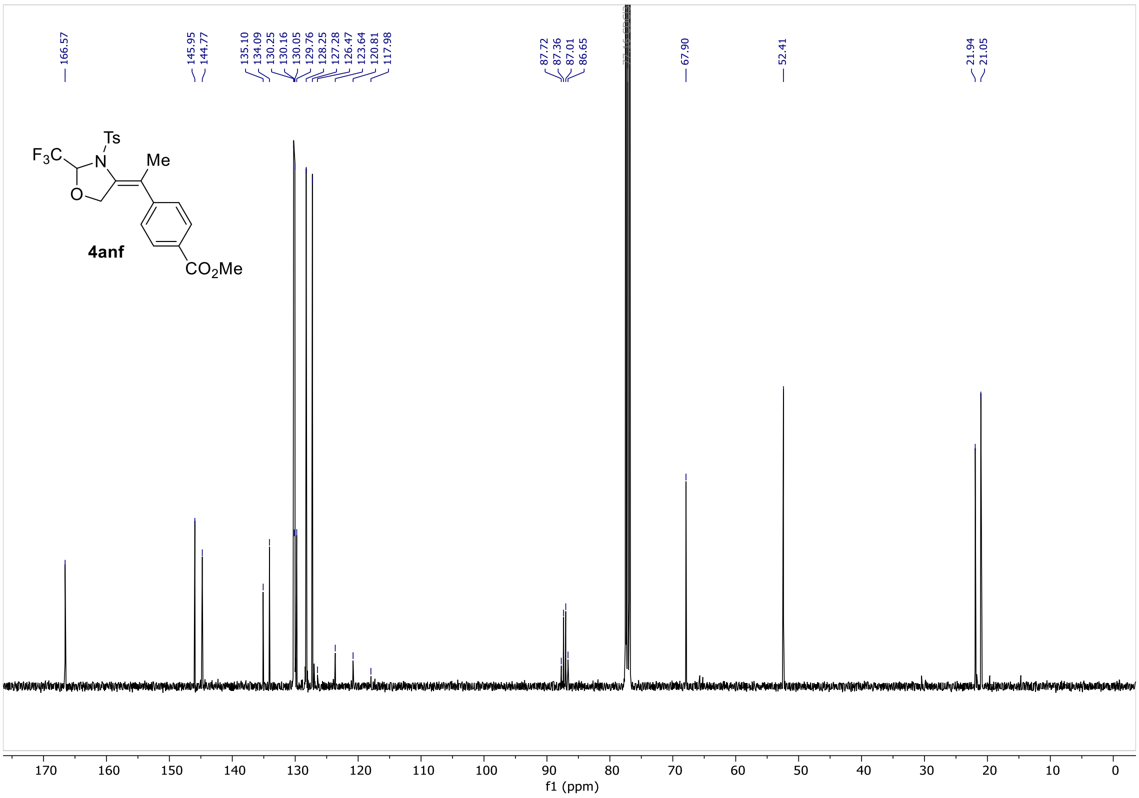

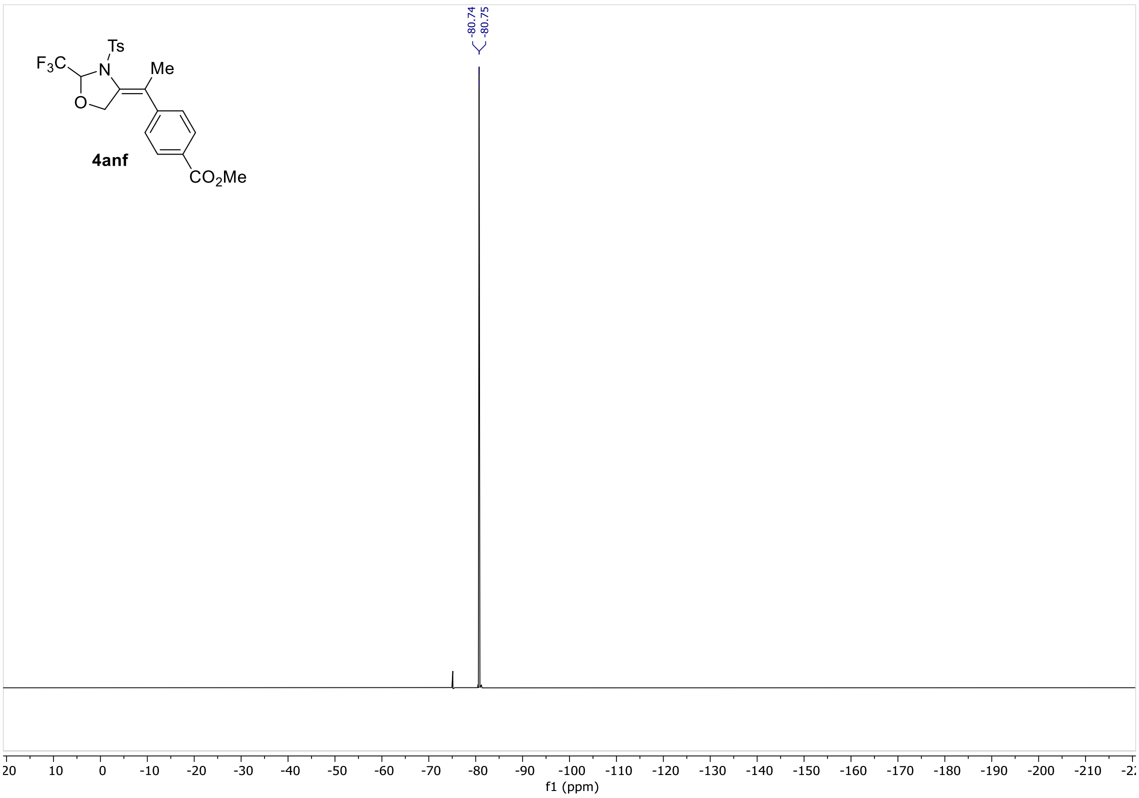

(*E*)-4-(1-(3,5-Bis(trifluoromethyl)phenyl)ethylidene)-3-tosyl-2-(trifluoromethyl)oxazolidine (4aof)

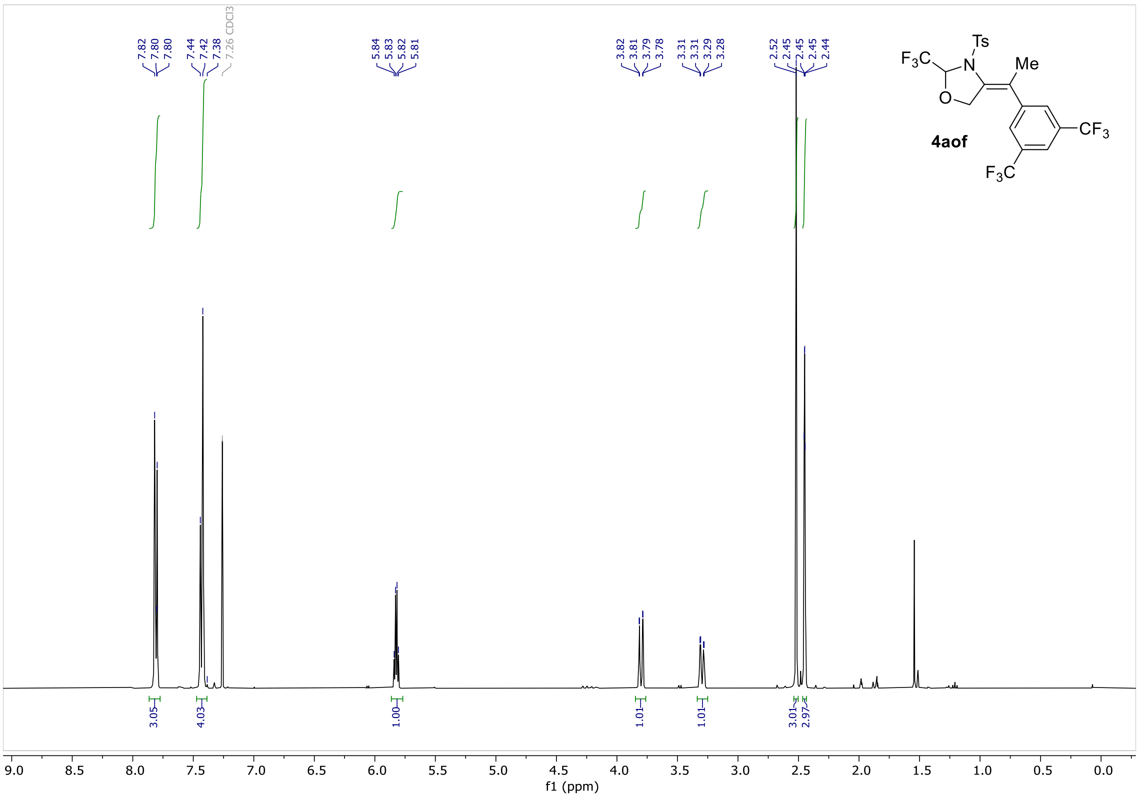

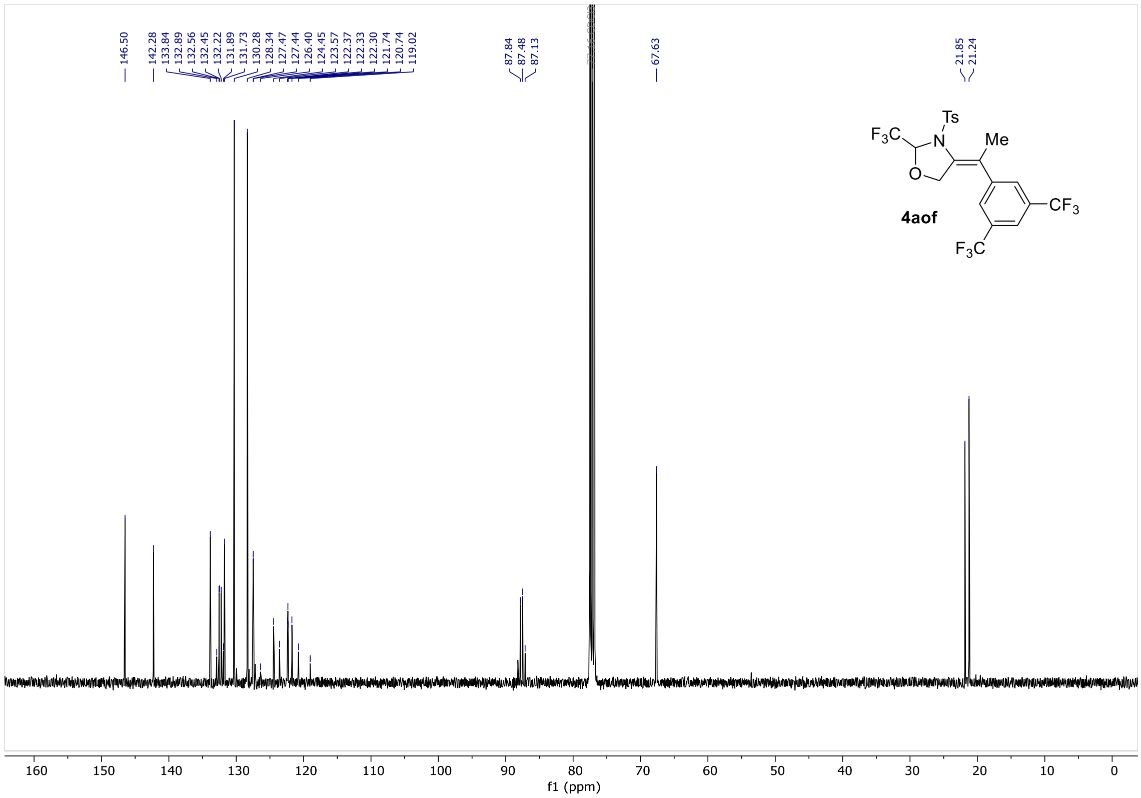
146.50
142.28
133.84
132.89
132.56
132.45
132.22
131.89
131.73
130.28
128.34
127.47
127.44
126.40
124.45
123.57
122.37
122.33
122.30
121.74
120.74
119.02
87.84
87.48
87.13
67.63
21.85
21.24
4aof
f1 (ppm)

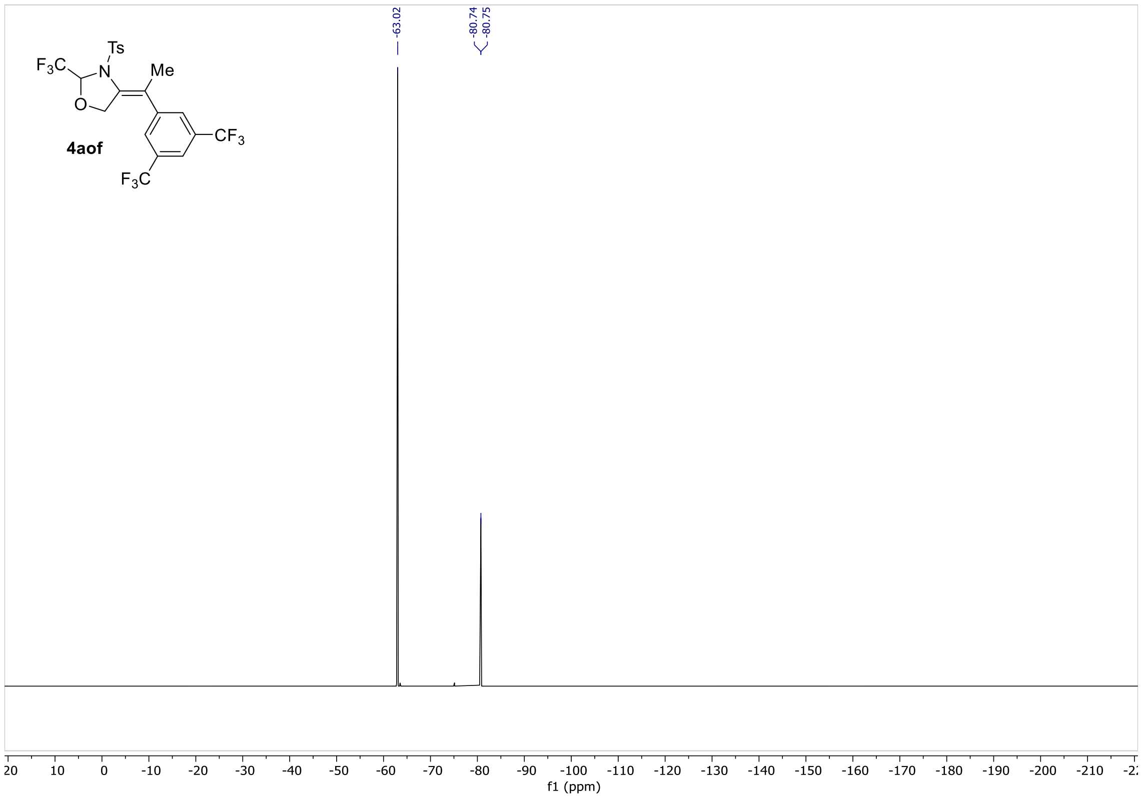
-63.02
-80.74
-80.75
4aof
f1 (ppm)

(*E*)-4-(1-(2-Fluorophenyl)ethylidene)-3-tosyl-2-(trifluoromethyl)oxazolidine (4apf)

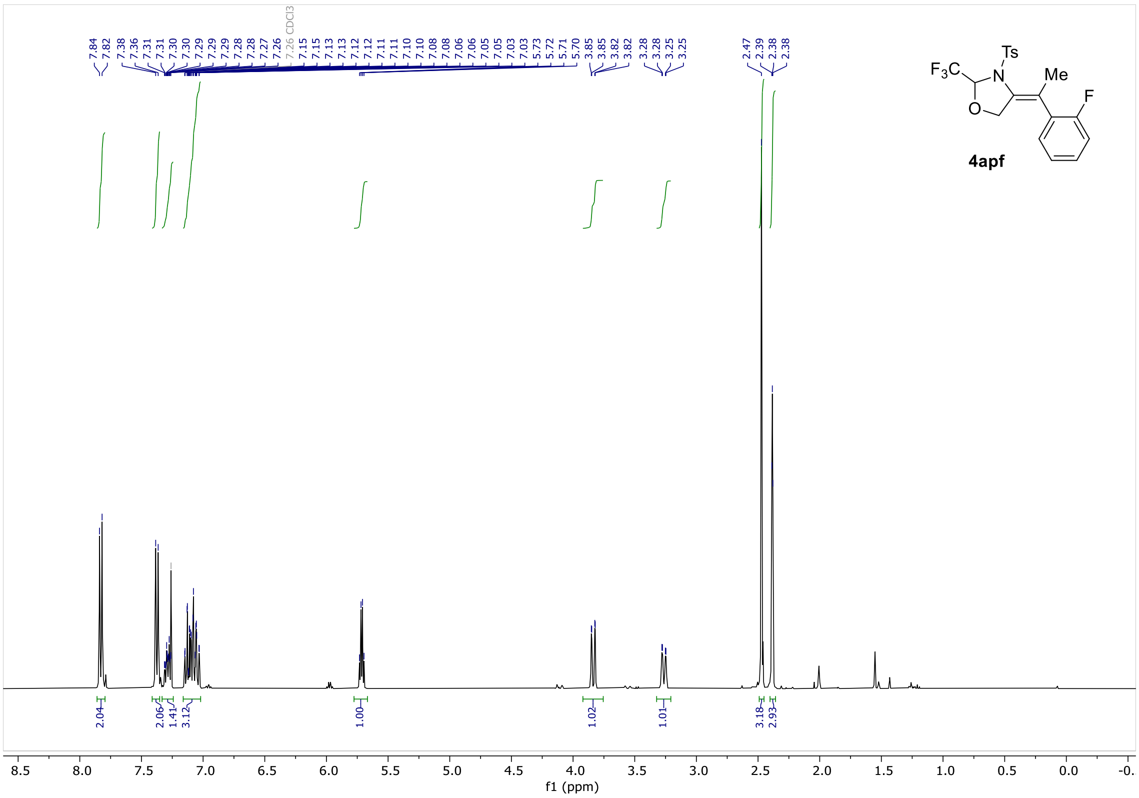

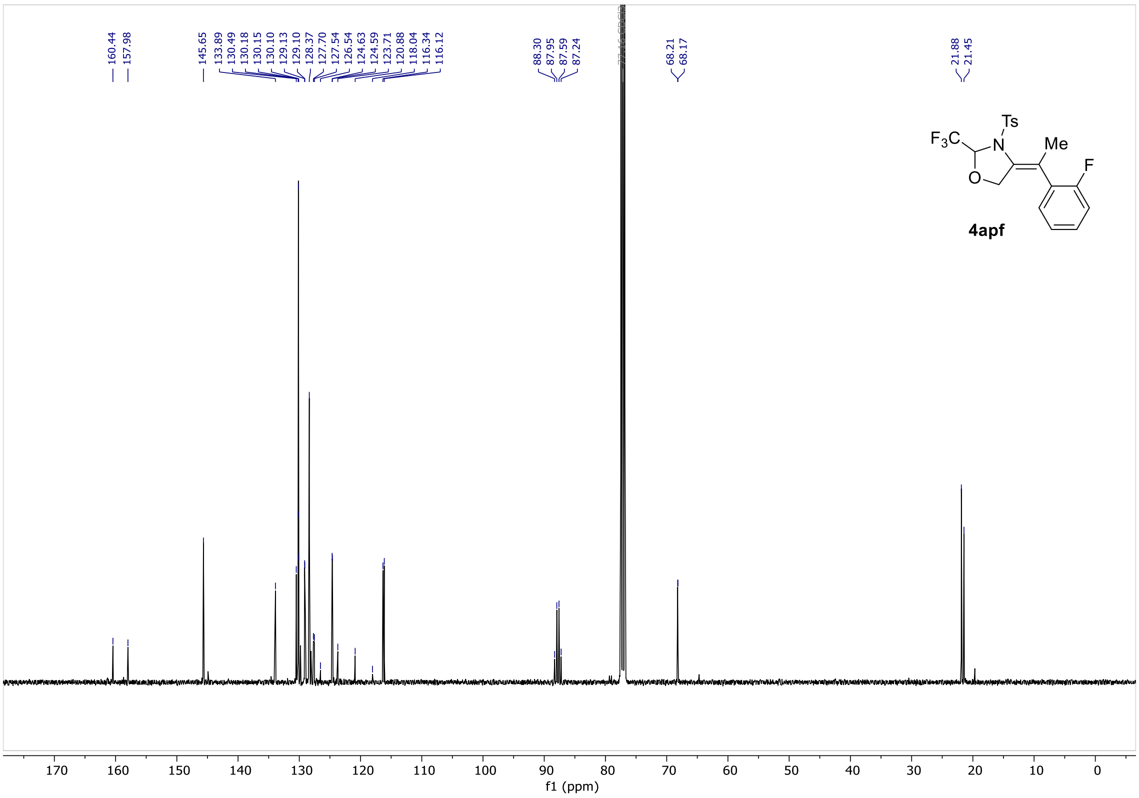

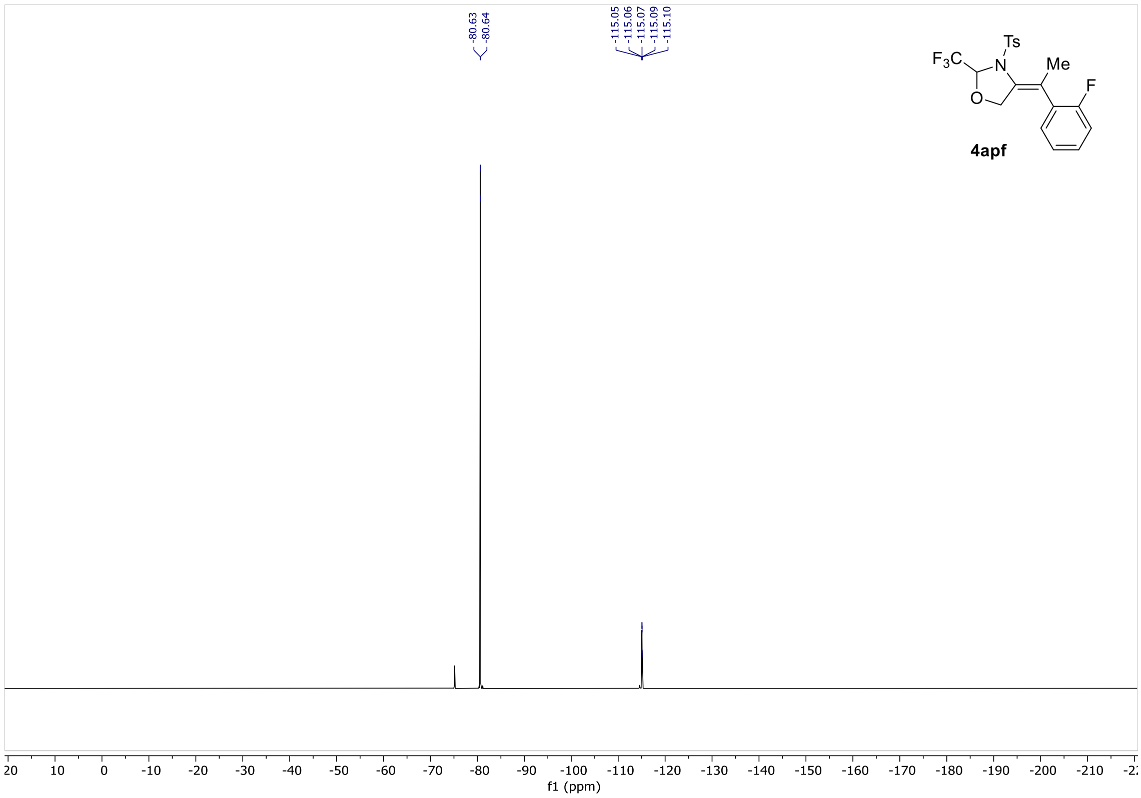
-80.63
-80.64
-115.05
-115.06
-115.07
-115.09
-115.10
F3C
N
Ts
Me
O
F
4apf
f1 (ppm)

I.3. Product modifications

3-(4-Methoxyphenyl)-4-methyl-5-(p-tolyl)-2-(trifluoromethyl)-1,3-oxazinane (6af)

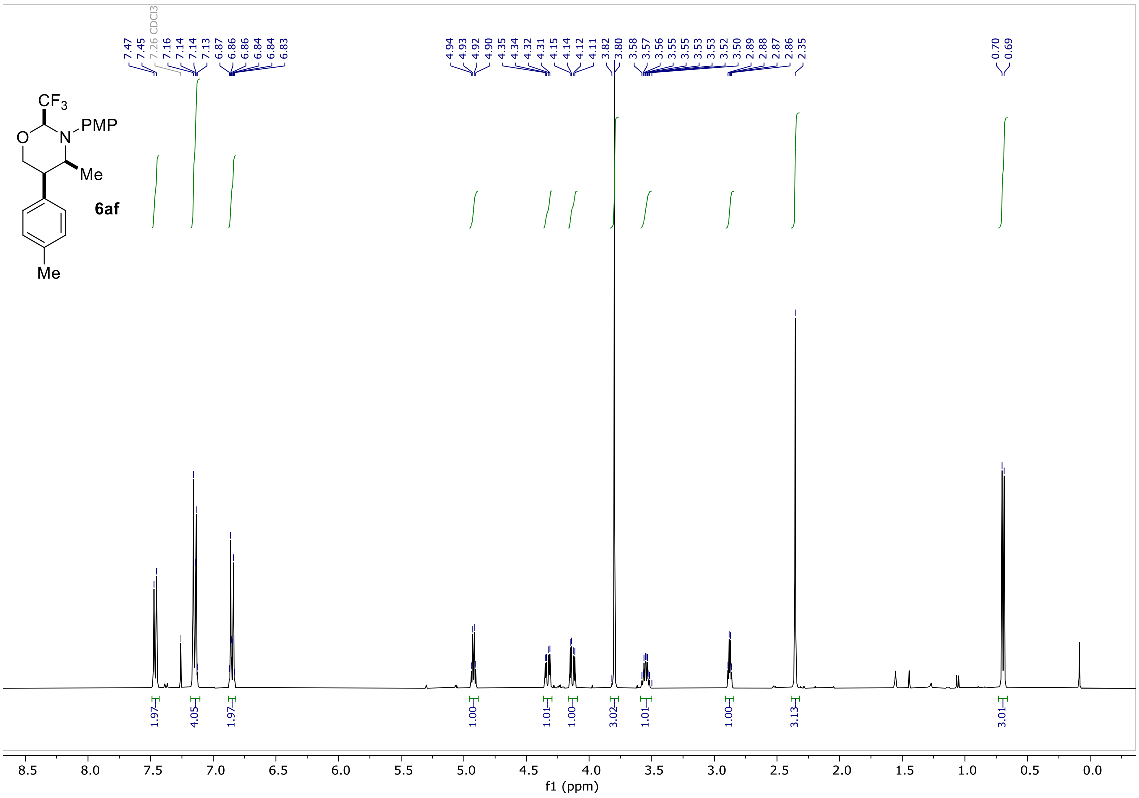

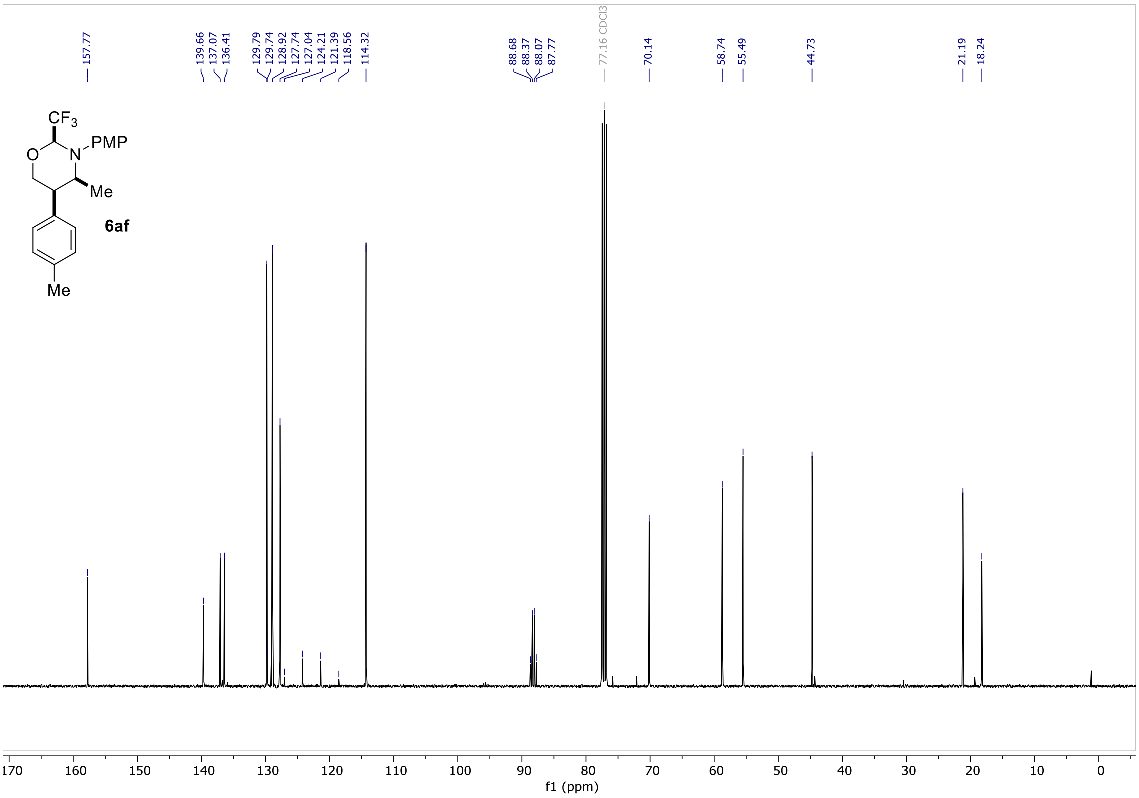

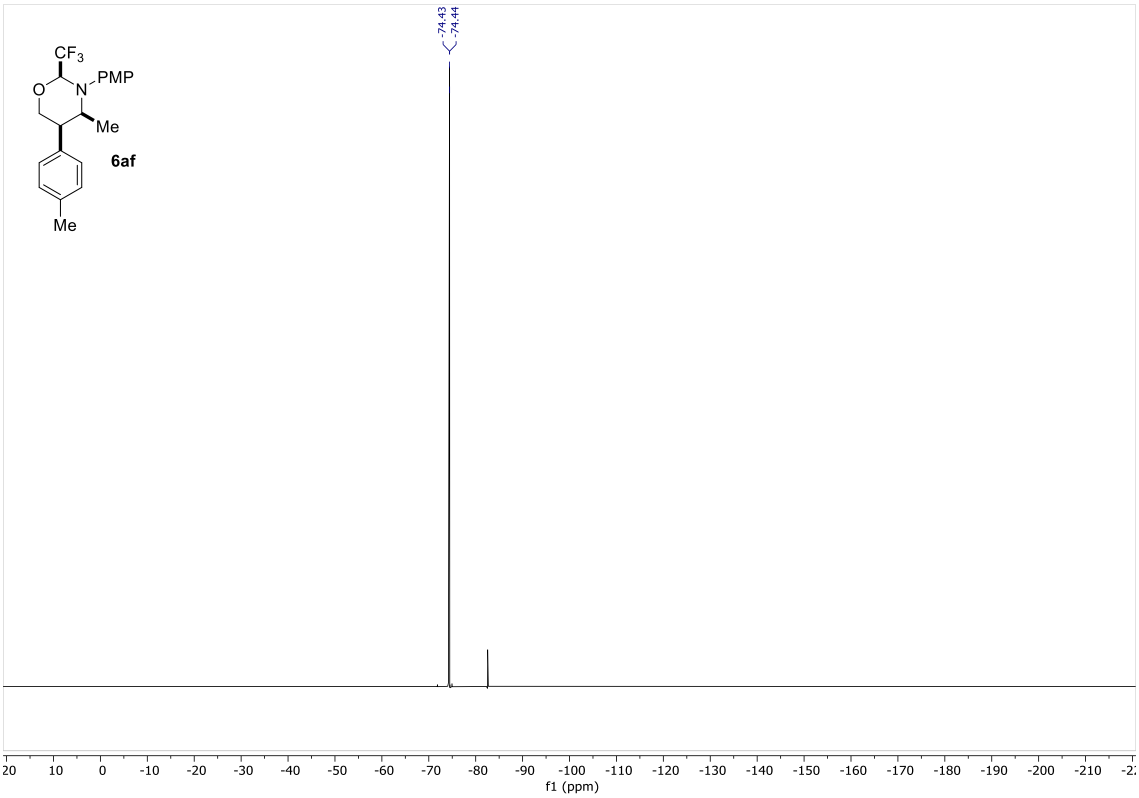

3-(4-Methoxyphenyl)-4-methyl-2-(trifluoromethyl)-5-(4-(trifluoromethyl)phenyl)-1,3-oxazinane (6ah)

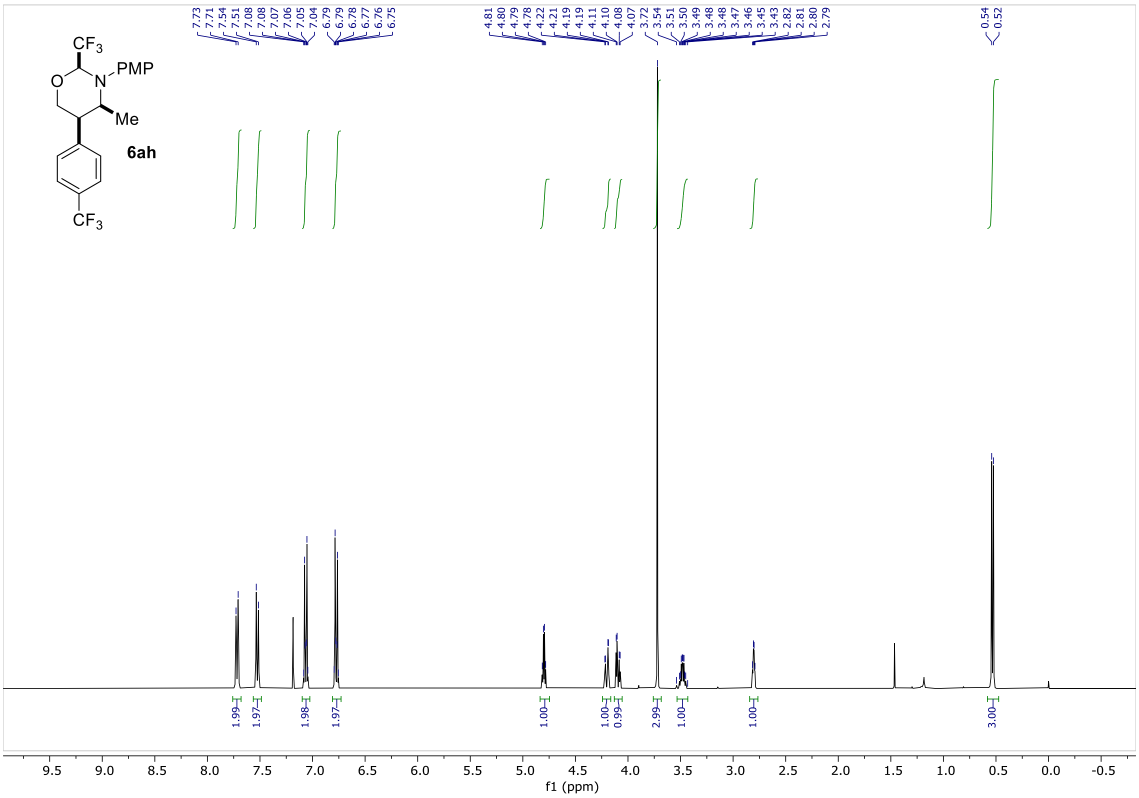

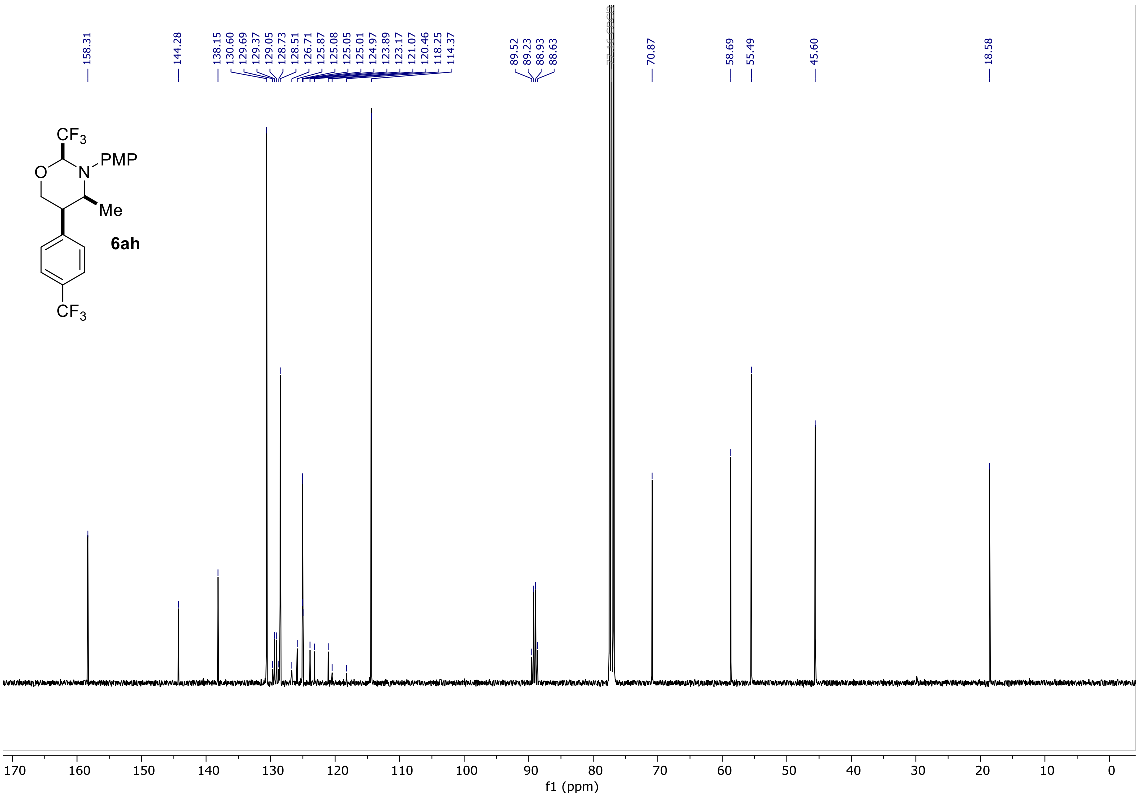
158.31
144.28
138.15
130.60
129.69
129.37
129.05
128.73
128.51
126.71
125.87
125.08
125.05
125.01
124.97
123.89
123.17
121.07
120.46
118.25
114.37
89.52
89.23
88.93
88.63
70.87
58.69
55.49
45.60
18.58
CF3
O
N
PMP
Me
6ah
CF3
f1 (ppm)

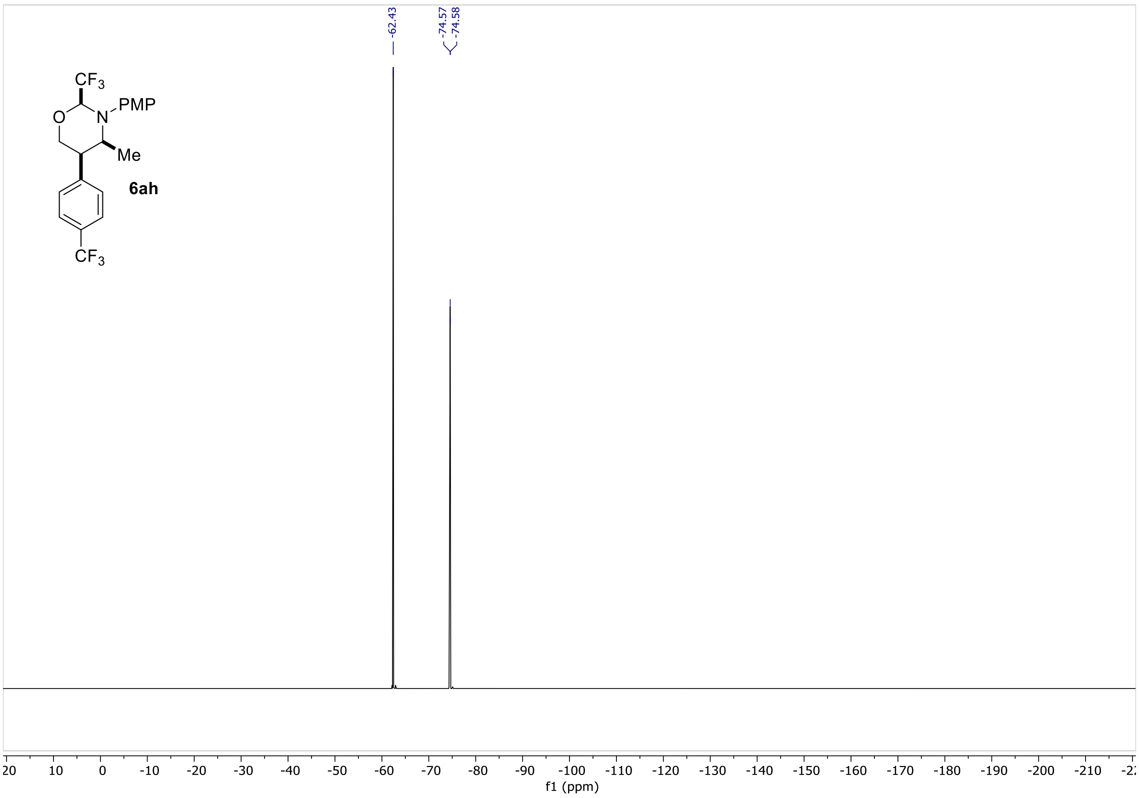
-62.43
-74.57
-74.58
CF3
O
N
PMP
Me
6ah
CF3
f1 (ppm)

4-(1-(*p*-Tolyl)ethyl)-3-tosyl-2-(trifluoromethyl)oxazolidine (13)

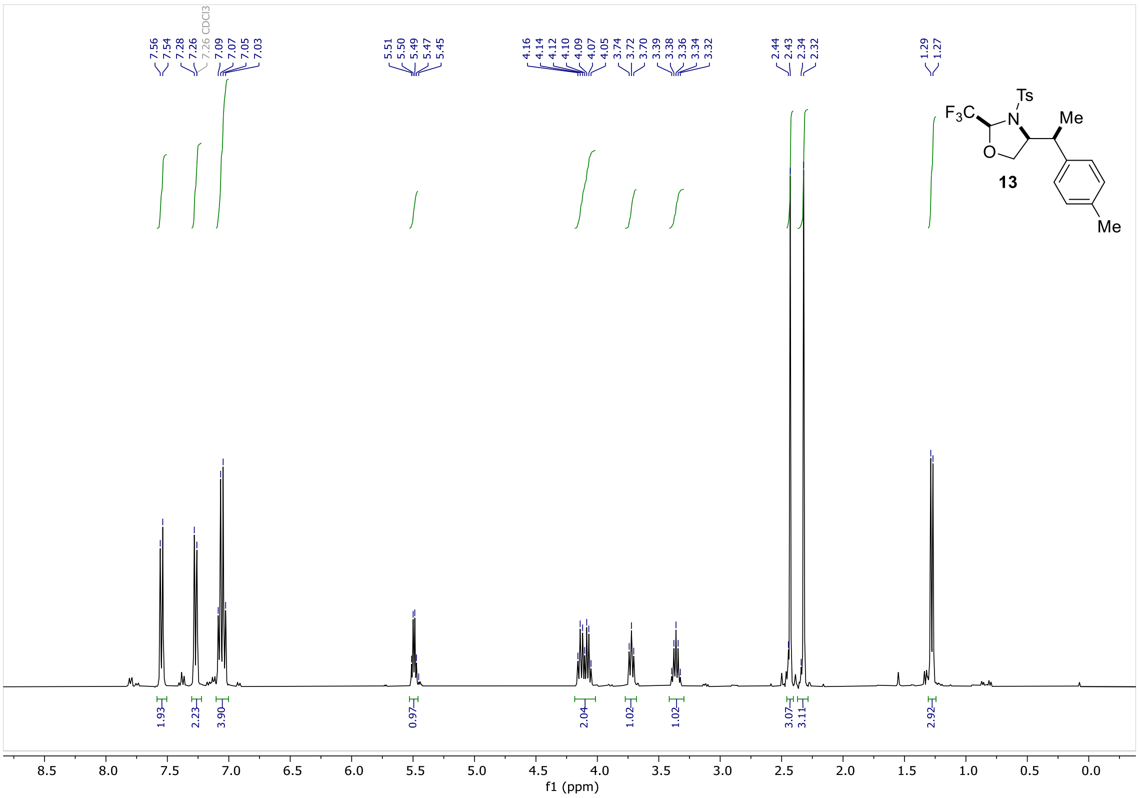

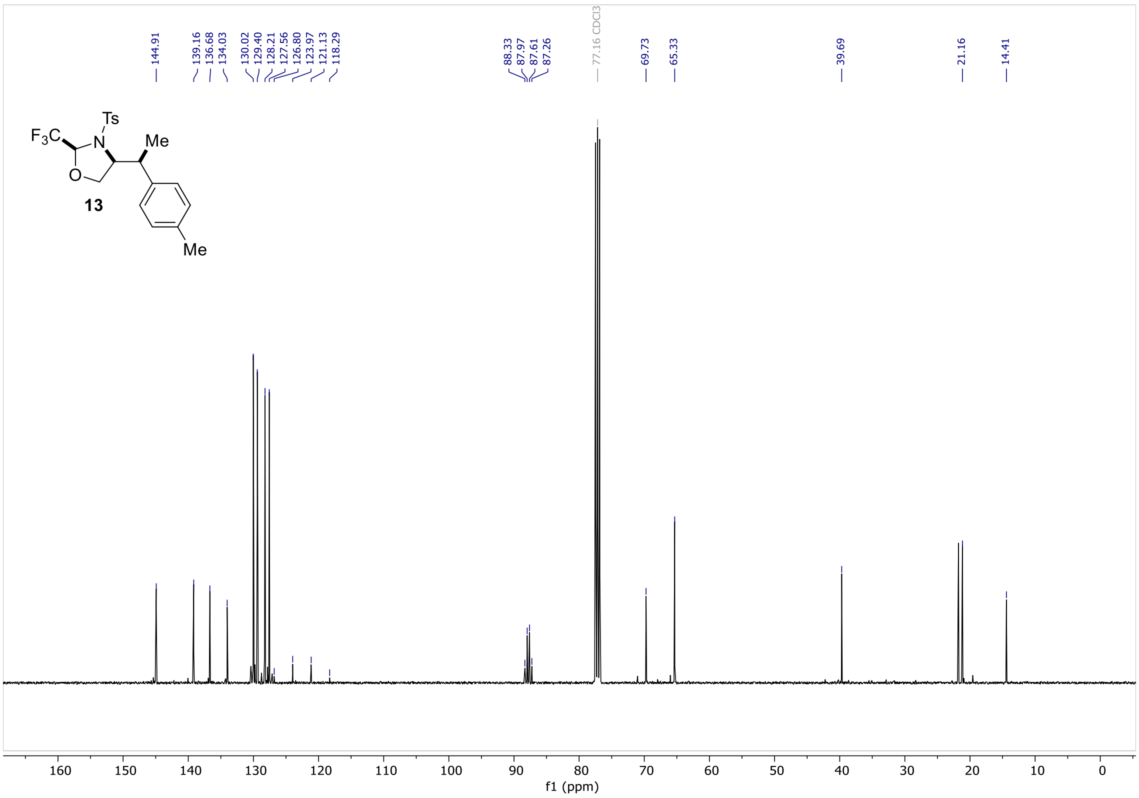

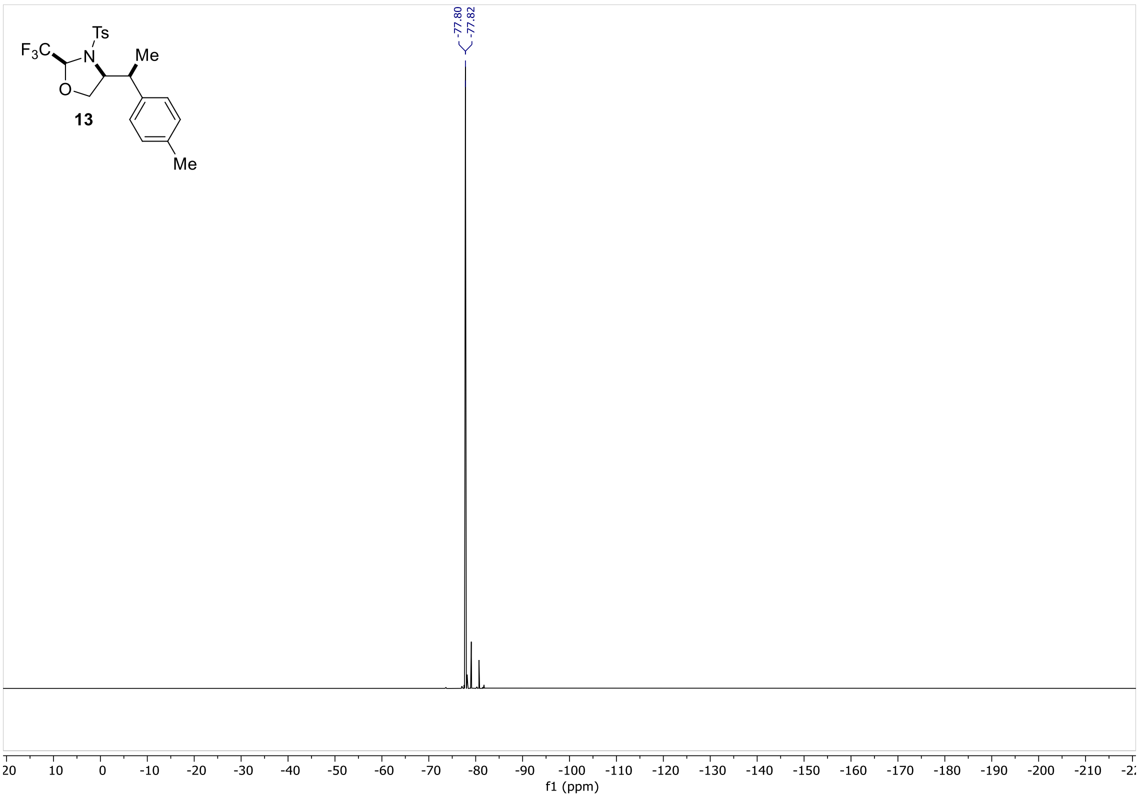

4-(1-(*p*-Tolyl)ethyl)-2-(trifluoromethyl)oxazolidine (14)

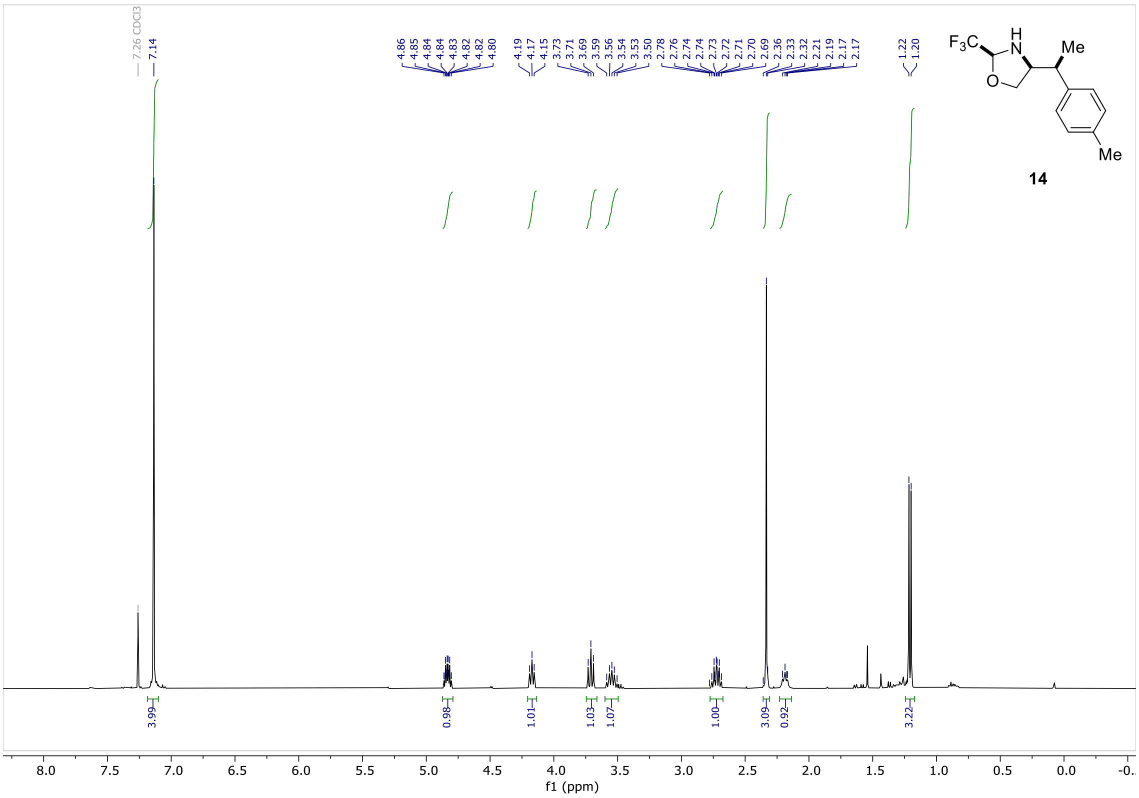

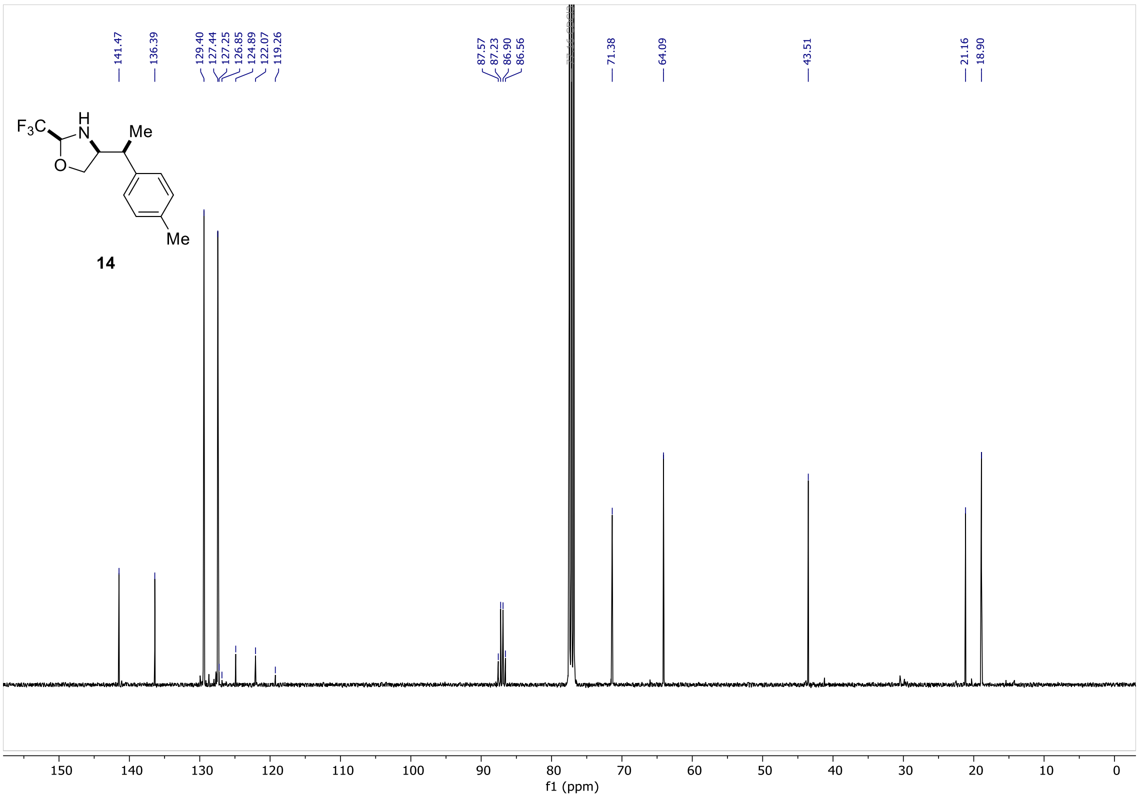
141.47
136.39
129.40
127.44
127.25
126.85
124.89
122.07
119.26
87.57
87.23
86.90
86.56
71.38
64.09
43.51
21.16
18.90
F3C
H
N
Me
O
Me
14
f1 (ppm)

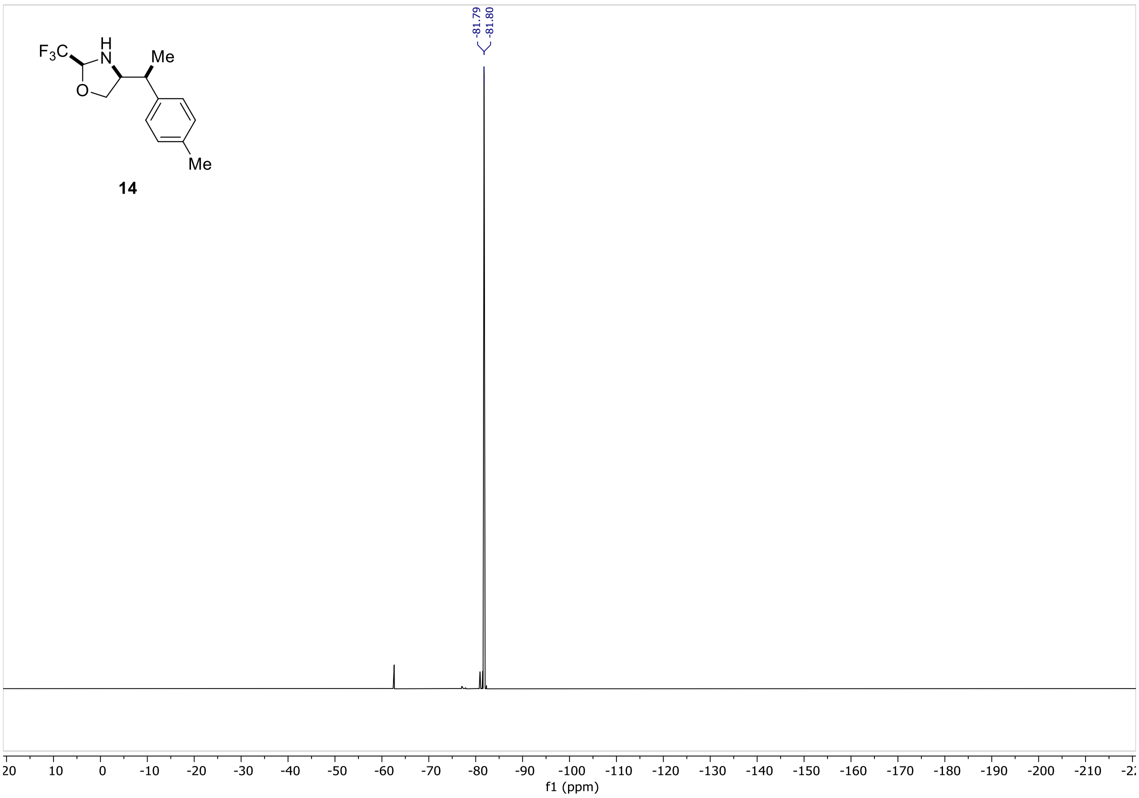
-81.79
-81.80
F3C
H
N
Me
O
Me
14
f1 (ppm)

3-((4-Methoxyphenyl)amino)-2-(p-tolyl)butan-1-ol (7af)

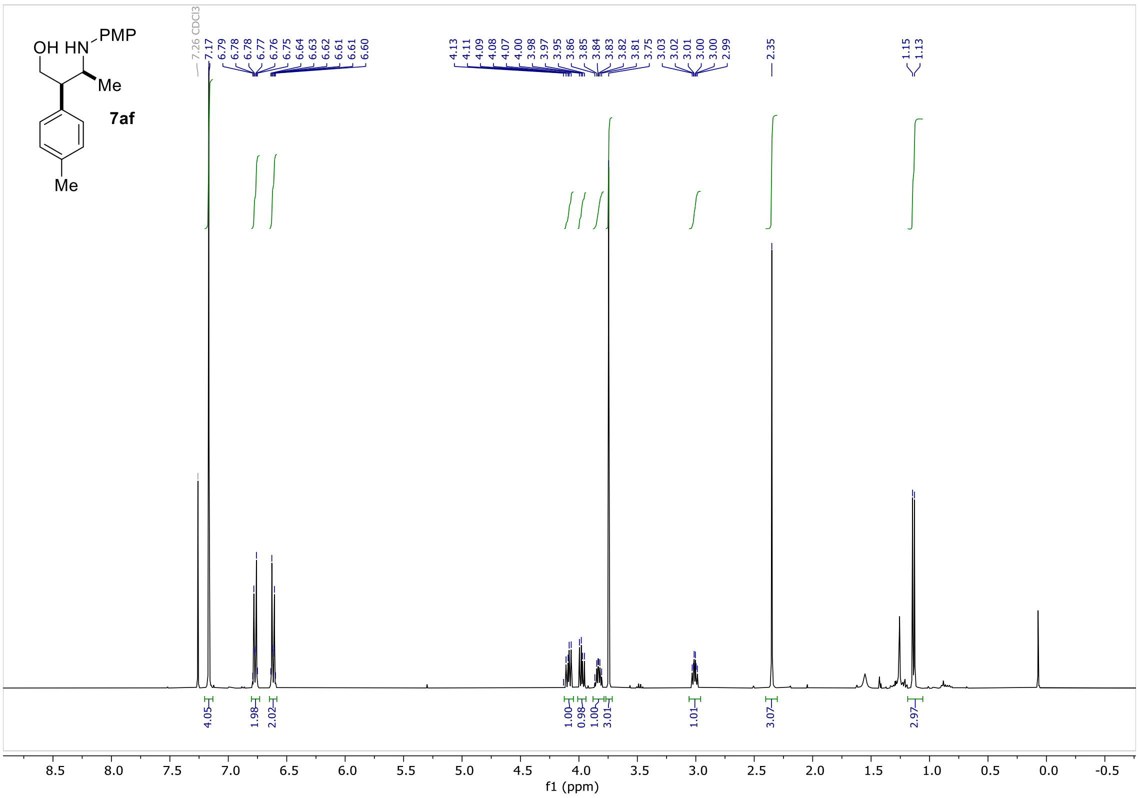

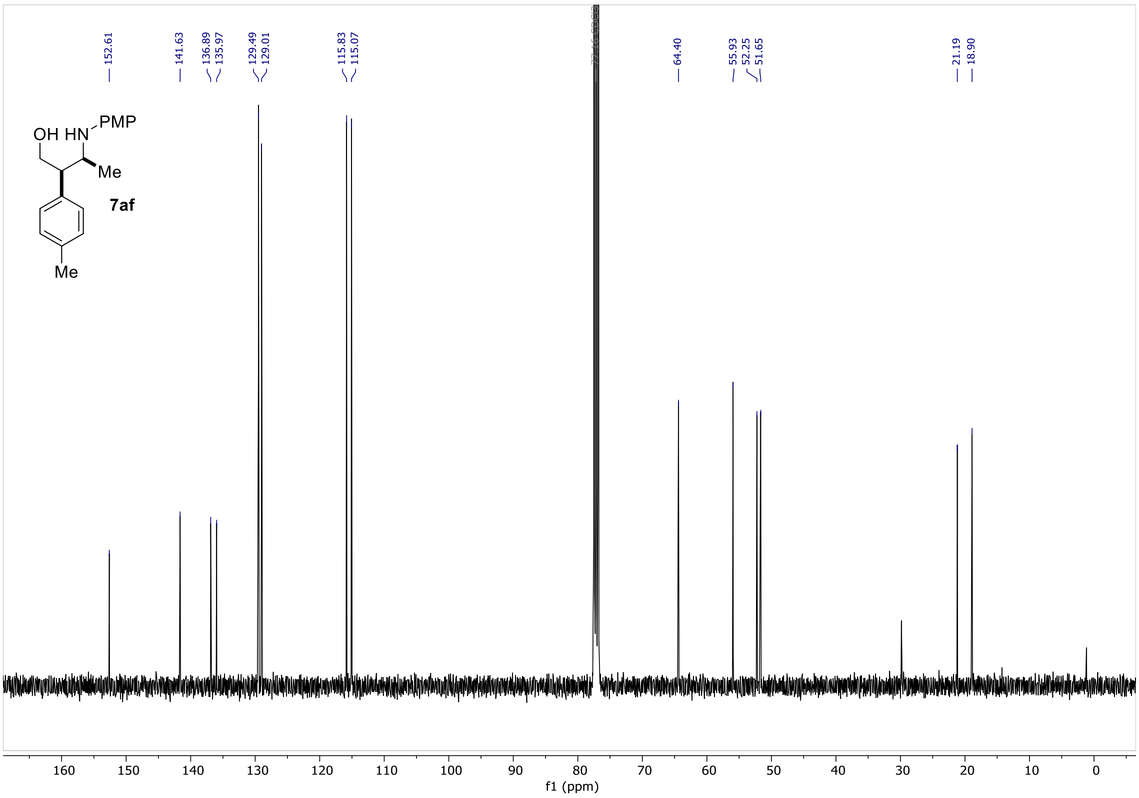

3-((4-Methoxyphenyl)amino)-2-(4-(trifluoromethyl)phenyl)butan-1-ol (7ah)

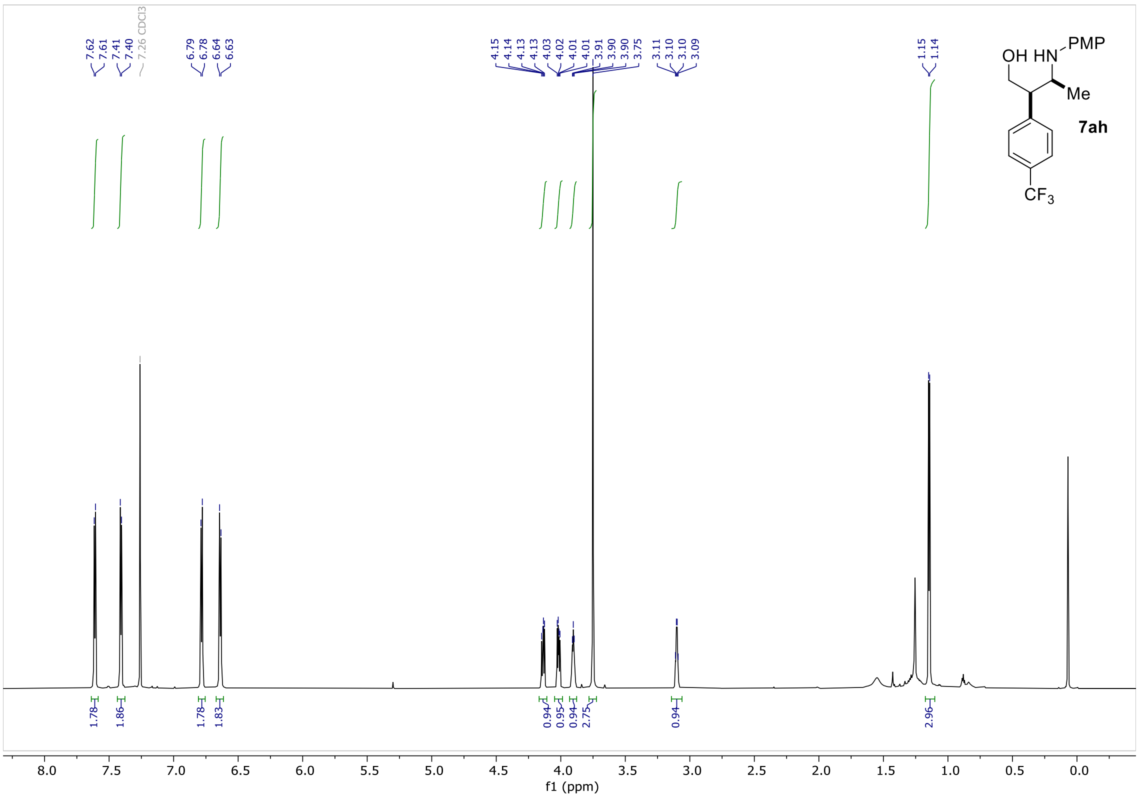

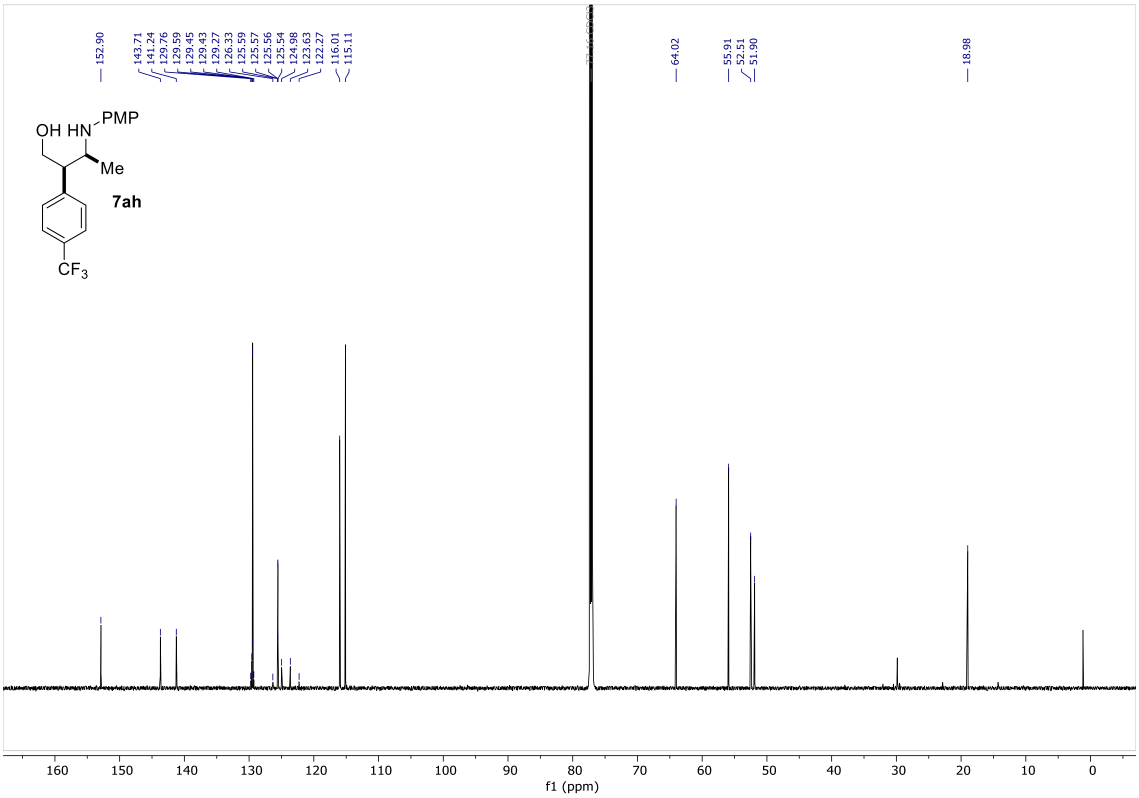

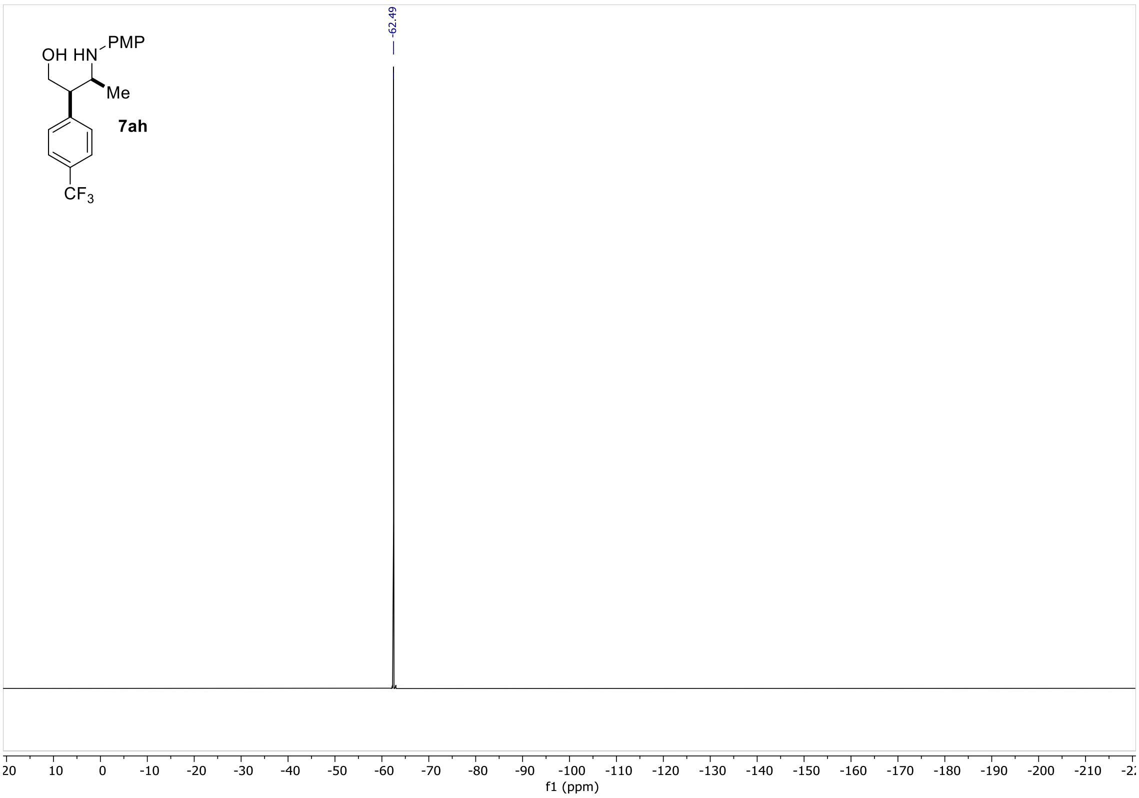

1-(4-Methoxyphenyl)-2-methyl-3-(4-(trifluoromethyl)phenyl)azetidine (8)

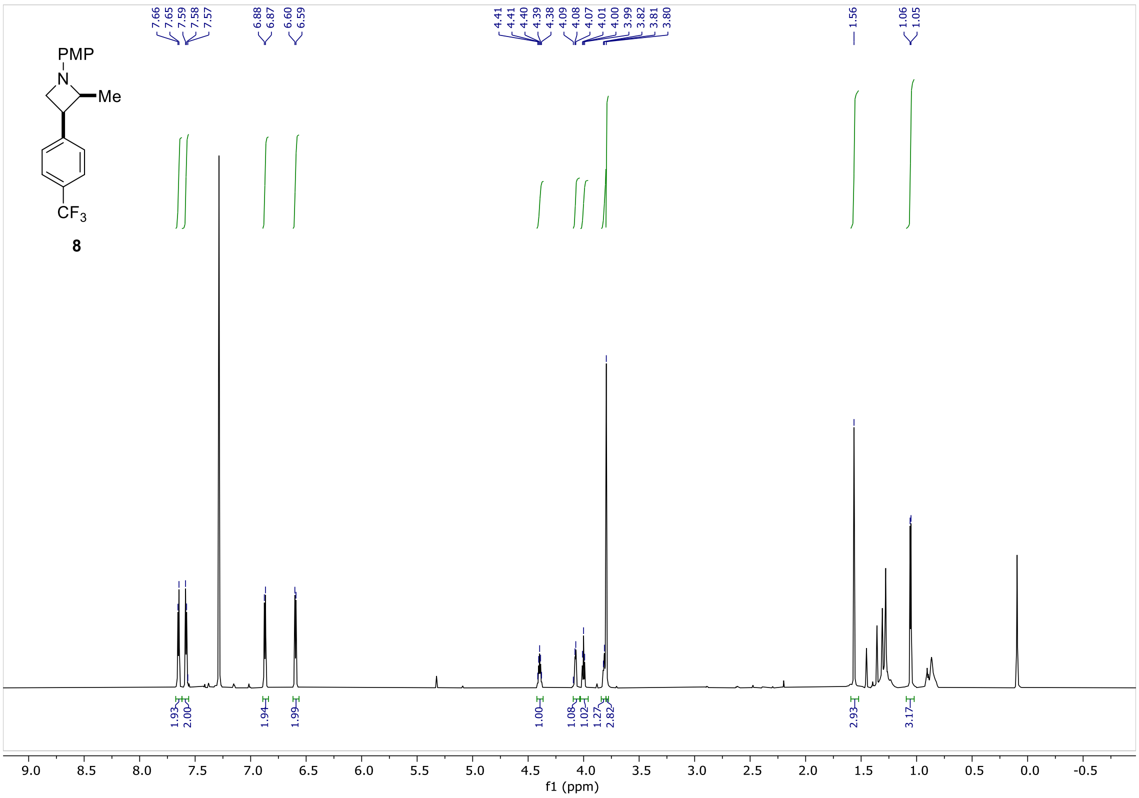

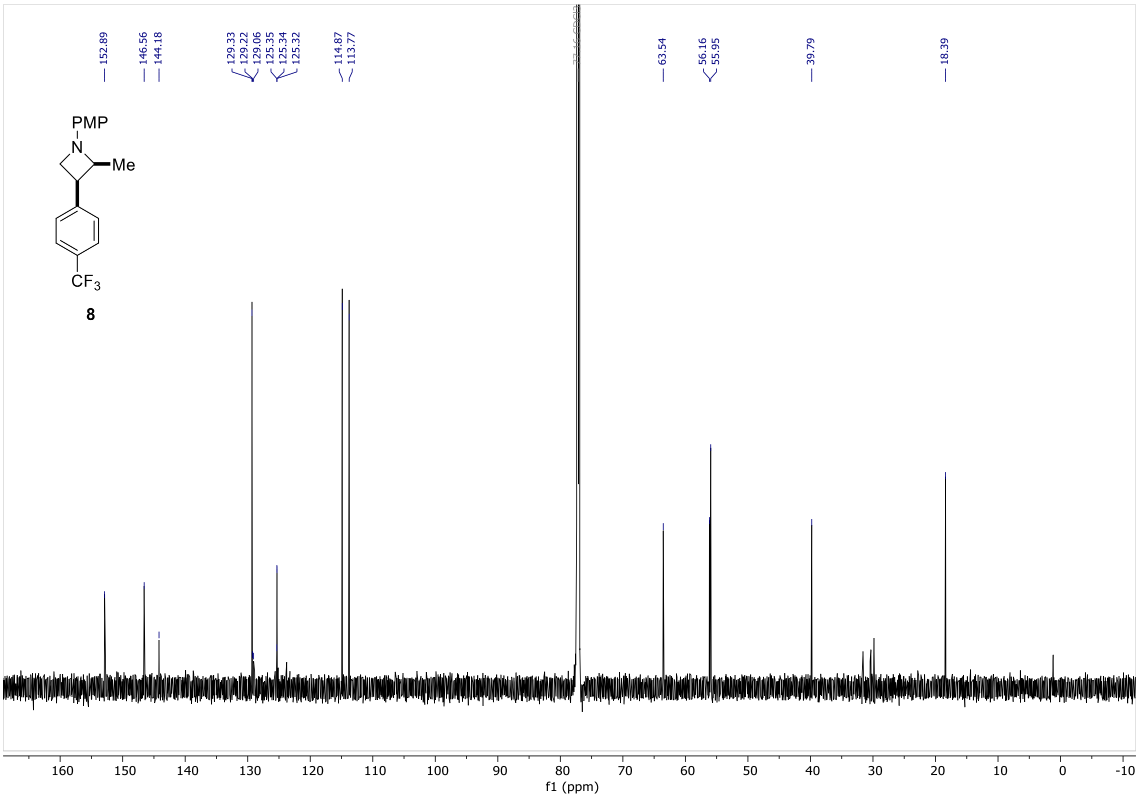
152.89
146.56
144.18
129.33
129.22
129.06
125.35
125.34
125.32
114.87
113.77
63.54
56.16
55.95
39.79
18.39
PMP
N
Me
CF3
8
160 150 140 130 120 110 100 90 80 70 60 50 40 30 20 10 0 -10
f1 (ppm)

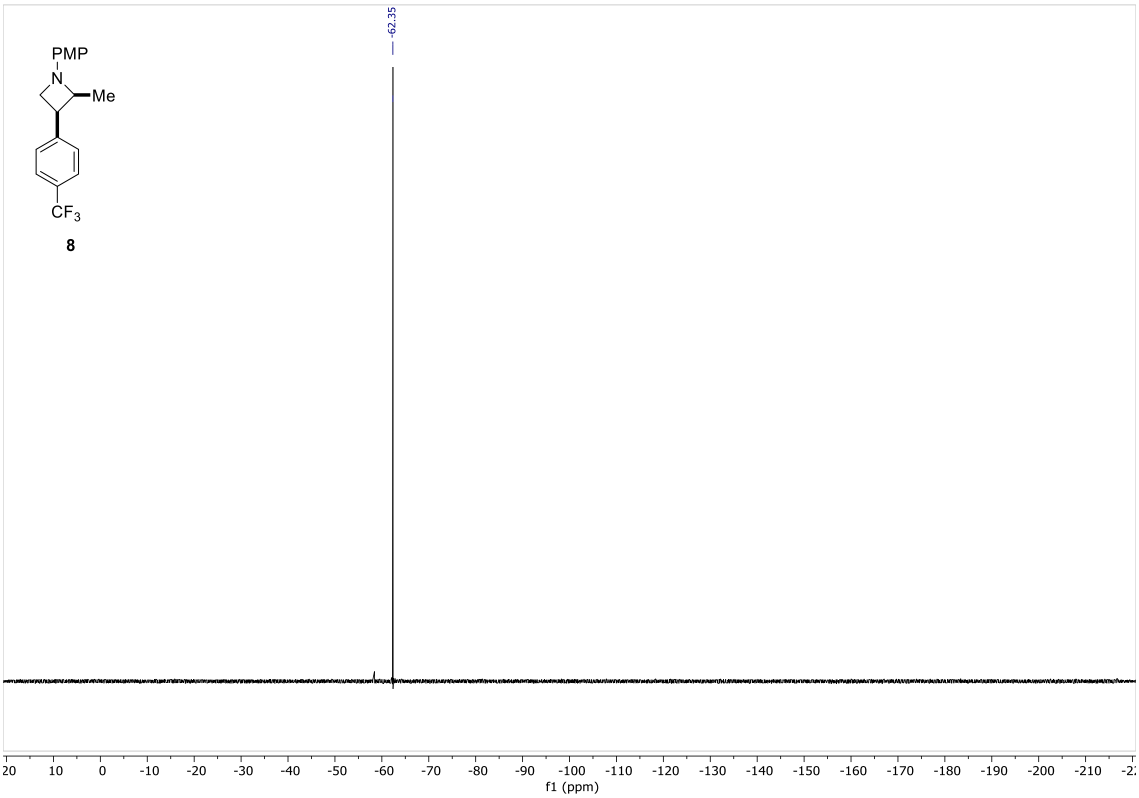
-62.35
PMP
N
Me
CF3
8
20 10 0 -10 -20 -30 -40 -50 -60 -70 -80 -90 -100 -110 -120 -130 -140 -150 -160 -170 -180 -190 -200 -210
f1 (ppm)

2-Amino-3-(*p*-tolyl)butan-1-ol (15)

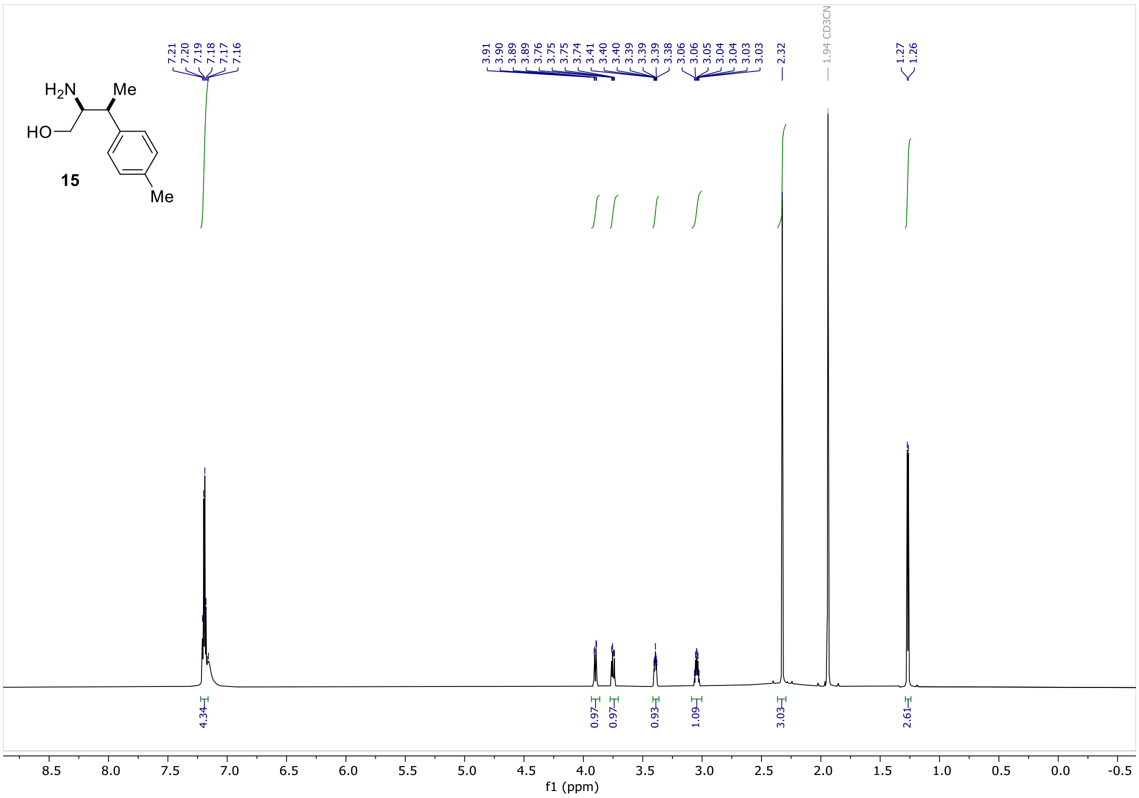

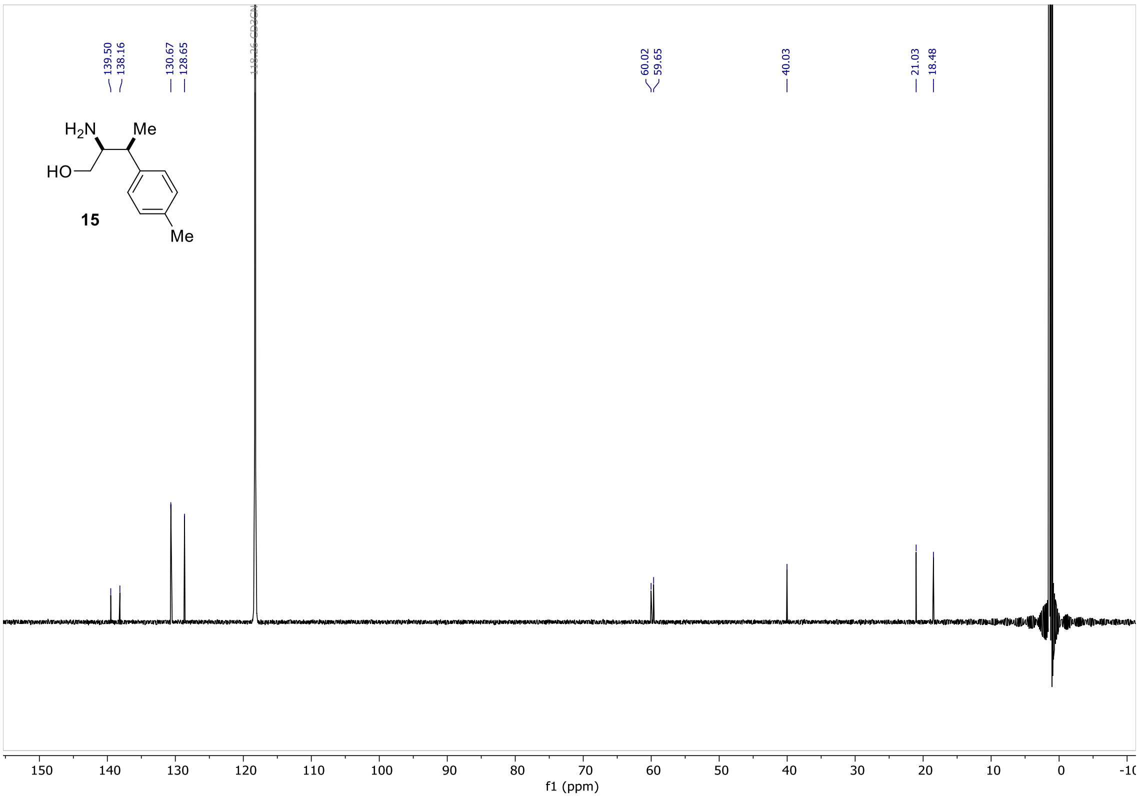

3-Fluoro-4-hydroxy-3-(*p*-tolyl)butan-2-one (10)

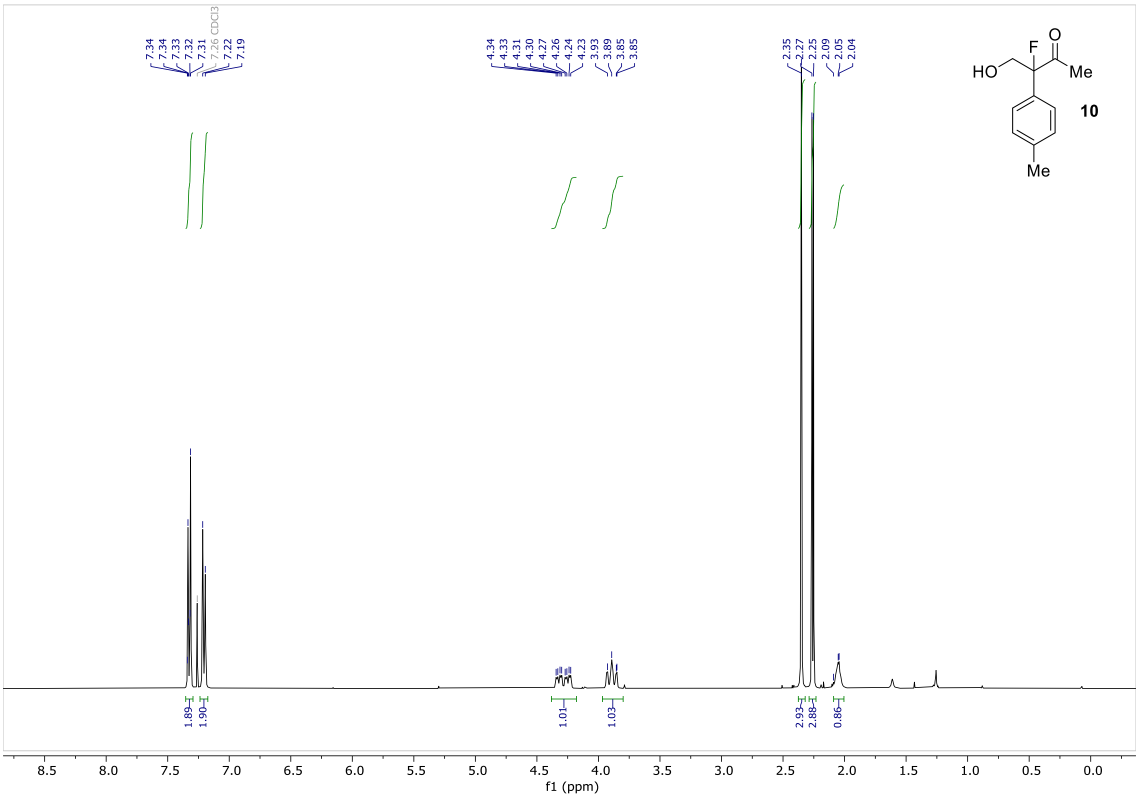

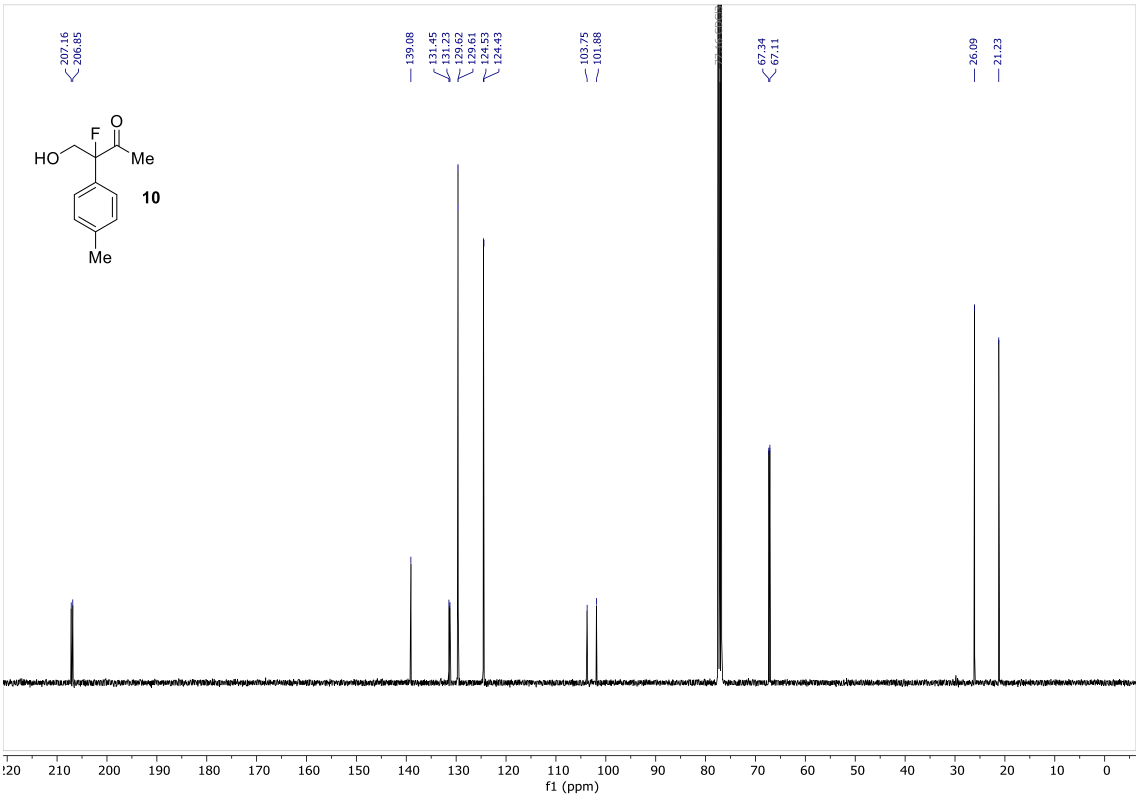

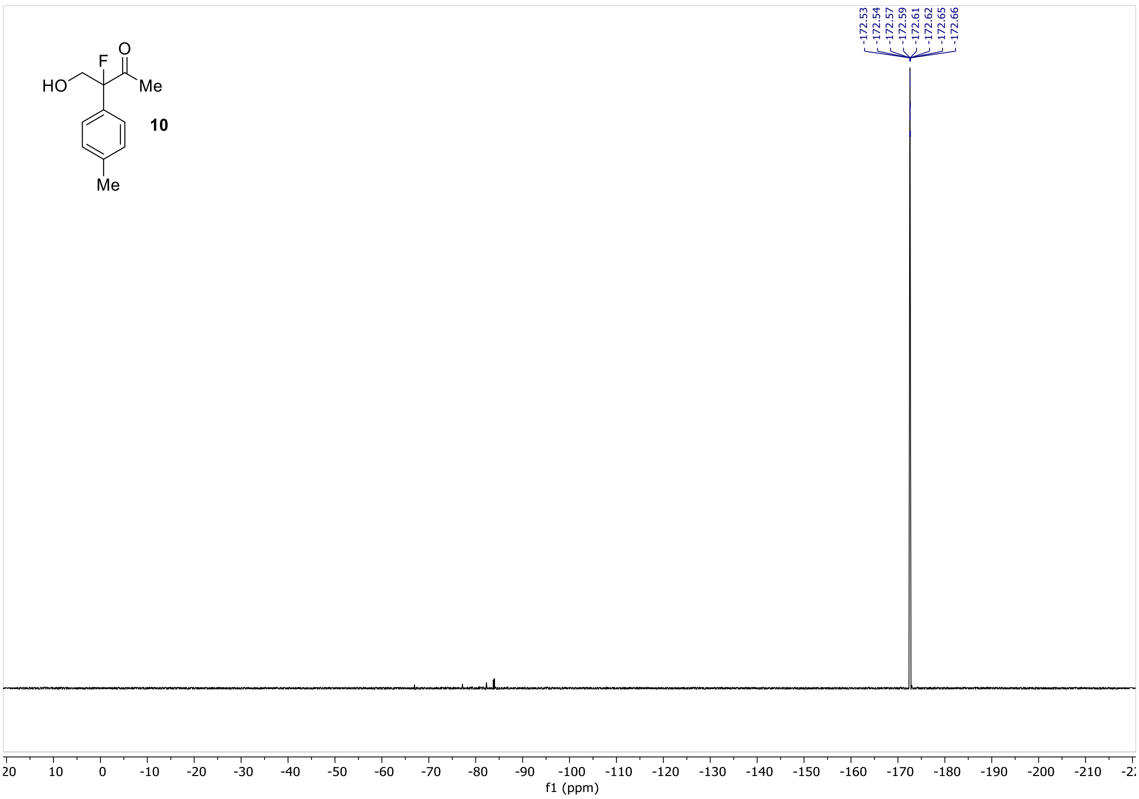

5-Fluoro-3-(4-methoxyphenyl)-4-methyl-5-(*p*-tolyl)-2-(trifluoromethyl)-1,3-oxazinane (11)

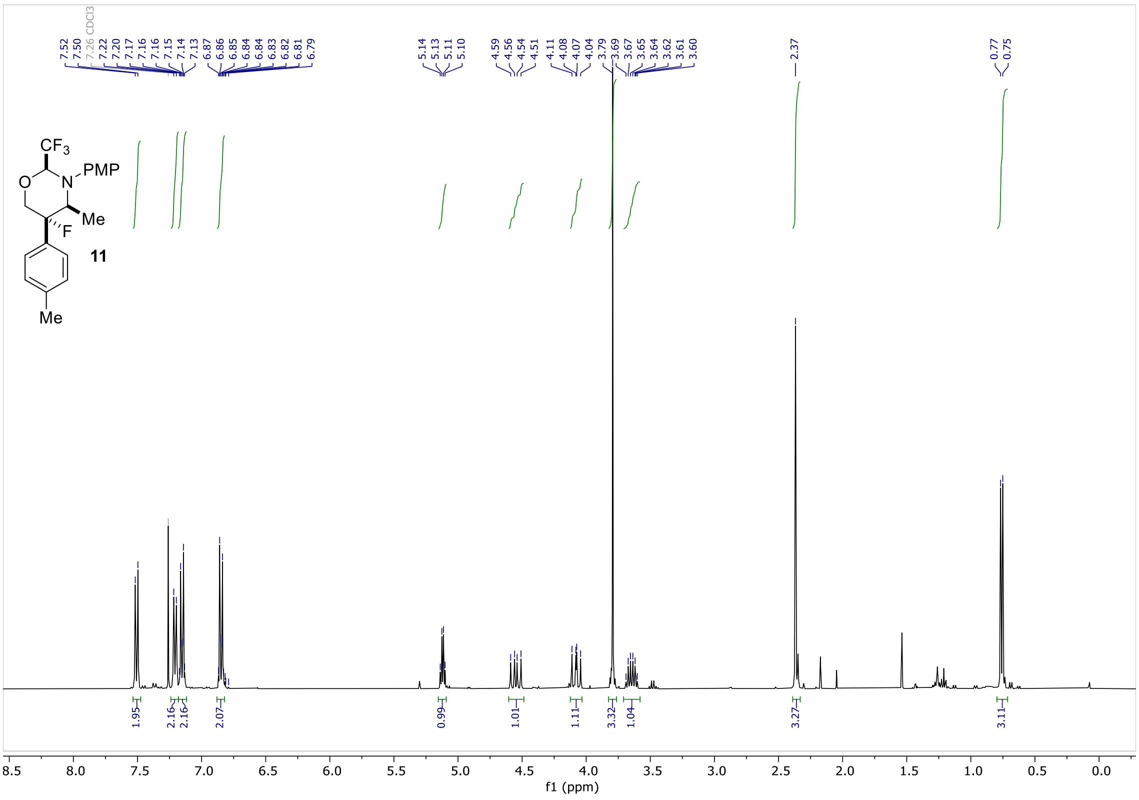

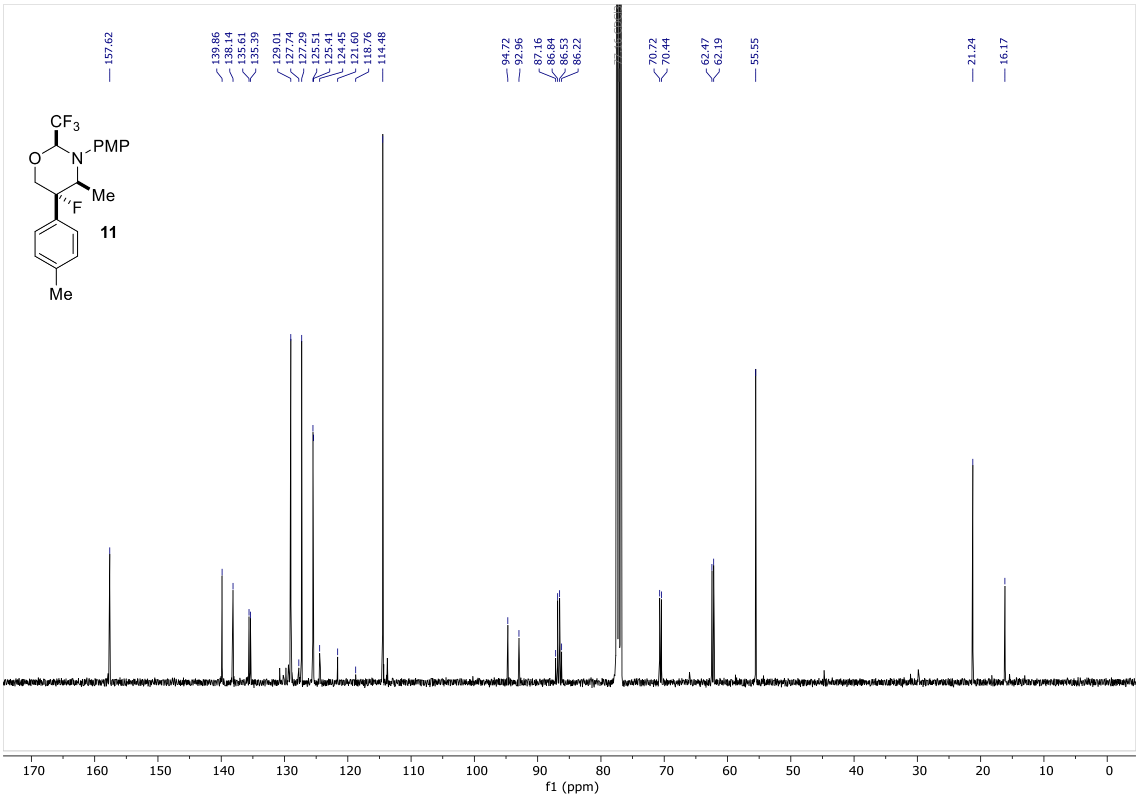
157.62
139.86
138.14
135.61
135.39
129.01
127.74
127.29
125.51
125.41
124.45
121.60
118.76
114.48
94.72
92.96
87.16
86.84
86.53
86.22
70.72
70.44
62.47
62.19
55.55
21.24
16.17
CF3
O
N
PMP
Me
F
11
Me
170
160
150
140
130
120
110
100
90
80
70
60
50
40
30
20
10
0
f1 (ppm)

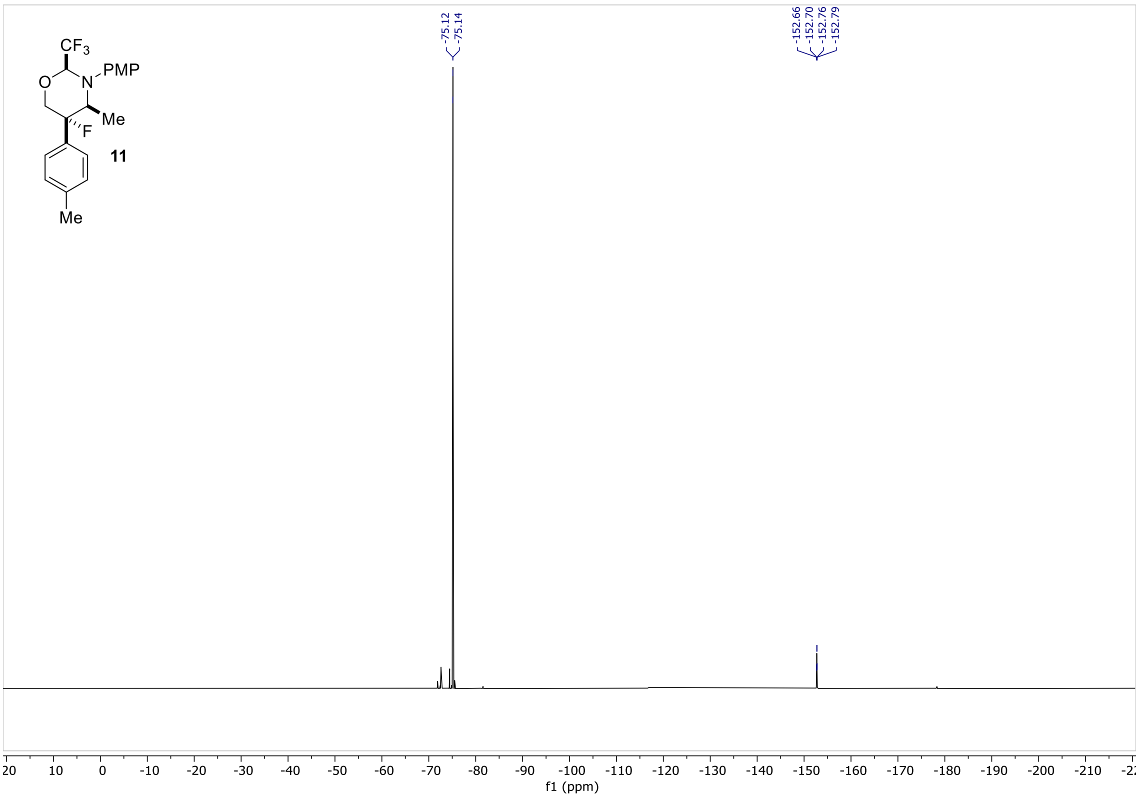
-75.12
-75.14
-152.66
-152.70
-152.76
-152.79
CF3
O
N
PMP
Me
F
11
Me
20
10
0
-10
-20
-30
-40
-50
-60
-70
-80
-90
-100
-110
-120
-130
-140
-150
-160
-170
-180
-190
-200
-210
f1 (ppm)

2-Fluoro-3-((4-methoxyphenyl)amino)-2-(*p*-tolyl)butan-1-ol (12)

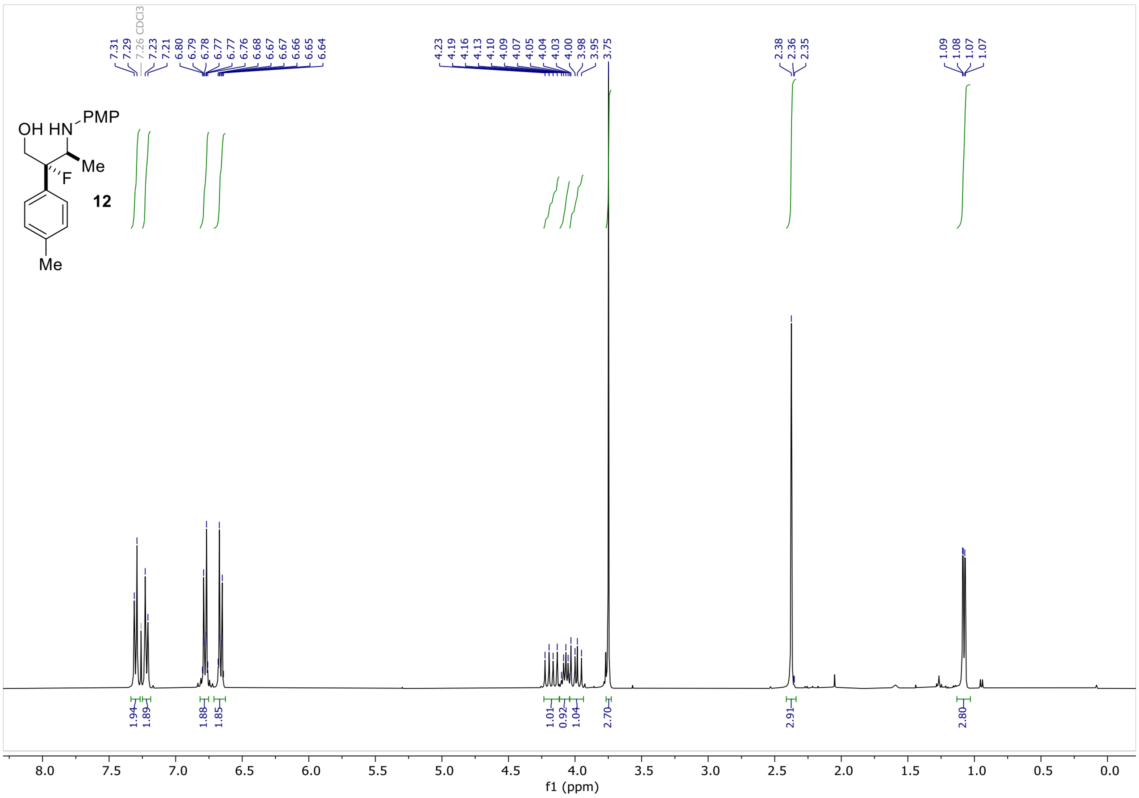

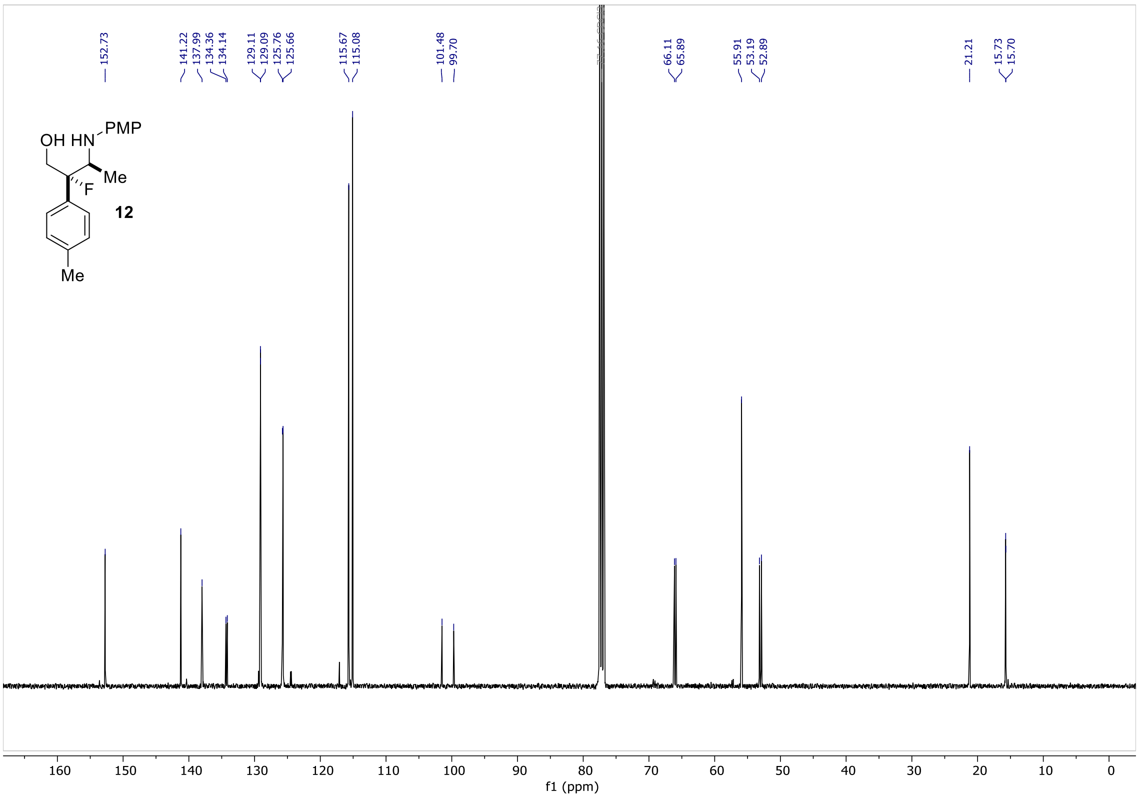

Supplement: Supplementary file 1 — Supporting Information [file ANIE-63-e202411383-s001.pdf]
